# Synthesis of 5,5,6-Fused Tricyclic Lactones: Stereocontrol of Three Consecutive Stereocenters

**DOI:** 10.1021/acs.orglett.5c05086

**Published:** 2026-01-12

**Authors:** Anass Ziari, Ivana Císařová, Eliška Matoušová

**Affiliations:** † Department of Organic Chemistry, Faculty of Science, 37740 Charles University, Hlavova 8, 128 00 Praha 2, Czech Republic; ‡ Department of Inorganic Chemistry, Faculty of Science, 37740 Charles University, Hlavova 8, 128 00 Praha 2, Czech Republic

## Abstract

We report a new, highly diastereoselective method for the synthesis of strained 5,5,6-fused tricyclic γ-lactones via a palladium-catalyzed tandem Heck/Suzuki cross-coupling reaction, followed by epoxidation and lactone-forming epoxide opening. Using this sequence, we prepared tricyclic γ-lactones with three consecutive stereocenters from simple, readily available starting materials, including one derived from enantiomerically pure biomass-based levoglucosenone. Our method provides a stereocontrolled approach to complex scaffolds of potential relevance in synthetic and medicinal chemistry.

## Graphical abstract

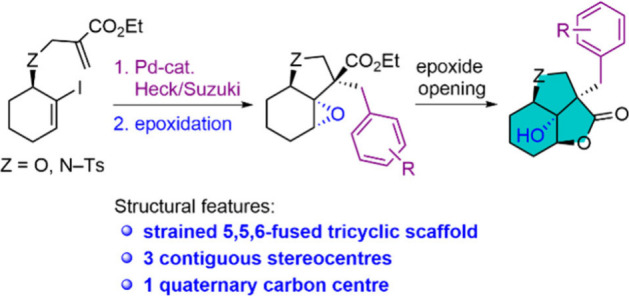

Natural products with a strained 5,5,6-fused tricyclic core, such as (−)-galiellalactone (Figure 1A), have attracted significant attention for their potent bioactivities. Galiellalactone, isolated from *Galiella rufa* fungus,^1^ contains an α,β-unsaturated lactone acting as a Michael acceptor and shows promise in prostate cancer therapy.^2,3^ It has also been patented for anti-inflammatory uses^4^ and studied for anti-HIV potential.^5^ Related biologically relevant compounds include diocollettines A^6^ and rehmaglutin B,^7^ which exhibit cytotoxicity^8^ and inhibition of nitric oxide production,^9^ respectively.

**Figure 1:**
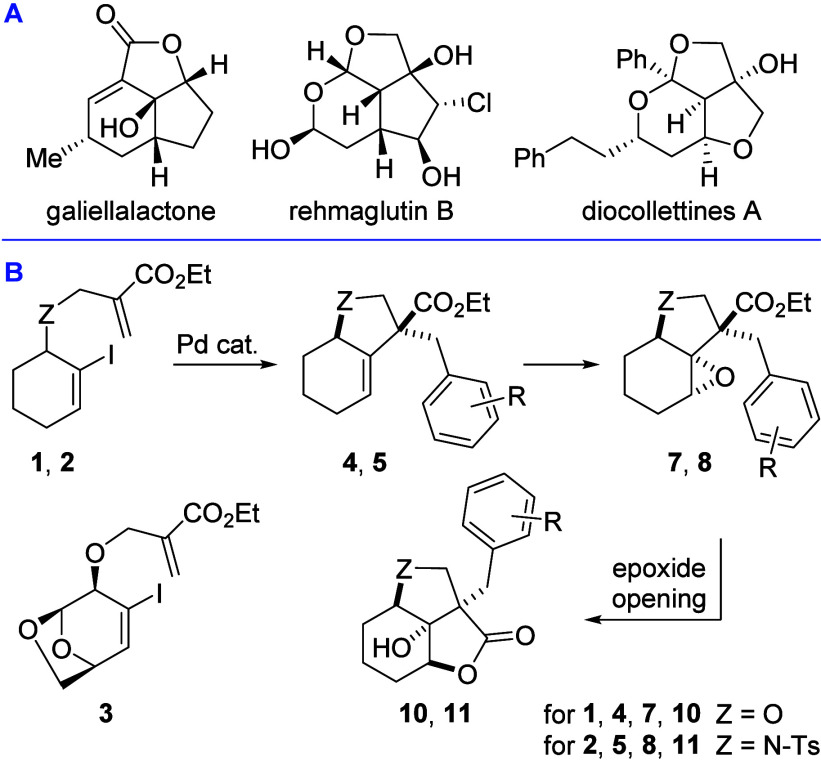
A. Bioactive 5,5,6-tricyclic natural products. B. Synthetic plan.

Sterner’s first synthesis of (−)-galiellalactone^10^ established its absolute configuration through the synthesis of opposite enantiomer^11^ but suffered from moderate diastereoselectivity of epoxidation. Subsequent syntheses of analogues encountered low yields in the epoxide opening step.^12^ An alternative route introduced the hydroxyl group by allylic (Riley) oxidation.^13^

Motivated by the biological relevance and synthetic challenges of this scaffold, and extending our palladium-catalyzed tandem protocols for polycycle synthesis,^14,15^ we sought a concise route to 5,5,6-fused tricyclic γ-lactones. Our strategy combines a tandem Heck/Suzuki cross-coupling with epoxidation and lactone-forming epoxide opening to prepare three consecutive stereocenters, including a quaternary carbon (Figure 1B). This approach would offer a stereocontrolled route to complex polycyclic frameworks for subsequent functional investigations.

Starting materials **1**–**3** were synthesized by alkylation or Mitsunobu reaction of the corresponding alcohols (see Supporting Information (SI)) and subjected to the palladium-catalyzed tandem reaction with arylboronic acids. Optimization on substrate **1** with phenylboronic acid surveyed various Pd catalysts, ligands, solvents, and temperature (Table 1). Reactions with Pd­(PPh_3_)_4_ and Pd­(OAc)_2_/P­(*o*-tol)_3_ showed modest yields (5–49%) depending on solvent used (entries 1–3). Ligand-free conditions with Pd_2_(dba)_3_ in EtOH at room temperature for 16 h furnished the product in 40% yield (entry 4). Notably, increasing the reaction temperature to 80 °C not only substantially improved the yield to 96% in 5 h (entry 10) but also led to a very clean transformation.

**Table 1:**
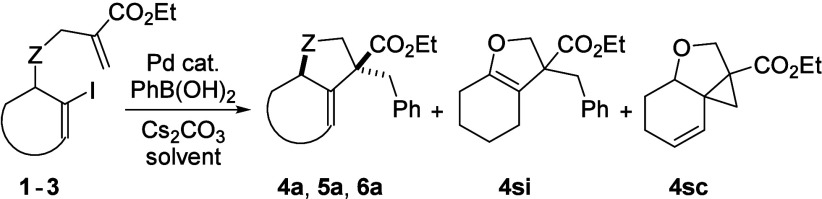
Optimization of the Tandem Reaction

Entry	Catalyst^a^	Solvent	Temp. [°C]	Time	Yield^b^ [%]
1, **1**	Pd(PPh_3_)_4_	DMF/H_2_O^c^	70	16 h	5
2, **1**	Pd(OAc)_2_/P(*o*-tol)_3_	MeCN	r.t. to 85	24 h	13
3, **1**	Pd(OAc)_2_/P(*o*-tol)_3_	EtOH	80	24 h	49
4, **1**	Pd_2_(dba)_3_	EtOH	r.t.	16 h	40
5, **1**	Pd_2_(dba)_3_	EtOH	80^d^	12 min	77^e^
6, **1**	Pd_2_(dba)_3_	EtOH	80^d^	5 min	74^e^
7, **1**	Herrmann catalyst	EtOH	80^d^	5 min	59^e^
8, **1**	Pd(OAc)_2_ /P(BTF)_3_ ^f^	EtOH	80^d^	10 min	19^e^
9, **1**	Pd(OAc)_2_ /TFP^g^	EtOH	80^d^	10 min	0
10, **1**	Pd_2_(dba)_3_	EtOH	80	5 h	96^h^
11, **2**	Pd_2_(dba)_3_	EtOH	80	5 h	98^h^
12, **3**	Pd_2_(dba)_3_	EtOH	80	4.5 h	traces
13, **3**	Pd(PPh_3_)_4_	toluene/H_2_O	60	5 h	44

a 5 mol % Pd used (2.5 mol % for Pd_2_(dba)_3_); 10 mol % ligand.

b ^1^H NMR yields of **4a**, **5a** (entry 11), or **6a** (entries 12, 13) using 3,4,5-trichloropyridine as internal standard.

c Ratio 4:1.

d Microwave irradiation.

e Side products **4si** and **4sc** formed in <15% combined yield.

f Tris­[3,5-bis­(trifluoromethyl)­phenyl]­phosphine.

g Tri­(2-furyl)­phosphine.

h Isolated yield.

Applying these optimized conditions to substrate **2** was equally effective, affording product **5a** in 98% isolated yield (entry 11). In contrast, for compound **3**, only traces of **6a** were obtained under the same conditions (entry 12). Substituting Pd_2_(dba)_3_ with Pd­(PPh_3_)_4_ in a toluene/H_2_O mixture at 60 °C, however, resulted in 44% yield (entry 13). These findings show the influence of substrate structure on the reaction outcome and highlight the need for condition adjustment.

To shorten the reaction time, we also explored the use of microwave irradiation. Under the optimized conditions, the desired product was obtained in 77% yield within 12 min or 74% yield in 5 min (entries 5 and 6), accompanied by side products **4si** and **4sc**. The former resulted from isomerization of product **4a**, while the latter was formed by a described Heck/Heck transformation^16,17^ of substrate **1**. These two side products were repeatedly observed in low quantities across all microwave-assisted experiments.

Other catalytic systems did not improve the results. Using the Herrmann catalyst gave **4a** in 59% yield, and Pd­(OAc)_2_ with other ligands resulted in only low to negligible yields (entries 7–9). Of the bases tested, none outperformed Cs_2_CO_3_ (see SI, Table SI1 for additional experiments).

Regarding the stereochemistry, only one diastereomer was observed for all products. This selectivity likely arises from the requirement for coplanarity of the reacting double bond with the C–Pd bond during carbopalladation.^18^ In the case of our substrates **1**–**3**, only one fully eclipsed conformation **13** is geometrically possible due to the shape of the substrate and the formation of a bicyclic product (Scheme 1), leading to the 5-exo products **4**–**6** with the indicated relative configuration, consistent with prior literature.^19,20^ We confirmed it in subsequent synthetic steps by the X-ray analysis of compounds **9a** and **10b** (SI, Figure S1 and Table 3). While the alternative 6-endo pathway might adopt a nearly eclipsed conformation, it is less favorable. Steric effects also favor the 5-exo mode, especially in intermolecular reactions,^21^ yet conformational constraints appear to dominate here.

**Scheme 1:**
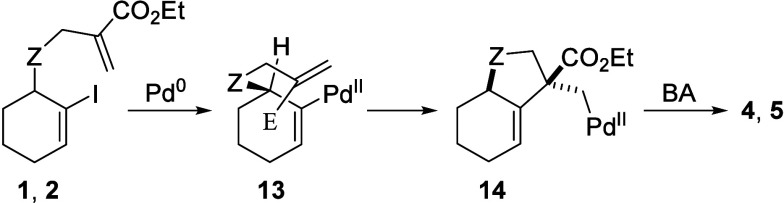
Rationalization of the Relative Configuration of Compounds **4** and **5**

Experiments with other boronic acids are shown in Table 2 and were carried out using Pd_2_(dba)_3_ in EtOH at either room temperature or 80 °C, employing both conventional and microwave heating. The reactions were performed in anhydrous EtOH for consistency, however, the yields were comparable with undried EtOH.

**Table 2:**
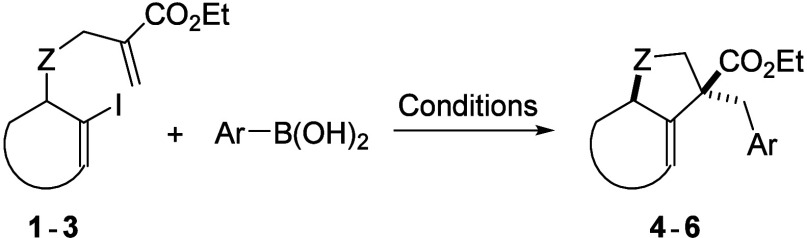
Substrate Scope of the Tandem Reaction^a^

Entry	Ar	Product	Yield [%] at r.t.^b^ ^,^ ^c^	Yield [%] at 80 °C^b^ ^,^ ^d^	Yield [%] in MW^b^ ^,^ ^e^
1, **1**	Ph	**4a**	40	96^f^	77
2, **1**	3,4-(OCH_2_O)-C_6_H_3_	**4b**	80^d^	(36)	(60)
3, **1**	4-MeO-C_6_H_4_	**4c**	41	85	46
4, **1**	4-CF_3‑_C_6_H_4_	**4d**	27	(21)	(30)
5, **1**	4-Me-C_6_H_4_	**4e**	–	85	40
6, **1**	2-benzofuranyl	**4f**	–	0	55
7, **1**	2-Cl-C_6_H_4_	**4g**	–	traces	30 (34)
8, **1**	2-Cl, 4-F-C_6_H_3_	**4h**	–	traces	traces
9, **1**	2-furyl	**4i**	–	0	traces
10, **1**	3-(2-bromopyridyl)	**4j**	0	–	–
11, **1**	2-MeO-C_6_H_4_	**4k**	-	60	-
12, **1**	4-pyridyl	**4l**	0	17^g^	-
13, **1**	3-thienyl	**4m**	-	81	-
14, **1**	3,5-Me_2_C_6_H_3_	**4n**	-	98	-
15, **1**	4-OH-C_6_H_4_	**4o**	-	traces	-
16, **1**	4-Ac-C_6_H_4_	**4p**	-	71	-
17, **1**	benzyl	**4q**	-	traces	-
18, **1**	5-pyrimidinyl	**4r**	-	0	-
19, **2**	Ph	**5a**	–	98^f^	–
20, **2**	4-MeO-C_6_H_4_	**5c**	–	72^f^	–
21, **2**	4-Me-C_6_H_4_	**5e**	-	82	-
22, **3**	Ph	**6a**	0	(44)^h^	–

a Standard conditions: Pd_2_(dba)_3_ (2.5 mol %), Cs_2_CO_3_ (2 equiv), boronic acid (1.5 equiv), anhydrous EtOH.

b Isolated yields. ^1^H NMR yields in parentheses (3,4,5-trichloropyridine used as internal standard).

c For 24 h.

d For 16 h.

e Microwave-irradiated (80 °C, 12 min).

f For 5 h.

g Side product **4sl** formed in ∼40% yield.

h Conditions: Pd­(PPh_3_)_4_ (5 mol %), Cs_2_CO_3_ (2 equiv), boronic acid (2 equiv), toluene/H_2_O (4:1), 60 °C, 5 h.

For electron-rich boronic acids, conventional heating proved most effective (as was also the case for phenylboronic acid, which is included again as entry 1 for comparison), affording products **4c**, **4e**, **4k**, and **4n** in 85%, 85%, 60%, and 98% yields, respectively (entries 3, 5, 11, and 14). This also shows that the reaction tolerates *ortho*-substituted aromatic rings. In the case of 3,4-methylenedioxyphenylboronic acid, however, the highest yield (80%) was obtained at room temperature (entry 2), likely due to a faster formation of side products^22^ at elevated temperatures, including a homocoupling of **4sc** and **4si** products. Substrates bearing a free hydroxy or a nonaromatic benzyl substituent gave only trace amounts of the corresponding products **4** in complex reaction mixtures (entries 15 and 17).

In contrast, electron-deficient 4-trifluoromethyl- and 2-chloro-substituted arylboronic acids gave **4d** and **4g** in approximately 30% yields upon microwave irradiation, with reduced product formation on conventional heating (entries 4, 7). The more electron-poor 2-chloro-4-fluorophenylboronic acid was significantly less reactive, with only trace amounts of **4h** detected (entry 8). The 4-acetyl-substituted boronic acid, on the other hand, afforded product **4p** in 71% at 80 °C (entry 16).

Among heteroaryl boronic acids, only benzofuranyl (**4f**) and thienyl (**4m**) derivatives were obtained in good isolated yields, 55% and 81% (entries 6, 13). By contrast, nitrogen-containing pyridyl (**4l**, **4j**; entries 10, 12) and pyrimidinyl (**4r**; entry 18) substrates were inefficient, yielding only trace product or none. For **4l**, we observed the formation of 2-(pyridin-4-yl)­cyclohex-2-en-1-ol (see SI) along with other side products. These results suggest that basic nitrogen coordination perturbs the catalytic process or deactivates the catalyst, unlike with nonbasic heterocycles.

The substrate effect on the tandem reaction was examined next. *N*-Derivative **2** reacted smoothly with phenylboronic acid to give **5a** in a near-quantitative yield (98%) and a clean crude product (entry 19). Reactions with 4-methoxy- and 4-methylphenylboronic acids afforded cyclized products **5c** and **5e** in 72% and 82% yields, respectively, at 80 °C (entries 20 and 21). However, substrate **3** gave only negligible yields of **6a** with phenylboronic acid under the original conditions at room temperature or 80 °C, and only switching the catalytic system increased the yield to 44% (entry 22 and SI, Table S2). These results indicate that reaction efficiency depends on both the substrate and the electronic nature of the boronic acid, which are crucial in the transmetalation step.

With cyclized alkenes **4**–**6** in hand, we evaluated the epoxidation and acid-mediated lactonization (Figure 1B). For epoxidation, *m*-CPBA outperformed H_2_O_2_, affording the corresponding epoxides **7**–**9** in moderate to good yields with high selectivity (a single diastereomer was formed). Single-crystal X-ray analysis of **9a** (Figure S1) confirmed its structure and absolute configuration, consistent with that of enantiopure substrate **3** synthesized from levoglucosenone.

Next, we optimized lactone formation. Lewis acids proved more effective than Bronsted acids: methylenedioxy-substituted lactone **10b** was formed in 64% yield using BF_3_·OEt_2_ and 82% with SnCl_4_, compared to 25% with sulfuric acid. Likewise, replacing BF_3_·OEt_2_ with TiCl_4_ increased the yield of electron-deficient **10d** from 40% to 84% (for full screening details, see SI, Table S3).

Application of these conditions across substrates is summarized in Table 3. For oxygen-containing derivatives, epoxidation gave 50–77% yields and SnCl_4_-mediated lactonization of 82–98%. The two nitrogen-containing compounds, **11a** and **11c**, were formed in very good yields on lactonization (91%, 92%) but were lower in the epoxidation step (47%, 45%).

**Table 3:**
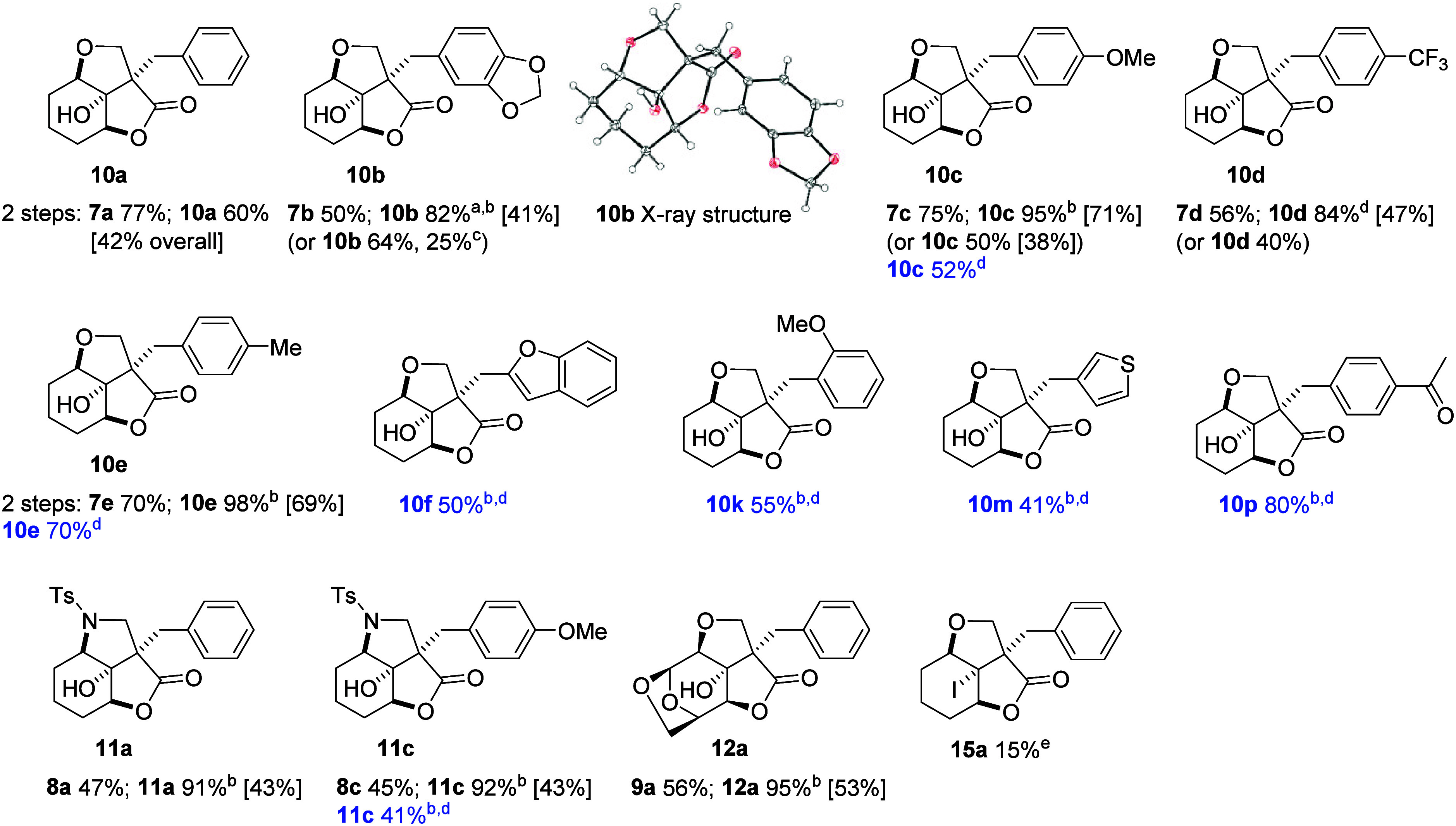
Substrate Scope of Epoxidation and Lactonization^a^

a ^1^H NMR yield (3,4,5-trichloropyridine used as internal standard).

b SnCl_4_ used for lactonization instead of BF_3_·OEt_2_.

c H_2_SO_4_ used for lactonization.

d Intermediate epoxide not purified.

e TiCl_4_ used for lactonization.

f Prepared by halolactonization with I_2_, CH_3_CN/H_2_O (2:1).

g Standard lactonization conditions: BF_3_·OEt_2_ (1.3 equiv), DCM.

For **10c** and **10e**, we also attempted the epoxidation/lactonization sequence without purifying the intermediate epoxides, using BF_3_·OEt_2_ in the second step. The yield of **10c** was thus improved from 38% to 52% compared to the previous protocol. For **10e**, the simplified method with BF_3_·OEt_2_ gave 70%, comparable to the 69% overall yield from the two-step sequence using SnCl_4_, in which lactonization was nearly quantitative. Compound **11c** was formed in similar yields by both methods when SnCl_4_ was used for lactonization. Substrates **4f**, **4k**, **4m**, and **4p** were only subjected to the simplified protocol, giving 41–80% yields and demonstrating applicability to oxygen- and sulfur-containing heterocycles and tolerance of a keto group.

We also explored lactone formation via halolactonization.^23^ Various reagents and temperatures were screened for substrates **4a**, **4b**, and **6a** (SI, Table S4). Most conditions failed, except treatment of alkene **4a** with six equivalents of iodine in a MeCN/H_2_O mixture at 110 °C, which afforded product **15a** in 15% yield. The relative configuration of the final products was corroborated by X-ray analysis of **10b** (Table 3).

Selected compounds (**9a**, **10a**, **10b**, **10c**, **10d**, **10e**, and **12a**) were tested for cytotoxic activity against five cell lines (four human cancer lines and normal dermal fibroblasts) but showed no significant activity (see SI for details).

In summary, we developed a stereoselective route to strained 5,5,6-fused tricyclic γ-lactones, achieving high diastereoselectivity across diverse substrates. Lactones **10**–**12** were synthesized in good yields, particularly in the lactone-forming step, often near-quantitative with SnCl_4_. The epoxidation/lactonization sequence can be efficiently performed without purifying the epoxide intermediates, affording higher yields than the protocol involving their isolation. In contrast, the alternative halocyclization pathway proved unsuccessful, with a maximum yield of 15%. The relative configuration of the products was confirmed by X-ray analysis of compounds **9a** and **10b**. Although cytotoxicity screening against five cell lines revealed no significant activity, the structural complexity and modularity of the method suggest potential for further derivatization and biological evaluation.

## Supplementary Material



## Data Availability

The data underlying this study are available in the published article and its Supporting Information.

## Notes

Additional optimization experiments, experimental procedures, characterization data for new compounds, X-ray and biological screeening details, and copies of NMR spectra (PDF)
