# Supplementary material for: Synthesis of 5,5,6-Fused Tricyclic Lactones: Stereocontrol of Three Consecutive Stereocenters
Source: Org Lett. 2026 Jan 12;28(3):1031–5. doi: 10.1021/acs.orglett.5c05086 (PMC12836344; doi:10.1021/acs.orglett.5c05086)
Supplement: Supplementary file 1 [file ol5c05086_si_001.pdf]

Supporting information for

Synthesis of 5,5,6-Fused Tricyclic Lactones: Stereocontrol of Three Consecutive Stereocentres

Anass Ziari,[a] Ivana Císařová[b] and Eliška Matoušová[a]*

[a] *Department of Organic Chemistry, Faculty of Science, Charles University, Hlavova 8, 128 00 Praha 2, Czech Republic*
E-mail: eliska.matousova@natur.cuni.cz

[b] *Department of Inorganic Chemistry, Faculty of Science, Charles University, Hlavova 8, 128 00 Praha 2, Czech Republic*

Contents

Contents 1

1. General information. 2

2. Synthesis of starting materials 3

2.1. Synthesis of compounds 1 and 2 3

2.2. Synthesis of compound 3 4

2. Tandem carbopalladation/Suzuki cross-coupling reaction 6

3.1. Optimisation with phenyl boronic acid 6

- General procedure A with MW irradiation 6
- General procedure B without MW irradiation 7

3.2. Optimisation of the tandem reaction with levoglucosenone-based compound 3 and phenylboronic acid 15

3. Formation of lactones 17

- General procedure for epoxidation 18
- General procedure for lactone formation *via* epoxide opening 22
- General procedure C for lactone formation *via* epoxide opening 22

4.2. Lactone formation *via* halolactonisation 28

4. X-ray structure data of compounds 9a and 10b 30

5. Cytotoxicity screening 33

6. References 34

7. Copies of ^{1}H and ^{13}C NMR spectra 35

1. General information.

Unless otherwise stated, the following general procedures were used for reactions. Commercially available compounds were used without further purification unless otherwise stated. Levoglucosenone was kindly provided by the Circa Group Pty Ltd (Australia). Tetrahydrofuran (THF) was purified and dried by distillation from sodium/benzophenone. Dry dichloromethane (DCM) and dry ethanol were purchased from Acros Organics and used without purification. Analytical thin layer chromatography (TLC) was performed on Merck Silica gel 60 F_{254}-coated aluminium plates. After development, the TLC plates were visualised under UV light (254 nm) and/or using an appropriate staining reagent, followed by heating. The following stains were used: phosphomolybdate stain: $Ce(SO_4)_2 \cdot 4H_2O$ (2 g), $H_3[P(Mo_3O_{10})_4]$ (4g), H_2SO_4 (10 mL), H_2O (200 mL), and ninhydrin: ninhydrin (0.3 g), *n*-butanol (100 mL), acetic acid (3 mL) was added. Column chromatography was performed on Acros Silica gel 60A (35–70 µm). Proton (^1H) and carbon (^{13}C) NMR spectra were recorded on a Bruker AVANCE III 600, Bruker AVANCE III HD 400, and Bruker NEO 400 instruments. The residual signals from $CHCl_3$, δ 7.26 ppm and δ 77.16 ppm, were used as internal references for ^1H and ^{13}C chemical shifts, respectively. Chemical shifts are reported in parts per million (δ scale) downfield from tetramethylsilane, and coupling constants (*J*) are given in Hertz. Multiplicity is defined as s = singlet, d = doublet, t = triplet, q = quartet, m = multiplet, or their combination. Structural assignments were made with additional information from gCOSY, gHSQC, and gHMBC experiments. IR spectra were measured on a Thermo Nicolet AVATAR 370 FT-IR spectrometer on KBr tablets of the compounds via the DRIFT method. ESI or APCI mass spectra (high resolution) were measured using a QTOF mass spectrometer (Bruker) equipped with an electrospray ion source. Microwave-assisted reactions were carried out in sealed vials using a CEM Discover SP + Explorer reactor. Reactions were heated to a setpoint of 80 °C with a rapid ramp, followed by an isothermal hold for the time indicated below. Temperature was monitored via the reactor's infrared sensor. Melting points were measured on a melting-point apparatus with a microscope and used without correction. Optical rotations were measured on AUTOMATIC POLARIMETER, Autopol III, are given in $deg \cdot mL \cdot g^{-1} \cdot dm^{-1}$ with accuracy ±2, and the mass concentrations (marked as c are given in g/100 mL).

2. Synthesis of starting materials

2.1. Synthesis of compounds 1 and 2

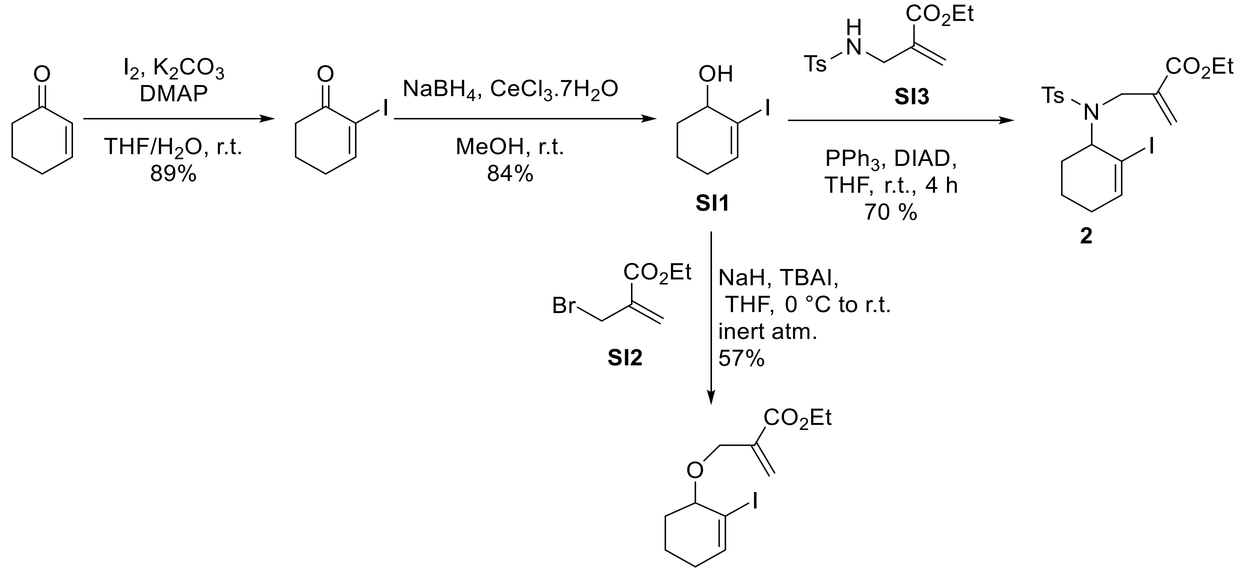

1

Ethyl 2-(((2-iodocyclohex-2-en-1-yl)oxy)methyl)acrylate (1).

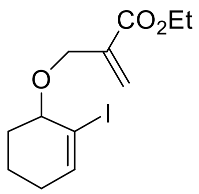

2-Iodocyclohex-2-en-1-ol[1] **(SI1)** (670 mg, 1.99 mmol) and tetrabutylammonium iodide (0.074 g, 0.198 mmol, 0.13 eq.) were dissolved in dry THF (5.94 mL) under an argon atmosphere. Ethyl 2-(bromomethyl)acrylate[2] (**SI2**) (0.459 g, 2.38 mmol, 1.2 eq.) was added, and the reaction mixture was cooled to 0 °C in an ice bath. NaH (60% dispersion in mineral oil, 0.143 g, 3.56 mmol, 1.8 eq.) was added in portions, and the reaction was stirred at 0 °C. After 20 min, it was allowed to warm to room temperature, and stirring was continued overnight. The reaction mixture was quenched with a saturated aqueous solution of NH_4Cl (10 mL) and extracted between brine (30 mL) and ethyl acetate (3 × 30 mL). The combined organic layers were dried over sodium sulfate, filtered, and concentrated under reduced pressure. The crude product was purified by column chromatography on silica gel (95/5 hexanes/ethyl acetate) to result in a yellow oil (253 mg, 57%).

^{1}H NMR (400 MHz, $CDCl_3$) δ 6.55 (ddd, J = 4.6, 3.5, 0.8 Hz, 1H), 6.33 (q, J = 1.4 Hz, 1H), 6.05 (q, J = 1.7 Hz, 1H), 4.36 (dt, J = 13.7, 1.5 Hz, 1H), 4.26 – 4.19 (m, 3H), 3.94 (m, 1H), 2.22 – 1.91 (m, 3H), 1.88 – 1.70 (m, 2H), 1.67 – 1.59 (m, 1H), 1.31 (t, J = 7.1 Hz, 3H). **^{13}C NMR** (101 MHz, $CDCl_3$) δ 166.1 142.1, 137.5, 126.5, 99.1, 79.6, 67.8, 60.8, 29.6, 29.2,

17.4, 14.3. **HRMS (ESI)** ***m/z:*** $[M + Na]^+$ calcd for $C_{12}H_{17}INaO_3$ 359.0115, found 359.0111. **IR (KBr) ν:** 2939, 1711, 1302, 1271, 1174 1155, 1088, 1068, 953, 752 cm^{-1}.

Ethyl 2-(((*N*-(2-iodocyclohex-2-en-1-yl)-4-methylphenyl)sulfonamido)methyl)acrylate (2).

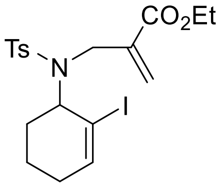

To a solution of 2-iodocyclohex-2-en-1-ol[1] **(SI1)** (500 mg, 2.36 mmol) in THF (72 mL) was added triphenylphosphine (621 mg, 2.95 mmol) and ethyl 2-(((4-methylphenyl)sulfonamido)methyl)acrylate[3] **(SI3)** (923 mg, 2.95 mmol). Once the solids were completely dissolved, DIAD (0.67 mL, 2.95 mmol) was added slowly at 0 °C in an ice bath. After warming up, the reaction mixture was stirred for 3 hours at room temperature, and the progress of the reaction was monitored by TLC. The solvent was then removed using a rotary evaporator. The crude product was purified by column chromatography on silica gel (15/1 hexanes/ethyl acetate), affording the product **2** as a yellowish oil (700 mg, 70%).

^{1}H NMR (400 MHz, $CDCl_3$) δ 7.78 – 7.72 (m, 2H), 7.31 – 7.27 (m, 2H), 6.67 – 6.63 (m, 1H), 6.43 – 6.41 (m, 1H), 6.09 – 6.07 (m, 1H), 4.51 – 4.44 (m, 1H), 4.37 – 4.29 (m, 1H), 4.20 (q, J = 7.1 Hz, 2H), 3.92 – 3.83 (m, 1H), 2.40 (s, 3H), 2.11 – 1.88 (m, 4H), 1.68 – 1.58 (m, 2H), 1.30 (t, *J* = 7.1 Hz, 3H). **^{13}C NMR** (101 MHz, $CDCl_3$) δ 166.2, 146.3, 143.6, 137.7, 137.6, 129.6 (2C), 128.1 (2C), 127.5, 97.0, 62.2, 61.0, 46.0, 32.5, 29.1, 21.7, 19.9, 14.3. **HRMS (ESI) m/z:** $[M + H]^+$ calcd for $C_{19}H_{25}INO_4S$ 490.0549, found 490.0540. **IR (KBr) ν:** 2935, 1706, 1157, 1122, 1093, 1034, 663, 600, 552, 534 cm^{-1}.

2.2. Synthesis of compound **3**

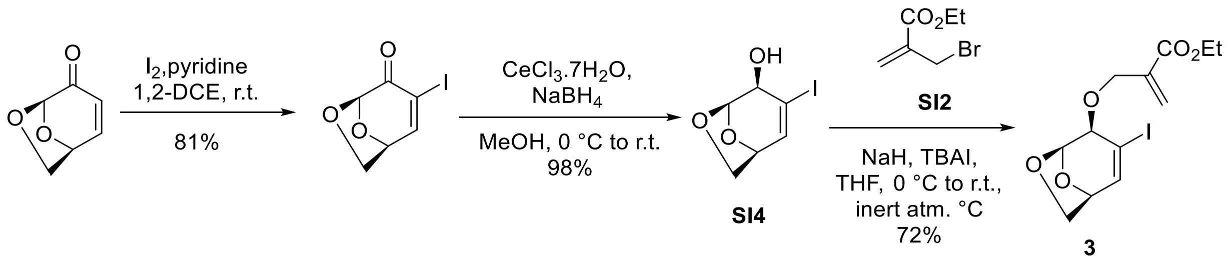

Ethyl 2-((((1*S*,4*R*,5*R*)-3-iodo-6,8-dioxabicyclo[3.2.1]oct-2-en-4-yl)oxy)methyl)acrylate (3).

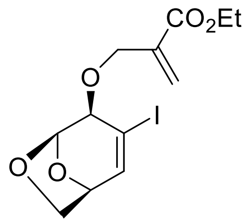

Alcohol **SI4**[1] (1.77 g, 6.99 mmol) and tetrabutylammonium iodide (0.13 eq., 0.9 mmol, 100 mg) were dissolved in dry THF (29.5 mL) under an argon atmosphere. Ethyl 2-(bromomethyl)acrylate[2] (**SI2**) (1.6 g, 8.39 mmol, 1.2 eq.) was added, and the reaction mixture was cooled to 0 °C in an ice bath. NaH (60% dispersion in mineral oil, 8.39 mmol, 335 mg, 1.8 eq.) was added in portions, and the reaction was stirred at 0 °C. After 20 min, it was allowed to warm to room temperature, and the stirring was continued overnight. The reaction mixture was quenched with a saturated aqueous solution of NH_4Cl (15 mL) and extracted between brine (30 mL) and ethyl acetate (50 mL) 3 times. The combined organic layers were dried over sodium sulfate, filtered, and concentrated under reduced pressure. The crude product was purified by column chromatography on silica gel (70/30 hexanes/ethyl acetate) to result in a sticky orange product (2.94 g, 72%).

Specific rotation $[\alpha]_D = -57.5^{\circ}$ (c 0.17, $CHCl_3$). **^{1}H NMR** (400 MHz, $CDCl_3$) δ 6.78 (dd, *J* = 4.8, 1.3 Hz, 1H), 6.37 – 6.35 (m, 1H), 6.09 – 6.06 (m, 1H), 5.61 (d, *J* = 2.5 Hz, 1H), 4.51 (t, *J* = 4.4 Hz, 1H), 4.47 – 4.45 (m, 2H), 4.23 (q, *J* = 7.2 Hz, 2H), 4.20 – 4.18 (m, 1H), 3.97 (d, *J* = 6.8 Hz, 1H), 3.70 (ddd, *J* = 7.0, 4.1, 1.3 Hz, 1H), 1.31 (t, *J* = 7.1 Hz, 3H). **^{13}C NMR** (101 MHz, $CDCl_3$) δ 165.8, 141.0, 137.0, 127.3, 100.0, 99.7, 82.0, 73.8, 71.2, 69.3, 61.0, 14.4. **HRMS (ESI) m/z:** $[M + Na]^+$ calcd for $C_{12}H_{15}INaO_5$ 388.9856, found 388.9856. **IR (KBr) ν:** 2979, 1709, 1329, 1269, 1176, 1101, 1024, 982, 849, 505 cm^{-1}.

2. Tandem carbopalladation/Suzuki cross-coupling reaction

3.1. Optimisation with phenyl boronic acid

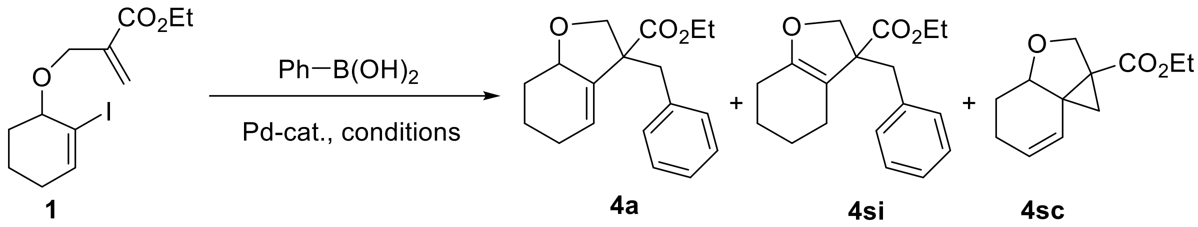

Table S1: Additional results of optimisation with phenyl boronic acid

Entry	Catalyst	Base	Ligand	Solvent	T (° C)	Time	Yield (%)[a]
1	$Pd(OAc)_2$	Cs_2CO_3	P(*o*-tol)$_3$	EtOH	80 (MW)	12 min	63[b]
2	$Pd(OAc)_2$	Cs_2CO_3	P(*o*-tol)$_3$	EtOH	80 (MW)	5 min	65[b]
3	$Pd(TFA)_2$	Cs_2CO_3	P(*o*-tol)$_3$	EtOH	80 (MW)	5 min	57
4	$Pd_2(dba)_3$	K_2CO_3	---	EtOH	80 (MW)	5 min	36[b, c]
5	$Pd_2(dba)_3$	Et_3N	---	EtOH	80 (MW)	10 min	0
6	$Pd_2(dba)_3$	K_3PO_4	---	EtOH	80 (MW)	5 min	46
7	Pd/C	Cs_2CO_3	---	EtOH	80 (MW)	5 min	0
8	$Pd_2(dba)_3$	Cs_2CO_3	---	EtOH	r.t. to 85	48 h	50[b]
9	$Pd_2(dba)_3$	Cs_2CO_3	Xphos	EtOH	85	12 min	traces
10	$Pd(OAc)_2$	*t*BuOK	PPh_3	Toluene	80	overnight	0

*5 mol% Pd was used in all cases (2.5 mol% for $Pd_2(dba)_3$); 10 mol% ligand. [a] ^{1}H NMR yields using 3,4,5-trichloropyridine as an internal standard. [b] Side products **4si** and **4sc** were formed in <15% combined yield. [c] Ratio **4a:4si** = 2.4:1.*

- General procedure A with MW irradiation

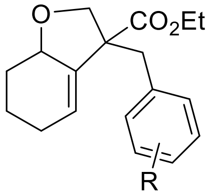

The starting material **1** (1.0 mmol, 0.10 M) was placed in a microwave reaction vial and dissolved in EtOH (10.2 mL) under an argon atmosphere. Boronic acid (1.5 mmol, 1.5 eq.) and cesium carbonate (2 mmol, 2 eq.) were added, followed by $Pd_2(dba)_3$ (0.025 mmol, 2.5 mol%). The mixture was degassed by three evacuating/refilling cycles, then it was allowed to stir at 80 °C under microwave irradiation for 12 min. The reaction mixture was filtered through a pad of Celite, and the solvent was removed using a rotary evaporator. The crude product was purified using column chromatography on silica gel (95/5 hexanes/ethyl acetate).

- General procedure B without MW irradiation

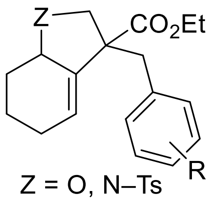

The starting material **1** or **2** (1 mmol, 0.10 M) was dissolved and stirred in anhydrous EtOH (10.2 mL) under an argon atmosphere. Boronic acid (1.5 mmol, 1.5 eq.) and cesium carbonate (2 mmol, 2 eq.) were added, followed by $Pd_2(dba)_3$ (0.025 mmol, 2.5 mol%). The mixture was degassed by three evacuating/refilling cycles, then it was allowed to stir at 80 °C in an oil bath, and the progress of the reaction was monitored by TLC. The reaction mixture was filtered through a pad of Celite, and the solvent was removed using a rotary evaporator. The crude product was purified by column chromatography on silica gel (95/5 hexanes/ethyl acetate).

Ethyl 3-(benzo[*d*][1,3]dioxol-5-ylmethyl)-2,3,5,6,7,7a-hexahydrobenzofuran-3-carboxylate (4a).

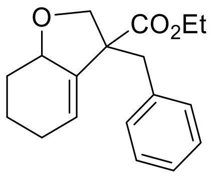

The title compound was prepared from compound **1** (200 mg, 0.59 mmol) according to General procedure B and purified by column chromatography on silica gel (95/5 hexanes/ethyl acetate) to afford the product as a colourless oil (228 mg, 96%).

^{1}H NMR (400 MHz, $CDCl_3$) δ 7.28 – 7.19 (m, 3H), 7.17 – 7.11 (m, 2H), 5.92 – 5.81 (m, 1H), 4.38 (d, *J* = 9.3 Hz, 1H), 4.17 (dq, *J* = 7.1, 2.8, 2H), 3.98 – 3.93 (m, 1H), 3.73 (d, *J* = 9.3 Hz, 1H), 3.35 (d, *J* = 13.8 Hz, 1H), 2.95 (d, *J* = 13.8 Hz, 1H), 2.21 – 1.98 (m, 3H), 1.98 – 1.74 (m, 1H), 1.54 – 1.40 (m, 1H), 1.33 – 1.25 (m, 1H), 1.23 (t, *J* = 7.1 Hz, 3H). **^{13}C NMR** (101 MHz, $CDCl_3$) δ 173.1, 142.6, 137.5, 129.8, 128.4, 126.8, 120.7, 77.5, 73.3, 61.2, 57.1, 42.8, 28.3, 25.0, 19.5, 14.2. **HRMS (ESI) m/z:** $[M + Na]^+$ calcd for $C_{18}H_{22}NaO_3$ 309.1461, found 309.1460. **IR (KBr) ν:** 2943, 1763, 1724, 1448, 1275, 1217, 1192, 1099, 1066, 999, 700 cm^{-1}.

Ethyl 3-(benzo[*d*][1,3]dioxol-5-ylmethyl)-2,3,5,6,7,7a-hexahydrobenzofuran-3-carboxylate (4b).

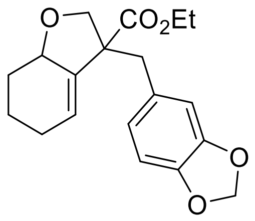

The title compound was prepared from compound **1** (38 mg, 0.11 mmol) according to General procedure B at room temperature and purified by column chromatography on silica gel (95/5 hexanes/ethyl acetate) to afford the product as a colourless oil (30 mg, 80%). The reaction was also repeated on a larger scale using compound **1** (350 mg, 1.03 mmol) following General Procedure B at room temperature, affording the product (245 mg, 72%).

^{1}**H NMR** (300 MHz, $CDCl_3$) δ 6.69 (d, *J* = 7.9 Hz, 1H), 6.64 (d, *J* = 1.7 Hz, 1H), 6.59 (dd, *J* = 7.9, 1.8 Hz, 1H), 5.91 (s, 2H), 5.84 – 5.80 (m, 1H), 4.36 (d, *J* = 9.3 Hz, 1H), 4.21 – 4.13 (m, 2H), 3.99 – 3.93 (m, 1H), 3.70 (d, *J* = 9.3 Hz, 1H), 3.26 (d, *J* = 13.9 Hz, 1H), 2.85 (d, *J* = 13.9 Hz, 1H), 2.12 (m, 3H), 1.84 (m, 1H), 1.47 (tddd, *J* = 13.3, 9.8, 6.9, 2.7 Hz, 1H), 1.34 – 1.26 (m, 1H), 1.24 (t, *J* = 7.1 Hz, 3H). ^{13}**C NMR** (101 MHz, $CDCl_3$) δ 173.1, 147.6, 146.4, 142.5, 131.1, 122.9, 120.7, 110.0, 108.1, 101.0, 77.5, 73.2, 61.2, 57.2, 42.5, 28.3, 25.0, 19.5, 14.2. **HRMS (ESI) m/z:** $[M + Na]^+$ calcd for $C_{19}H_{22}NaO_5$ 353.1359, found 353.1366. **IR (KBr) ν:** 2933, 1722, 1502, 1489, 1442, 1244, 1038, 926, 864, 812 cm^{-1}.

Ethyl 3-(4-methoxybenzyl)-2,3,5,6,7,7a-hexahydrobenzofuran-3-carboxylate (4c).

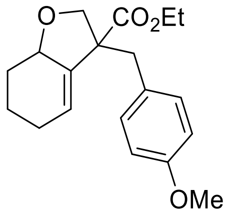

The title compound was prepared from compound **1** (100 mg, 0.3 mmol) according to General procedure B, and purified by column chromatography on silica gel (95/5 hexanes/ethyl acetate) to afford the product as a colourless oil (81 mg**,** 85%).

^{1}**H NMR (400** MHz, $CDCl_3$) δ 7.08 – 7.03 (m, 2H), 6.81 – 6.76 (m, 2H), 5.84 – 5.8 (m, 1H), 4.36 (d, *J* = 9.3 Hz, 1H), 4.17 (qd, *J* = 7.1, 2.3 Hz, 2H), 3.98 – 3.91 (m, 1H), 3.77 (s, 3H), 3.72 (d, *J* = 9.3 Hz, 1H), 3.27 (d, *J* = 13.9 Hz, 1H), 2.89 (d, *J* = 13.9 Hz, 1H), 2.20 – 2.10 (m, 2H), 2.10 – 1.98 (m, 1H), 1.89 – 1.79 (m, 1H), 1.37 (m, 1H), 1.34 – 1.22 (m, 1H), 1.14 (t, *J* = 7.1 Hz, 3H). ^{13}**C NMR** (101 MHz, $CDCl_3$) δ 173.2, 158.5, 142.6, 130.8 (2C), 129.5, 120.6, 113.8 (2C), 77.6, 73.3, 61.1, 57.3, 55.3, 42.0, 28.3, 25.1, 19.6, 14.3. **HRMS (ESI) m/z:** $[M + Na]^+$ calcd for $C_{19}H_{24}NaO_4$ 339.1567, found 339.1565. **IR (KBr) ν:** 2933, 1724, 1610, 1512, 1464, 1277, 1248, 1178, 1113, 1070, 1036, 837 cm^{-1}.

Ethyl 3-(4-(trifluoromethyl)benzyl)-2,3,5,6,7,7a-hexahydrobenzofuran-3-carboxylate (4d).

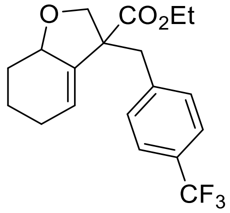

The title compound was prepared from compound **1** (200 mg, 0.59 mmol) according to General procedure A and purified by column chromatography on silica gel (95/5 hexanes/ethyl acetate) to afford the product as a colourless oil (100 mg, 30%).

^{1}**H NMR** (400 MHz, $CDCl_3$) δ 7.51 (d, *J* = 8.0 Hz, 2H), 7.26 (d, *J* = 8.1 Hz, 2H), 5.84 – 5.80 (m, 1H), 4.37 (d, *J* = 9.3 Hz, 1H), 4.17 (q, *J* = 7.1 Hz, 2H), 3.99 – 3.93 (m, 1H), 3.69 (d, *J* = 9.3 Hz, 1H), 3.39 (d, *J* = 13.8 Hz, 1H), 3.01 (d, *J* = 13.9 Hz, 1H), 2.22 – 1.97 (m, 3H), 1.89 – 1.80 (m, 1H), 1.53 – 1.41 (m, 1H), 1.35 – 1.25 (m, 1H), 1.22 (td, *J* = 7.2, 0.9 Hz, 3H). ^{13}**C**

NMR (101 MHz, $CDCl_3$) δ 172.8, 142.2, 141.7, 129.2 (q, *J* = 32.4 Hz), 130.1 (2C), 124.3 (q, *J* = 272.1 Hz), 125.3 (q, *J* = 3.8 Hz, 2C), 121.2, 77.5, 73.3, 61.4, 56.9, 42.4, 28.3, 25.0, 19.5, 14.2. **HRMS (ESI) m/z:** $[M + Na]^+$ calcd for $C_{19}H_{21}F_3NaO_3$ 377.1335, found 377.1333. **IR (KBr) ν:** 2941, 1726, 1618, 1446, 1323, 1161, 1113, 1066, 1018, 937 cm^{-1}.

Ethyl 3-(4-methylbenzyl)-2,3,5,6,7,7a-hexahydrobenzofuran-3-carboxylate (4e).

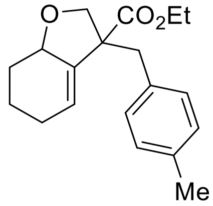

The title compound was prepared from compound **1** (100 mg, 0.3 mmol) according to General procedure B and purified by column chromatography on silica gel (95/5 hexanes/ethyl acetate) to afford the product as a colourless oil (75 mg, 85%).

H NMR (400 MHz, $CDCl_3$) δ 7.08 – 7.00 (m, 4H), 5.85 – 5.80 (m, 1H), 4.37 (d, *J* = 9.3 Hz, 1H), 4.17 (dq, *J* = 7.1, 2.8 Hz, 2H), 3.99 – 3.93 (m, 1H), 3.72 (d, *J* = 9.4 Hz, 1H), 3.31 (d, *J* = 13.8 Hz, 1H), 2.90 (d, *J* = 13.8 Hz, 1H), 2.30 (s, 3H), 2.21 – 1.99 (m, 3H), 1.91 – 1.79 (m, 1H), 1.53 – 1.40 (m, 1H), 1.36 – 1.27 (m, 1H), 1.24 (t, *J* = 7.1 Hz, 3H). **^{13}C NMR** (101 MHz, $CDCl_3$) δ 173.2, 142.7, 136.4, 134.4, 129.6 (2C), 129.1 (2C), 120.6, 77.6, 73.3, 61.1, 57.2, 42.4, 28.3, 25.1, 21.1, 19.6 14.3. **HRMS (ESI) m/z:** $[M + Na]^+$ calcd for $C_{19}H_{24}NaO_3$ 323.1617, found 323.1614. **IR (KBr) ν:** 2933, 1724, 1514, 1448, 1367, 1273, 1186, 1093, 1070, 1020, 698 cm^{-1}.

Ethyl 3-(benzofuran-2-ylmethyl)-2,3,5,6,7,7a-hexahydrobenzofuran-3-carboxylate (4f).

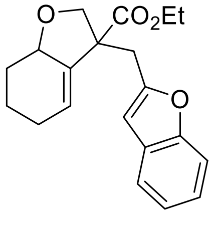

The title compound was prepared from compound **1** (200 mg, 0.59 mmol) according to General procedure A and purified by column chromatography on silica gel (95/5 to 80/20 hexanes/ethyl acetate) to afford the product as a colourless oil (107 mg, 55%).

^{1}H NMR (400 MHz, $CDCl_3$) δ 7.51 – 7.44 (m, 1H), 7.38 (m, 1H), 7.25 – 7.15 (m, 2H), 6.41 – 6.40 (m, 1H), 5.86 – 5.84 (m, 1H), 4.56 (d, *J* = 9.5 Hz, 1H), 4.21 (q, *J* = 7.1 Hz, 2H), 4.09 – 4.02 (m, 1H), 3.82 (d, *J* = 9.5 Hz, 1H), 3.55 (dd, *J* = 15.2, 0.9 Hz, 1H), 3.07 (dd, *J* = 15.3, 0.8 Hz, 1H), 2.26 – 1.99 (m, 3H), 1.92 – 1.80 (m, 1H), 1.55 – 1.43 (m, 1H), 1.39 – 1.27 (m, 1H), 1.25 (t, *J* = 7.1 Hz, 3H). **^{13}C NMR** (101 MHz, $CDCl_3$) δ 172.6, 155.2, 154.7, 142.4, 128.6, 123.8, 122.7, 120.9, 120.6, 111.0, 104.4, 77.3, 74.0, 61.4, 55.6, 36.1, 28.4, 25.0, 19.5, 14.2. **HRMS (ESI) m/z:** $[M + Na]^+$ calcd for $C_{20}H_{22}NaO_4$ 349.1410, found 349.1410. **IR (KBr) ν:** 2937, 1728, 1454, 1294, 1252, 1196, 1171, 1101, 1065, 1001, 947, 750, 741 cm^{-1}.

Ethyl 3-(2-chlorobenzyl)-2,3,5,6,7,7a-hexahydrobenzofuran-3-carboxylate (4g).

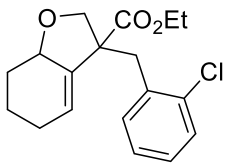

The title compound was prepared from compound **1** (200 mg, 0.59 mmol) according to General procedure A and purified by column chromatography on silica gel (95/5 hexanes/ethyl acetate) to afford the product as a colourless oil (65 mg, 30%).

Some traces of dba[4] remained in the product. **^{1}H NMR** (400 MHz, $CDCl_3$) δ 7.21 – 7.11 (m, 3H), 5.82 – 5.78 (m, 1H), 4.48 (d, *J* = 9.4 Hz, 1H), 4.19 (dq, *J* = 7.1, 1.1 Hz, 2H), 4.01 – 3.89 (m, 1H), 3.68 (d, *J* = 9.4 Hz, 1H), 3.46 (d, *J* = 14.8 Hz, 1H), 3.24 (d, *J* = 14.8 Hz, 1H), 2.23 – 2.10 (m, 2H), 2.10 – 1.98 (m, 1H), 1.92 – 1.78 (m, 1H), 1.56 – 1.36 (m, 2H), 1.31 – 1.26 (m, 1H), 1.23 (t, *J* = 7.1 Hz, 3H). **^{13}C NMR** (101 MHz, $CDCl_3$) δ 173.3, 142.4, 135.6, 135.0, 130.6, 129.7, 128.1, 126.7, 121.1, 77.4, 73.2, 61.3, 56.5, 38.8, 28.3, 25.1, 19.5, 14.2. **HRMS (ESI) m/z:** $[M + Na]^+$ calcd for $C_{18}H_{21}ClNaO_3$ 343.1071, found 343.1069. **IR (KBr) ν:** 2939, 1724, 1442, 1273, 1209, 1192, 1095, 1070, 1039, 752 cm^{-1}.

Ethyl 3-(2-methoxybenzyl)-2,3,5,6,7,7*a*-hexahydrobenzofuran-3-carboxylate (4k).

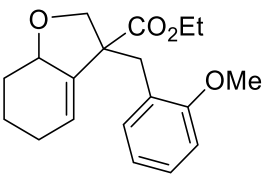

The title compound was prepared from compound **1** (340 mg, 1.01 mmol) according to General procedure B and purified by column chromatography on silica gel (95/5 to 90/10 hexanes/ethyl acetate) to afford the product as a colourless oil (190 mg, 60%).

^{1}H NMR (400 MHz, $CDCl_3$) δ 7.18 (td, *J* = 7.8, 1.8 Hz, 1H), 7.09 (dd, *J* = 7.5, 1.7 Hz, 1H), 6.87 – 6.79 (m, 2H), 5.87 – 5.79 (m, 1H), 4.43 (d, *J* = 9.3 Hz, 1H), 4.23 – 4.10 (m, 2H), 3.95 – 3.88 (m, 1H), 3.79 (s, 3H), 3.69 (d, *J* = 9.4 Hz, 1H), 3.31 (d, *J* = 14.1 Hz, 1H), 3.11 (d, *J* = 14.2 Hz, 1H), 2.20 – 2.00 (m, 3H), 1.88 – 1.76 (m, 1H), 1.52 – 1.38 (m, 1H), 1.33 – 1.25 (m, 1H), 1.23 (t, *J* = 7.1 Hz, 3H). **^{13}C NMR** (101 MHz, $CDCl_3$) δ 173.6, 157.8, 143.1, 131.0, 128.0, 126.1, 120.41, 120.37, 110.4, 77.5, 73.3, 61.1, 56.8, 55.2, 36.0, 28.4, 25.1, 19.6, 14.3. **HRMS (ESI) m/z:** $[M + Na]^+$ calcd for $C_{19}H_{24}NaO_4$ 339.1567, found 339.1570. **IR (KBr) ν:** 2975, 1724, 1494, 1440, 1244, 1221, 1205, 1119, 1026, 999, 754 cm^{-1}.

Ethyl 3-(pyridin-4-ylmethyl)-2,3,5,6,7,7*a*-hexahydrobenzofuran-3-carboxylate (4l).

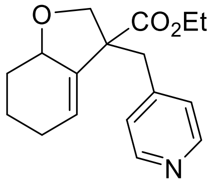

The title compound was prepared from compound **1** (60 mg, 0.18 mmol) according to General procedure B and purified by column chromatography on silica gel (80/20 to 50/50 hexanes/ethyl acetate) to afford the product as a colourless oil (10 mg, 17 %).

^{1}H NMR (400 MHz, $CDCl_3$) δ 8.52 (s, 2H), 7.15 (s, 2H), 5.80 – 5.75 (m, 1H), 4.39 (d, *J* = 9.3 Hz, 1H), 4.18 (q, *J* = 7.1 Hz, 2H), 4.03 – 3.94 (m, 1H), 3.67 (d, *J* = 9.3 Hz, 1H), 3.35 (d, *J* = 13.9 Hz, 1H), 2.98 (d, *J* = 13.9 Hz, 1H), 2.22 – 2.00 (m, 3H), 1.90 – 1.83 (m, 1H), 1.54 – 1.41 (m, 1H), 1.35 – 1.21 (m, 1H), 1.22 (t, *J* = 7.1 Hz, 3H). **^{13}C NMR** (101 MHz, $CDCl_3$) δ 172.6, 148.6 (2C), 148.1, 141.9, 125.5 (2C), 121.7, 77.5, 73.5, 61.5, 56.4, 42.0, 28.3, 25.0, 19.4, 14.2. **HRMS (ESI) m/z:** $[M + H]^+$ calcd for $C_{17}H_{22}NO_3$ 288.1600, found 288.1594. **IR (KBr) ν:** 2933, 1724, 1230, 1213, 1184, 1097, 1070, 1018, 1003, 731 cm^{-1}.

2-(Pyridin-4-yl)cyclohex-2-en-1-ol (4sl) (side product).

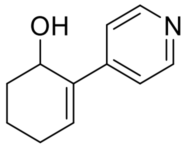

The title compound was isolated with **4l** as a side product.

^{1}H NMR (400 MHz, $CDCl_3$) δ 8.54 – 8.44 (m, 2H), 7.42 – 7.36 (m, 2H), 6.45 – 6.40 (m, 1H), 4.70 – 4.64 (m, 1H), 2.56 (s, 1H), 2.29 (t, *J* = 4.4 Hz, 1H), 2.26 – 2.13 (m, 1H), 2.02 – 1.91 (m, 1H), 1.91 – 1.77 (m, 2H), 1.74 – 1.63 (m, 1H). **^{13}C NMR** (101 MHz, $CDCl_3$) δ 149.7 (2C), 148.1, 137.0, 132.4, 120.7 (2C), 64.7, 31.9, 26.3, 17.1.

Ethyl 3-(thiophen-3-ylmethyl)-2,3,5,6,7,7a-hexahydrobenzofuran-3-carboxylate (4m).

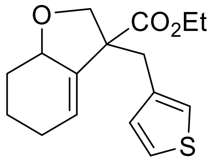

The title compound was prepared from compound **1** (50 mg, 0.15 mmol) according to General procedure B and purified by column chromatography on silica gel (95/5 to 90/10 hexanes/ethyl acetate) to afford the product as a colourless oil (36 mg, 81%).

^{1}H NMR (400 MHz, $CDCl_3$) δ 7.21 (dd, *J* = 5, 3.0 Hz, 1H), 6.96 (dd, *J* = 3.0, 1.3 Hz, 1H), 6.88 (dd, *J* = 5.0, 1.3 Hz, 1H), 5.82 – 5.76 (m, 1H), 4.41 (d, *J* = 9.3 Hz, 1H), 4.21 – 4.14 (m, 2H), 4.01 – 3.93 (m, 1H), 3.69 (d, *J* = 9.3 Hz, 1H), 3.35 (d, *J* = 14.2 Hz, 1H), 2.96 (d, *J* = 14.2 Hz, 1H), 2.22 – 1.98 (m, 3H), 1.89 – 1.80 (m, 1H), 1.55 – 1.41 (m, 1H), 1.34 – 1.26 (m, 1H), 1.23 (t, *J* = 7.1 Hz, 3H). **^{13}C NMR** (101 MHz, $CDCl_3$) δ 173.2, 142.7, 137.8, 129.0, 125.4, 122.8, 120.5, 77.5, 73.7, 61.2, 56.9, 37.4, 28.3, 25.0, 19.6, 14.3. **HRMS (ESI) m/z:** $[M + Na]^+$ calcd

for $C_{16}H_{20}NaO_3S$ 315.1031, found 315.1018. **IR (KBr) ν:** 2933, 1724, 1233, 1214, 1156, 1107, 1075, 1006, 866, 783 cm^{-1}.

Ethyl 3-(3,5-dimethylbenzyl)-2,3,5,6,7,7a-hexahydrobenzofuran-3-carboxylate (4n).

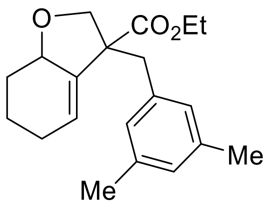

The title compound was prepared from compound **1** (50 mg, 0.15 mmol) according to General procedure B and purified by column chromatography on silica gel (95/5 to 90/10 hexanes/ethyl acetate) to afford the product as a colourless oil (46 mg, 98%).

^{1}H NMR (400 MHz, $CDCl_3$) δ 6.84 (s, 1H), 6.78 – 6.72 (m, 2H), 5.87 – 5.81 (m, 1H), 4.36 (d, *J* = 9.3 Hz, 1H), 4.23 – 4.13 (m, 2H), 4.01 – 3.93 (m, 1H), 3.72 (d, *J* = 9.3 Hz, 1H), 3.30 (d, *J* = 13.8 Hz, 1H), 2.85 (d, *J* = 13.8 Hz, 1H), 2.26 (s, 6H), 2.20 – 1.99 (m, 3H), 1.90 – 1.81 (m, 1H), 1.55 – 1.40 (m, 1H), 1.34 – 1.28 (m, 1H), 1.27 (t, 3H). **^{13}C NMR** (101 MHz, $CDCl_3$) δ 173.2, 142.8, 137.8 (2C), 137.3, 128.4, 127.6 (2C), 120.5, 77.5, 73.2, 61.1, 57.1, 42.8, 28.4, 25.1, 21.4 (2C), 19.6, 14.3. **HRMS (ESI) m/z:** $[M + Na]^+$ calcd for $C_{18}H_{22}NaO_4$ 337.1780, found 337.1774. **IR (KBr) ν:** 2929, 1732, 1601, 1504, 1390, 1234, 1207, 1155, 1115, 1101, 1062 cm^{-1}.

Ethyl 3-(4-acetylbenzyl)-2,3,5,6,7,7a-hexahydrobenzofuran-3-carboxylate (4p).

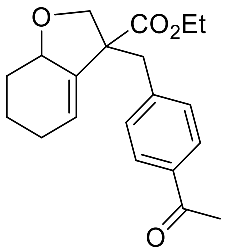

The title compound was prepared from compound **1** (200 mg, 1.01 mmol) according to General procedure B and purified by column chromatography on silica gel (95/5 to 90/10 hexanes/ethyl acetate) to afford the product as an amorphous oil (137 mg, 71 %).

^{1}H NMR (400 MHz, $CDCl_3$) δ 7.84 (d, *J* = 8.3 Hz, 1H), 7.23 (d, *J* = 8.3 Hz, 1H), 5.83 – 5.79 (m, 1H), 4.35 (d, *J* = 9.3 Hz, 1H), 4.19 – 4.13 (m, 2H), 3.98 – 3.91 (m, 1H), 3.69 (d, *J* = 9.3 Hz, 1H), 3.38 (d, *J* = 13.8 Hz, 1H), 3.01 (d, *J* = 13.8 Hz, 1H), 2.56 (s, 3H), 2.19 – 1.98 (m, 3H), 1.89 – 1.79 (m, 1H), 1.52 – 1.39 (m, 1H), 1.32 – 1.25 (m, 1H), 1.22 (t, *J* = 7.1 Hz, 3H). **^{13}C NMR** (101 MHz, $CDCl_3$) δ 197.9, 172.8, 143.2 (2C), 142.2 (2C), 135.8, 130.0, 128.4, 121.2, 77.5, 73.3, 61.3, 56.9, 42.6, 28.3, 26.7, 25.0, 19.5, 14.2. **HRMS (ESI) m/z:** $[M + H]^+$ calcd for $C_{20}H_{25}O_4$ 329.1747, found 329.1741. **IR (KBr) ν:** 2935, 1724, 1682, 1606, 1358, 1271, 1271, 1184, 1072, 602 cm^{-1}.

Ethyl 3-benzyl-1-tosyl-2,3,5,6,7,7a-hexahydro-1*H*-indole-3-carboxylate (5a).

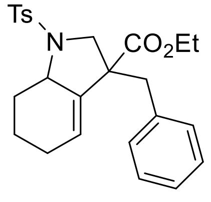

The title compound was prepared from compound **2** (30.5 mg, 0.06 mmol) according to General procedure B and purified by column chromatography on silica gel (95/5 to 80/20 hexanes/ethyl acetate) to afford the product as a colourless oil (27 mg, 98%).

^{1}H NMR (400 MHz, $CDCl_3$) δ 7.73 – 7.64 (m, 2H), 7.32 – 7.28 (m, 2H), 7.20 – 7.13 (m, 3H), 6.98 – 6.92 (m, 2H), 5.87 – 5.81 (m, 1H), 4.21 – 4.07 (m, 2H), 3.78 (d, *J* = 10.5 Hz, 1H), 3.64 – 3.57 (m, 1H), 3.29 (d, *J* = 10.5 Hz, 1H), 3.03 (d, *J* = 13.8 Hz, 1H), 2.69 (d, *J* = 13.8 Hz, 1H), 2.55 – 2.47 (m, 1H), 2.44 (s, 3H), 2.16 – 1.95 (m, 2H), 1.89 – 1.80 (m, 1H), 1.52 – 1.38 (m, 1H), 1.37 – 1.26 (m, 1H), 1.21 (t, *J* = 7.1 Hz, 3H). **^{13}C NMR** (101 MHz, $CDCl_3$) δ 171.9, 143.6, 139.3, 136.7, 134.9, 130.0 (2 C), 129.8 (2 C), 128.3 (2 C), 127.8 (2 C), 126.9, 122.9, 61.4, 59.2, 55.5, 53.4, 42.3, 29.2, 24.5, 21.7, 19.9, 14.2. **HRMS (ESI) m/z:** $[M + Na]^+$ calcd for $C_{25}H_{29}NNaO_4S$ 462.1715, found 462.1707. **IR (KBr) ν:** 2931, 1726, 1342, 1452, 1165, 1092, 1045, 702, 665, 552 cm^{-1}.

Ethyl 3-(4-methoxybenzyl)-1-tosyl-2,3,5,6,7,7a-hexahydro-1*H*-indole-3-carboxylate (5c).

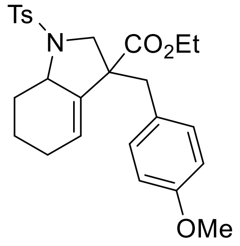

The title compound was prepared from compound **2** (181 mg, 0.37 mmol) according to General procedure B and purified by column chromatography on silica gel (95/5 to 80/20 hexanes/ethyl acetate) to afford the product as a colourless oil (123 mg, 72%), together with an impurity 2-(4-methoxy)-cyclohex-2-en-1-ol.[5]

^{1}H NMR (400 MHz, $CDCl_3$) δ 7.73 – 7.66 (m, 2H), 7.33 – 7.27 (m, 2H), 6.87 – 6.85 (m, 2H), 6.75 – 6.67 (m, 2H), 5.86 – 5.80 (m, 1H), 4.14 (q, *J* = 7.0 Hz, 2H), 3.76 (d, *J* = 10.3 Hz, 1H), 3.76 (s, 3H), 3.64 – 3.56 (m, 1H), 3.29 (d, *J* = 10.5 Hz, 1H), 2.96 (d, *J* = 13.8 Hz, 1H), 2.65 (d, *J* = 13.9 Hz, 1H), 2.55 – 2.46 (m, 1H), 2.43 (s, 3H), 2.19 – 1.91 (m, 1H), 1.89 – 1.82 (m, 1H), 1.53 – 1.37 (m, 1H), 1.36 – 1.25 (m, 2H), 1.22 (t, *J* = 7.1 Hz, 3H). **^{13}C NMR** (101 MHz, $CDCl_3$) δ 171.9, 158.5, 143.6, 139.3, 131.0 (2C), 129.7 (2C), 128.6, 127.7 (2C), 127.1, 122.1, 113.6 (2C), 61.3, 59.1, 55.7, 55.2, 53.2, 41.4, 29.1, 24.4, 21.6, 19.9, 14.2. **HRMS (ESI) m/z:** $[M + H]^+$ calcd for $C_{26}H_{32}NO_5S$ 470.1996, found 470.2010.

Ethyl 3-(4-methylbenzyl)-1-tosyl-2,3,5,6,7,7a-hexahydro-1*H*-indole-3-carboxylate (5e).

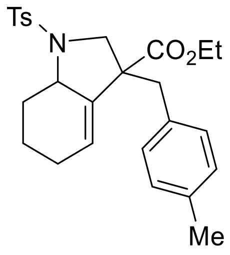

The title compound was prepared from compound **2** (50 mg, 0.1 mmol) according to General procedure B and purified by column chromatography on silica gel (95/5 to 90/10 hexanes/ethyl acetate) to afford the product as a colourless oil (38 mg, 82 %).

^{1}H NMR (400 MHz, $CDCl_3$) δ 7.69 (d, *J* = 8.0 Hz, 2H), 7.31 (d, *J* = 8.0 Hz, 2H), 6.97 (d, *J* = 8.1 Hz, 2H), 6.83 (d, *J* = 8.1 Hz, 2H), 5.86 – 5.82 (m, 1H), 4.23 – 4.08 (m, 2H), 3.78 (d, *J* = 10.6 Hz, 1H), 3.64 – 3.48 (m, 1H), 3.27 (d, *J* = 10.6 Hz, 1H), 2.99 (d, *J* = 13.7 Hz, 1H), 2.64 (d, *J* = 13.7 Hz, 1H), 2.54 – 2.46 (m, 1H), 2.44 (s, 3H), 2.28 (s, 3H), 2.15 – 1.94 (m, 3H), 1.87 – 1.78 (m, 1H), 1.51 – 1.38 (m, 1H), 1.36 – 1.26 (m, 2H), 1.22 (t, *J* = 7.1 Hz, 3H). **^{13}C NMR** (101 MHz, $CDCl_3$) δ 172.0, 143.6, 139.3, 136.4, 134.9, 133.5, 129.9 (2C), 129.7 (2C), 129.0 (2C), 127.8 (2C), 122.8, 61.3, 59.2, 55.6, 53.3, 42.0, 29.2, 24.5, 21.7, 21.2, 20.0, 14.2. **HRMS (ESI) m/z:** $[M + Na]^+$ calcd for $C_{26}H_{31}NNaO_4S$ 476.1871, found 476.1867. **IR (KBr) ν:** 2927, 1728, 1337, 1155, 1063, 822, 663, 625, 569, 540 cm^{-1}

Ethyl 3a,4-dihydro-1*H*,5*H*-cyclopropa[*c*]benzofuran-1a(2*H*)-carboxylate (4sc).

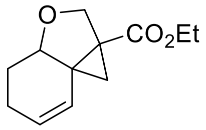

The title compound was purified by column chromatography on silica gel (95/5 hexanes/ethyl acetate) to afford the product as a colourless oil.

^{1}H NMR (400 MHz, $CDCl_3$) δ 5.86 – 5.78 (m, 1H), 5.56 – 5.52 (m, 1H), 4.33 (dd, *J* = 8.6, 1.1 Hz, 1H), 4.21 – 4.11 (m, 2H), 4.00 (dd, *J* = 12.0, 4.7 Hz, 1H), 3.90 (d, *J* = 8.8 Hz, 1H), 2.26 – 2.16 (m, 2H), 1.90 – 1.84 (m, 1H), 1.71 (dt, *J* = 4.6, 0.9 Hz, 1H), 1.67 – 1.54 (m, 1H), 1.41 (d, *J* = 4.5 Hz, 1H), 1.25 (t, *J* = 7.1 Hz, 3H). **^{13}C NMR** (101 MHz, $CDCl_3$) δ 170.5, 129.9, 124.7, 78.4, 67.6, 60.8, 41.5, 37.7, 27.1, 24.2, 22.2, 14.4. **HRMS (ESI) m/z:** $[M + Na]^+$ calcd for $C_{12}H_{16}NaO_3$ 231.0992, found 231.0996. **IR (KBr) ν:** 2935, 1726, 1450, 1377, 1273, 1228, 1134, 1068, 1049, 1028, 968, 862 cm^{-1}.

3.2. Optimisation of the tandem reaction with levoglucosenone-based compound 3 and phenylboronic acid.

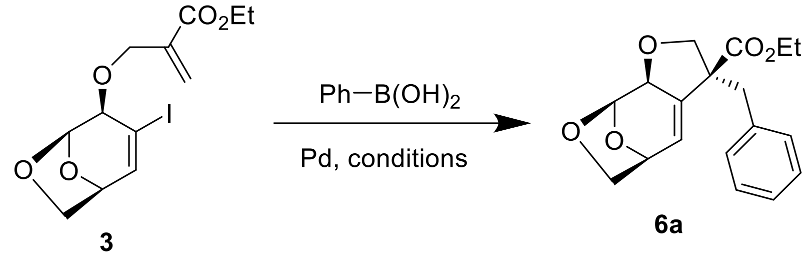

Table S2: Optimisation of the reaction of compound **3** with phenylboronic acid.

Entry	Catalyst	Base	Ligand	Solvent	Temp. (° C)	Time	Yield (%)
1	$Pd_2(dba)_3$	Cs_2CO_3	---	anhydrous EtOH	80	4-5 h	traces
2	$Pd_2(dba)_3$	Cs_2CO_3	---	anhydrous EtOH	r.t.	16 h	traces
3	$Pd(OAc)_2$	Cs_2CO_3	P(*o*-tol)$_3$	anhydrous EtOH	80	24 h	traces
4	$Pd_2(dba)_3$	Cs_2CO_3	---	dioxane	r.t.	16 h	0
5	$Pd(PPh_3)_4$	Cs_2CO_3	---	toluene/H_2O	60	5 h	44[a, b]
6	$Pd_2(dba)_3$	Cs_2CO_3	---	anhydrous EtOH	60	overnight	traces

5 mol% Pd was used in all cases (2.5 mol% for $Pd_2(dba)_3$); 10 mol% ligand. [a] ^1H NMR yield using 3,4,5-trichloropyridine as an internal standard. [b] Homocoupling of boronic acid and Heck/Heck side product formed. Conditions: $Pd(PPh_3)_4$ 5 mol%, Cs_2CO_3 (2 eq.), toluene/H_2O, 60 °C under argon atmosphere.

Ethyl (5*S*,8*R*,8a*S*)-3-benzyl-2,3,5,6,8,8a-hexahydro-5,8-epoxyfuro[2,3-*c*]oxepine-3-carboxylate (6a).

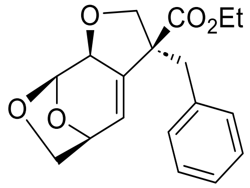

The starting material **3** (230 mg, 0.62 mmol) was dissolved and stirred in toluene/H_2O (4:1, 7 mL) under an argon atmosphere. Phenylboronic acid (0.11 g, 0.93 mmol, 1.5 eq.) and cesium carbonate (0.293 g, 1.24 mmol, 2 eq.) were added, followed by $Pd(PPh_3)_4$ (0.057 g, 0.031 mmol, 5 mol%). The mixture was degassed by three evacuation and refilling cycles, then it was allowed to stir at 60 °C in an oil bath for 4 hours. The reaction mixture was extracted between water and ethyl acetate. The organic layers were combined and dried over sodium sulfate. The solvent was removed using a rotary evaporator. The crude product was purified by column chromatography on silica gel (75/25 hexanes/ethyl acetate) to result in an orange oil (100 mg, 44%).

^{1}H NMR (400 MHz, $CDCl_3$) δ 7.36 –7.20 (m, 3H), 7.16 – 7.09 (m, 2H), 5.95 – 5.90 (m, 1H), 5.71 (d, *J* = 2.3 Hz, 1H), 4.84 – 4.79 (m, 2H), 4.45 (d, *J* = 9.3 Hz, 1H), 4.37 – 4.32 (m, 1H), 4.17 (q, *J* = 7.1 Hz, 2H), 3.90 (d, *J* = 9.4 Hz, 1H), 3.81 (dd, *J* = 6.9, 3.7 Hz, 1H), 3.18 (d, *J* = 13.7 Hz, 1H), 3.03 (d, *J* = 13.7 Hz, 1H), 1.24 (t, *J* = 7.2 Hz, 3H). **^{13}C NMR** (101 MHz, $CDCl_3$)

δ 172.1, 138.7, 136.5, 130.0 (2C), 128.5 (2C), 127.2, 121.9, 100.1, 78.5, 74.9, 74.0, 72.4, 61.4, 56.2, 42.0, 14.3. **HRMS (ESI) m/z:** $[M + Na]^+$ calcd for $C_{18}H_{20}NaO_5$ 339.1203, found 339.1202. **IR (KBr) ν:** 2958, 1722, 1446, 1186, 1130, 1082, 1066, 1022, 982, 860, 700 cm^{-1}.

3. Formation of lactones

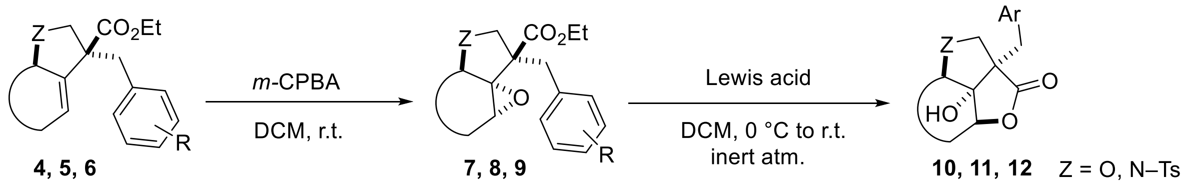

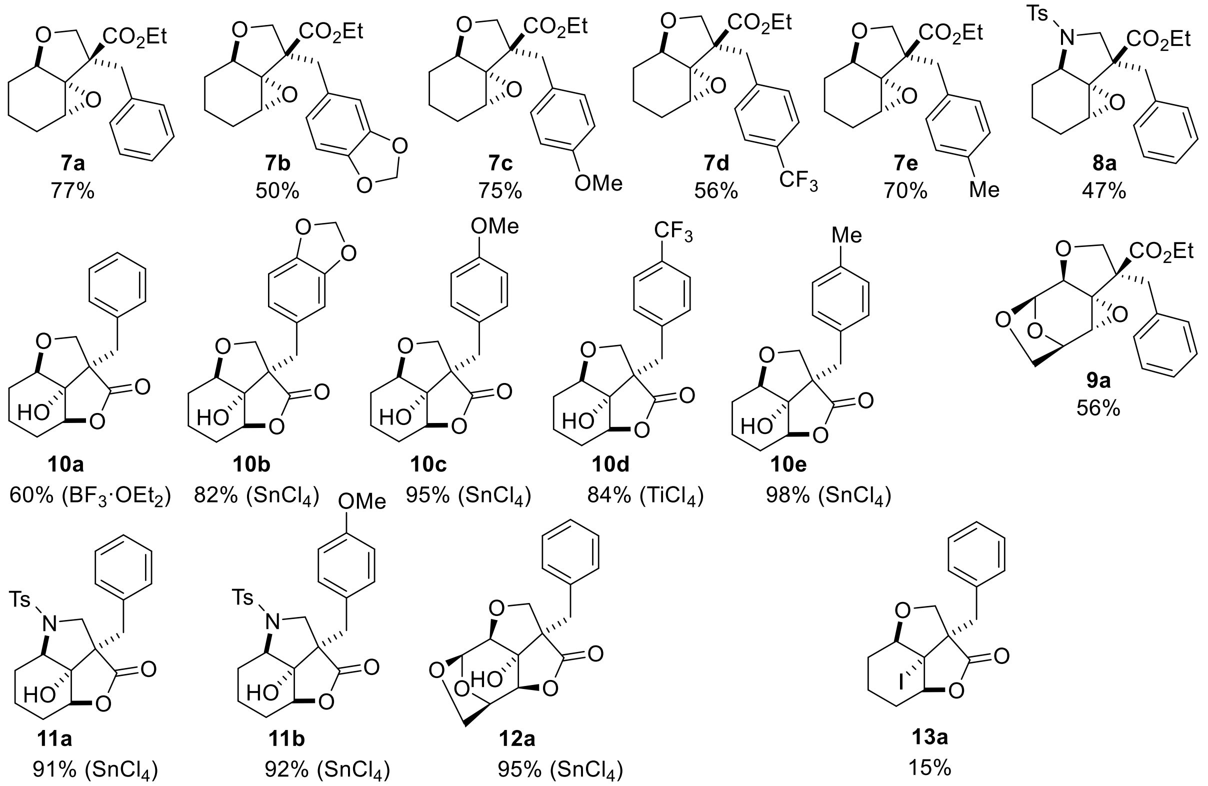

Table S3: Optimisation of lactone formation.

Entry	Substrate	Reagent	Temp. (° C)	Epoxide/ Yield (%) [a, b]	Acid	Solvent	Yield (%) [c]
1	**4a**	*m*-CPBA	r.t. to 42	77	$BF_3{\cdot}OEt_2$	DCM	60
2	**4b**	*m*-CPBA	r.t.	50	$BF_3{\cdot}OEt_2$	DCM	64
3	**4b**	H_2O_2	r.t.	0	---	---	---
4	**4b**	*m*-CPBA	r.t.	50	$SnCl_4$	DCM	82 [a]
5	**4b**	*m*-CPBA	r.t.	50	H_2SO_4	DCM	25
6	**4d**	*m*-CPBA	r.t.	56	$TiCl_4$	DCM	84
7	**6a**	*m*-CPBA	r.t.	50	$SnCl_4$	DCM	95

a) ^{1}H NMR yields using 3,4,5-trichloropyridine as an internal standard. b) The epoxide was isolated before using in the next step. c) Isolated yields.

- General procedure for epoxidation

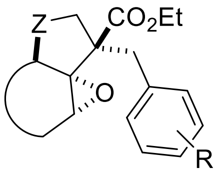

Z = O, N–Ts

The starting material (1 mmol, 40 mM) was dissolved in DCM (25 ml), and then *m*-CPBA (2 mmol, 2 eq.) was added to the solution at room temperature and continued to stir overnight, with progress and reaction time monitored by TLC. The reaction mixture was quenched with a saturated aqueous solution of Na_2SO_3 (15 mL) and extracted with DCM (3 × 25 mL). The organic layers were combined and dried over sodium sulfate. The solvent was removed using a rotary evaporator. The crude product was purified using column chromatography on silica gel (95/5 to 85/15 hexanes/ethyl acetate) to yield the corresponding epoxide, and the compound **8c** was not isolated but was used as a crude product in the next step. The progress and reaction time were monitored by TLC for each compound.

Ethyl 7-benzylhexahydro-3*H*-oxireno[2,3-*d*]benzofuran-7-carboxylate (7a).

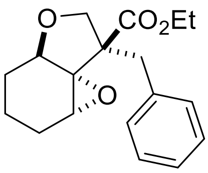

The title compound was prepared from compound **4a** (100 mg, 0.35 mmol) according to the General procedure for epoxidation and purified by column chromatography on silica gel (95/5 to 85/15 hexanes/ethyl acetate) to afford the product as a colourless oil (79 mg, 77%).

^{1}H NMR (400 MHz, $CDCl_3$) δ 7.25 – 7.13 (m, 5H), 4.38 (dd, *J* = 9.4, 0.6 Hz, 1H), 4.11 (q, *J* = 7.1 Hz, 2H), 3.79 (d, *J* = 9.4 Hz, 1H), 3.71 (dd, *J* = 10.5, 6.0 Hz, 1H), 3.49 – 3.46 (m, 1H), 3.07 (d, *J* = 14.0 Hz, 1H), 2.87 (d, *J* = 14.0 Hz, 1H), 2.16 – 2.00 (m, 2H), 1.72 – 1.60 (m, 1H), 1.59 – 1.47 (m, 2H), 1.19 (t, *J* = 7.1 Hz, 3H), 1.15 – 1.05 (m, 1H). **^{13}C NMR** (101 MHz, $CDCl_3$) δ 172.8, 136.9, 129.8 (2C), 128.4 (2C), 126.8, 77.6, 74.0, 68.3, 61.2, 57.3, 55.1, 36.6, 27.3, 25.0, 15.2, 14.3. **HRMS (ESI) m/z:** $[M + Na]^+$ calcd for $C_{18}H_{22}NaO_4$ 325.1410, found 325.1409. **IR (KBr) ν:** 2937, 1724, 1454, 1215, 1188, 1101, 995, 750, 700 cm^{-1}.

Ethyl 7-(benzo[*d*][1,3]dioxol-5-ylmethyl)hexahydro-3*H*-oxireno[2,3-*d*]benzofuran-7-carboxylate (7b).

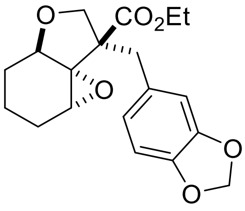

The title compound was prepared from compound **4b** (179 mg, 0.54 mmol) according to the General procedure for epoxidation and purified by column chromatography on silica gel (95/5 to 85/15 hexanes/ethyl acetate) to afford the product as a colourless oil (89 mg, 50%). The

reaction was also repeated on a larger scale using compound **4b** (350 mg, 1.03 mmol) following the General procedure for epoxidation, affording the product (178 mg, 49%).

^{1}H NMR (400 MHz, $CDCl_3$) δ 6.71 – 6.57 (m, 3H), 5.91 (s, 2H), 4.37 (d, *J* = 9.4 Hz, 1H), 4.14 (q, *J* = 7.1 Hz, 2H), 3.75 (d, *J* = 9.5 Hz, 1H), 3.69 (dd, *J* = 10.5, 6.1 Hz, 1H), 3.48 – 3.44 (m, 1H), 2.99 (d, *J* = 14.1 Hz, 1H), 2.78 (d, *J* = 14.1 Hz, 1H), 2.16 – 1.95 (m, 2H), 1.74 – 1.58 (m, 1H), 1.57 – 1.46 (m, 1H), 1.22 (t, *J* = 7.1 Hz, 3H), 1.17 – 1.04 (m, 2H). **^{13}C NMR** (101 MHz, $CDCl_3$) δ 172.8, 147.6, 146.5, 130.4, 123.0, 110.2, 108.2, 101.0, 77.6, 74.0, 68.3, 61.3, 57.3, 55.2, 36.2, 27.3, 25.0, 15.2, 14.3. **HRMS (ESI) m/z:** $[M + H]^+$ calcd for $C_{19}H_{23}O_6$ 347.1489, found 347.1487. **IR (KBr) ν:** 2954, 1712, 1444, 1360, 1246, 1219, 1101, 1038, 997, 530 cm^{-1}.

Ethyl 7-(4-methoxybenzyl)hexahydro-3*H*-oxireno[2,3-*d*]benzofuran-7-carboxylate (7c).

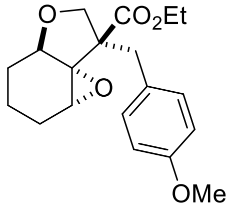

The title compound was prepared from compound **4c** (71 mg, 0.25 mmol) according to the General procedure for epoxidation and purified by column chromatography on silica gel (95/5 to 85/15 hexanes/ethyl acetate) to afford the product as a colourless oil (59 mg, 75%).

^{1}H NMR (400 MHz, $CDCl_3$) δ 7.10 – 7.06 (m, 2H), 6.79 – 6.74 (m, 2H), 4.37 (dd, *J* = 9.4, 0.6 Hz, 1H), 4.12 (q, *J* = 7.1 Hz, 2H), 3.77 (d, *J* = 9.4 Hz, 1H), 3.76 (s, 3H), 3.72 – 3.67 (m, 1H), 3.45 – 3.42 (m, 1H), 3.01 (d, *J* = 14.2 Hz, 1H), 2.82 (d, *J* = 14.1 Hz, 1H), 2.14 – 2.06 (m, 1H), 2.05 – 1.98 (m, 1H), 1.71 – 1.60 (m, 2H), 1.57 – 1.47 (m, 1H), 1.21 (t, *J* = 7.1 Hz, 3H), 1.23 – 1.04 (m, 1H). **^{13}C NMR** (101 MHz, $CDCl_3$) δ 172.9, 158.5, 130.9 (2C), 128.8, 113.8 (2C), 77.6, 74.1, 68.3, 61.2, 57.3, 55.3, 55.2, 35.7, 27.3, 25.0, 15.2, 14.3. **HRMS (ESI) m/z:** $[M + Na]^+$ calcd for $C_{19}H_{24}NaO_5$ 355.1516, found 355.1513. **IR (KBr) ν:** 2933, 1726, 1512, 1248, 1178, 1099, 1034, 995, 750 cm^{-1}.

Ethyl 7-(4-(trifluoromethyl)benzyl)hexahydro-3*H*-oxireno[2,3-*d*]benzofuran-7-carboxylate (7d).

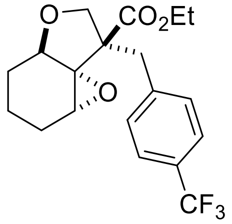

The title compound was prepared from compound **4d** (80.5 mg, 0.23 mmol) according to the General procedure for epoxidation and purified by column chromatography on silica gel (95/5 to 85/15 hexanes/ethyl acetate) to afford the product as a colourless oil (48 mg, 56%).

^{1}H NMR (400 MHz, $CDCl_3$) δ 7.52 – 7.47 (m, 2H), 7.33 – 7.28 (m, 2H), 4.37 (d, *J* = 9.4 Hz, 1H), 4.12 (q, *J* = 7.2 Hz, 2H), 3.77 (d, *J* = 9.5 Hz, 1H), 3.71 (dd, *J* = 10.4, 6.0 Hz, 1H), 3.41 –

3.38 (m, 1H), 3.12 (d, J = 14.0 Hz, 1H), 2.93 (d, J = 14.0 Hz, 1H), 2.15 – 2.08 (m, 1H), 2.07 – 1.99 (m, 1H), 1.74 – 1.58 (m, 1H), 1.56 – 1.49 (m, 1H), 1.20 (t, J = 7.1 Hz, 3H), 1.21 – 1.07 (m, 2H). **^{13}C NMR** (101 MHz, $CDCl_3$) δ 172.6, 141.1, 130.3 (2C), 129.2 (q, J = 32.4 Hz), 125.3 (q, J = 3.8 Hz, 2C), 124.3 (q, J = 271.9 Hz), 77.6, 74.0, 68.2, 61.4, 57.4, 54.9, 36.3, 27.2, 25.0, 15.1, 14.2. **HRMS (ESI) m/z:** $[M + Na]^+$ calcd for $C_{19}H_{21}F_3NaO_4$ 393.1284, found 393.1282. **IR (KBr) ν:** 2939, 1724, 1323, 1163, 1115, 1066, 1018, 995, 852, 621 cm^{-1}.

Ethyl 7-(4-methylbenzyl)hexahydro-3*H*-oxireno[2,3-*d*]benzofuran-7-carboxylate (7e).

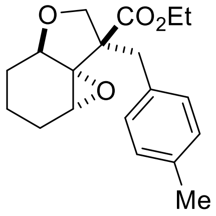

The title compound was prepared from compound **4e** (61 mg, 0.2 mmol) according to the General procedure for epoxidation and purified by column chromatography on silica gel (95/5 to 85/15 hexanes/ethyl acetate) to afford the product as a yellowish oil (55 mg, 70%).

^{1}H NMR (400 MHz, $CDCl_3$) δ 7.07 – 7.00 (m, 4H), 4.37 (d, J = 9.4 Hz, 1H), 4.12 (q, J = 7.1 Hz, 2H), 3.78 (d, J = 9.5 Hz, 1H), 3.73 – 3.65 (m, 1H), 3.52 – 3.40 (m, 1H), 3.03 (d, J = 14.0 Hz, 1H), 2.83 (d, J = 14.0 Hz, 1H), 2.29 (s, 3H), 2.16 – 1.97 (m, 2H), 1.72 – 1.61 (m, 1H), 1.60 – 1.39 (m, 1H), 1.33 – 1.23 (m, 1H), 1.21 (t, J = 7.1 Hz, 3H), 1.16 – 1.04 (m, 1H). **^{13}C NMR** (101 MHz, $CDCl_3$) δ 172.9, 136.4, 133.7, 129.7 (2C), 129.1 (2C), 77.6, 74.0, 68.3, 61.2, 57.2, 55.1, 36.2, 27.3, 25.0, 21.1, 15.2, 14.3. **HRMS (ESI) m/z:** $[M + Na]^+$ calcd for $C_{19}H_{24}NaO_4$ 339.1572, found 339.1564. **IR (KBr) ν:** 2935, 1726, 1217, 1186, 1101, 1061, 995, 731, 474, 409 cm^{-1}.

Ethyl 7-benzyl-5-tosyloctahydrooxireno[2,3-*d*]indole-7-carboxylate (8a).

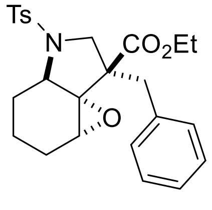

The title compound was prepared from compound **5a** (61 mg, 0.13 mmol) according to the General procedure for epoxidation and purified by column chromatography on silica gel (95/5 to 70/30 hexanes/ethyl acetate) to afford the product as oil a yellowish oil (55 mg, 47%).

^{1}H NMR (400 MHz, $CDCl_3$) δ 7.69 – 7.65 (m, 2H), 7.35 – 7.31 (m, 2H), 7.23 – 7.18 (m, 3H), 7.09 – 7.03 (m, 2H), 4.14 – 4.05 (m, 2H), 3.96 (d, J = 10.0 Hz, 1H), 3.36 – 3.32 (m, 1H), 3.12 (d, J = 10.1 Hz, 1H), 3.10 – 3.06 (m, 1H) 2.96 (d, J = 13.9 Hz, 1H), 2.74 (d, J = 13.9 Hz, 1H), 2.46 (s, 3H), 2.38 – 2.26 (m, 1H), 2.11 – 2.02 (m, 1H), 1.75 – 1.38 (m, 3H), 1.17 (t, J = 7.2 Hz, 3H), 1.18 – 1.08 (m, 1H).**^{13}C NMR** (101 MHz, $CDCl_3$) δ 171.7, 144.0, 136.1, 133.6, 130.0 (2C), 129.9 (2C), 128.5 (2C), 127.9 (2C), 127.1, 67.7, 61.6, 59.8, 57.2, 54.5, 53.6, 36.6, 27.6,

24.2, 21.7, 16.0, 14.2. **HRMS (ESI) m/z:** $[M + Na]^+$ calcd for $C_{25}H_{29}NNaO_5S$ 478.1664, found 478.1652. **IR (KBr) ν:** 2931, 1726, 1346, 1213, 1165, 1092, 1043, 733, 704, 663, 552 cm^{-1}.

Ethyl 7-(4-methoxybenzyl)-5-tosyloctahydrooxireno[2,3-*d*]indole-7-carboxylate (8c).

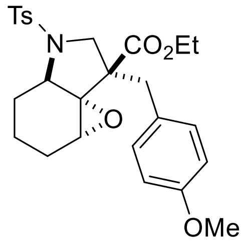

The title compound was prepared from compound **5c** (61 mg, 0.13 mmol) according to the General procedure for epoxidation and used for the next step as crude product.

Ethyl (1a*R*,2*R*,5*R*,5a*S*,8*R*,8a*R*)-8-benzylhexahydro-3*H*-2,5-epoxyfuro[2,3-*c*]oxireno[2,3-*d*]oxepine-8-carboxylate (9a).

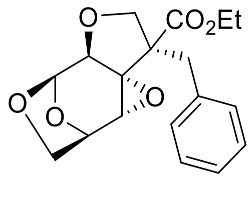

The title compound was prepared from compound **6a** (200 mg, 0.63 mmol) according to the General procedure for epoxidation and purified by column chromatography on silica gel (85/15 to 70/30 hexanes/ethyl acetate) to afford the product as an orange crystalline compound (111 mg, 56%).

m.p.: 114–116 °C; recrystallized from chloroform. **Specific rotation** $[\alpha]_D = -33.7^\circ$ (c 0.19, $CHCl_3$).

^{1}H NMR (400 MHz, $CDCl_3$) δ 7.30 – 7.18 (m, 3H), 7.18 – 7.10 (m, 2H), 5.50 (d, *J* = 2.7 Hz, 1H), 4.73 (d, *J* = 4.2 Hz, 1H), 4.47 (d, *J* = 9.5 Hz, 1H), 4.10 (q, *J* = 7.1 Hz, 2H), 3.98 (d, *J* = 7.5 Hz, 1H), 3.90 (d, *J* = 7.5 Hz, 1H), 3.85 (dd, *J* = 7.5, 4.3 Hz, 1H), 3.82 (d, *J* = 2.7 Hz, 1H), 3.48 – 3.45 (m, 1H), 3.19 (d, *J* = 13.9 Hz, 1H), 2.93 (d, *J* = 13.9 Hz, 1H), 1.19 (t, *J* = 7.1 Hz, 3H). **^{13}C NMR** (101 MHz, $CDCl_3$) δ 171.5, 136.2, 129.9 (2C), 128.6 (2C), 127.2, 98.0, 75.9, 73.7, 70.1, 68.0, 63.9, 61.6, 54.2, 54.0, 37.1, 14.2. **HRMS (ESI) m/z:** $[M + Na]^+$ calcd for $C_{18}H_{20}NaO_6$ 355.1152, found 355.1153. **IR (KBr) ν:** 2916, 2850, 1714, 1284, 1203, 1190, 1084, 982, 947, 885, 702, 663, 598, 492 cm^{-1}.

- General procedure for lactone formation *via* epoxide opening

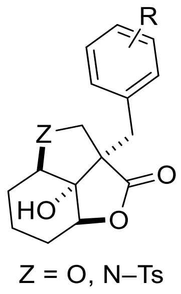

The resulted epoxide (1 mmol, 37 mM) was dissolved under an argon atmosphere in dry DCM (26.8 mL) and cooled to 0 °C in an ice bath, then Lewis acid (1.3 eq.) was added to the solution and continued to stir for 30 min up to 3 h and the progress of the reaction was monitored by TLC. The reaction was quenched with a saturated aqueous solution of $NaHCO_3$, and the reaction mixture was extracted with DCM. The combined organic layers were dried over sodium sulfate. The crude product was purified by column chromatography on silica gel (70/30 to 1/1 hexanes/ethyl acetate) to result final product.

- General procedure C for lactone formation without purification

The starting material **4**, **5** or **6** (1 mmol, 40 mM) was dissolved in DCM (25 ml), and then *m*-CPBA (2 mmol, 2 eq.) was added to the solution at room temperature and continued to stir overnight, with progress and reaction time monitored by TLC. The reaction mixture was quenched with a saturated aqueous solution of Na_2SO_3 (15 mL) and extracted with DCM (3 × 25 mL). The organic layers were combined and dried over sodium sulfate. The solvent was removed using a rotary evaporator. The resulting crude product was left to dry under high vacuum, and then it was dissolved in dry DCM (1.35 mL) under an argon atmosphere and cooled to 0 °C in an ice bath, then Lewis acid (1.3 eq.) was added to the solution and continued to stir for 1 hour. The reaction was quenched with a saturated aqueous solution of sodium bicarbonate $NaHCO_3$ (5 mL), and the reaction mixture was extracted with DCM (3 × 5 mL). The combined organic layers were dried over sodium sulfate. The crude product was purified by column chromatography on silica gel (70/30 hexanes/ethyl acetate).

2a-Benzyl-2a^{1}-hydroxyoctahydro-2*H* 1,4-dioxacyclopenta[*cd*]inden-2-one (10a).

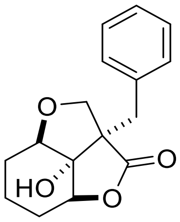

The title compound was prepared from compound **7a** (200 mg, 0.66 mmol) according to the General procedure for lactone formation using $BF_3{\cdot}OEt_2$ and purified by column chromatography on silica gel (70/30 to 1/1 hexanes/ethyl acetate) as a white crystalline compound (19 mg, 60%).

m.p.: 159–164 °C; the compound was recrystallized from chloroform.

^{1}H NMR (400 MHz, $CDCl_3$) δ 7.37 – 7.26 (m, 5H), 4.36 (d, *J* = 8.9 Hz, 1H), 3.78 – 3.73 (m, 1H), 3.67 – 3.64 (m, 1H), 3.62 (d, *J* = 9.0 Hz, 1H), 3.22 (d, *J* = 13.9 Hz, 1H), 2.94 (d, *J* = 13.9 Hz, 1H), 2.10 – 1.96 (m, 2H), 1.75 – 1.57 (m, 3H), 1.56 – 1.45 (m, 1H), 1.39 – 1.27 (m, 1H). **^{13}C NMR** (101 MHz, $CDCl_3$) δ 179.2, 135.7, 129.9 (2C), 129.2 (2C), 127.6, 83.8, 81.0, 79.7, 75.7, 61.0, 33.7, 25.7, 25.6, 12.5. **HRMS (ESI) m/z:** $[M + Na]^+$ calcd for $C_{16}H_{18}NaO_4$ 297.1097, found 297.1098. **IR (KBr) ν:** 3521, 2931, 2860, 1757, 1442, 1192, 1105, 1068, 1036, 987, 906, 698 cm^{-1}.

2a-(Benzo[*d*][1,3]dioxol-5-ylmethyl)-2a^1-hydroxyoctahydro-2*H*-1,4-dioxacyclopenta[*cd*]-inden-2-one (10b).

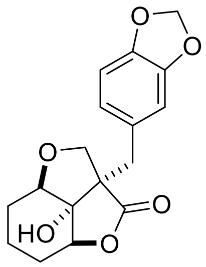

The title compound was prepared from compound **7b** (178 mg, 0.51 mmol) according to the General procedure for lactone formation using $BF_3 \cdot OEt_2$ and purified by column chromatography on silica gel (70/30 to 1/1 hexanes/ethyl acetate) as a white crystalline compound (106 mg, 64%).
The title compound was prepared in 82% NMR yield using $SnCl_4$.

m.p.: 144–150 °C; the compound was recrystallized from chloroform.
^{1}H NMR (600 MHz, $CDCl_3$) δ 6.85 (d, *J* = 1.7 Hz, 1H), 6.79 (dd, *J* = 7.9, 1.8 Hz, 1H), 6.73 (d, *J* = 7.9 Hz, 1H), 5.94 (s, 2H), 4.31 (d, *J* = 8.9 Hz, 1H), 3.85 – 3.82 (m, 1H), 3.76 – 3.73 (m, 1H), 3.58 (d, *J* = 9.0 Hz, 1H), 3.07 (d, *J* = 14.1 Hz, 1H), 2.88 (d, *J* = 14.1 Hz, 1H), 2.09 – 2.00 (m, 3H), 1.70 – 1.60 (m, 1H), 1.59 – 1.47 (m, 2H), 1.37 – 1.30 (m, 1H). **^{13}C NMR** (151 MHz, $CDCl_3$) δ 179.1, 148.2, 147.0, 129.2, 123.0, 110.3, 108.7, 101.3, 83.9, 81.0, 79.7, 75.6, 61.1, 33.3, 25.7, 25.6, 12.5. **HRMS (ESI) m/z:** $[M + Na]^+$ calcd for $C_{17}H_{18}NaO_6$ 341.0996, found 341.0992. **IR (KBr) ν:** 3510, 2868, 1759, 1489, 1439, 1238, 1211, 1072, 1039, 931, 752 cm^{-1}.

2a^1-Hydroxy-2a-(4-methoxybenzyl)octahydro-2*H*-1,4-dioxacyclopenta[*cd*]inden-2-one (10c).

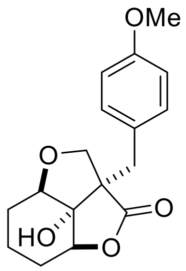

The title compound was prepared from compound **7c** (58 mg, 0.17 mmol) according to the General procedure for lactone formation using $SnCl_4$ and purified by column chromatography on silica gel (70/30 to 1/1 hexanes/ethyl acetate) as a white crystalline compound (49 mg, 95%).

m.p.: 153–158 °C; the compound was recrystallized from chloroform.
^{1}H NMR (400 MHz, $CDCl_3$) δ 7.28 – 7.23 (m, 2H), 6.86 – 7.81 (m, 2H), 4.32 (d, *J* = 8.9 Hz, 1H), 3.78 (s, 3H), 3.76 – 3.70 (m, 2H), 3.59 (d, *J* = 8.9 Hz, 1H), 3.12 (dd, *J* = 13.9, 2.8 Hz,

1H), 2.90 (d, J = 14.1 Hz, 1H), 2.15 – 1.93 (m, 3H), 1.71 – 1.57 (m, 1H), 1.58 – 1.45 (m, 2H), 1.38 – 1.27 (m, 1H). **^{13}C NMR** (101 MHz, $CDCl_3$) δ 179.3, 158.9, 131.0 (2C), 127.4, 114.5 (2C), 83.8, 81.0, 79.7, 75.6, 61.1, 55.3, 32.7, 25.7, 25.6, 12.5. **HRMS (ESI) m/z:** $[M + Na]^+$ calcd for $C_{17}H_{20}NaO_5$ 327.1203, found 327.1196. **IR (KBr) ν:** 3363, 2951, 1724, 1466, 1514, 1248, 1225, 1186, 1072, 1034, 989, 837, 600 cm^{-1}.

2a^1-Hydroxy-2a-(4-(trifluoromethyl)benzyl)octahydro-2*H*-1,4-dioxacyclopenta[*cd*]-inden-2-one (10d).

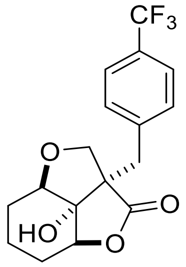

The title compound was prepared from compound **7e** (27 mg, 0.07 mmol) according to the General procedure for lactone formation using $BF_3{\cdot}OEt_2$ and purified by column chromatography on silica gel (70/30 to 1/1 hexanes/ethyl acetate)as a white crystalline compound (10 mg, 40%).
The title compound was prepared in 84% using $TiCl_4$.

m.p.: 164–166 °C; the compound was recrystallized from chloroform.
^{1}H NMR (400 MHz, $CDCl_3$) δ 7.58 – 7.53 (m, 2H), 7.50 – 7.46 (m, 2H), 4.35 (d, J = 9.0 Hz, 1H), 3.85 – 3.81 (m, 1H), 3.75 – 3.71 (m, 1H), 3.62 (d, J = 9.0 Hz, 1H), 3.14 (d, J = 13.8 Hz, 1H), 3.04 (d, J = 13.8 Hz, 1H), 2.11 – 1.99 (m, 2H), 1.82 (bs, 1H), 1.72 –1.45 (m, 4H). **^{13}C NMR** (101 MHz, $CDCl_3$) δ 178.7, 139.8, 130.8 (2C), 129.7 (q, J = 32.6 Hz), 125.6 (q, J = 3.7 Hz, 2C), 124.2 (d, J = 272.1 Hz, 84.4, 80.6, 79.4, 75.3, 61.2, 33.1, 25.7, 25.6, 12.4

2a^1-Hydroxy-2a-(4-methylbenzyl)octahydro-2*H*-1,4-dioxacyclopenta[*cd*]inden-2-one (10e).

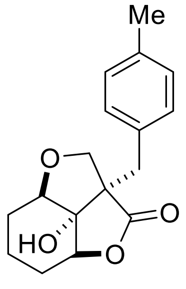

The title compound was prepared from compound **4e** (75 mg, 0,24 mmol) according to the General procedure C for lactone formation using $BF_3{\cdot}OEt_2$ and isolated as a white crystalline compound (51 mg, 68%).
The title compound was prepared from compound **7e** (11 mg, 0.04 mmol) according to the General procedure for lactone formation using $SnCl_4$ and purified by column chromatography on silica gel (70/30 to 1/1 hexanes/ethyl acetate) as a white crystalline compound (10 mg, 98%).

m.p.: 168–174 °C; the compound was recrystallized from chloroform.

^{1}H NMR (400 MHz, $CDCl_3$) δ 7.25 – 7.20 (m, 2H), 7.15 – 7.10 (m, 2H), 4.34 (d, J = 9.0 Hz, 1H), 3.77 – 3.73 (m, 1H), 3.69 – 3.64 (m, 1H), 3.61 (d, J = 8.9 Hz, 1H), 3.18 (d, J = 13.9 Hz, 1H), 2.89 (d, J = 14.1 Hz, 1H), 2.32 (s, 3H), 2.09 – 1.98 (m, 2H), 1.84 (bs, 1H), 1.68 – 1.57 (m, 1H), 1.55 – 1.45 (m, 2H), 1.35 – 1.29 (m, 1H). **^{13}C NMR** (101 MHz, $CDCl_3$) δ 179.2, 137.3, 132.5, 130.0 (2C), 129.6 (2C), 83.7, 81.1, 79.8, 75.8, 60.9, 33.3, 25.7, 25.6, 21.2, 12.5. **HRMS (ESI) m/z:** $[M + Na]^+$ calcd for $C_{17}H_{20}NaO_4$ 311.1254, found 311.1252. **IR (KBr) ν:** 3504, 2941, 1768, 1512, 1439, 1207, 1190, 1130, 1101, 1074, 989, 617, 567 cm^{-1}.

2a-(benzofuran-3-ylmethyl)-2a^1-hydroxyoctahydro-2*H*-1,4-dioxacyclopenta[*cd*]inden-2-one (10f).

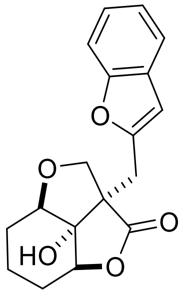

The title compound was prepared from compound **4f** (60 mg, 0.21 mmol) according to General procedure C for lactone formation using $SnCl_4$ and purified by column chromatography on silica gel (7/3 hexanes/ethyl acetate) to afford the product as a white solid (33 mg, 50 %).

m.p.: 165-172 °C.

^{1}H NMR (400 MHz, $CDCl_3$) δ 7.55 – 7.49 (m, 1H), 7.45 – 7.40 (m, 1H), 7.32 – 7.21 (m, 2H), 6.61 (s, 1H), 4.36 (d, J = 9.0 Hz, 1H), 4.03 – 4.00 (m, 1H), 3.89 – 3.86 (m, 1H), 3.64 (d, J = 9.0 Hz, 1H), 3.28 (bs, 1H), 3.26 (d, J = 15.0 Hz, 1H), 3.16 (d, J = 15.0 Hz, 1H), 2.13 – 2.03 (m, 2H), 1.75 – 1.48 (m, 3H), 1.41 – 1.32 (m, 1H). **^{13}C NMR** (101 MHz, $CDCl_3$) δ 177.9, 154.5, 152.4, 128.3, 124.7, 123.7, 121.5, 110.9, 106.6, 83.3, 81.4, 78.9, 74.9, 60.4, 26.4, 26.0, 25.9, 12.5. **HRMS (ESI) m/z:** $[M + H]^+$ calcd for $C_{18}H_{19}O_5$ 315.1227, found 315.122. **IR (KBr) ν:** 3377, 2948, 1738, 1456, 1321, 1234, 1157, 1072, 994, 756 cm^{-1}.

2a^1-hydroxy-2a-(2-methoxybenzyl)octahydro-2H-1,4-dioxacyclopenta[*cd*]inden-2-one (10k).

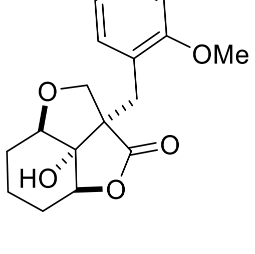

The title compound was prepared from compound **4k** (360 mg, 1.14 mmol) according to General procedure C for lactone formation using $SnCl_4$ and purified by column chromatography on silica gel (7/3 hexanes/ethyl acetate) to afford the product as a white solid (191 mg, 55 %).

m.p.: 156.7 °C.

^{1}**H NMR** (400 MHz, $CDCl_3$) δ 7.32 – 7.27 (m, 2H), 6.99 (t, *J* = 7.5 Hz, 1H), 6.93 (d, *J* = 8.6 Hz, 1H), 4.67 (bs, 1H), 4.36 (d, *J* = 8.7 Hz, 1H), 3.93 (s, 3H), 3.82 – 3.78 (m, 2H), 3.60 (d, *J* = 8.7 Hz, 1H), 3.17 (d, *J* = 14.2 Hz, 1H), 2.84 (d, *J* = 14.2 Hz, 1H), 2.07 – 1.93 (m, 2H), 1.74 – 1.41 (m, 3H), 1.35 – 1.23 (m, 1H). ^{13}**C NMR** (101 MHz, $CDCl_3$) δ 178.1, 155.7, 133.8, 129.2, 123.7, 122.6, 111.4, 84.0, 81.2, 78.2, 74.9, 62.4, 56.2, 26.1, 26.0, 25.8, 12.6. **HRMS (ESI) m/z:** $[M + Na]^+$ calcd for $C_{17}H_{20}NaO_5$ 327.1208, found 327.1203. **IR (KBr) ν:** 3506, 2939, 1177, 1495, 1240, 1178, 1115, 1072, 1038, 996, 762 cm^{-1}.

2a-(benzofuran-3-ylmethyl)-2a^1-hydroxyoctahydro-2*H*-1,4-dioxacyclopenta[*cd*]inden-2-one (10m).

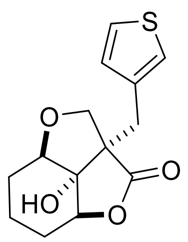

The title compound was prepared from compound **4m** (100 mg, 0.34 mmol) according to General procedure C for lactone formation using $SnCl_4$ and purified by column chromatography on silica gel (7/3 hexanes/ethyl acetate) to afford the product as a white solid (40 mg, 41 %).

m.p.: 161.7 °C.

^{1}**H NMR** (400 MHz, $CDCl_3$) δ 7.31 (dd, *J* = 4.9, 3.0 Hz, 1H), 7.20 – 7.18 (m, 1H), 7.06 (dd, *J* = 4.9, 1.4 Hz, 1H), 4.32 (d, *J* = 8.9 Hz, 1H), 3.77 – 3.74 (m, 1H), 3.74 – 3.71 (m, 1H), 3.58 (d, *J* = 9.0 Hz, 1H), 3.14 (d, *J* = 14.2 Hz, 1H), 3.04 (d, *J* = 14.2 Hz, 1H), 2.09 – 1.99 (m, 2H), 1.69 – 1.59 (m, 1H), 1.58 – 1.48 (m, 2H), 1.36 – 1.31 (m, 1H). ^{13}**C NMR** (101 MHz, $CDCl_3$) δ 179.4, 135.9, 128.9, 127.0, 123.7, 83.7, 81.3, 79.5, 75.5, 60.6, 28.1, 25.7, 25.7, 12.5. **HRMS (ESI) m/z:** $[M + Na]^+$ calcd for $C_{14}H_{16}$NaO4S 303.0661, found 303.0658. **IR (KBr) ν:** 35114, 2941, 1757, 1212, 1075, 1038, 985, 757, 773, 642 cm^{-1}.

2a-(4-acetylbenzyl)-2a^1-hydroxyoctahydro-2*H*-1,4-dioxacyclopenta[*cd*]inden-2-one (10p).

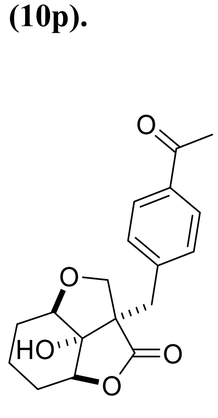

The title compound was prepared from compound **4p** (57 mg, 0.17 mmol) according to General procedure C for lactone formation using $SnCl_4$ and purified by column chromatography on silica gel (6/4 hexanes/ethyl acetate) to afford the product as a white solid (44 mg, 80 %).

m.p.: 151.7-154.4 °C.

^{1}**H NMR** (400 MHz, $CDCl_3$) δ 7.88 (d, *J* = 8.3 Hz, 2H), 7.46 (d, *J* = 8.3 Hz, 1H), 4.34 (d, *J* = 8.9 Hz, 1H), 3.85 – 3.81 (m, 1H), 3.77 – 3.73 (m, 1H), 3.63 (d, *J* = 9.0 Hz,

1H), 3.14 (d, J = 13.7 Hz, 1H), 3.05 (d, J = 13.7 Hz, 1H), 2.59 (s, 2H), 2.09 – 1.98 (m, 1H), 1.66 (m, 1H), 1.58 – 1.47 (m, 2H), 1.34 (m, 1H). **^{13}C NMR** (101 MHz, $CDCl_3$) δ 198.2, 178.7, 141.6, 136.1, 130.7 (2C), 128.8 (2C), 84.3, 80.6, 79.4, 75.4, 61.2, 33.3, 26.8, 25.7, 25.7, 12.5. **HRMS (ESI) m/z:** $[M + Na]^+$ calcd for $C_{18}H_{20}NaO_5$ 339.1203, found 339.1205. **IR (KBr) ν:** 3376, 2945, 1736, 1684, 1265, 1219, 1077, 994, 600, 581 cm^{-1}.

2a-Benzyl-2a^1-hydroxy-4-tosyloctahydrofuro[4,3,2-*cd*]indol-2(2a*H*)-one (11a).

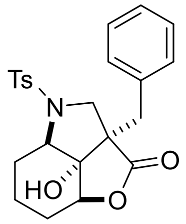

The title compound was prepared from compound **8a** (18.7 mg, 0.04 mmol) according to the General procedure for lactone formation using $SnCl_4$ and purified by column chromatography on silica gel (75/25 to 1/1 hexanes/ethyl acetate) as a white crystalline product (17 mg, 91%).

m.p.: 174–180 °C; the compound was recrystallized from chloroform.

^{1}H NMR (400 MHz, $CDCl_3$) δ 7.70 – 7.64 (m, 2H), 7.39 – 7.33 (m, 2H), 7.34 – 7.21 (m, 5H), 4.02 (d, J = 9.3 Hz, 1H), 3.58 – 3.51 (m, 1H), 3.15 (d, J = 13.6 Hz, 1H), 3.00 – 2.92 (m, 1H), 2.90 – 2.82 (m, 2H), 2.54 – 2.41 (m, 1H), 2.46 (s, 3H), 2.03 – 1.89 (m, 1H), 1.82 (bs, 1H), 1.59 – 1.44 (m, 3H), 1.37 – 1.26 (m, 1H). **^{13}C NMR** (101 MHz, $CDCl_3$) δ 177.6, 144.6, 135.2, 132.3, 130.1 (2C), 130.0 (2C), 129.2 (2C), 128.2 (2C), 127.8, 81.4, 80.8, 66.3, 57.6, 56.9, 35.0, 24.8, 24.3, 21.8, 13.0. **HRMS (ESI) m/z:** $[M + H]^+$ calcd for $C_{23}H_{26}NO_5S$ 428.1526, found 428.1540. **IR (KBr) ν:** 3340, 2925, 1739, 1346, 1163, 1078, 1001, 727, 700, 663, 580, 548 cm^{-1}.

2a-Benzyl-2a^1-hydroxy-4-tosyloctahydrofuro[4,3,2-*cd*]indol-2(2a*H*)-one (11c).

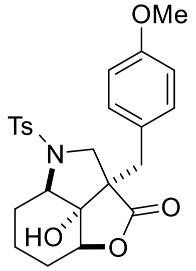

The title compound was prepared from compound **8c** (18 mg, 0.04 mmol) according to the General procedure C for lactone formation using $SnCl_4$ and purified by column chromatography on silica gel (75/25 to 1/1 hexanes/ethyl acetate) as a yellowish oil (17 mg, 92%).

^{1}H NMR (400 MHz, $CDCl_3$) δ 7.69 – 7.64 (m, 2H), 7.39 – 7.32 (m, 2H), 7.18 (d, J = 8.3 Hz, 2H), 6.81 (d, J = 8.3 Hz, 2H), 4.00 (d, J = 9.5 Hz, 1H), 3.77 (s, 3H), 3.59 – 3.54 (m, 1H), 3.08 (d, J = 13.9 Hz, 1H), 2.98 – 2.92 (m, 1H), 2.88 – 2.77 (m, 2H), 2.52 – 2.42 (m, 1H), 2.46 (s, 3H), 2.01 – 1.90 (m, 1H), 1.74 (bs, 1H), 1.59 – 1.45 (m, 3H), 1.37 – 1.27 (m, 1H).**^{13}C NMR** (101 MHz, $CDCl_3$) δ 177.8, 159.1, 144.6, 132.3, 131.1 (2C), 130.0 (2C), 128.2 (2C), 126.9, 114.6 (2C), 81.4, 80.8, 66.3, 57.5, 56.9, 55.4, 34.2, 24.8, 24.3, 21.8, 13.1. **HRMS**

(ESI) m/z: [M + Na]$^+$ calcd for $C_{24}H_{27}NNaO_6S$ 480.1457, found 480.1453. **R (KBr) ν:** 3342, 2927, 1739, 1342, 1159, 1074, 812, 723, 661, 575, 548 cm^{-1}.

(2a*R*,2a^1*R*,4a*S*,5*R*,8*R*,8a*S*)-2a-Benzyl-2a^1-hydroxyoctahydro-2*H*-1,4,6,9-tetraoxa-5,8-methanocyclopenta[*cd*]azulen-2-one (12a).

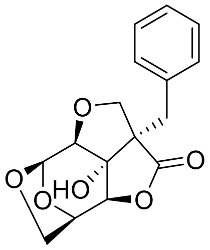

The title compound was prepared from compound **5e** (18.7 mg, 0.06 mmol) according to the General procedure for lactone formation using $SnCl_4$ and purified by column chromatography on silica gel (75/25 to 1/1 hexanes/ethyl acetate) as a white crystalline product (17 mg, 95%).

m.p.: 174–180 °C; the compound was recrystallised from chloroform. **Specific rotation** $[\alpha]_D = -50.7^\circ$ (c 0.16, $CHCl_3$).
^{1}H NMR (400 MHz, $CDCl_3$) δ 7.41 – 7.32 (m, 2H), 7.32 – 7.20 (m, 3H), 5.72 (d, *J* = 5.0 Hz, 1H), 4.79 – 4.76 (m, 1H), 4.49 (d, *J* = 9.1 Hz, 1H), 4.27 – 4.23 (m, 1H), 4.13 (d, *J* = 8.1 Hz, 1H), 4.04 – 4.01 (m, 1H), 3.80 (d, *J* = 9.2 Hz, 1H), 3.60 (dd, *J* = 8.0, 4.9 Hz, 1H), 3.1 – 3.01 (m, 2H), 2.52 (s, 1H). **^{13}C NMR** (101 MHz, $CDCl_3$) δ 177.5, 135.1, 130.5 (2C), 128.7 (2C), 127.5, 97.1, 88.3, 80.4, 79.9, 76.4, 71.0, 64.0, 60.2, 33.5. **HRMS (ESI) m/z:** [M + Na]$^+$ calcd for $C_{16}H_{16}NaO_6$ 327.0839, found 327.0839. **IR (KBr) ν:** 3359, 2922 1741, 1373, 1200, 1132, 1055, 1020, 968, 700 cm^{-1}.

4.2. Lactone formation *via* halolactonisation

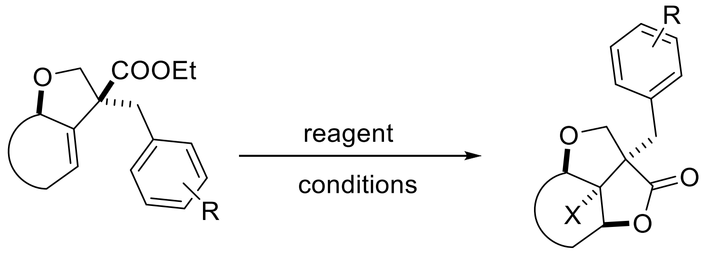

Table S4: Halolactonisation experiments.

Entry	Substrate	Reagent	Eq.	Solvent	Temp. (°C)	Yield (%)
1	**4a**	BDSB	1.2	MeCN	0 to 50	0
2	**4b**	NIS	6	MeCN	50 to 110	0
3	**4a**	I_2	6	MeCN/H_2O	50 to 110	**15**[a]
4	**6a**	I_2	6	THF/ H_2O	80 to 110	0
5	**6a**	NIS	6	MeCN/ H_2O	80 to 110	0
6	**6a**	NIS	6	THF/ H_2O	80 to 110	0

a) ^{1}HNMR yields using 3,4,5-trichloropyridine as an internal standard.

2a-Benzyl-2a[1]-iodooctahydro-2*H*-1,4-dioxacyclopenta[*cd*]inden-2-one (15a).

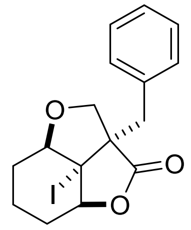

The compound **4a** (24 mg, 0.083 mmol, 0.166 M) was dissolved in a mixture of 2:1 ratio of CH_3CN/H_2O (0.5 mL), then I_2 (63 mg, 0.48 mmol, 6eq.) was added, and the reaction mixture was stirred at 50 °C in an oil bath and heated up to 110 °C for several hours. The progress of the reaction was monitored by TLC, and the reaction was quenched with a saturated aqueous solution of $Na_2S_2O_3$ (15 mL) and extracted between H_2O (15 mL) and ethyl acetate (3 × 20 mL). The combined organic layers were dried over Na_2SO_4, filtered, and concentrated under reduced pressure. The crude product was purified by column chromatography on silica gel (70/30 hexanes/ethyl acetate) to result in a yellow oil (2.49 mg, 15%).

Some traces of dba[4] in the sample were observed.
^{1}H NMR (400 MHz, $CDCl_3$) δ 7.46 – 7.35 (m, 3H), 7.32 – 7.27 (m, 2H), 4.71 – 4.68 (m, 1H), 4.50 (dd, *J* = 3.2, 3.2 Hz, 1H), 4.28 (d, *J* = 8.9 Hz, 1H), 3.60 (d, *J* = 8.9 Hz, 1H), 3.43 (d, *J* = 14.4 Hz, 1H), 3.08 (d, *J* = 14.4 Hz, 1H), 2.07 – 1.85 (m, 4H), 1.69 – 1.53 (m, 1H), 1.44 – 1.33 (m, 1H). **^{13}C NMR** (101 MHz, $CDCl_3$) δ 175.8, 135.4, 130.6 (2C), 128.5 (2C), 127.4, 89.5, 83.5, 75.5, 61.4, 47.6, 40.9, 24.4, 24.3, 11.8. **HRMS (ESI) m/z:** $[M + Na]^+$ calcd for $C_{16}H_{17}INaO_3$ 407.0115, found 407.0118. **IR (KBr) ν:** 2941, 1770, 1452, 1211, 1190, 1095, 987, 926, 814, 752, 698 cm^{-1}.

4. X-ray structure data of compounds 9a and 10b

The diffraction experiment for crystal structure determination of **9a** and **10b** were performed on Bruker D8 VENTURE Kappa Duo PHOTONIII by IμS micro-focus sealed tube at low temperature preserved by Cryostream Cooler. The structures were solved by direct methods (XT)[6] and refined by full matrix least squares based on F^2 (SHELXL2019)[7]. The hydrogen atoms on carbon were fixed into idealized positions (riding model) and assigned temperature factors either $H_{iso}(H) = 1.2\ U_{eq}$(pivot atom) or $H_{iso}(H) = 1.5\ U_{eq}$(pivot atom) for methyl moiety. The hydrogen atoms in -O-H moiety was found on difference Fourier map and refined under rigid-body approximation. The determination of absolute configuration[8] of chiral **9a** was based on anomalous dispersion of oxygen atoms. Basic crystallographic data are given in Table S5.

X-ray crystallographic data have been deposited with the Cambridge Crystallographic Data Centre (CCDC 2385726 and 2385725), the deposition numbers are in Table S5 and can be obtained free of charge from the Centre via its website (www.ccdc.cam.ac.uk/structures/).

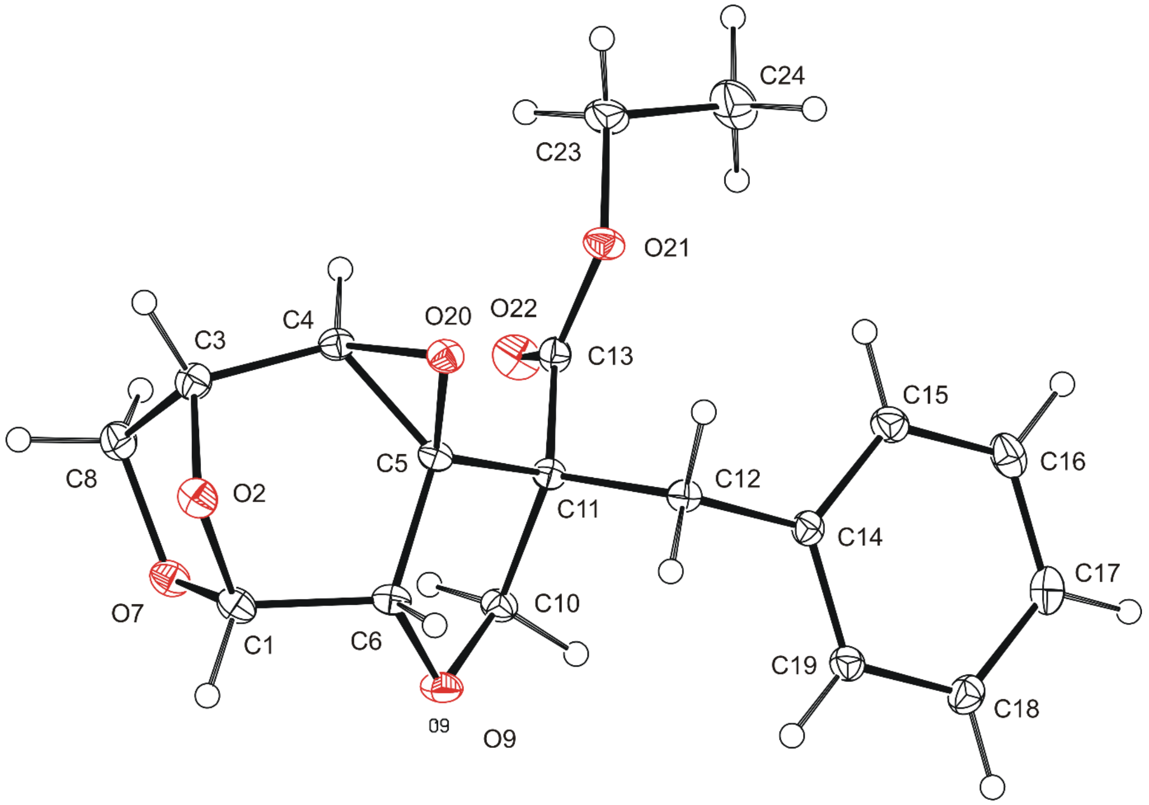

Figure S1: View on the molecule of **9a** with atom numbering schema. The displacement ellipsoids are drawn on 30% probability level. The chirality parameters *R*, *R*, *R*, *S*, *S* and *R* for C1, C3, C4, C5, C6 and C11, respectively

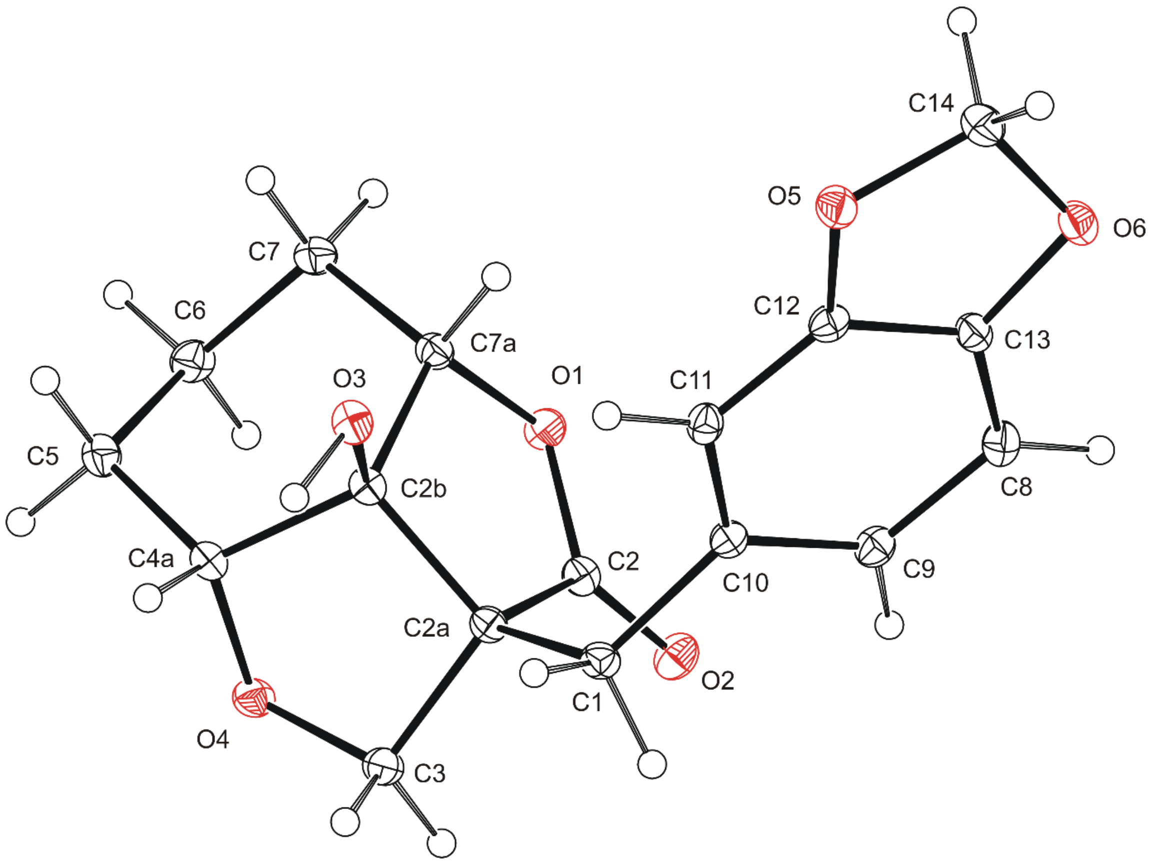

Figure 2: View on the molecule of **10b** with atom numbering schema. The displacement ellipsoids are drawn on 30% probability level.

Table S5: Crystal data, data collection, and refinement parameters for **9a** and **10b**

**Compound**	**9a**	**10b**
CCDC	2385726	2385725
Formula	$C_{18}H_{20}O_6$	$C_{17}H_{18}O_6$
M.w.	332.34	318.31
Crystal system	Monoclinic	Triclinic
Space group	$P\ 2_1$ (No. 4)	P -1 (No. 2)
a [Å]	11.0773 (7)	6.4653 (2)
b [Å]	6.6200 (4)	11.0379 (3)
c [Å]	11.1302 (8)	11.4037 (3)
α [°]	90	109.960 (1)
β [°]	104.129 (2)	90.674 (1)
γ [°]	90	104.580 (1)
Z	2	2
V[Å^3]	791.51 (9)	736.01 (4)
Temperature	120	120
D_x [g cm^{-3}]	1.394	1.436
Wavelength, Å	1.54178	1.54178
Crystal size [mm]	0.82 × 0.15 × 0.09	0.48 × 0.05 × 0.04
Crystal color, shape	bar, colourless	needle, colourless
μ [mm^{-1}]	0.87	0.91
T_{min},T_{max}	0.71, 0.93	0.52, 0.66
Measured reflections	23821	13646
Independent diffractions ($R_{int}{}^{a}$)	3353, (0.031)	2874, (0.047)
Observed diffract. [I>2σ(I)]	3323	2689
No. of parameters	218	208
R^b	0.026	0.036
$wR(F^2)$ for all data	0.068	0.090
GOF^c	1.05	1.05
Residual electron density [e/Å^3]	0.19, −0.17	0.33, −0.28
Absolute structure parameter	-0.03(4)	

$^{a}R_{int} = \Sigma|F_o^2 - F_{o,mean}^2|/\Sigma F_o^2$; $^{b}R(F) = \Sigma||F_o| - |F_c||/\Sigma|F_o|$; $wR(F^2) = [\Sigma(w(F_o^2 - F_c^2)^2)/(\Sigma w(F_o^2)^2)]^{½}$;
$^{c}GOF = [\Sigma(w(F_o^2 - F_c^2)^2)/(N_{diffrs} - N_{params})]^{½}$

5. Cytotoxicity screening

Method:

Cytotoxicity of compounds **9a**, **10a**, **10b**, **10c**, **10d**, **10e** and **12a** was evaluated in four cancer cell lines (CCRF-CEM, HepG2, Hela S3, HL-60) and non-tumour human dermal fibroblasts (NHDF). All cell lines were from ATCC (Manassas, VA, USA). The cells were maintained in RPMI-1640 or DMEM culture medium containing 10% FBS and 1% GlutaMax without antibiotics. Cells were seeded in 384-well white plates (Thermo Fisher Scientific, Waltham, USA) at a concentration between 2,000 – 50,000 cells per well and left to rest overnight. The next day, indicated concentrations of the test compounds were added, the cells were incubated at 37 °C, 5% CO_2 for 72 h after which CellTiter-Glo® 2.0 detection reagent (Promega, Madison, USA) was added. The plate was left on a shaker (350 rpm) for 20 min at room temperature. Luminescence was measured by a multimode plate reader. The signal of the compound-treated cells was related to the value of untreated control which was arbitrarily set to 100% viability. IC_{50} values were calculated from dose-response curves using non-linear regression method using GraphPad Prism software.

Cell lines:

CCRF-CEM - human T-lymhpoblastic leukemia (suspension)

HL-60 - human promyelocytic leukemia (suspension)

HeLa - human cervical carcinoma (adherent)

HepG2 - human hepatocellular carcinoma (adherent)

NHDF - human normal dermal fibroblasts (adherent)

Results:

Compound	CEM		HL60		HeLa		HepG2		NHDF	
	% of ctrl	SD	% of ctrl	SD	% of ctrl	SD	% of ctrl	SD	% of ctrl	sd
**9a**	74	4	95	2	105	2	91	2	109	3
**10a**	92	7	96	2	102	3	91	1	102	5
**10b**	100	9	96	2	101	0	94	0	101	3
**10c**	98	4	98	2	103	2	95	1	103	4
**10d**	72	8	91	3	103	1	88	3	106	1
**10e**	96	9	102	1	102	2	94	1	104	1
**12a**	110	6	96	3	103	1	97	6	100	4

*percentage of viability of compound-treated cells (10 µM) vs. untreated cells

6. References

(1) Nechaev, A. A.; Jagtap, P. R.; Bažíková, E.; Neumannová, J.; Císařová, I.; Matoušová, E. Synthesis of Fused 1,2-Naphthoquinones with Cytotoxic Activity Using a One-Pot Three-Step Reaction. *Org. Biomol. Chem.* **2021**, *19* (15), 3434–3440. https://doi.org/10.1039/D1OB00205H.

(2) Patil, S.; Chen, L.; Tanko, J. M. C–H Bond Functionalization with the Formation of a C–C Bond: A Free Radical Condensation Reaction Based on the Phthalimido-N-Oxyl Radical. *Eur. J. Org. Chem.* **2014**, *2014* (3), 502–505. https://doi.org/10.1002/ejoc.201301530.

(3) Xuan, J.; Daniliuc, C. G.; Studer, A. Construction of Polycyclic γ-Lactams and Related Heterocycles via Electron Catalysis. *Org. Lett.* **2016**, *18* (24), 6372–6375. https://doi.org/10.1021/acs.orglett.6b03267.

(4) Lin, D.; Yang, H.; Zhang, X.; Sun, H.; Zhang, X.; Jian, Y.; Zhang, W.; Liu, Y.; Gao, Z. Palladium-Catalysed Suzuki–Miyaura Coupling of α,β-Unsaturated Superactive Triazine Esters. *Chem. Commun.* **2023**, *59* (32), 4810–4813. https://doi.org/10.1039/D3CC00336A.

(5) Kallitsakis, M. G.; Gioftsidou, D. K.; Tzani, M. A.; Angaridis, P. A.; Terzidis, M. A.; Lykakis, I. N. Selective C–H Allylic Oxygenation of Cycloalkenes and Terpenoids Photosensitized by [Cu(Xantphos)(Neoc)]BF4. *J. Org. Chem.* **2021**, *86* (19), 13503–13513. https://doi.org/10.1021/acs.joc.1c01591.

(6) Sheldrick, G. M. SHELXT – Integrated Space-Group and Crystal-Structure Determination. *Acta Crystallogr. A: Found. Adv.* **2015**, *71* (1), 3–8. https://doi.org/10.1107/S2053273314026370.

(7) Sheldrick, G. M. Crystal Structure Refinement with SHELXL. *Acta Crystallogr. C: Struct. Chem.* **2015**, *71* (1), 3–8. https://doi.org/10.1107/S2053229614024218.

(8) Parsons, S.; Flack, H. D.; Wagner, T. Use of Intensity Quotients and Differences in Absolute Structure Refinement. *Acta Crystallogr. B: Struct. Sci. Cryst. Eng. Mater.* **2013**, *69* (Pt 3), 249–259. https://doi.org/10.1107/S2052519213010014.

7. Copies of ^{1}H and ^{13}C NMR spectra

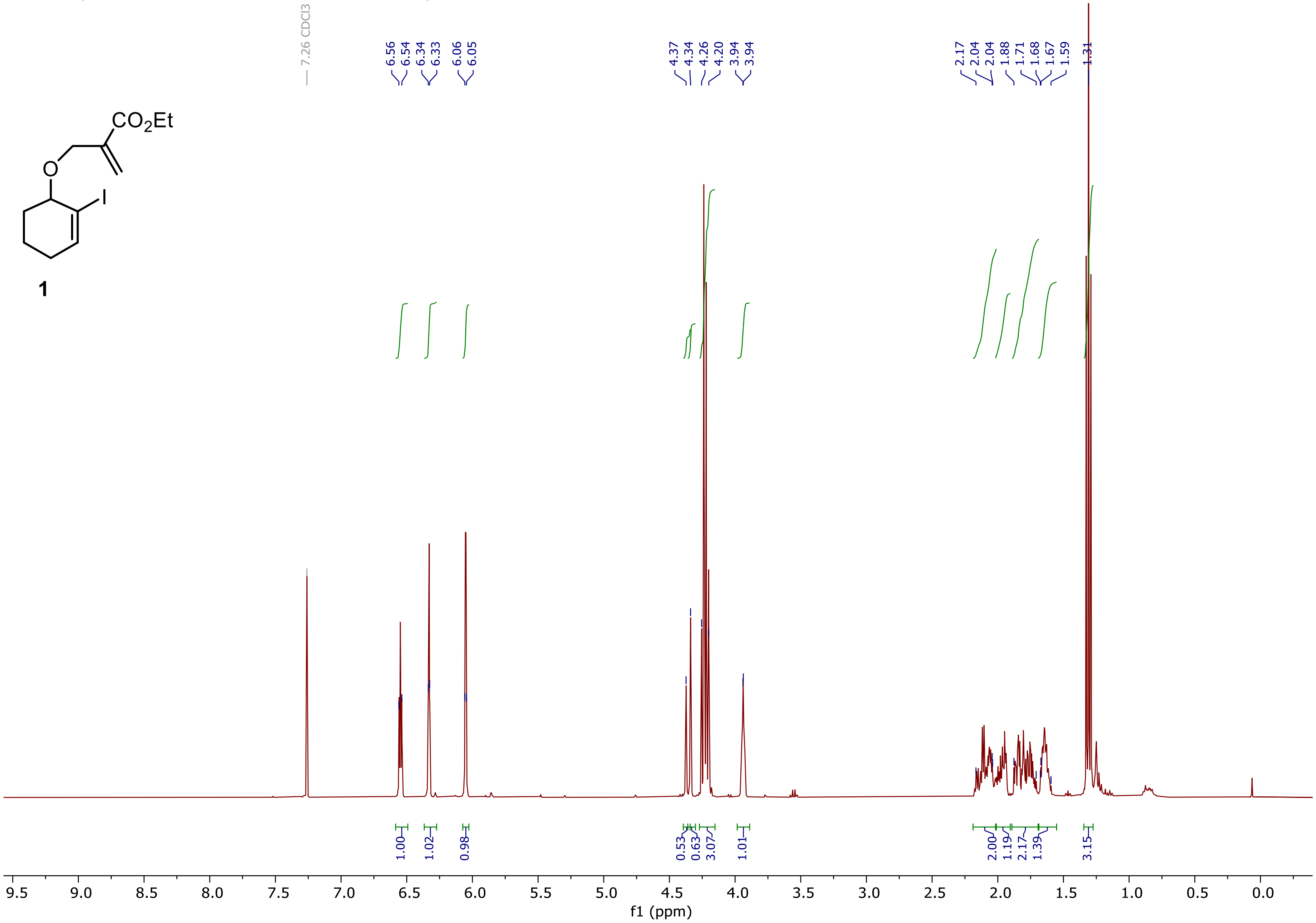

^{1}H NMR spectrum (400 MHz, $CDCl_3$) of compound **1**

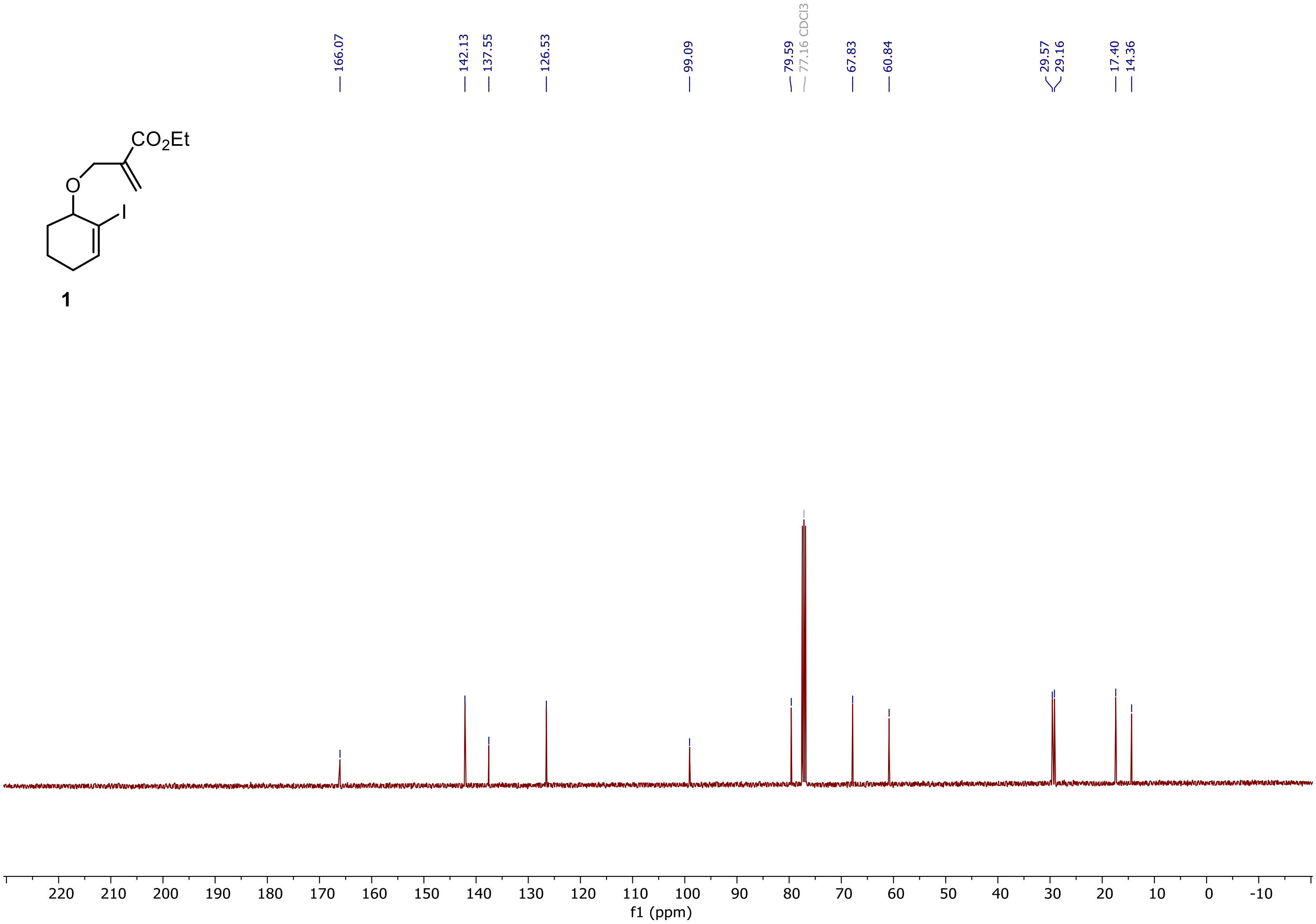

^{13}C NMR spectrum (101 MHz, $CDCl_3$) of compound **1**

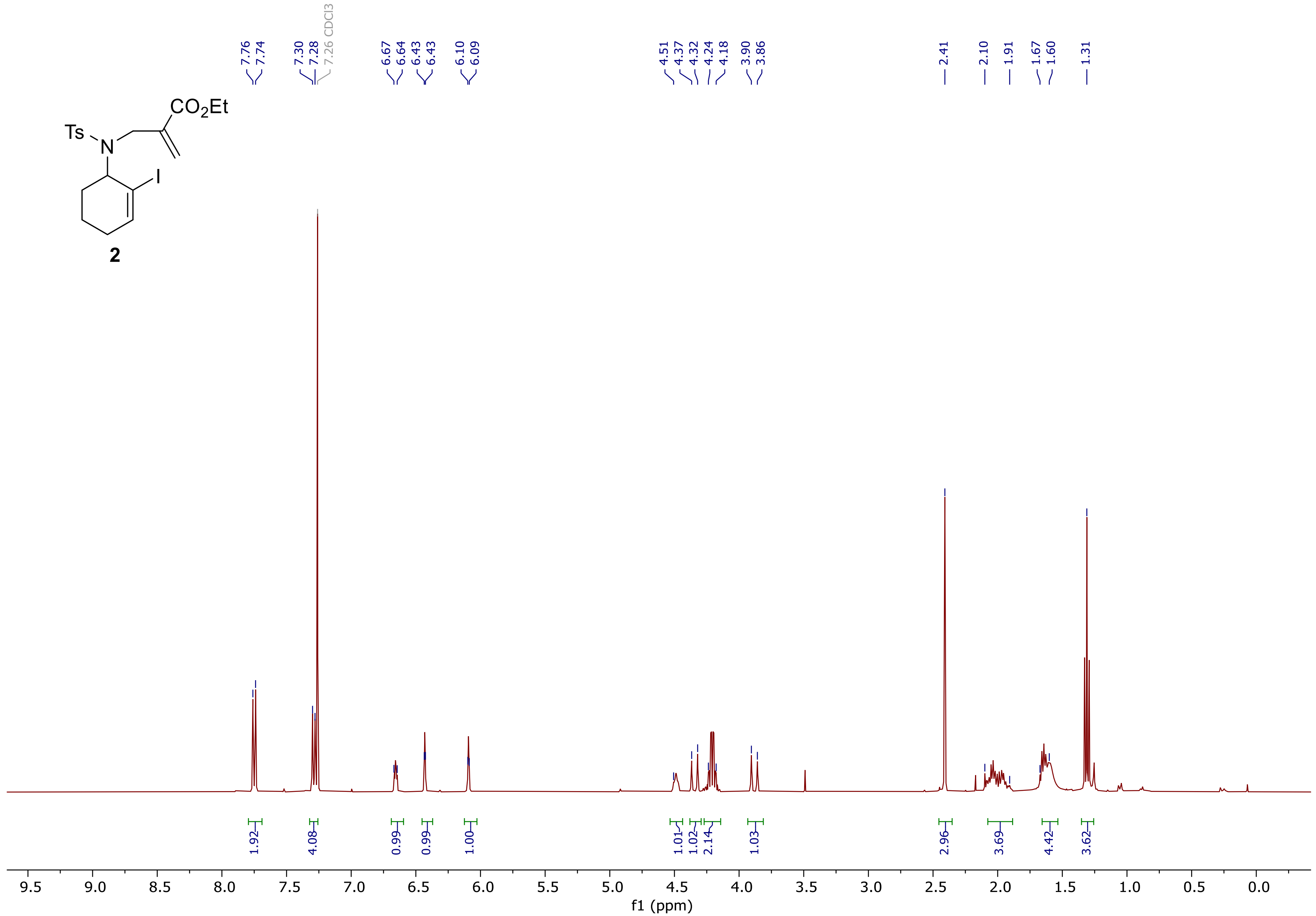

^{1}H NMR spectrum (400 MHz, $CDCl_3$) of compound **2**

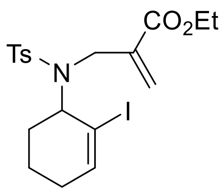

2

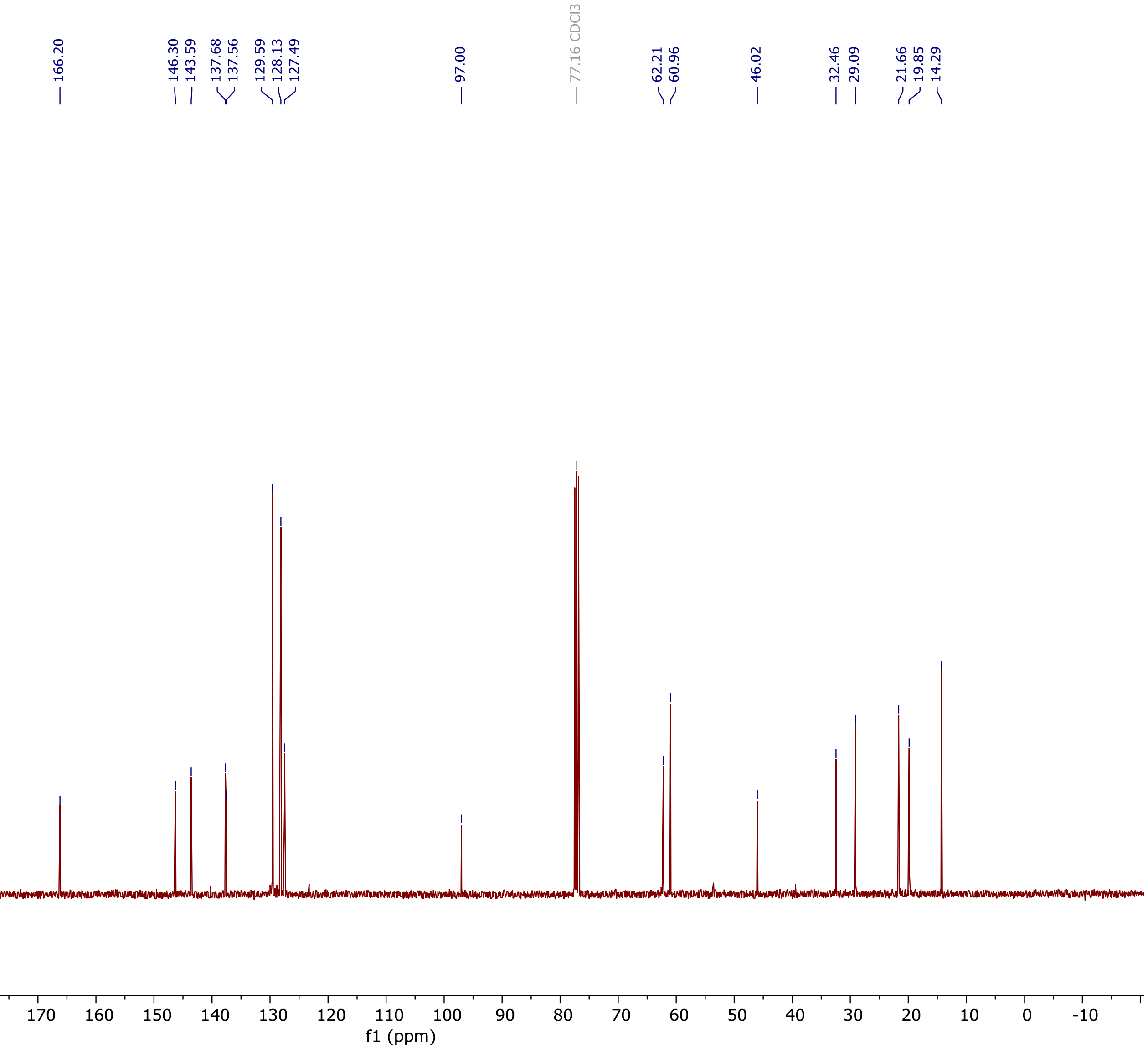

^{13}C NMR spectrum (101 MHz, $CDCl_3$) of compound **2**

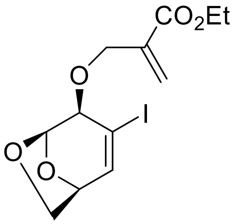

3

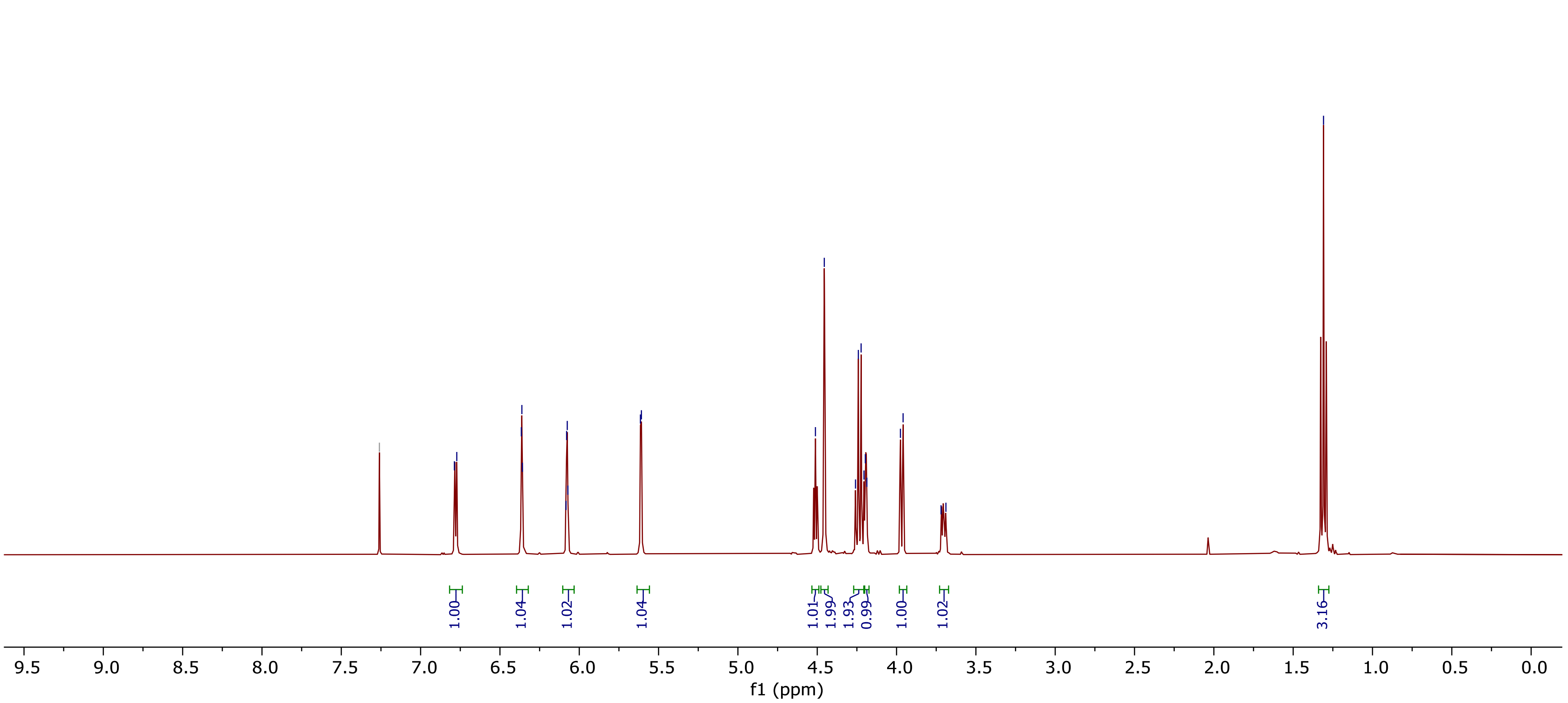

^{1}H NMR spectrum (400 MHz, $CDCl_3$) of compound **3**

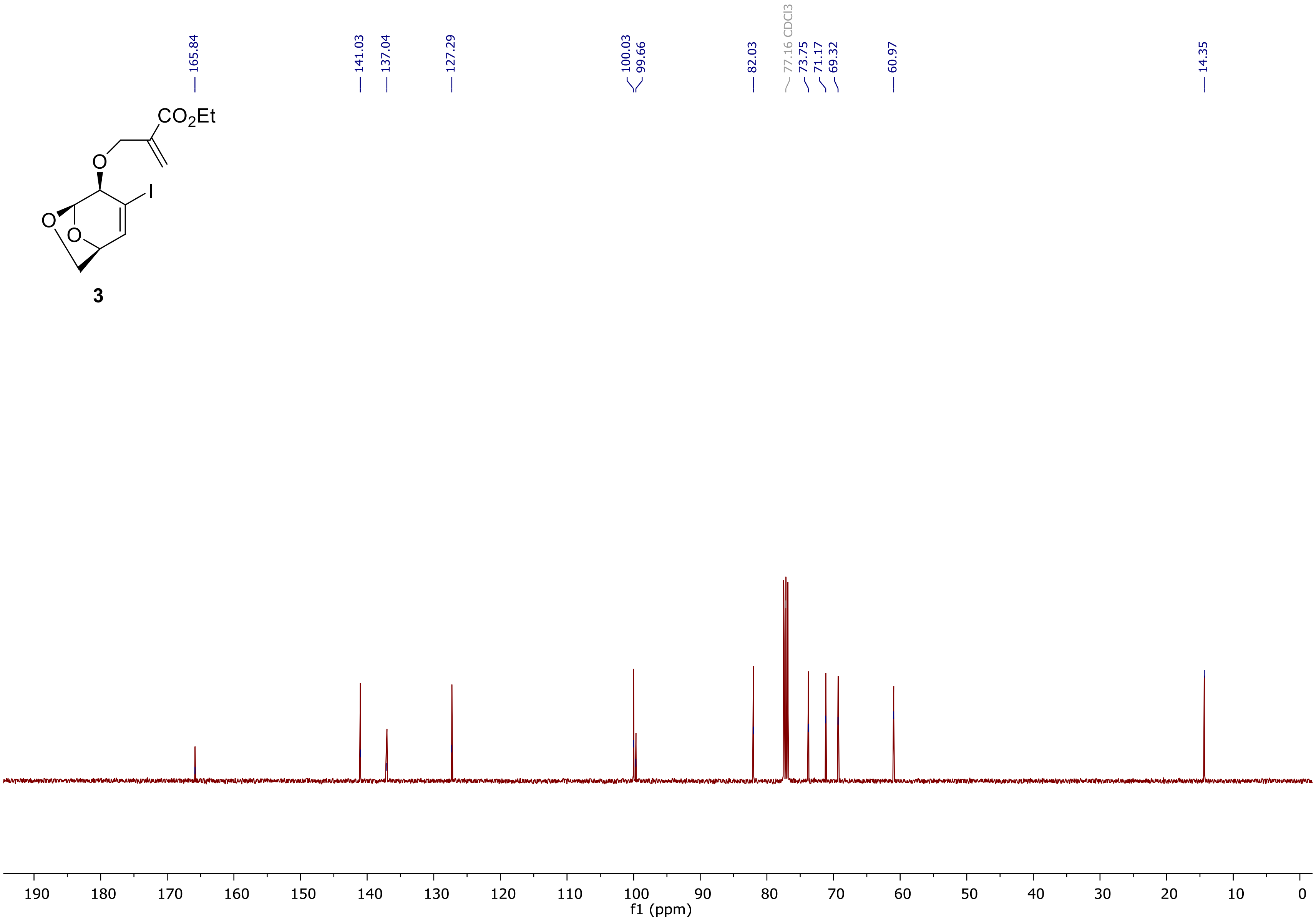

^{13}C NMR spectrum (101 MHz, $CDCl_3$) of compound **3**

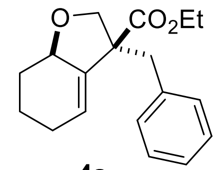

4a

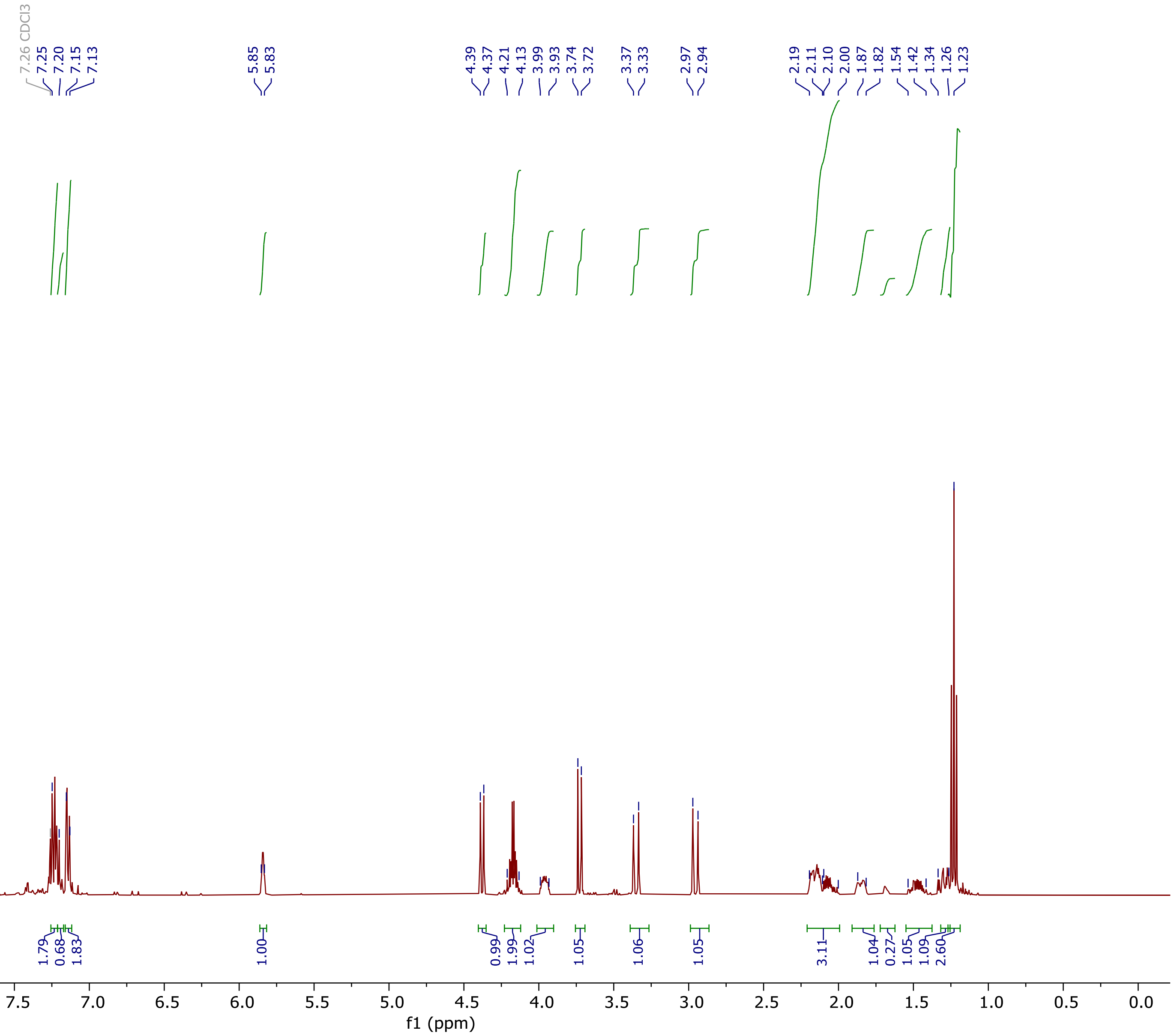

^{1}H NMR spectrum (400 MHz, $CDCl_3$) of compound **4a**

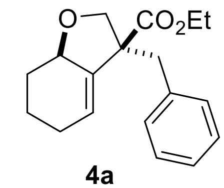

4a

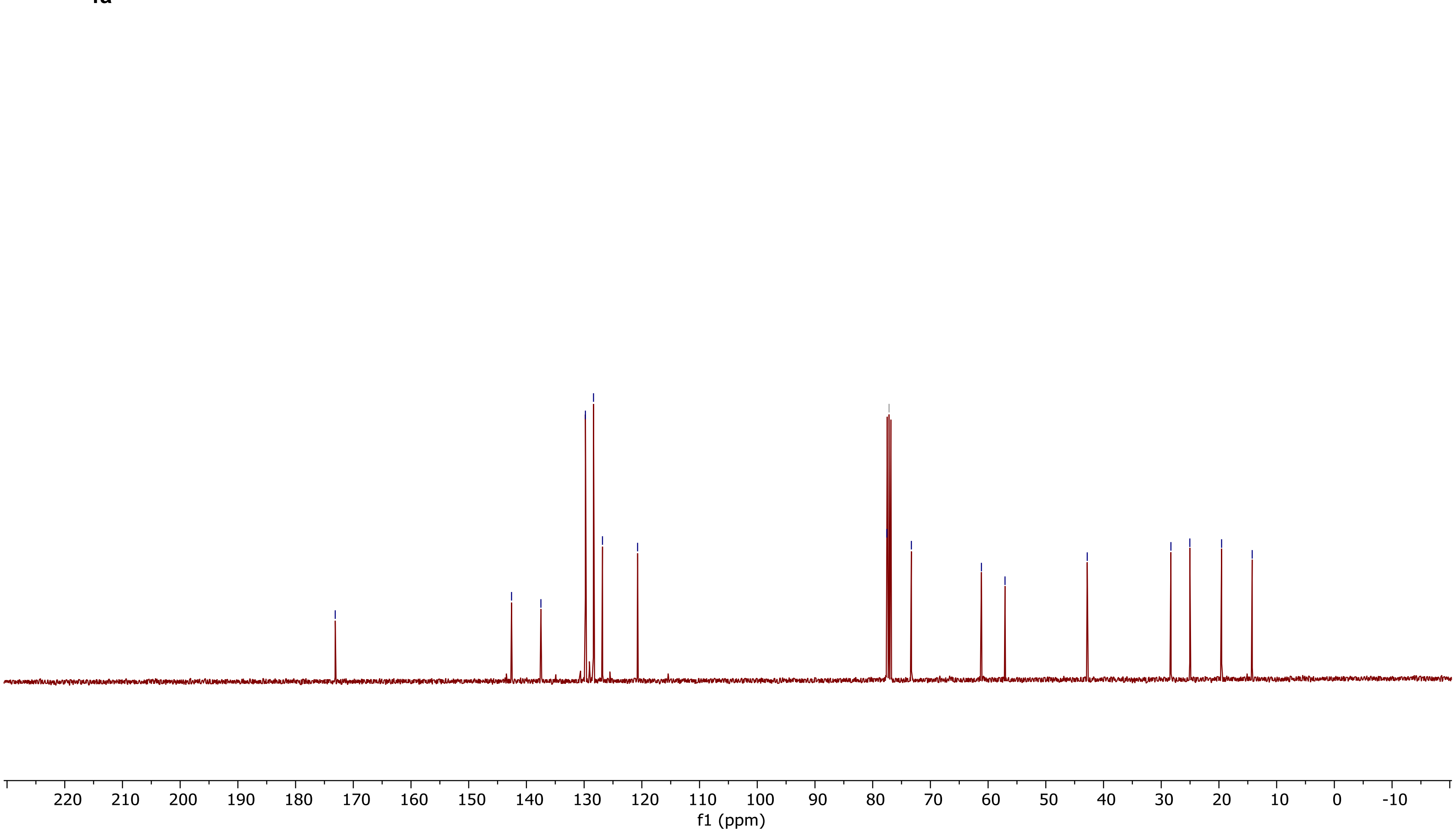

^{13}C NMR spectrum (101 MHz, $CDCl_3$) of compound **4a**

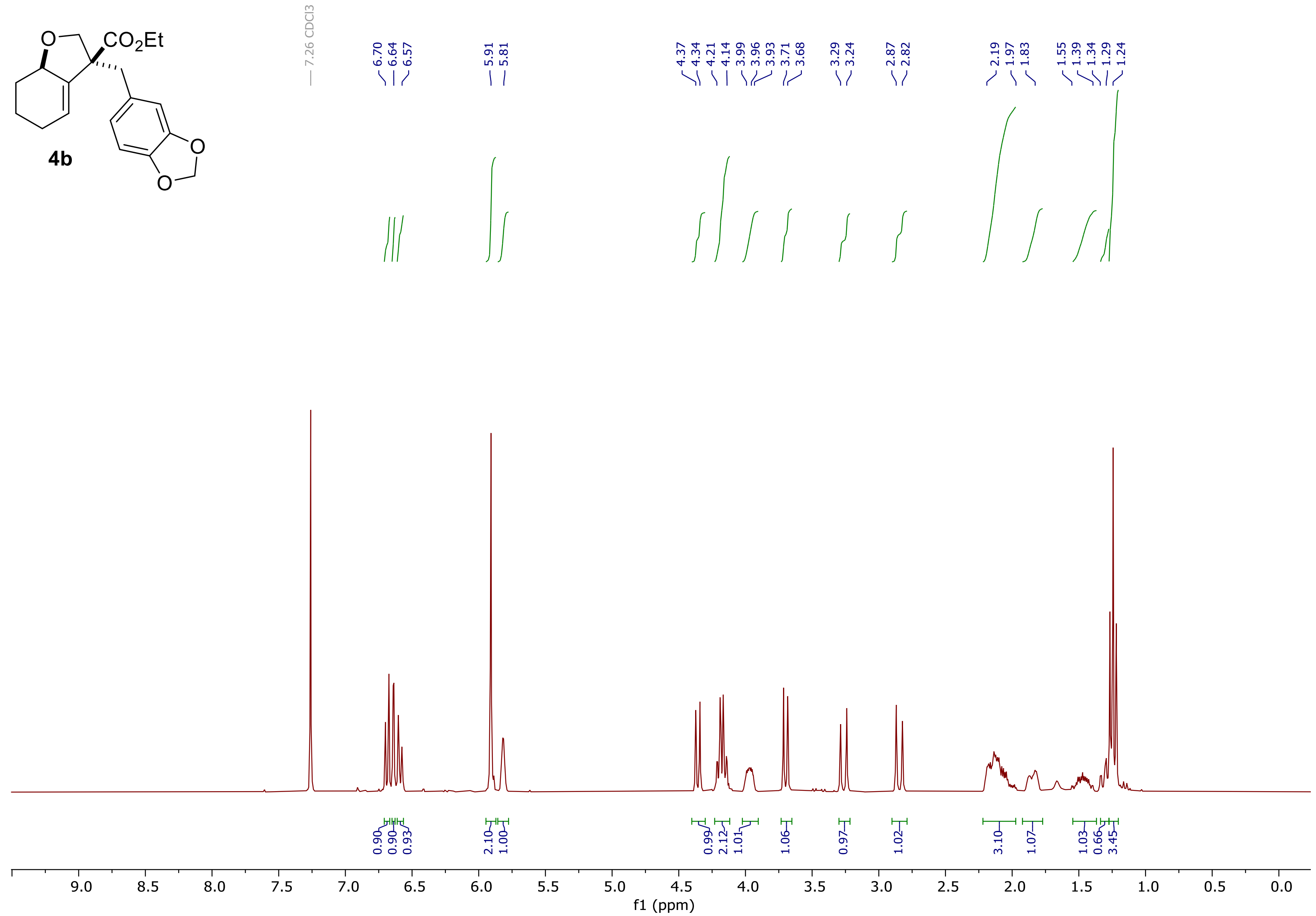

4b

^{1}H NMR spectrum (400 MHz, $CDCl_3$) of compound **4b**

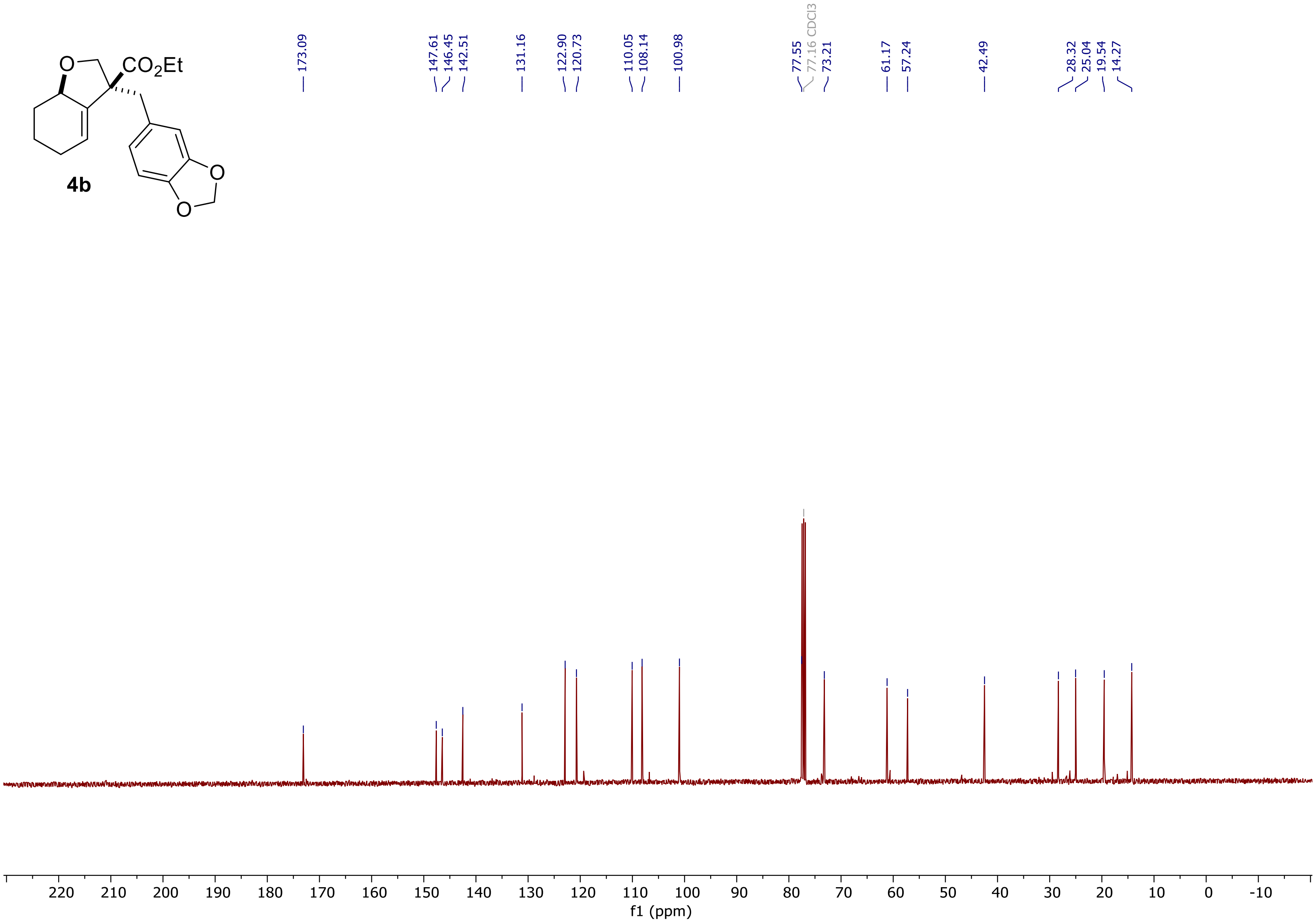

^{13}C NMR spectrum (101 MHz, $CDCl_3$) of compound **4b**

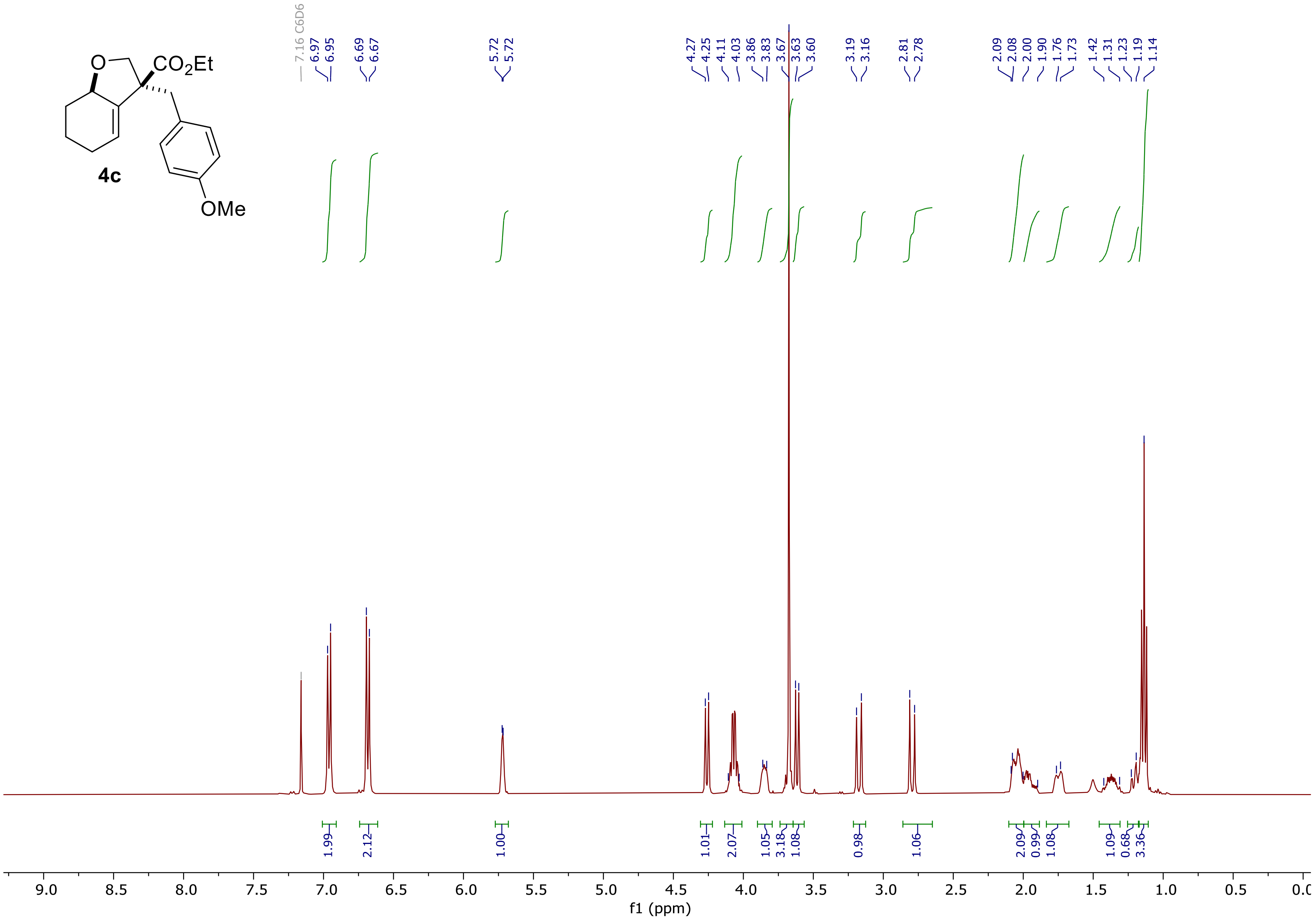

^{1}**H NMR** spectrum (400 MHz, $CDCl_3$) of compound **4c**

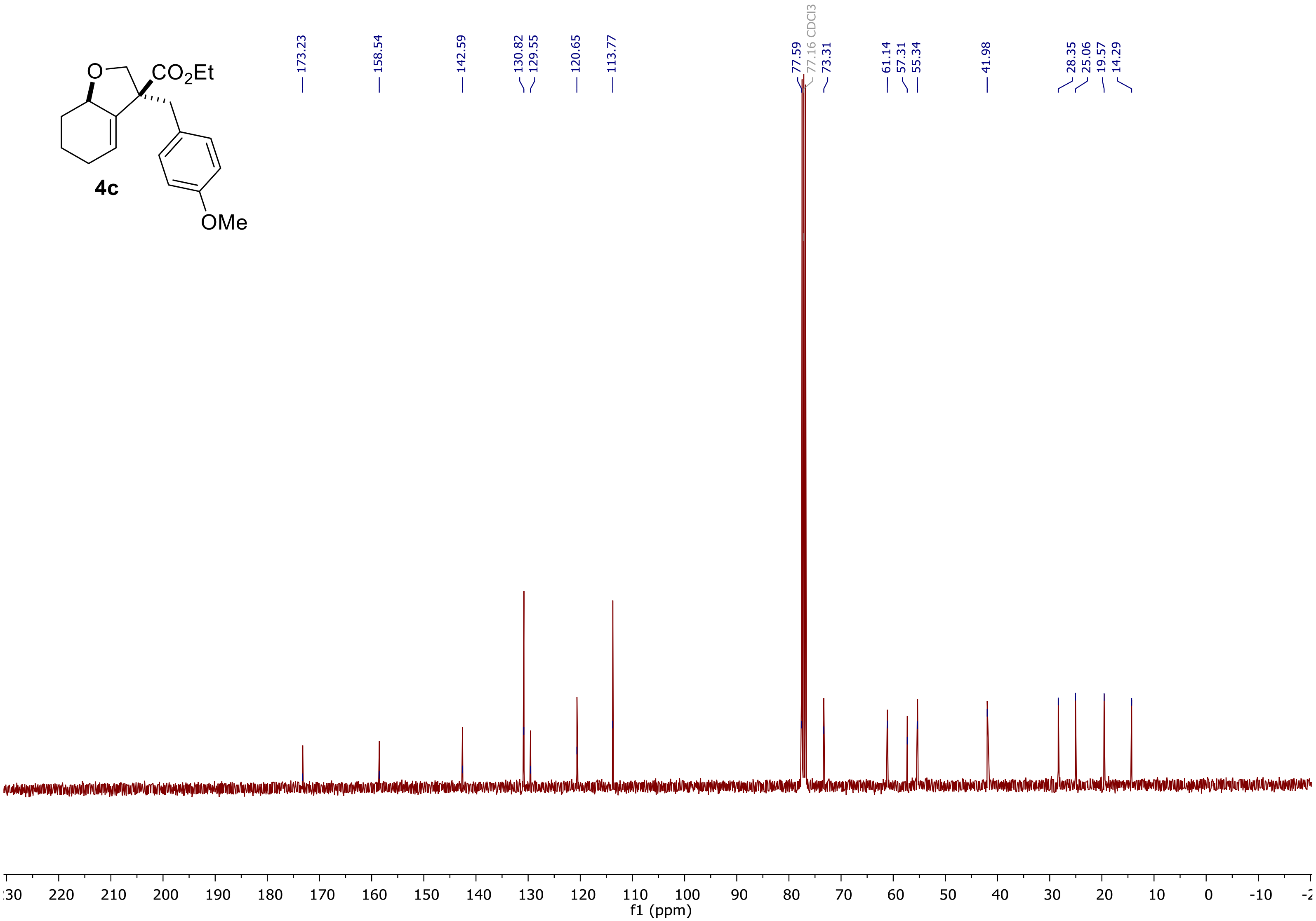

^{13}C NMR spectrum (101 MHz, $CDCl_3$) of compound **4c**

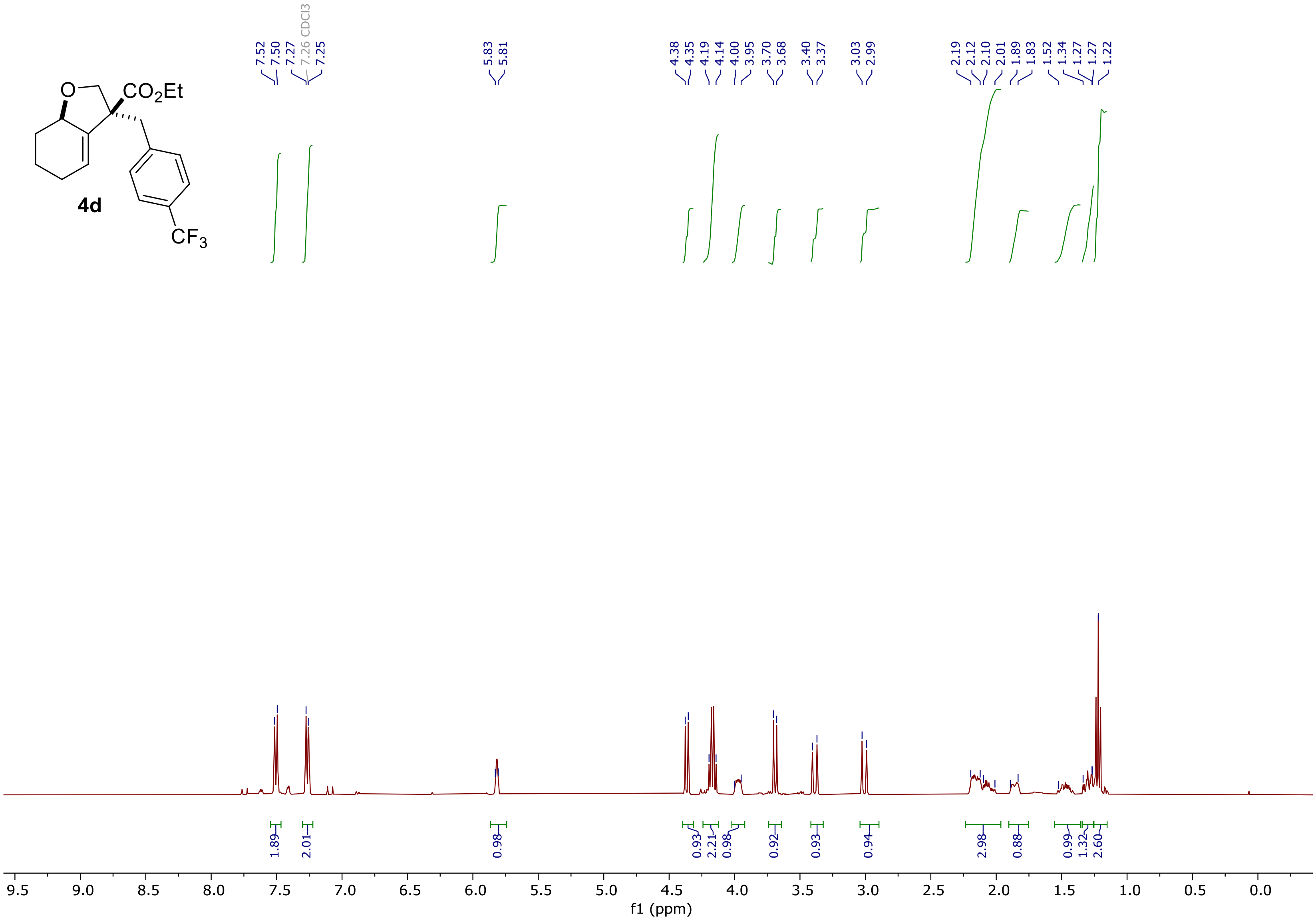

^{1}H NMR spectrum (400 MHz, $CDCl_3$) of compound **4d**

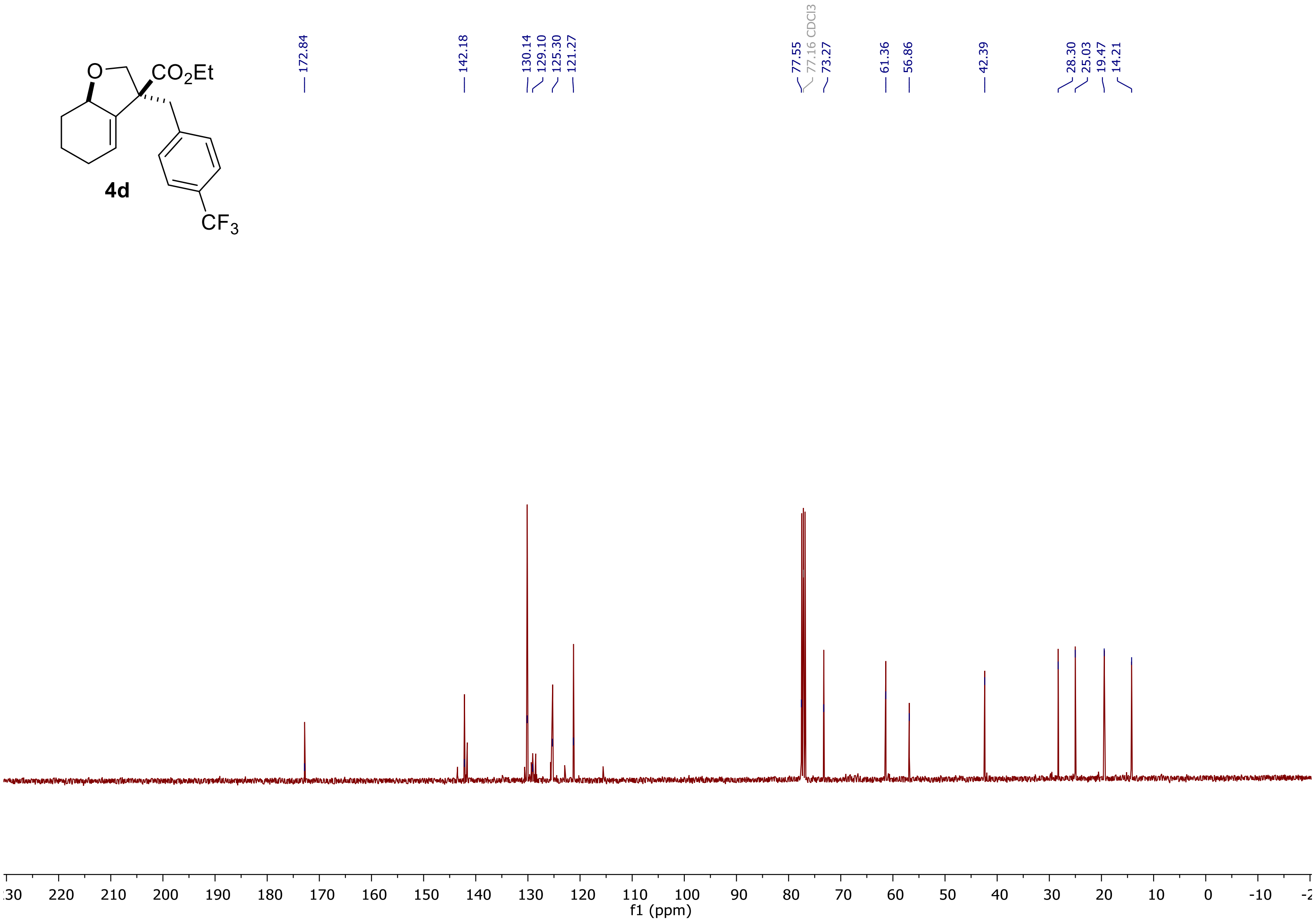

^{13}**C NMR** spectrum (101 MHz, $CDCl_3$) of compound **4d**

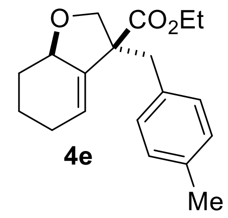

4e

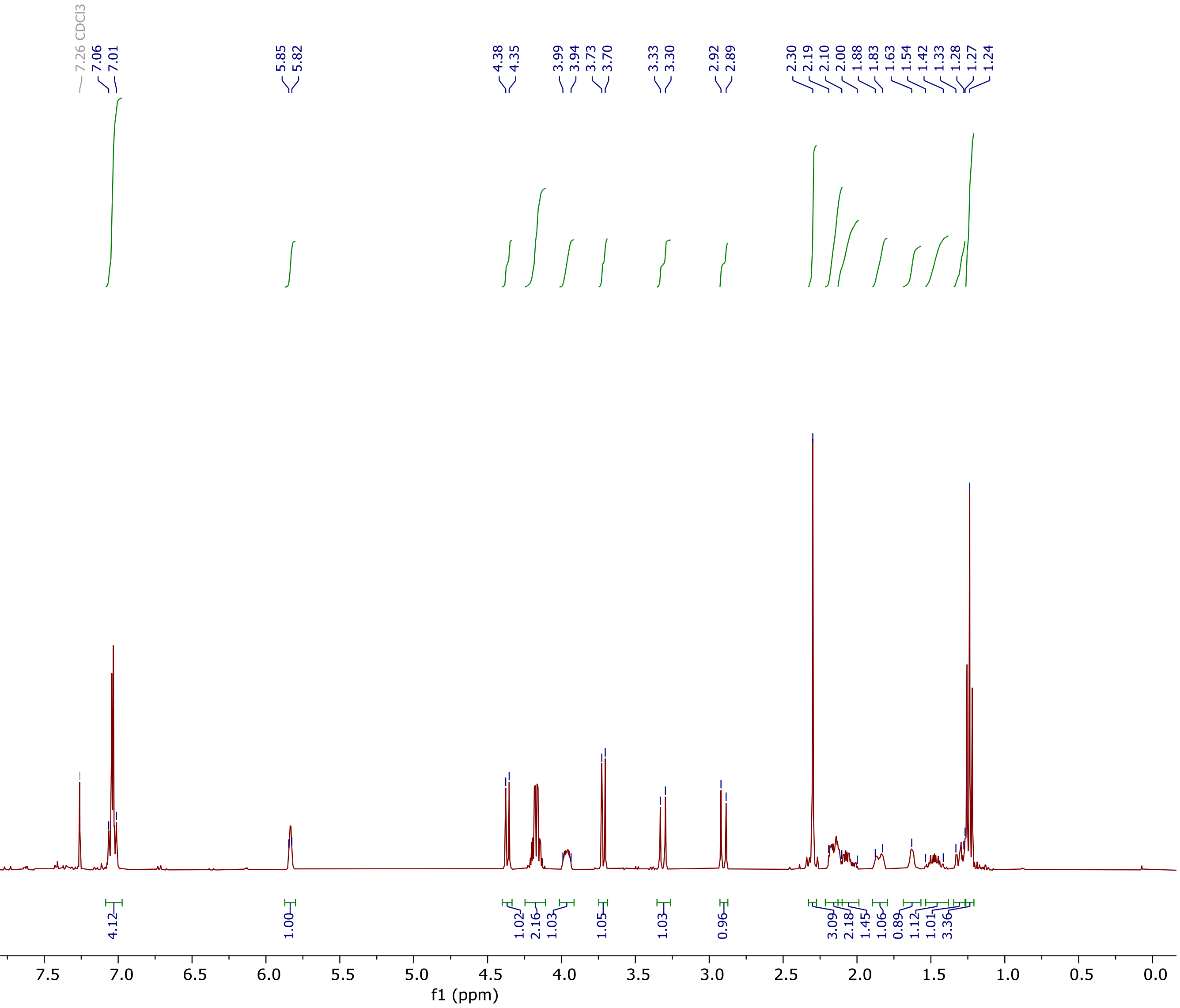

^{1}H NMR (400 MHz, $CDCl_3$) of compound **4e**

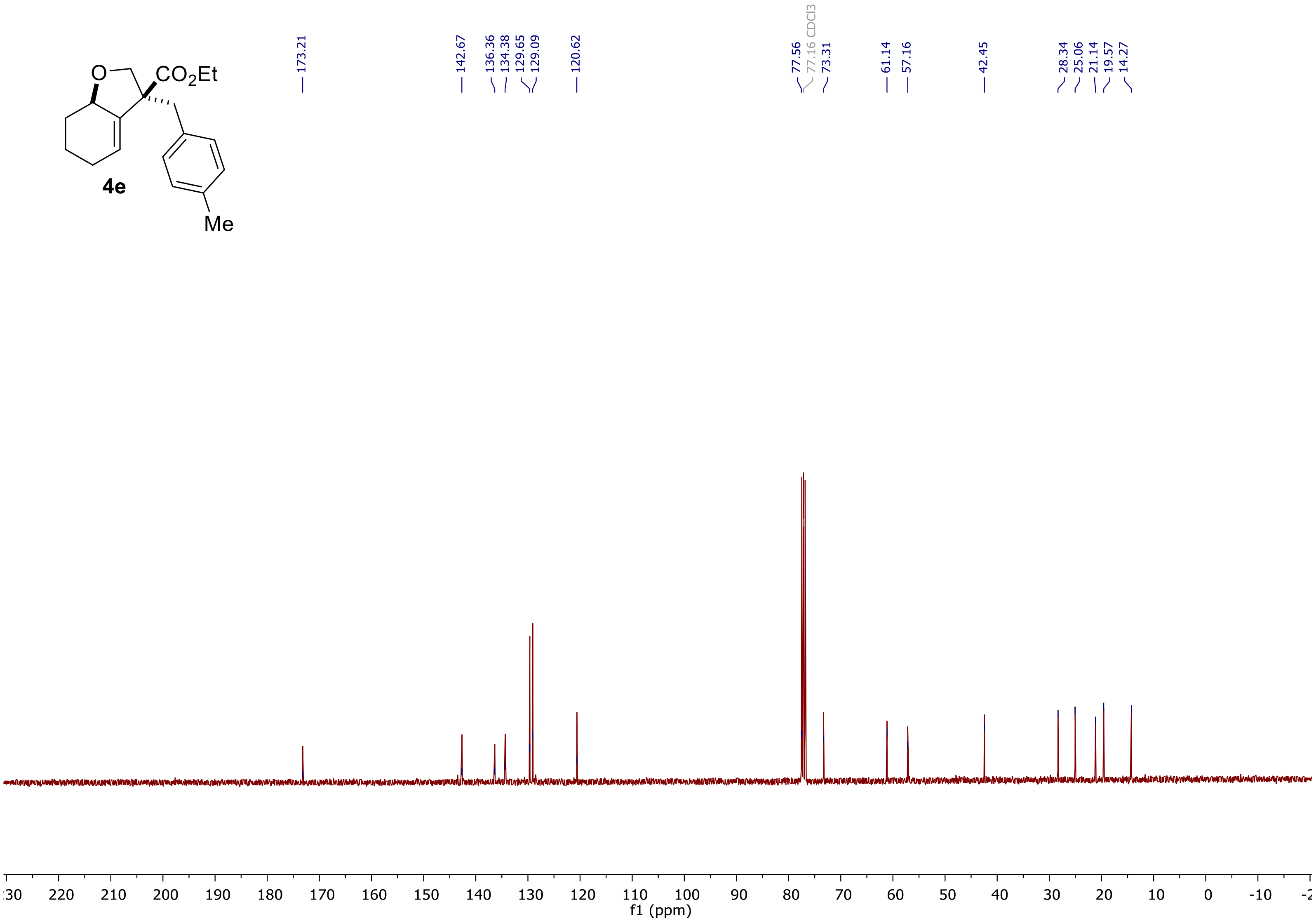

^{13}C NMR spectrum (101 MHz, $CDCl_3$) of compound **4e**

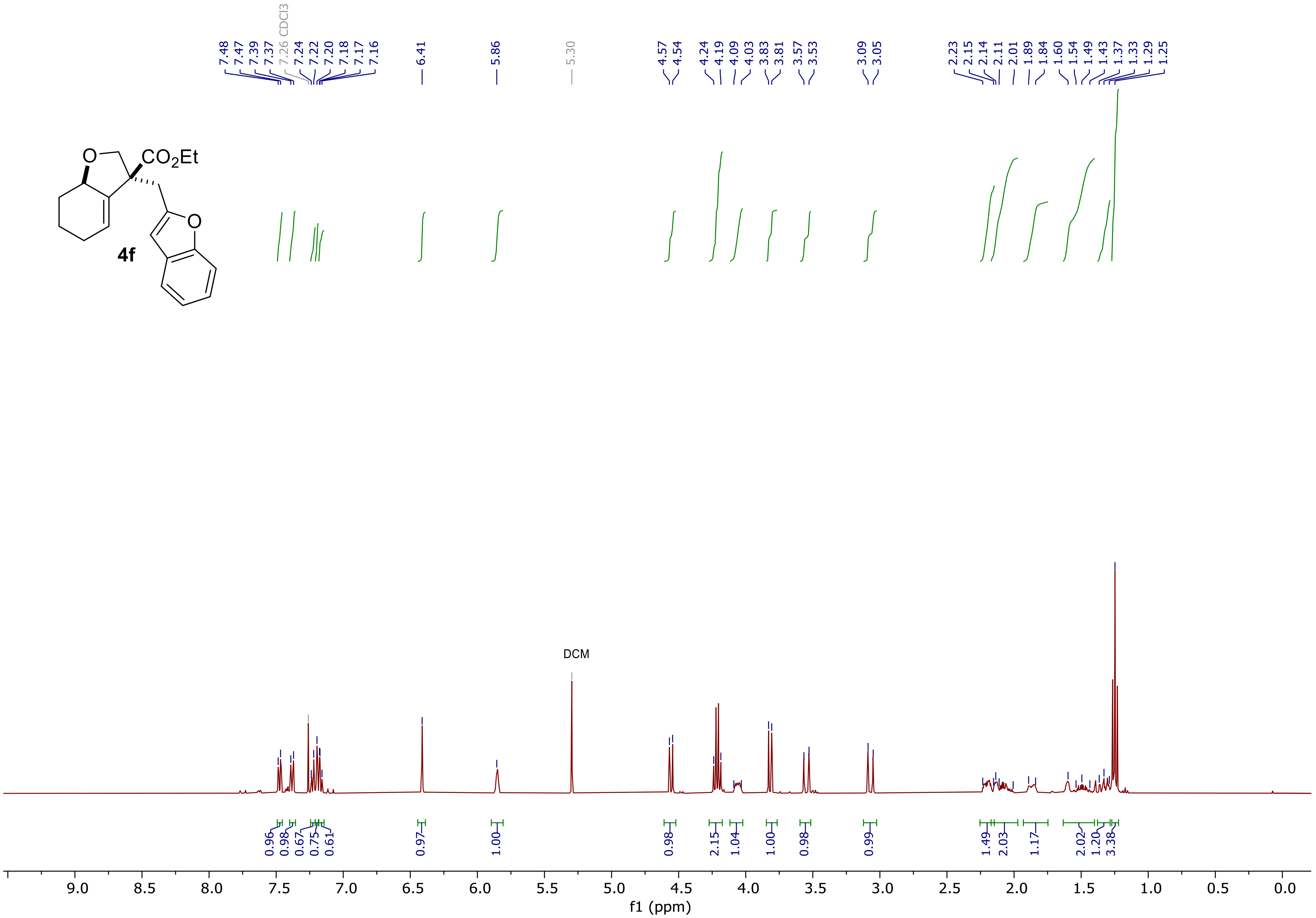

[1]**H NMR** spectrum (400 MHz, $CDCl_3$) of compound **4f**

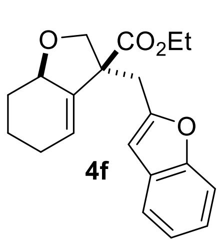

4f

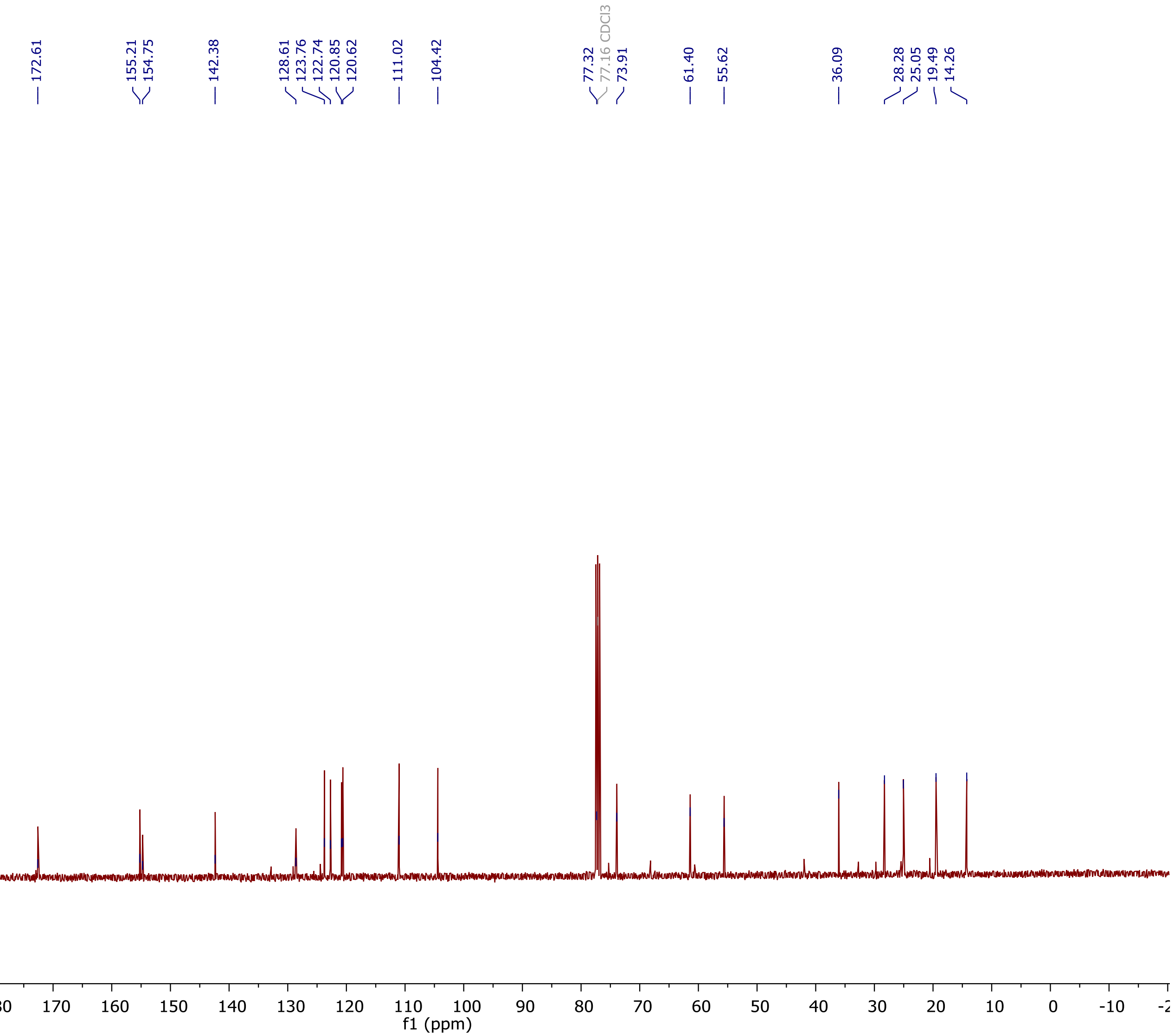

^{13}C NMR spectrum (101 MHz, $CDCl_3$) of compound **4f**

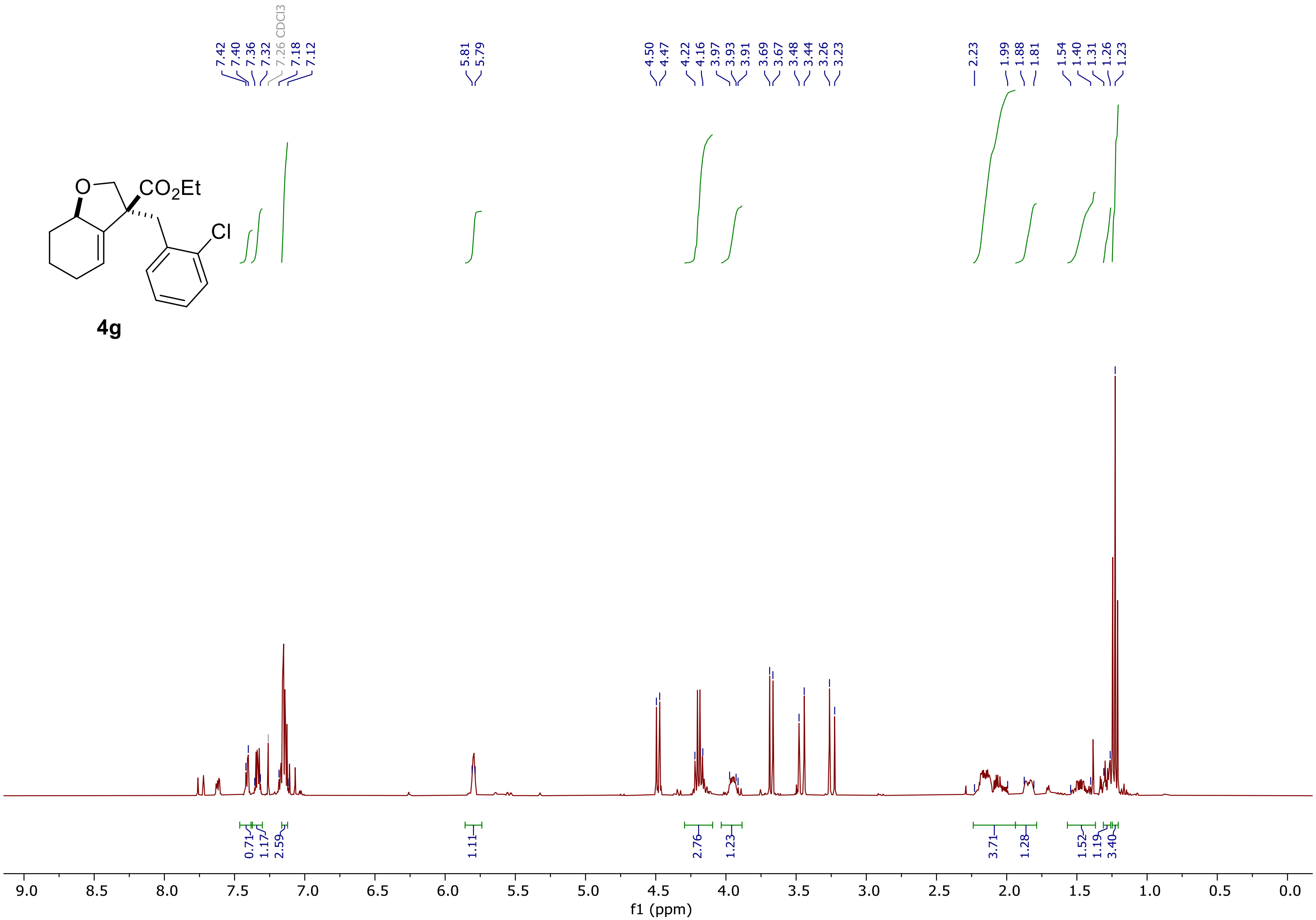

^{1}**H NMR** spectrum (400 MHz, $CDCl_3$) of compound **4g**

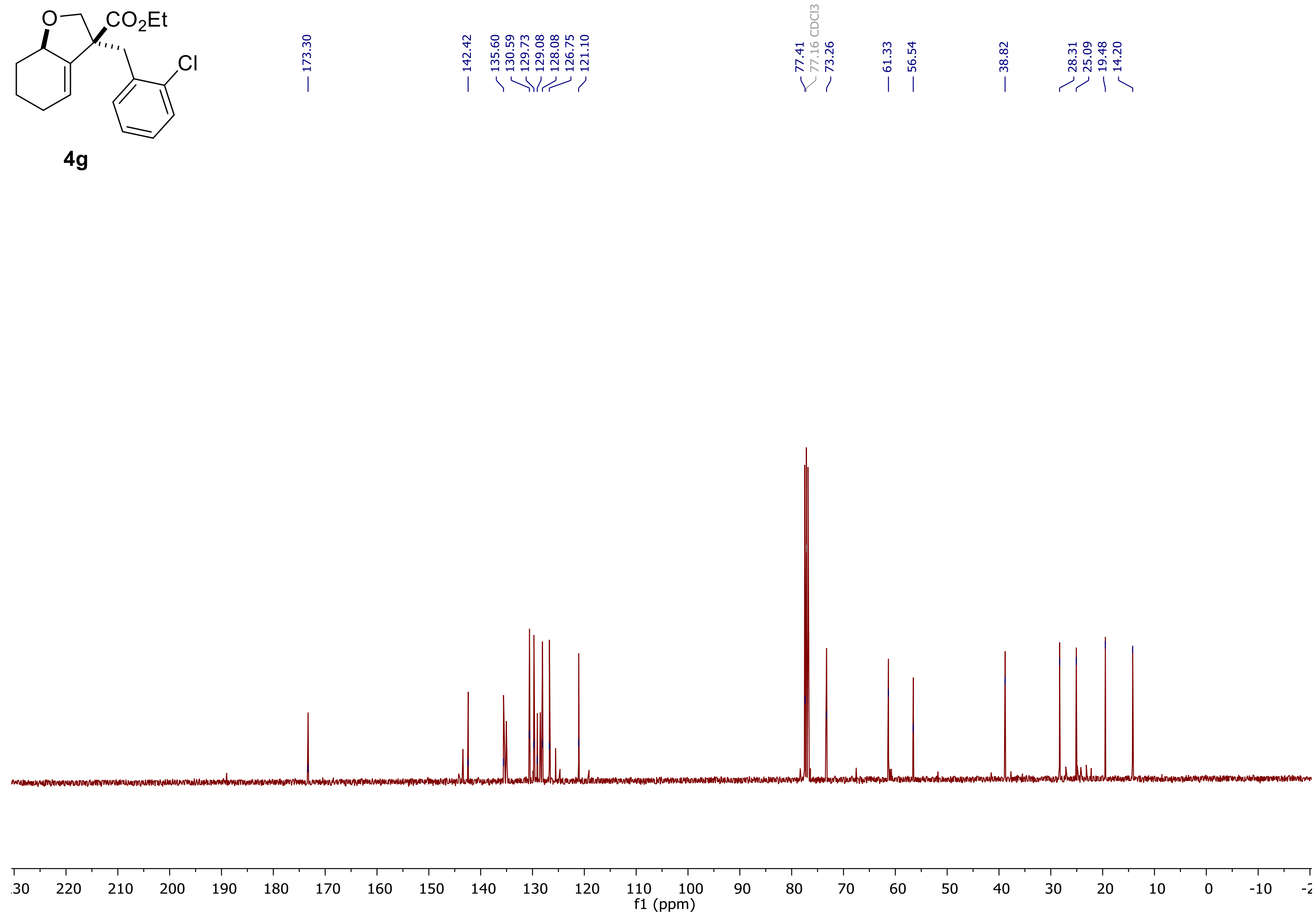

^{13}C NMR spectrum (101 MHz, $CDCl_3$) of compound **4g**

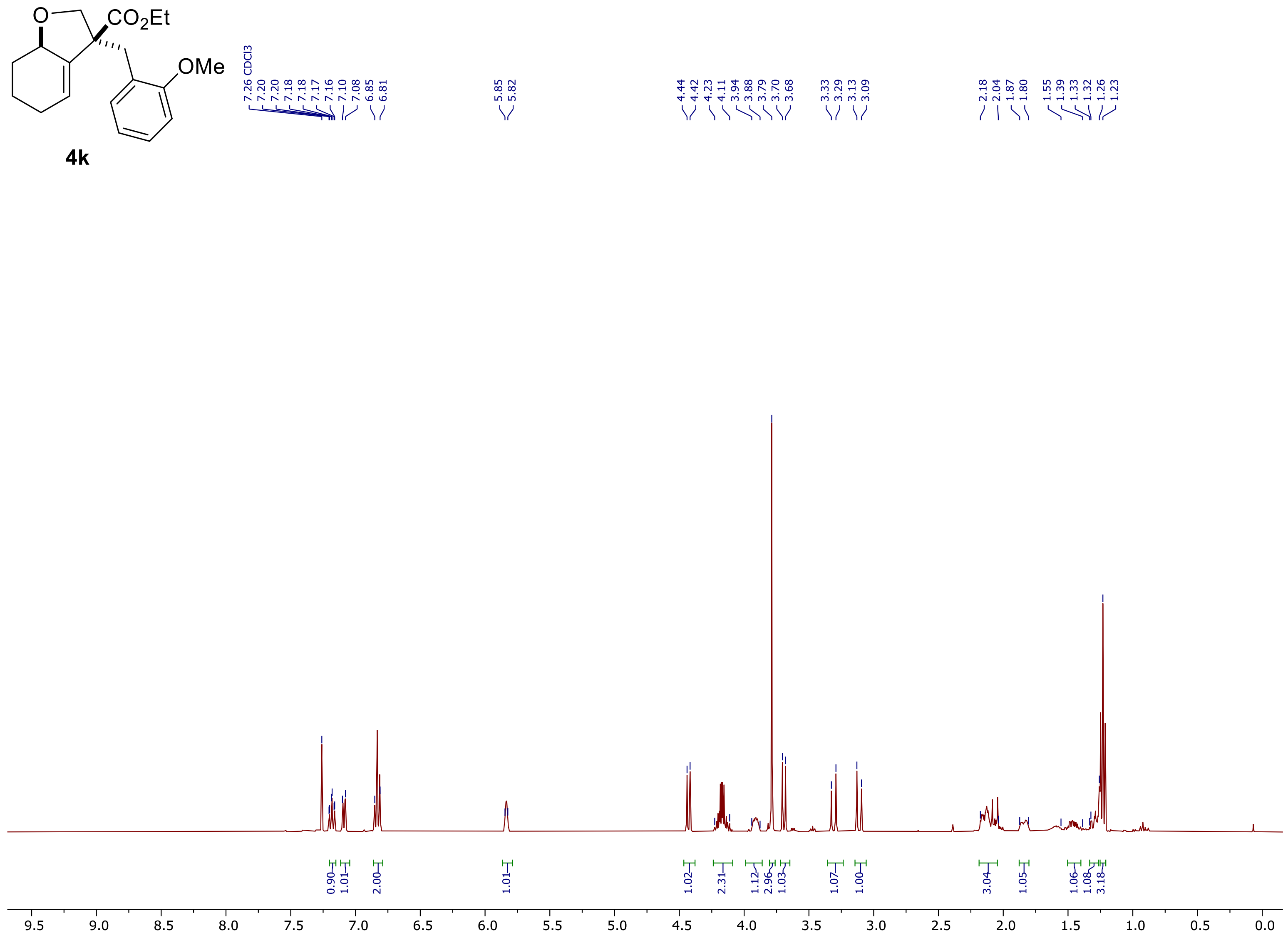

^{1}H NMR spectrum (400 MHz, $CDCl_3$) of compound **4k**

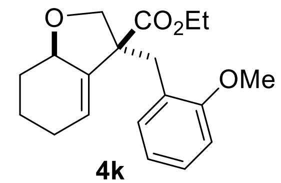

4k

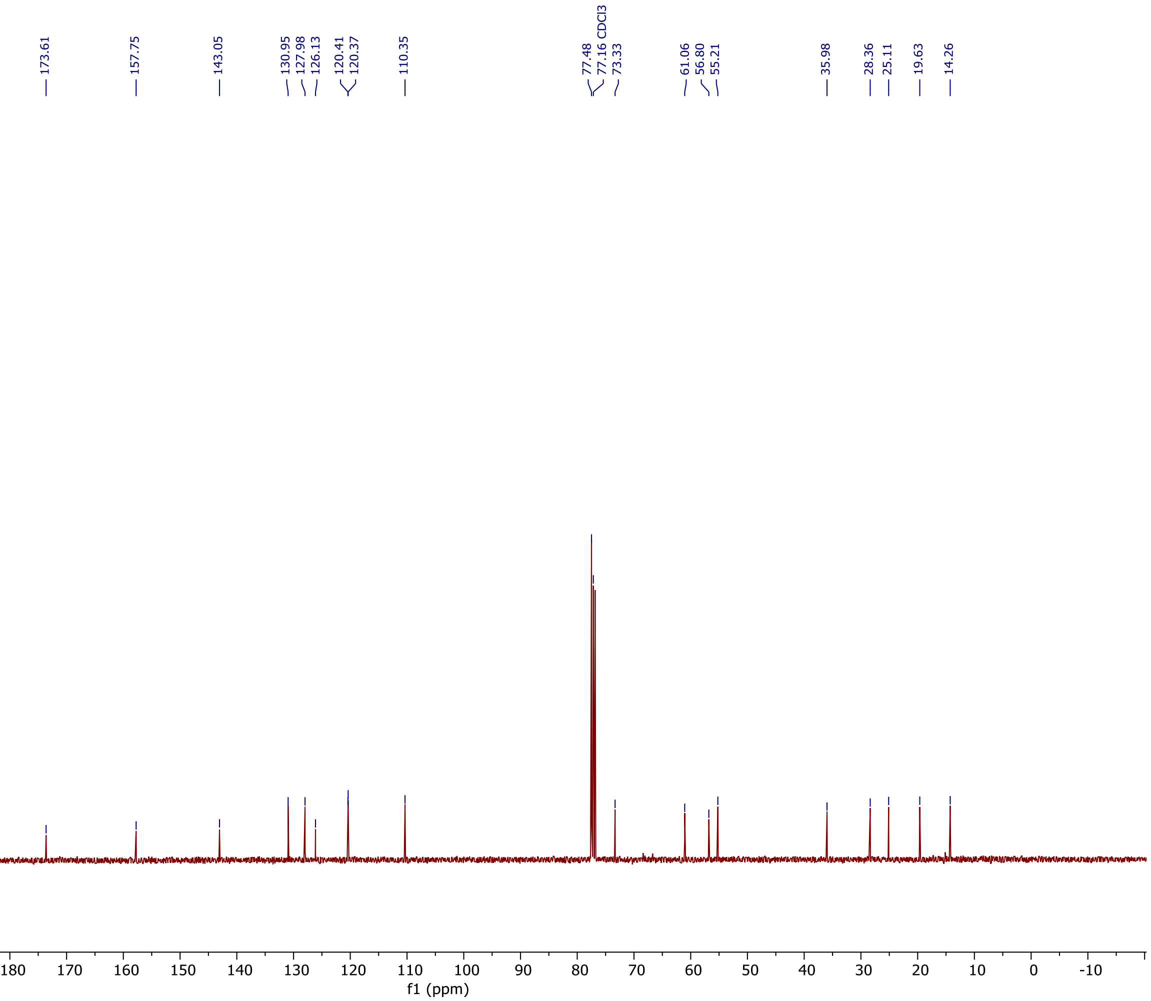

^{13}C NMR spectrum (101 MHz, $CDCl_3$) of compound **4k**

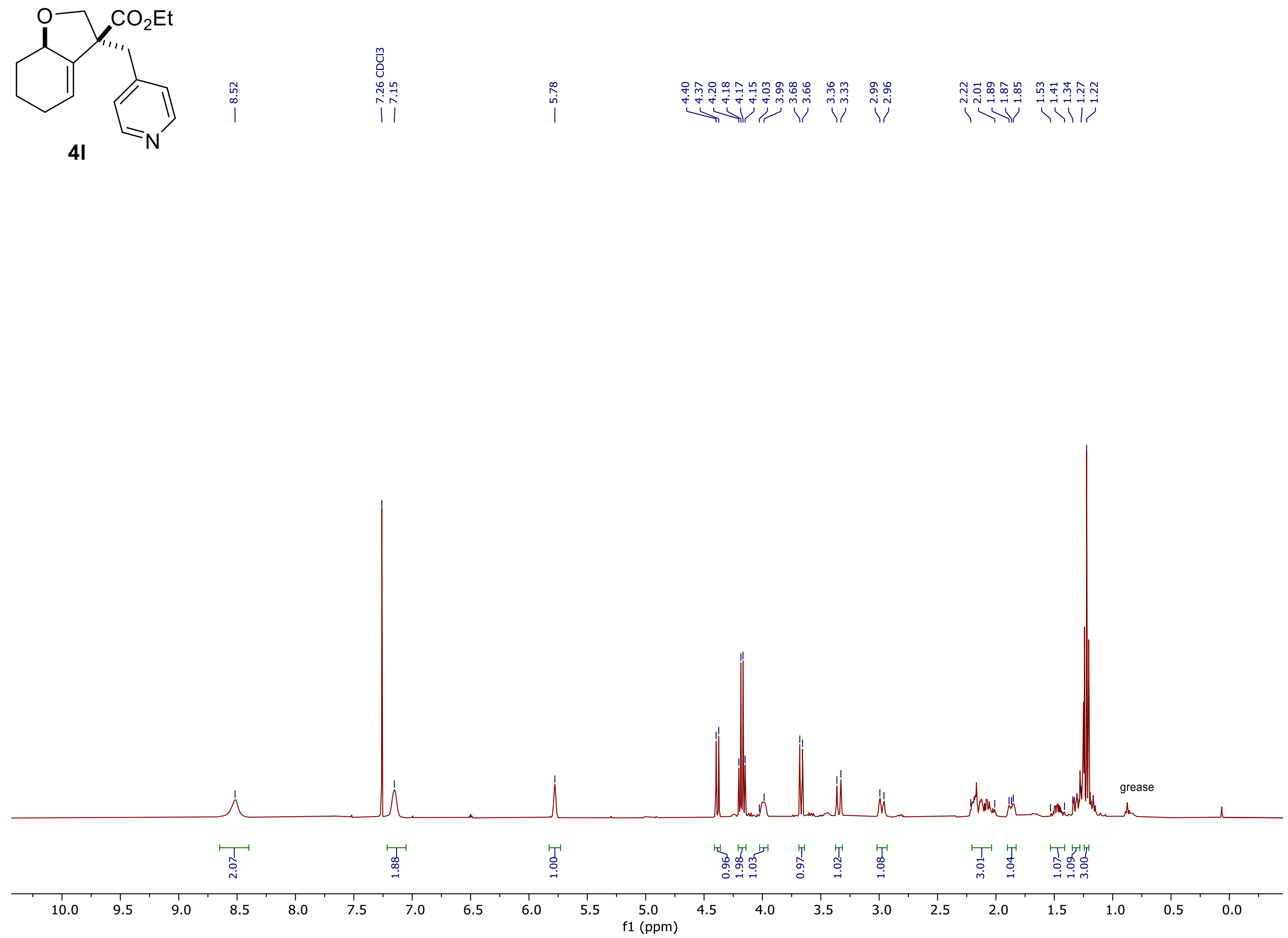

^{1}H NMR spectrum (400 MHz, $CDCl_3$) of compound **4l**

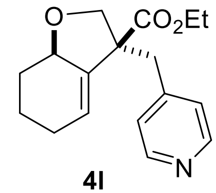

4l

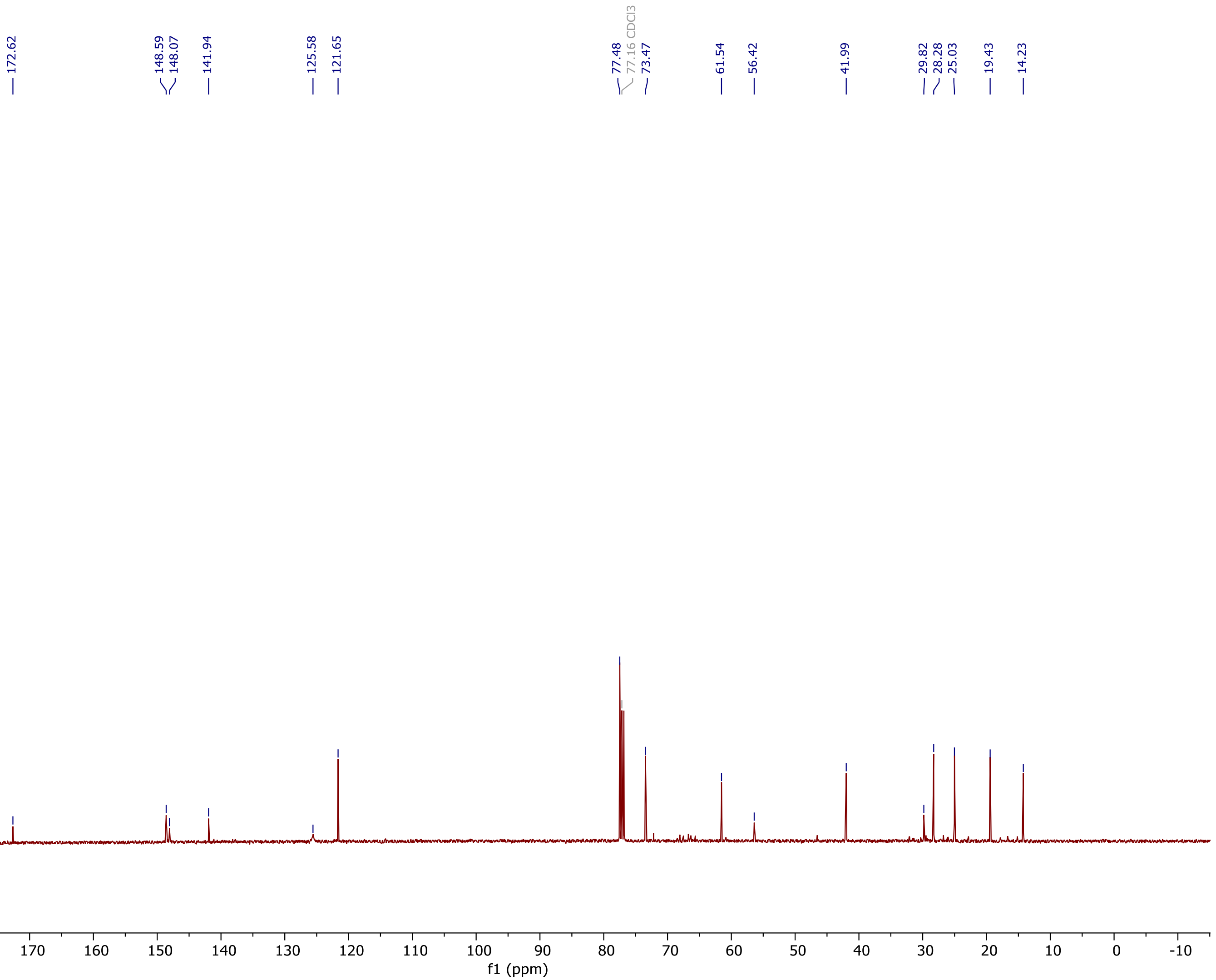

^{13}C NMR spectrum (101 MHz, $CDCl_3$) of compound **4l**

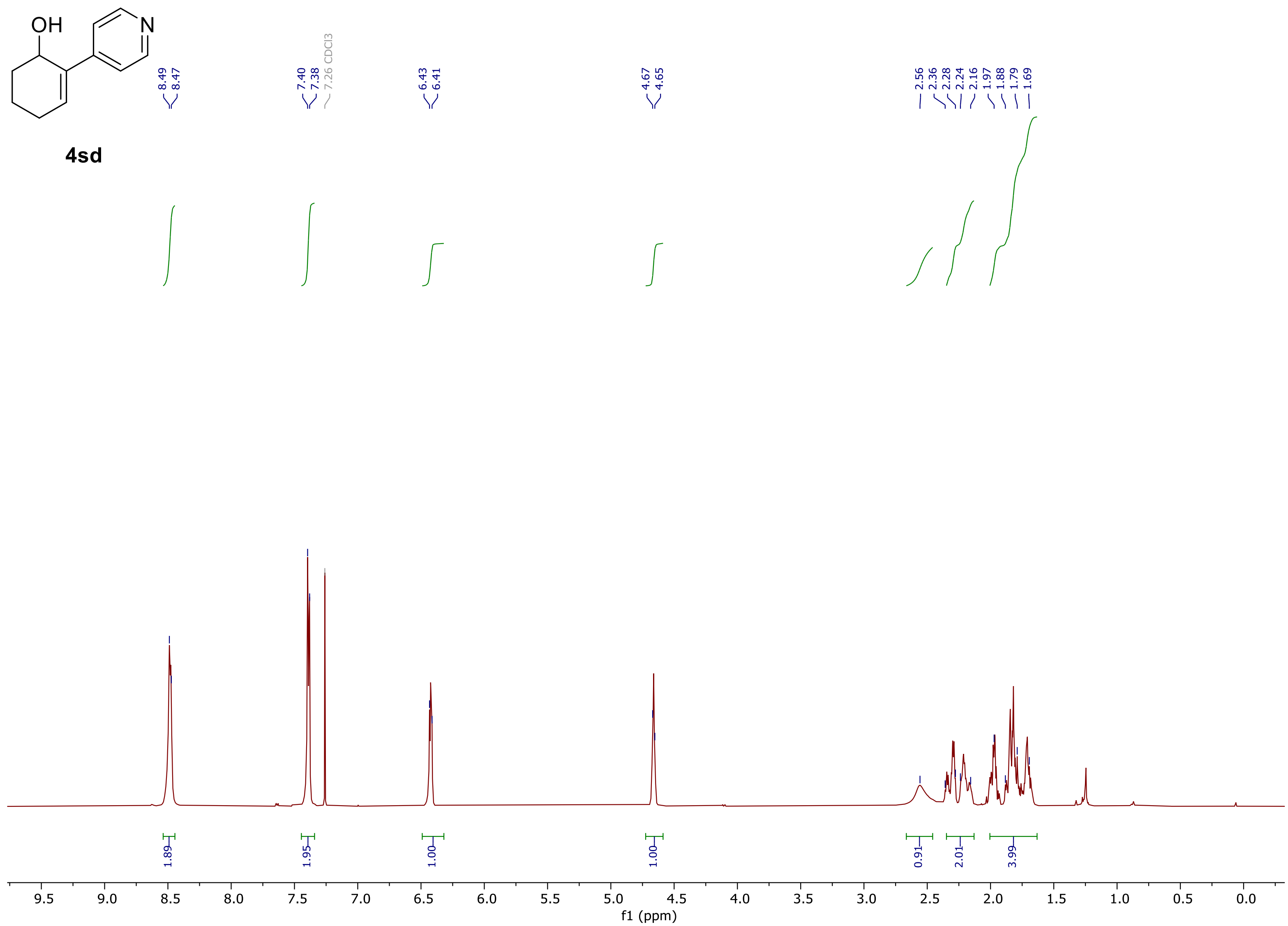

^{1}H NMR spectrum (400 MHz, $CDCl_3$) of compound **4sl**

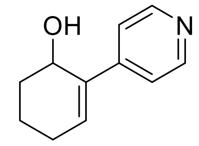

4sd

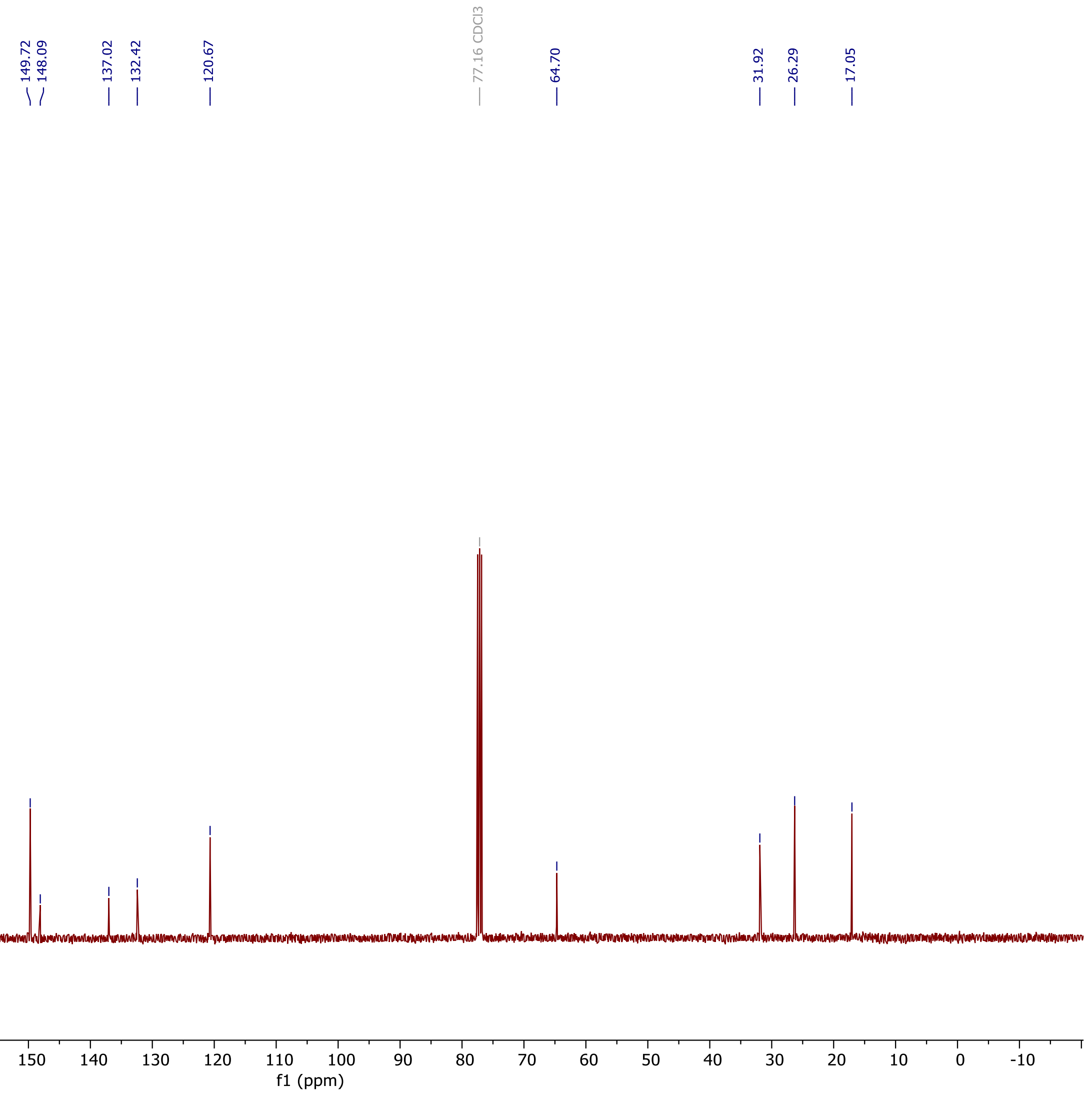

^{13}**C NMR** spectrum (101 MHz, $CDCl_3$) of compound **4sl**

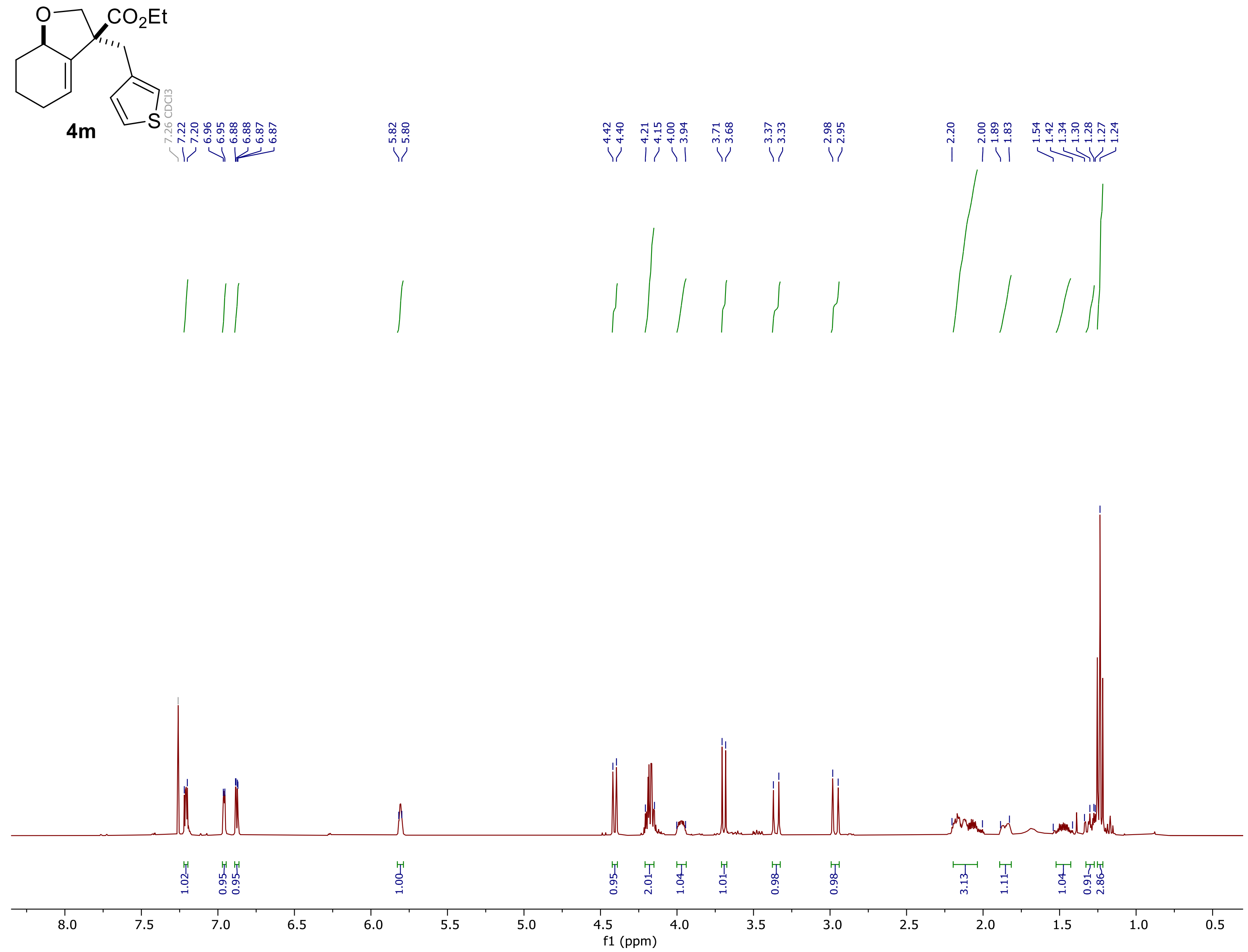

¹H NMR spectrum (400 MHz, $CDCl_3$) of compound **4m**

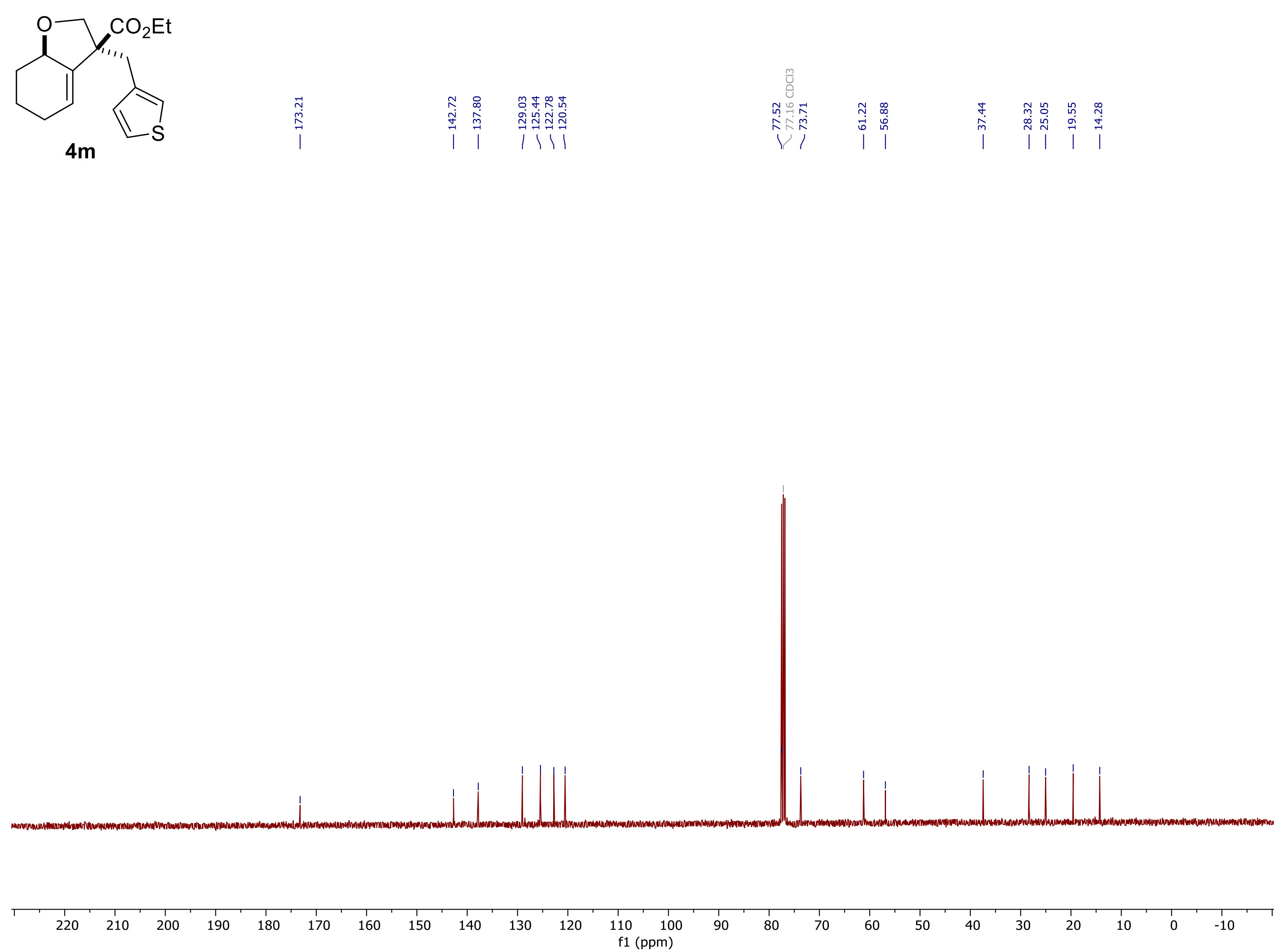

^{13}C NMR spectrum (101 MHz, $CDCl_3$) of compound **4m**

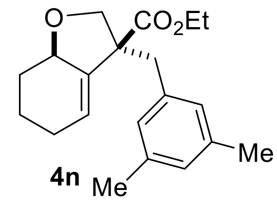

4n

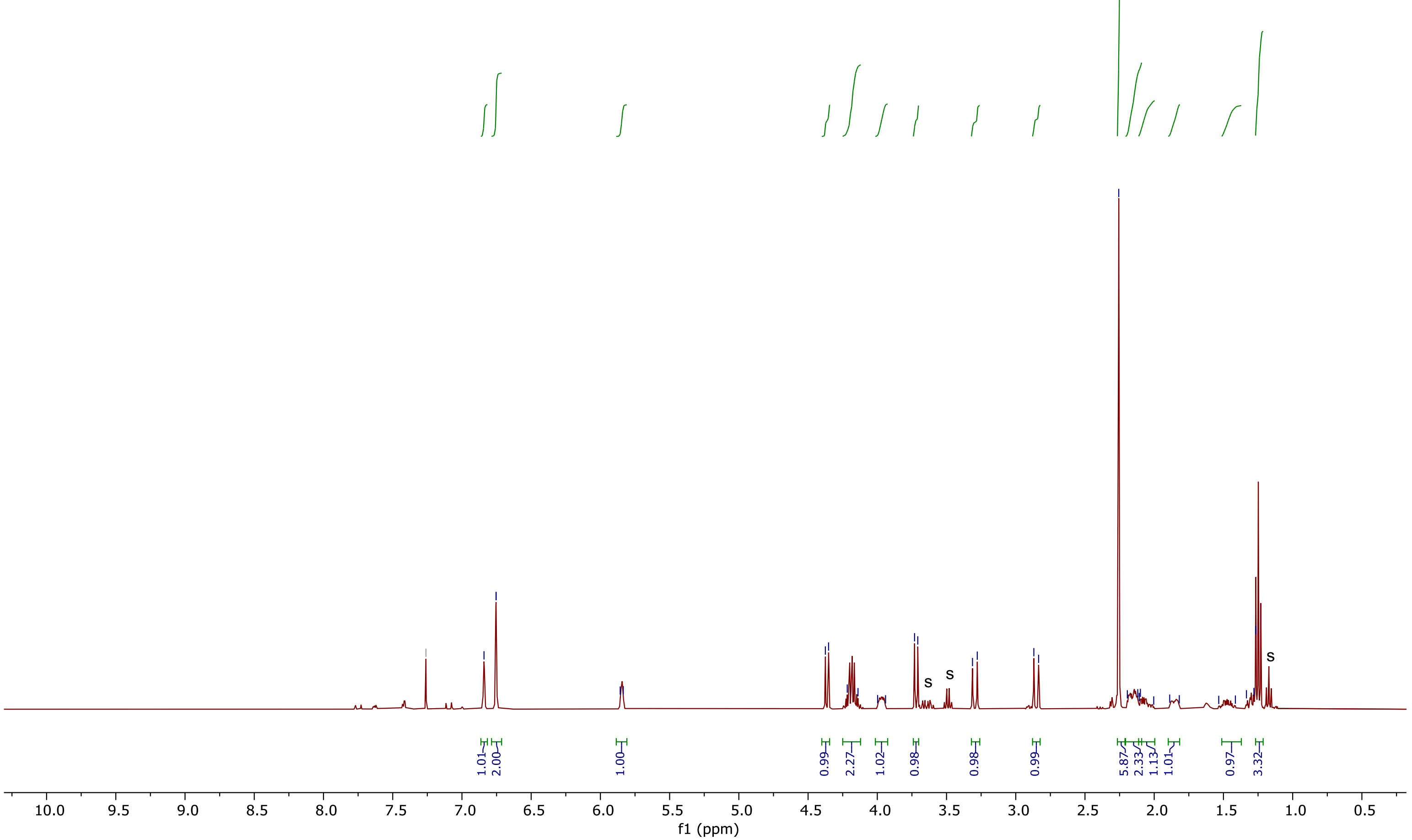

[1]**H NMR** spectrum (400 MHz, $CDCl_3$) of compound **4n** (s: signals of impurities)

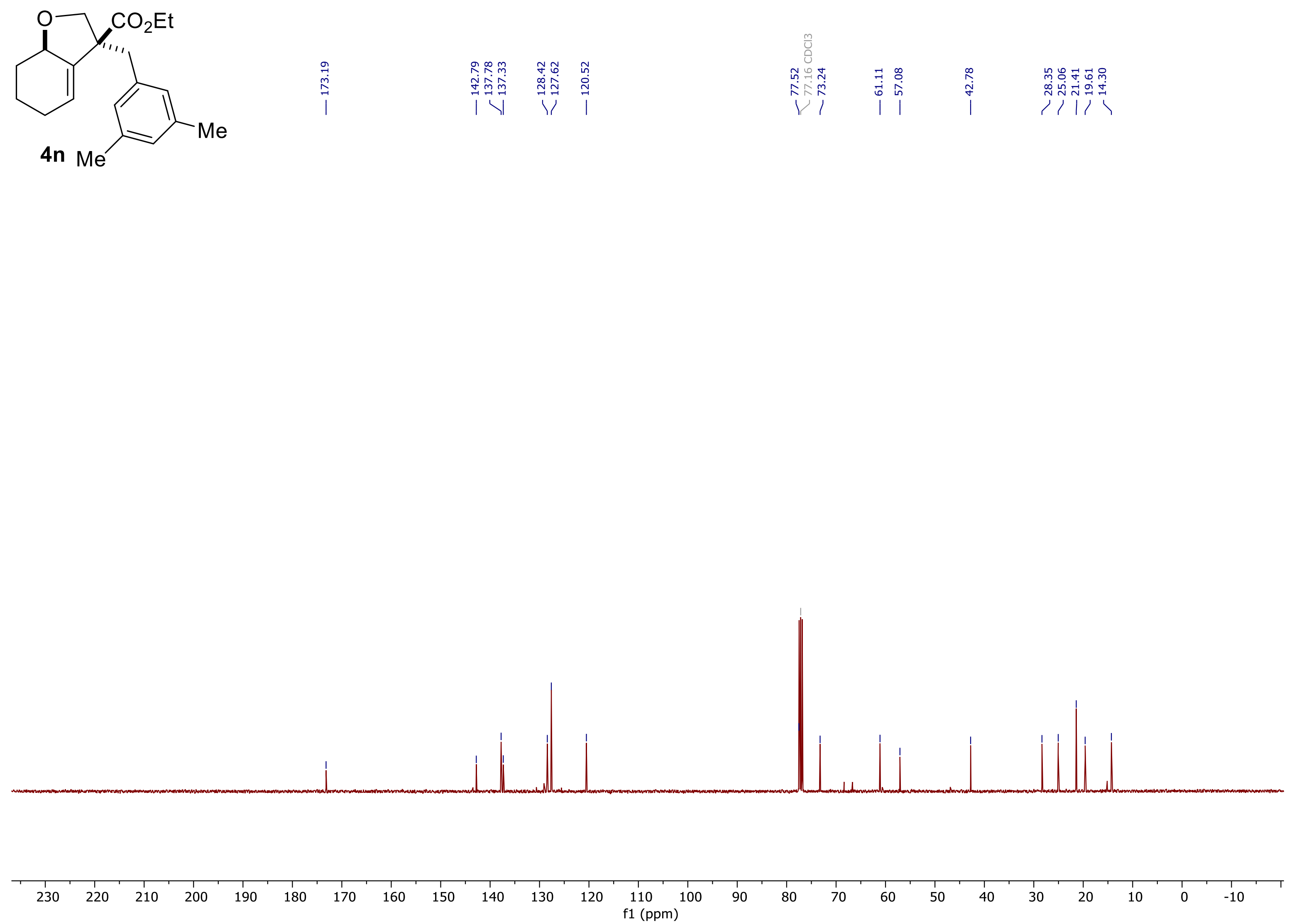

^{13}**C NMR** spectrum (101 MHz, $CDCl_3$) of compound **4n**

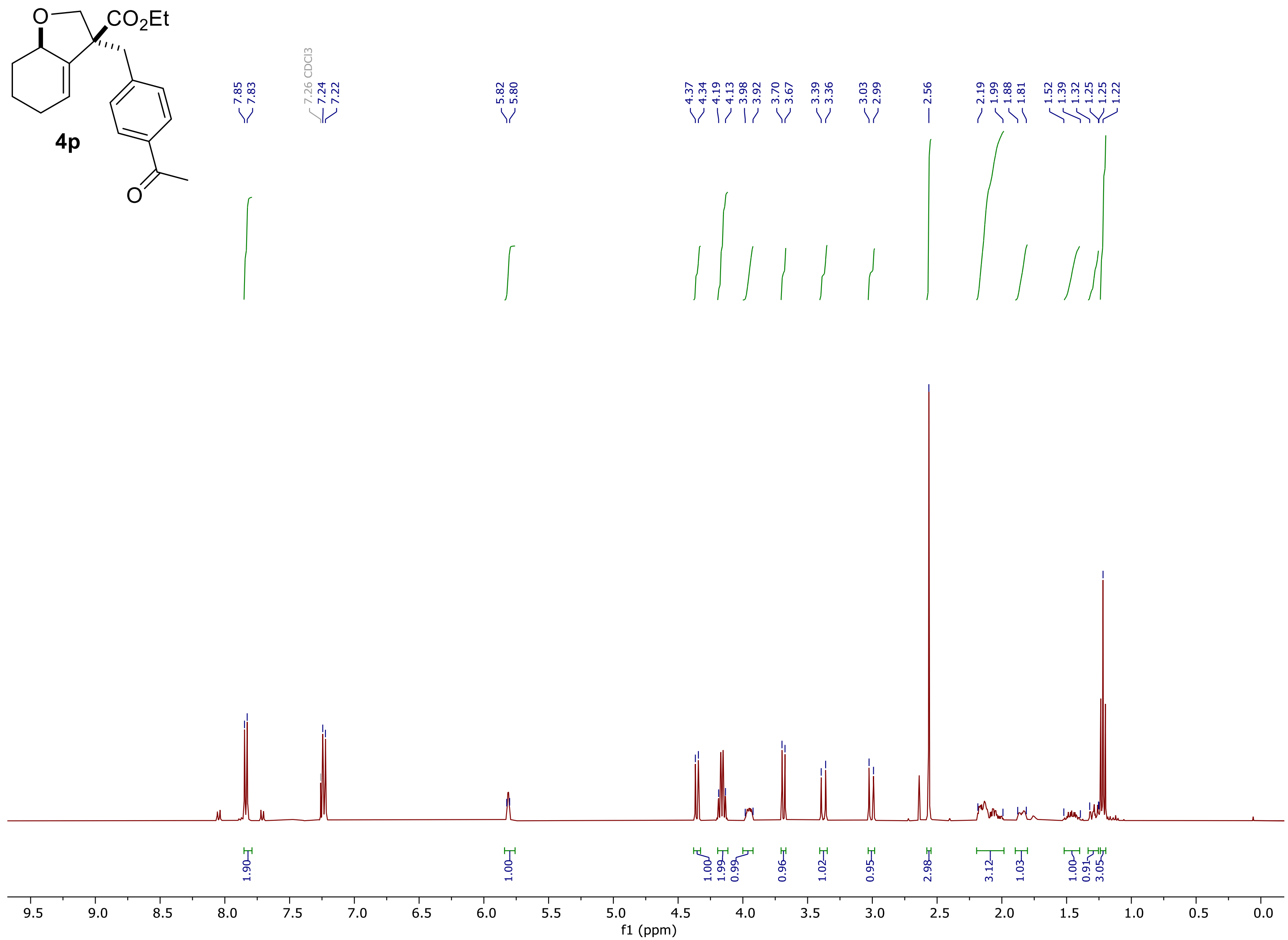

^{1}H NMR spectrum (400 MHz, $CDCl_3$) of compound **4p**

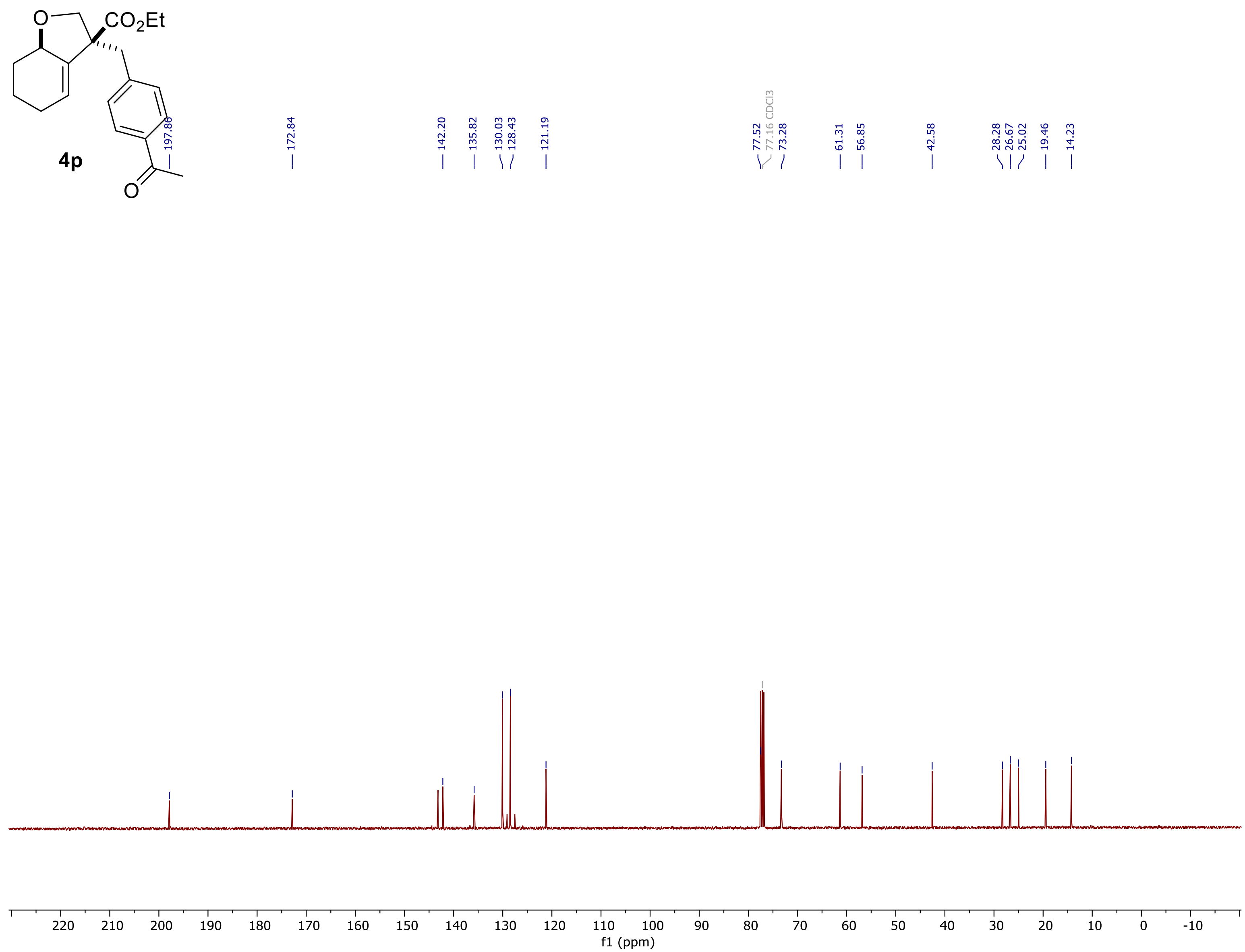

^{13}C NMR spectrum (101 MHz, $CDCl_3$) of compound **4p**

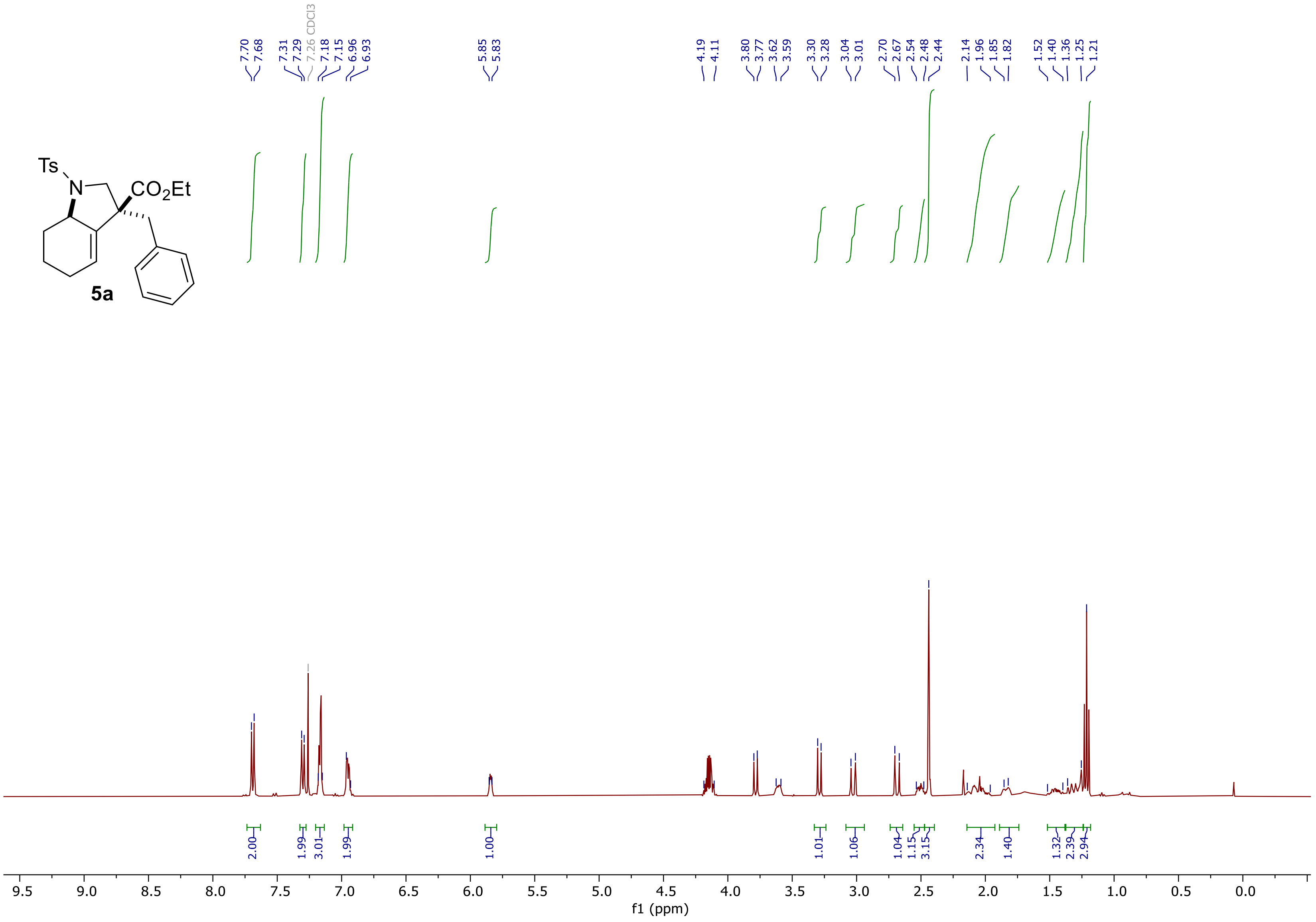

^{1}H NMR spectrum (400 MHz, $CDCl_3$) of compound **5a**

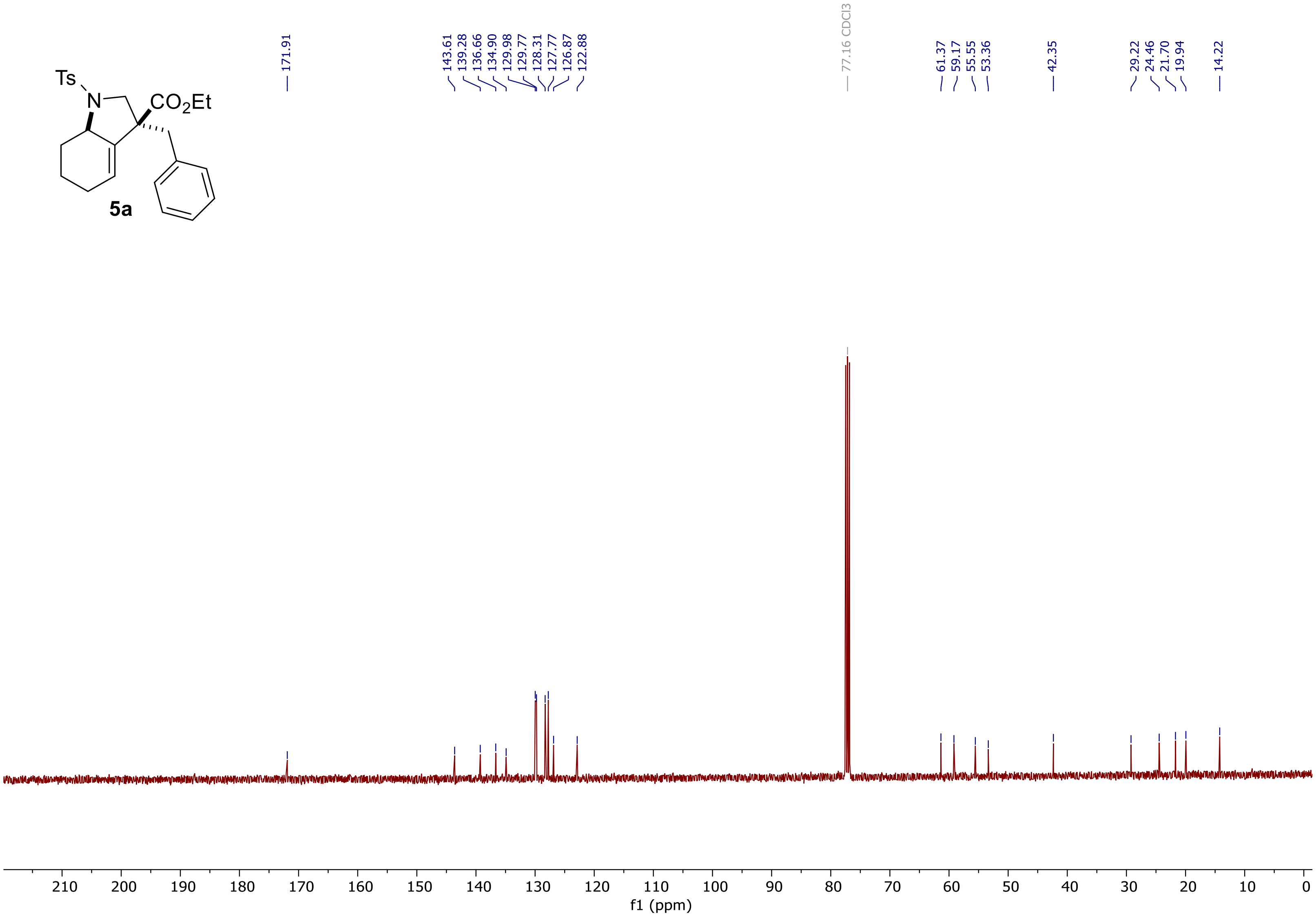

^{13}C NMR spectrum (101 MHz, $CDCl_3$) of compound **5a**

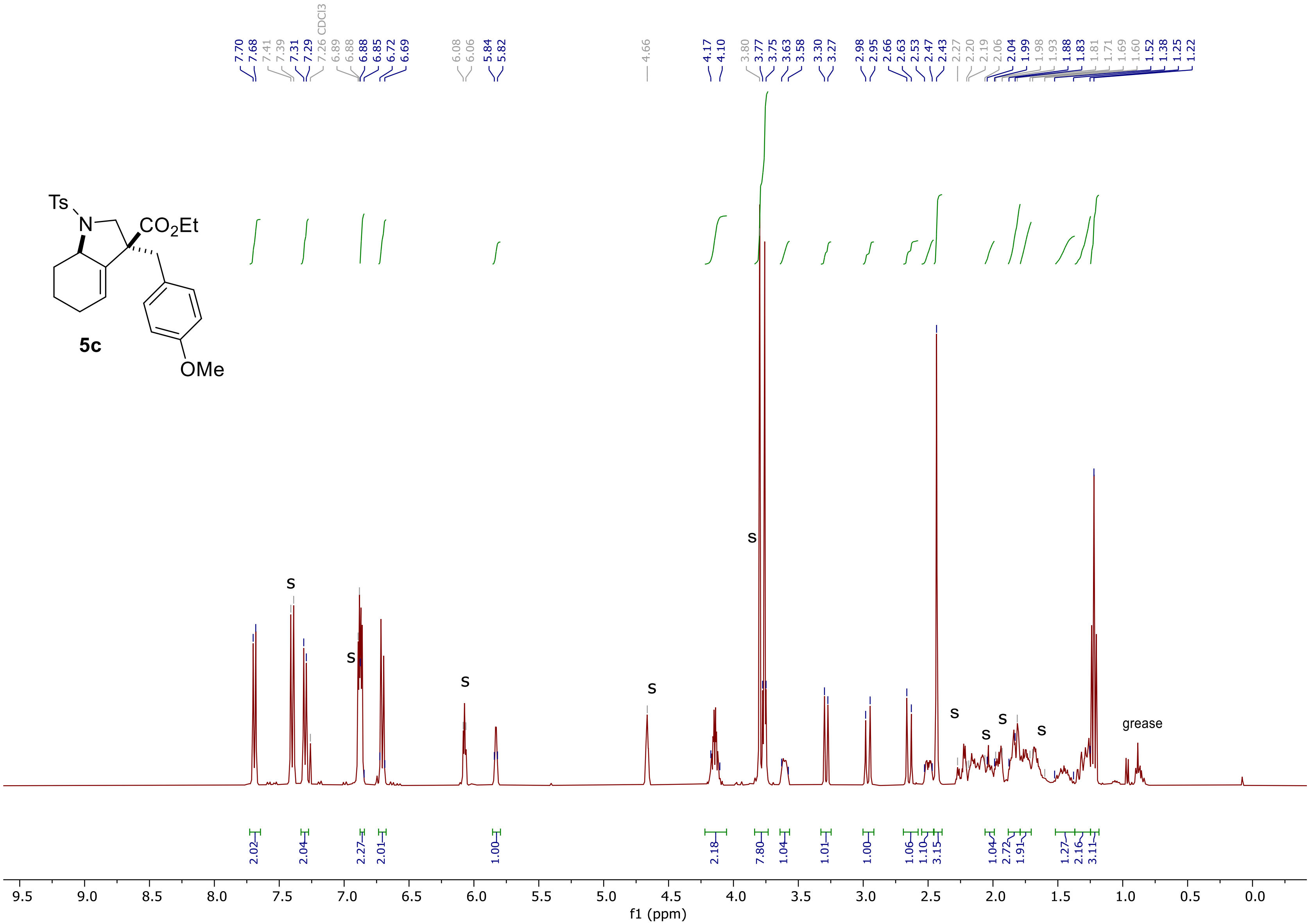

¹H NMR spectrum (400 MHz, $CDCl_3$) of compound **5c** (s = signals of side product 2-(4-methoxy)cyclohex-2-en-1-ol[5])

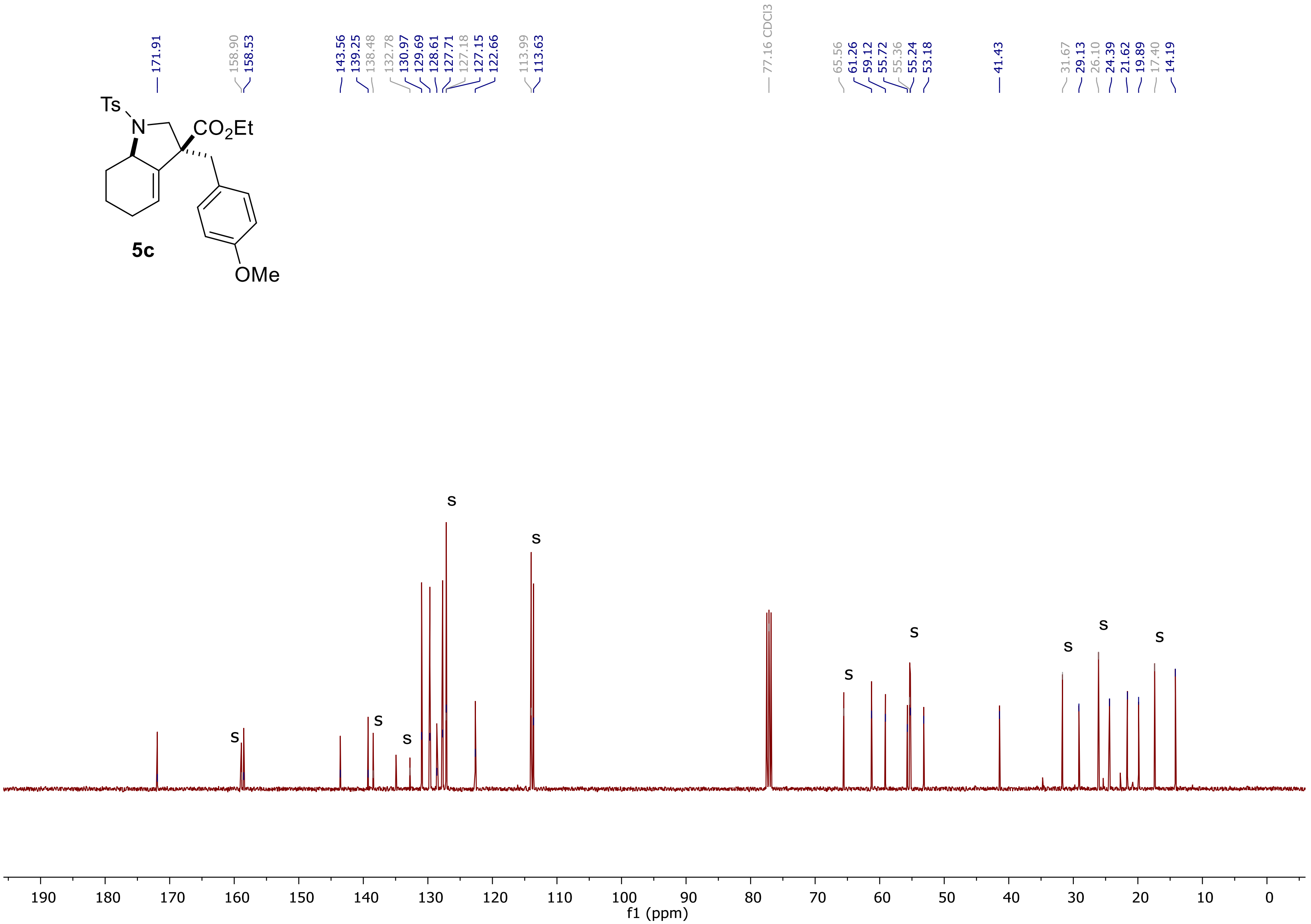

^{13}**C NMR** spectrum (101 MHz, $CDCl_3$) of compound **5c** (s = signals of side product 2-(4-methoxy)cyclohex-2-en-1-ol[5])

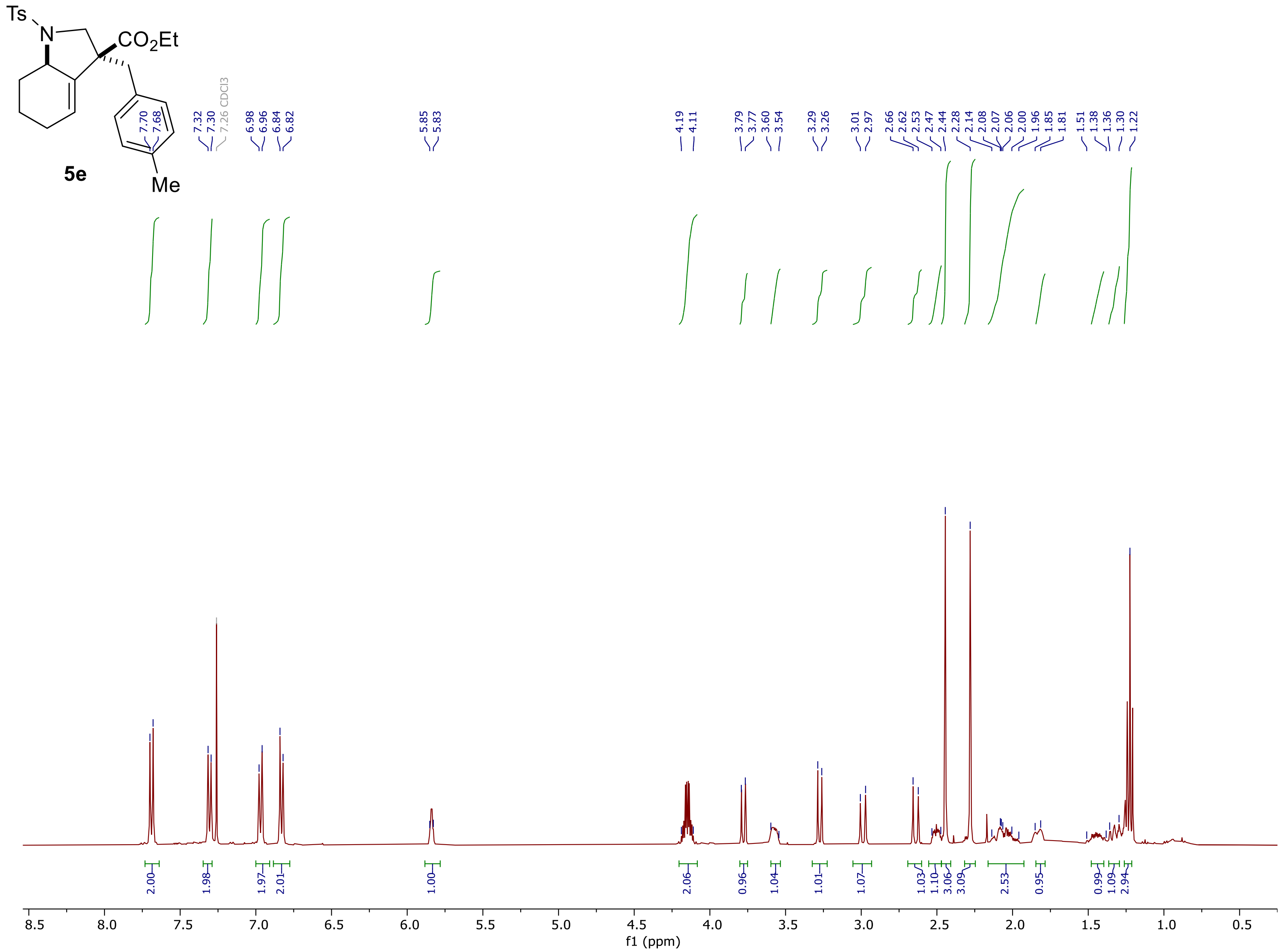

[1]**H NMR** spectrum (400 MHz, $CDCl_3$) of compound **5e**

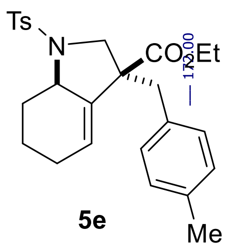

5e

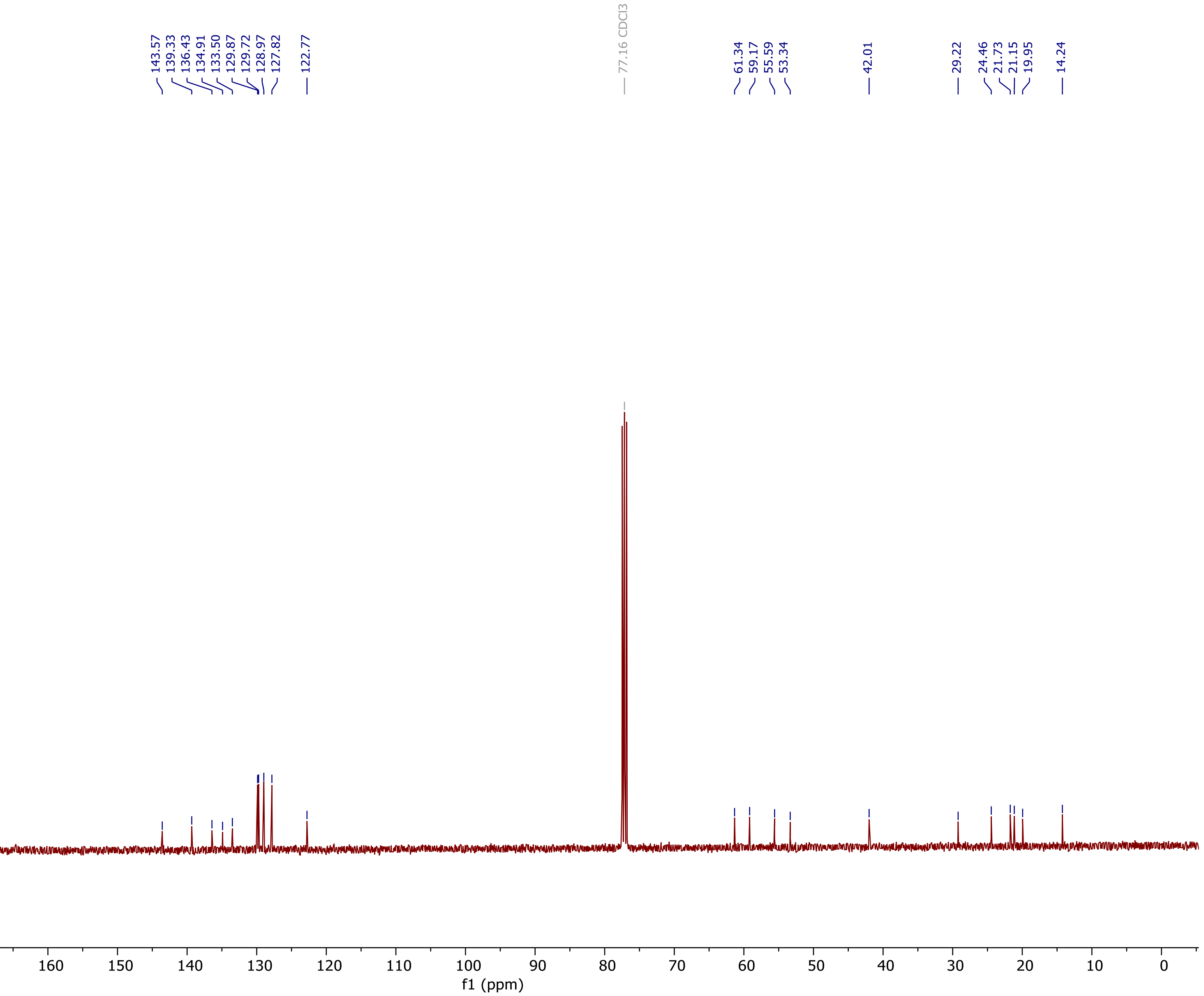

^{13}C NMR spectrum (101 MHz, $CDCl_3$) of compound **5e.**

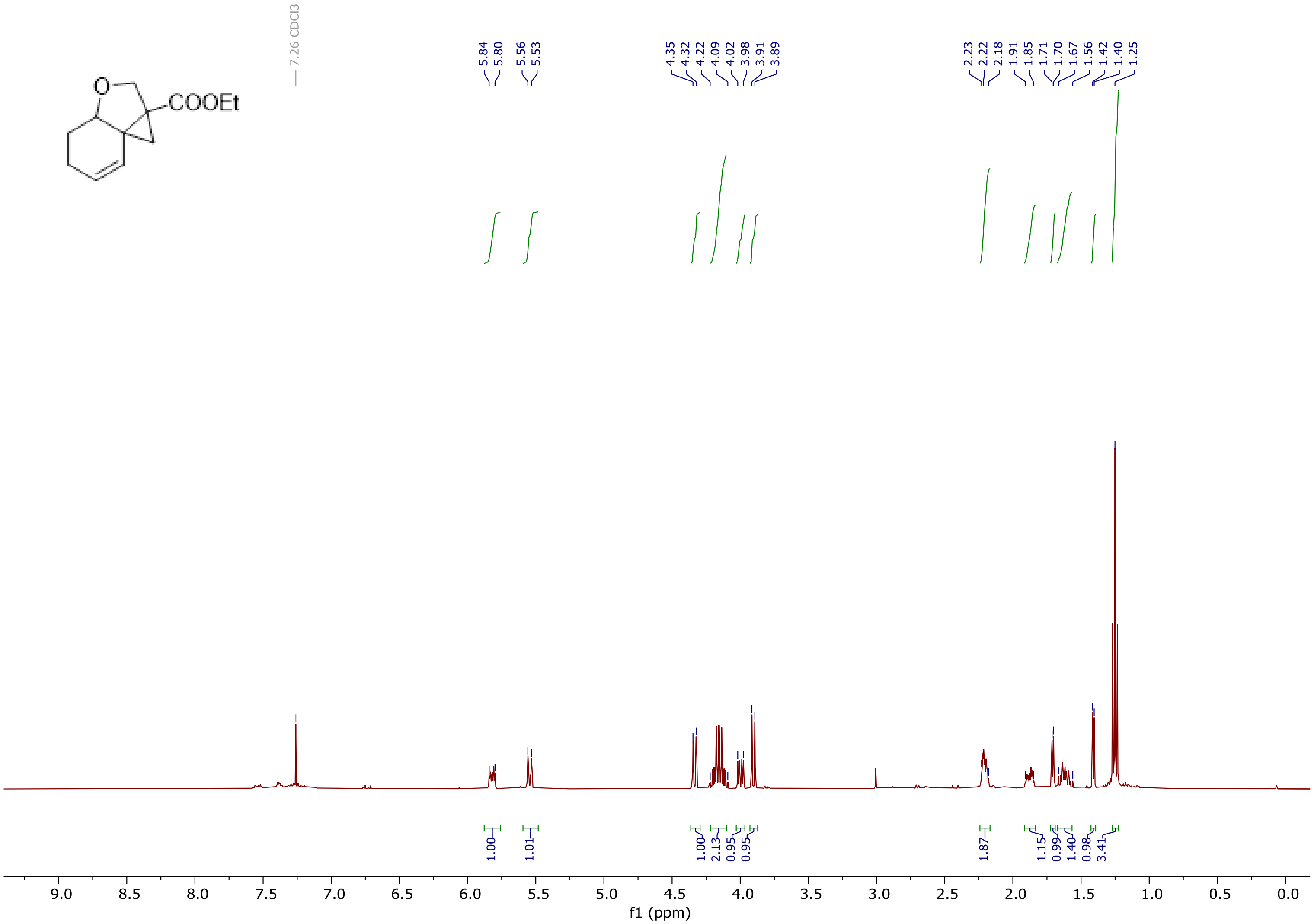

^{1}H NMR spectrum (400 MHz, $CDCl_3$) of compound **4sc**

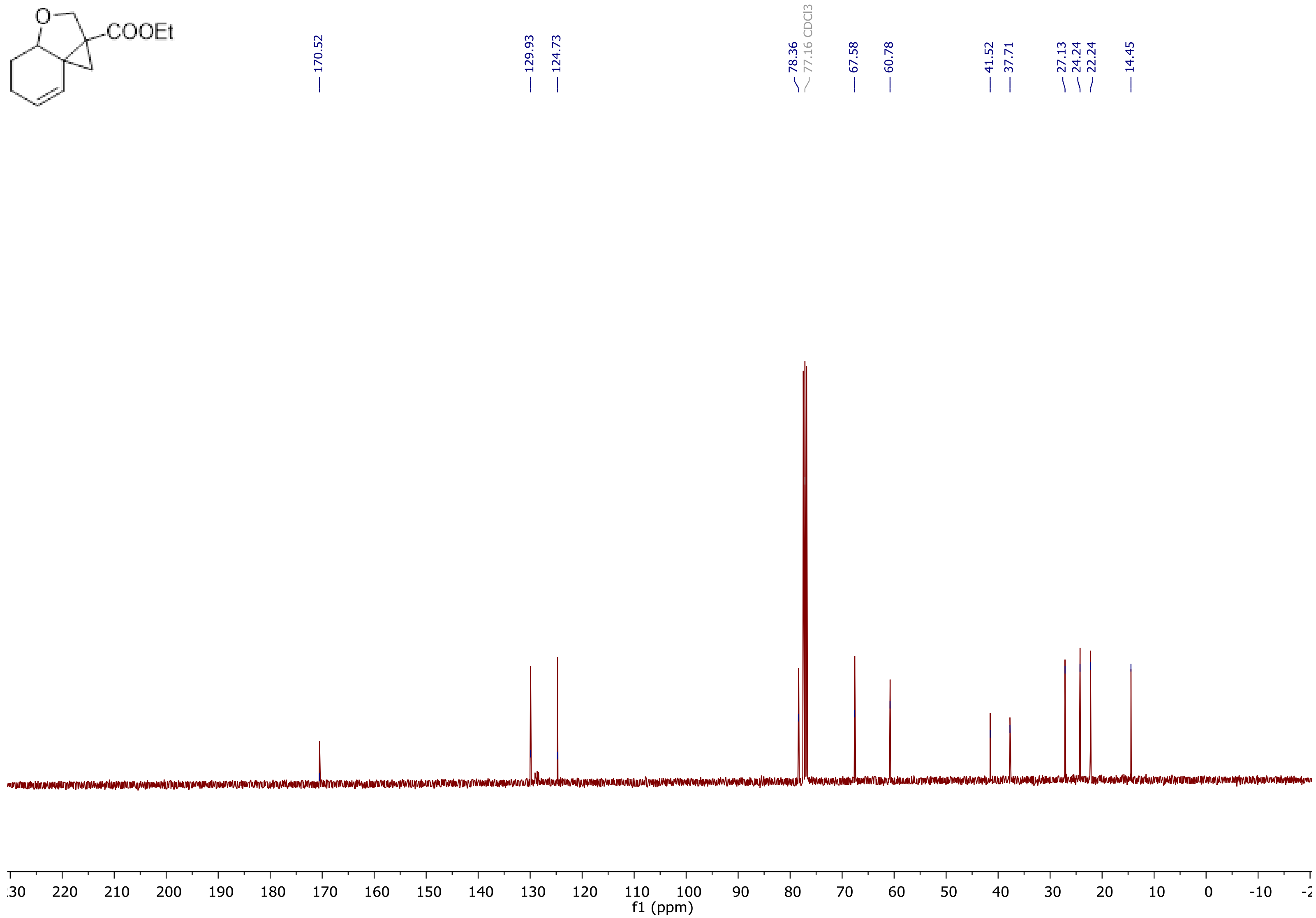

^{13}C NMR spectrum (101 MHz, $CDCl_3$) of compound **4sc**

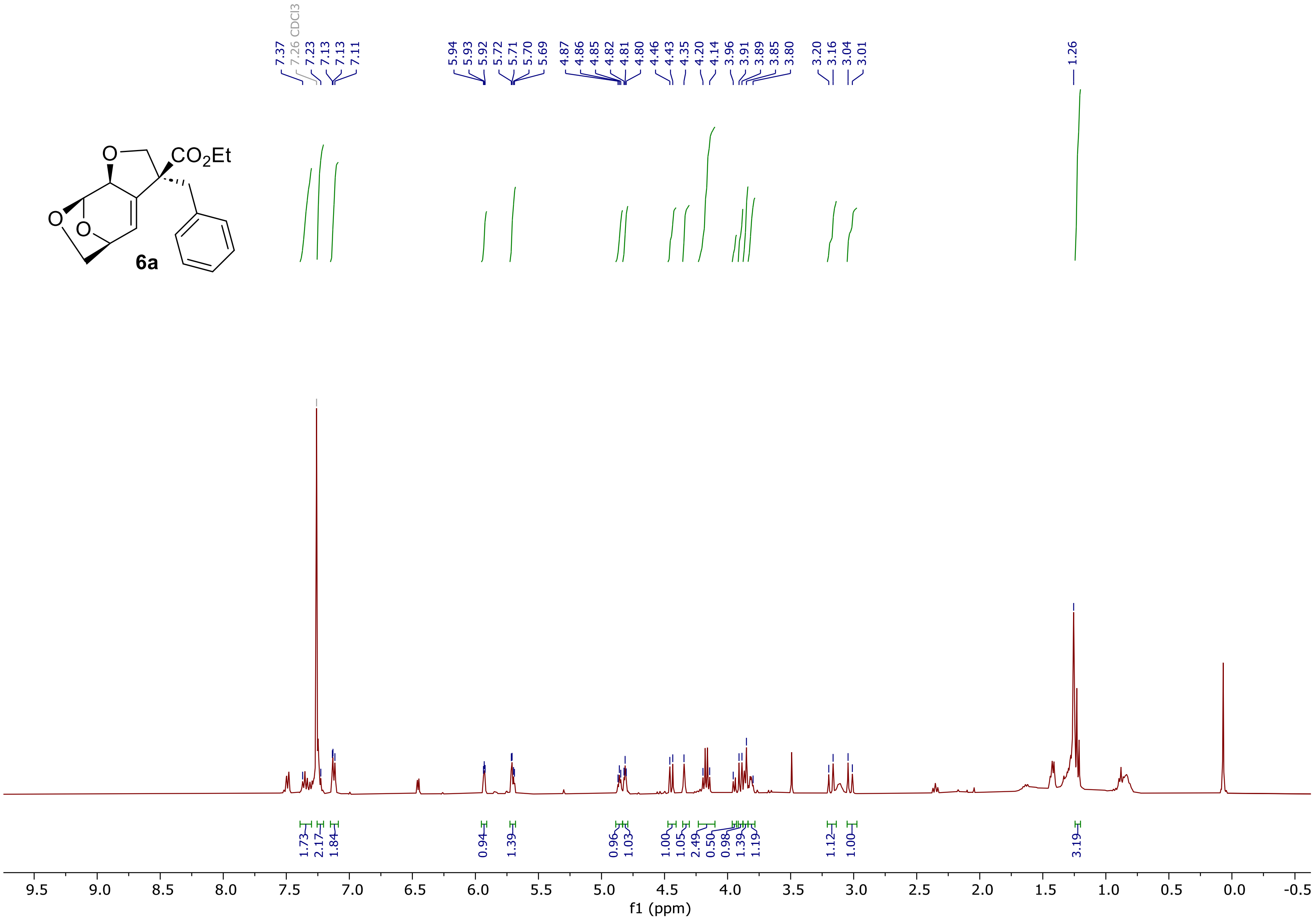

^{1}**H NMR** spectrum (400 MHz, $CDCl_3$) of compound **6a**

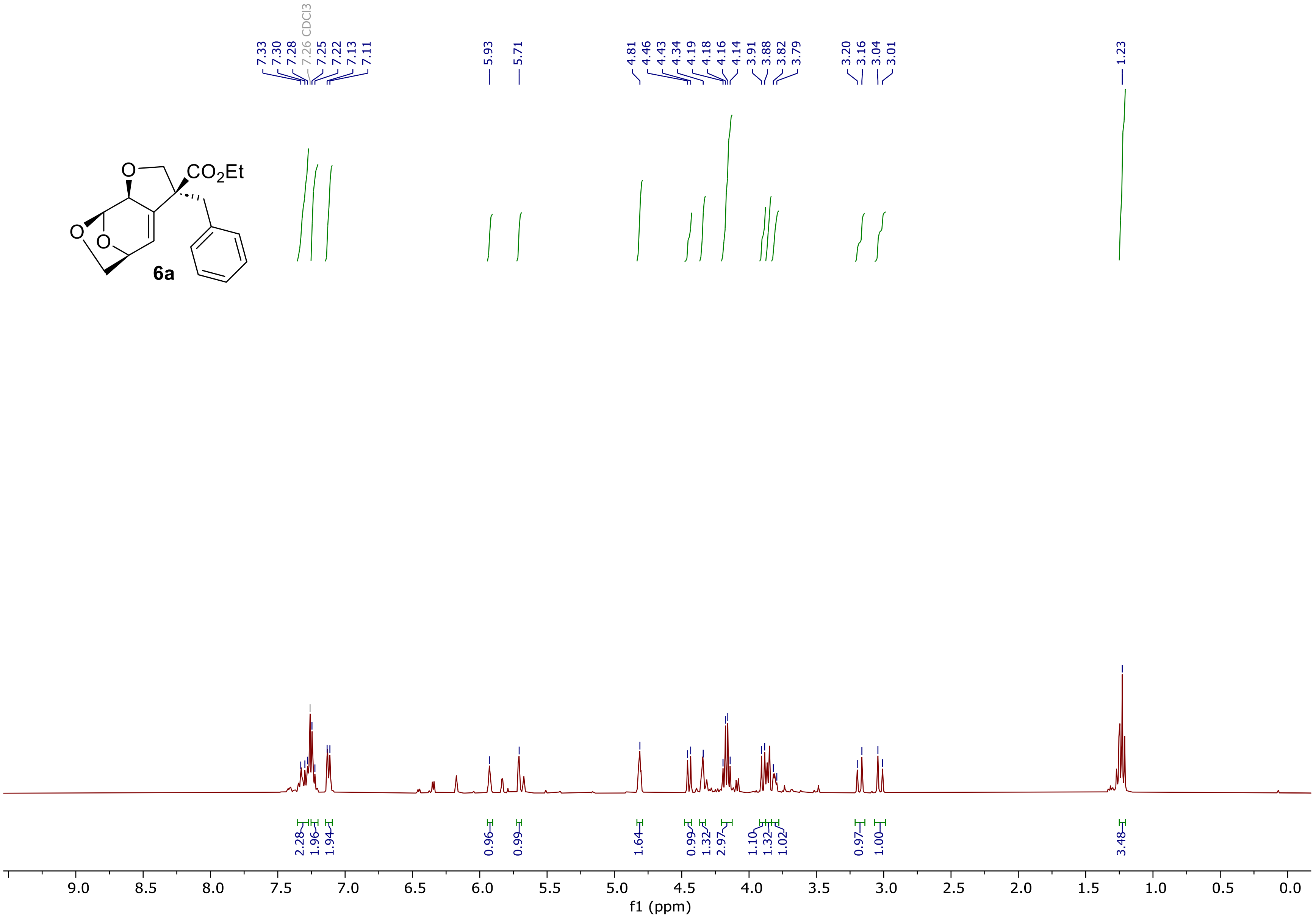

^{1}H NMR spectrum (400 MHz, $CDCl_3$) of compound **6a**

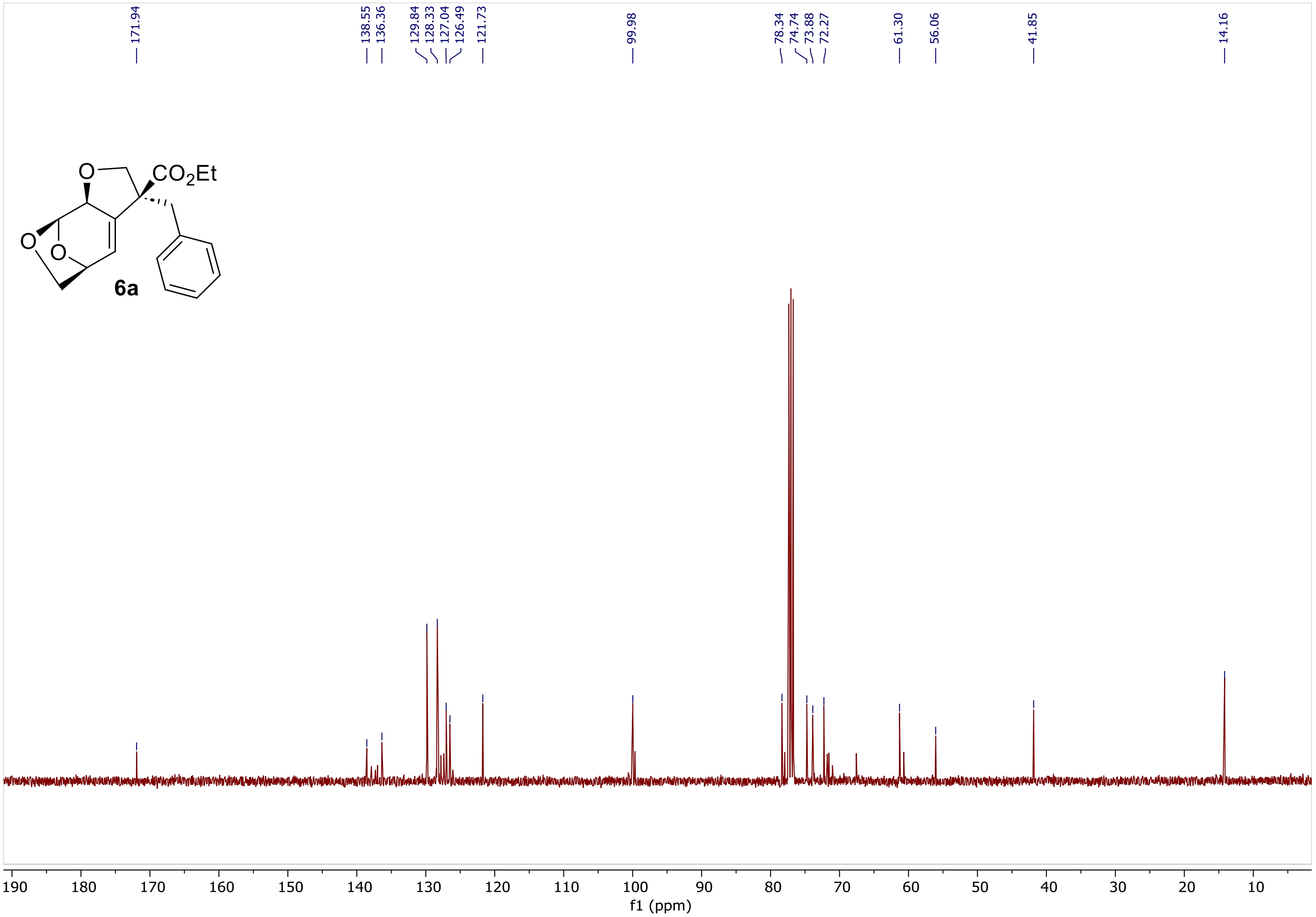

^{13}C NMR spectrum (101 MHz, $CDCl_3$) of compound **6a**

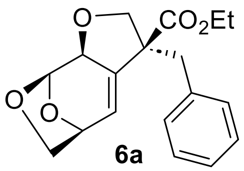

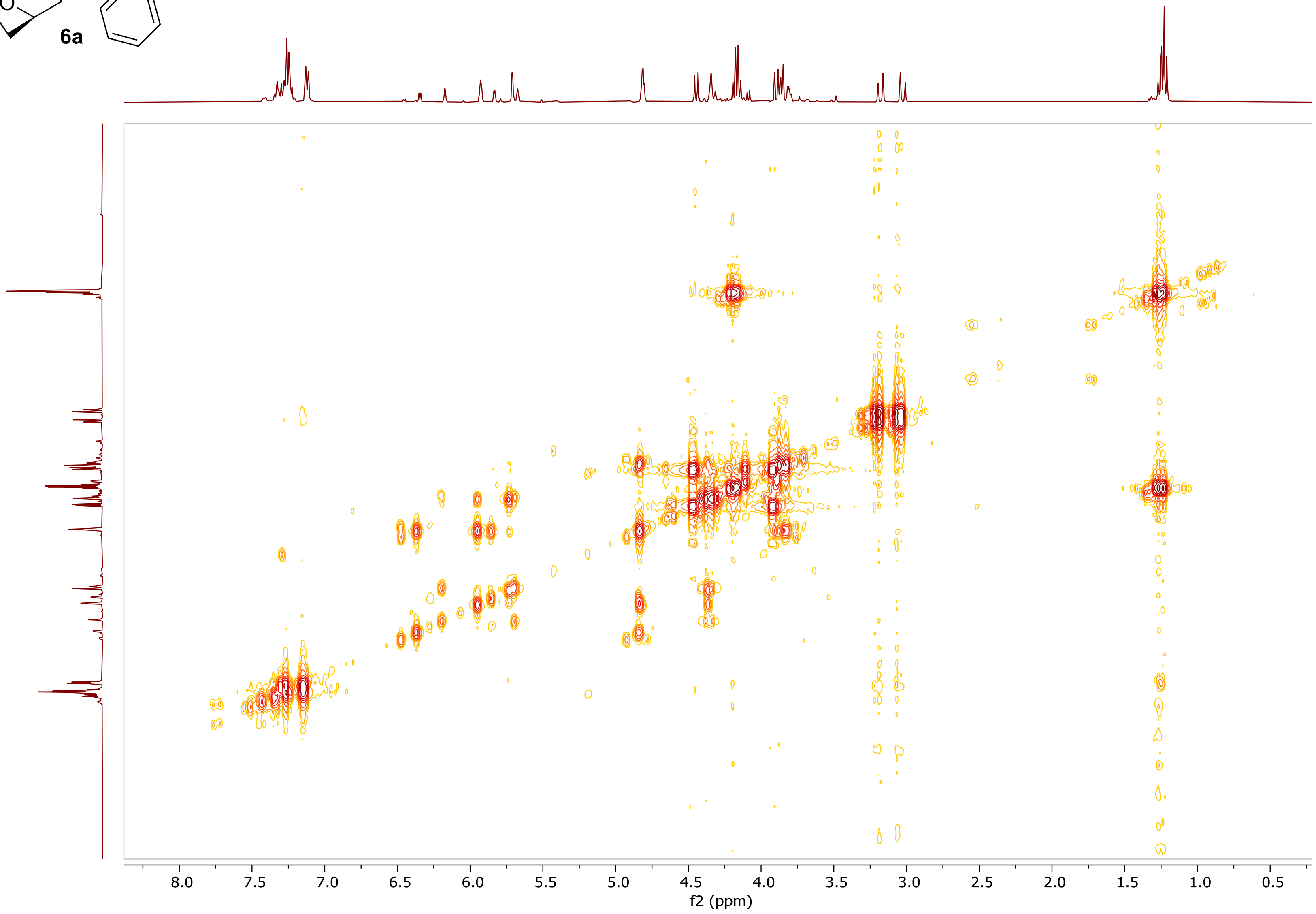

2D COSY Spectrum (400 MHz, $CDCl_3$) of compound **6a**

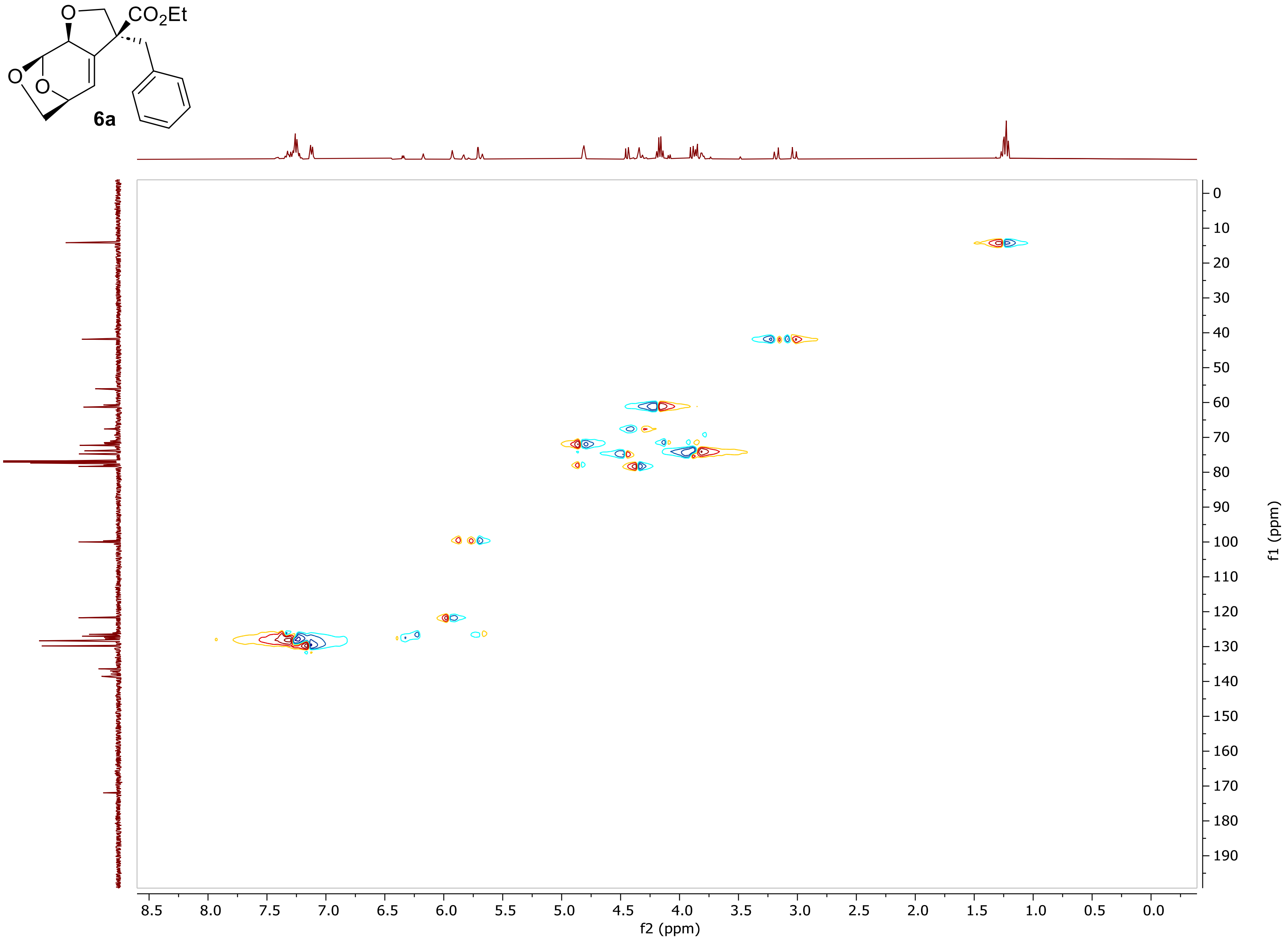

2D HSQC Spectrum (400 MHz, $CDCl_3$) of compound **6a**

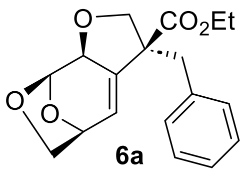

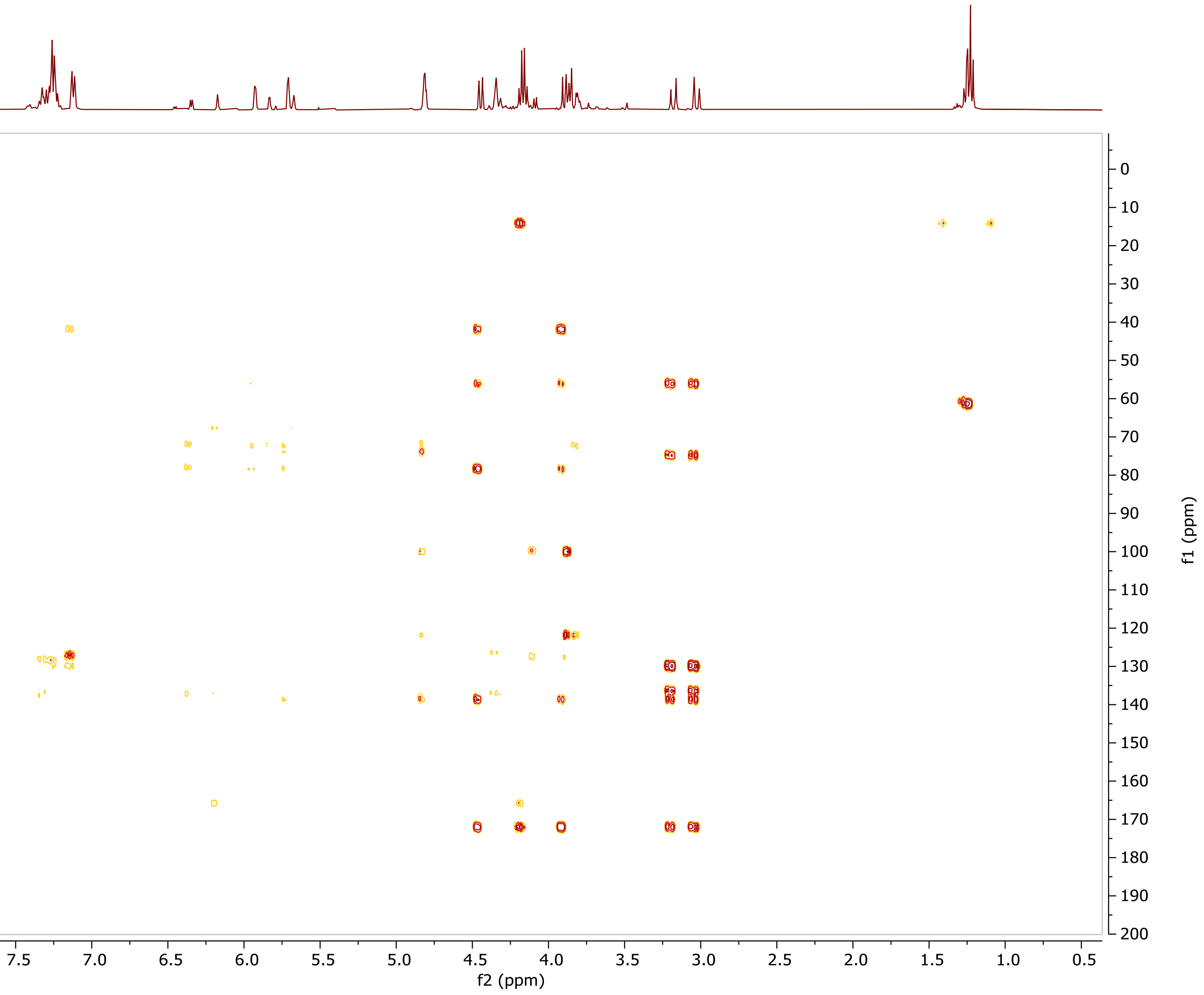

2D HMBC Spectrum (101 MHz, $CDCl_3$) of Compound **6a**

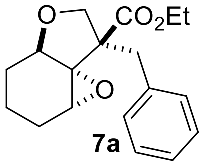

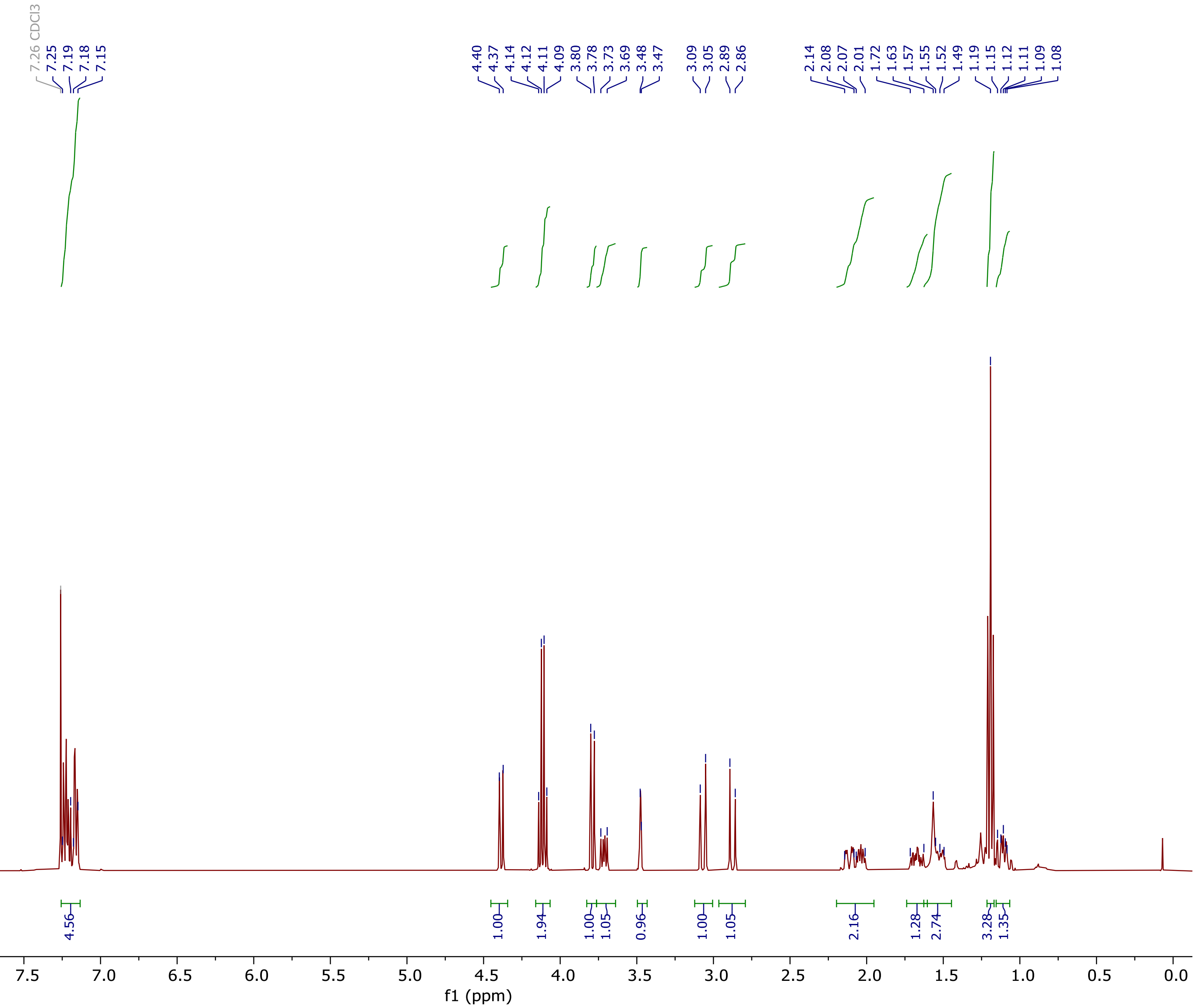

^{1}**H NMR** spectrum (400 MHz, $CDCl_3$) of compound **7a**

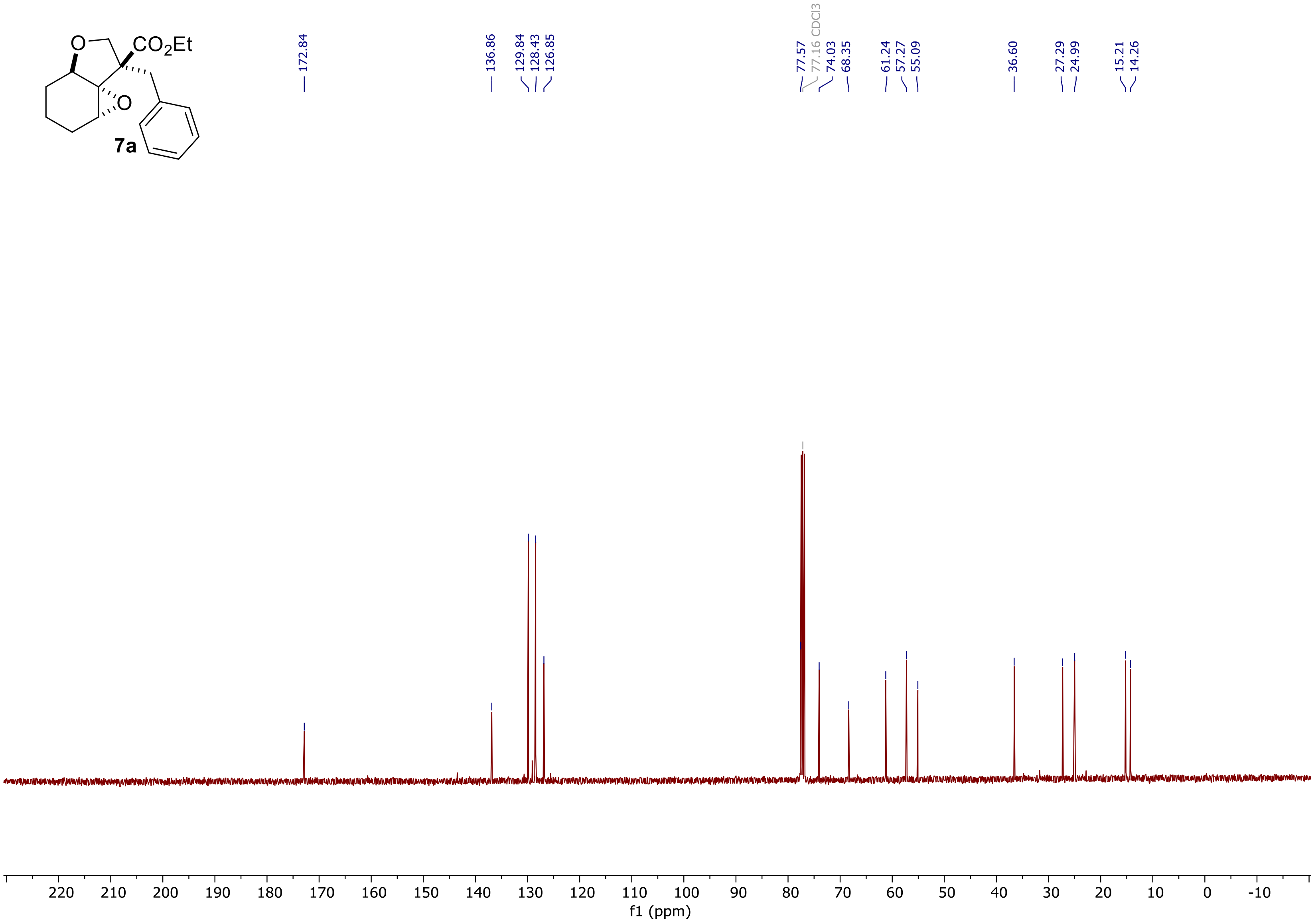

^{13}C NMR spectrum (101 MHz, $CDCl_3$) of compound **7a**

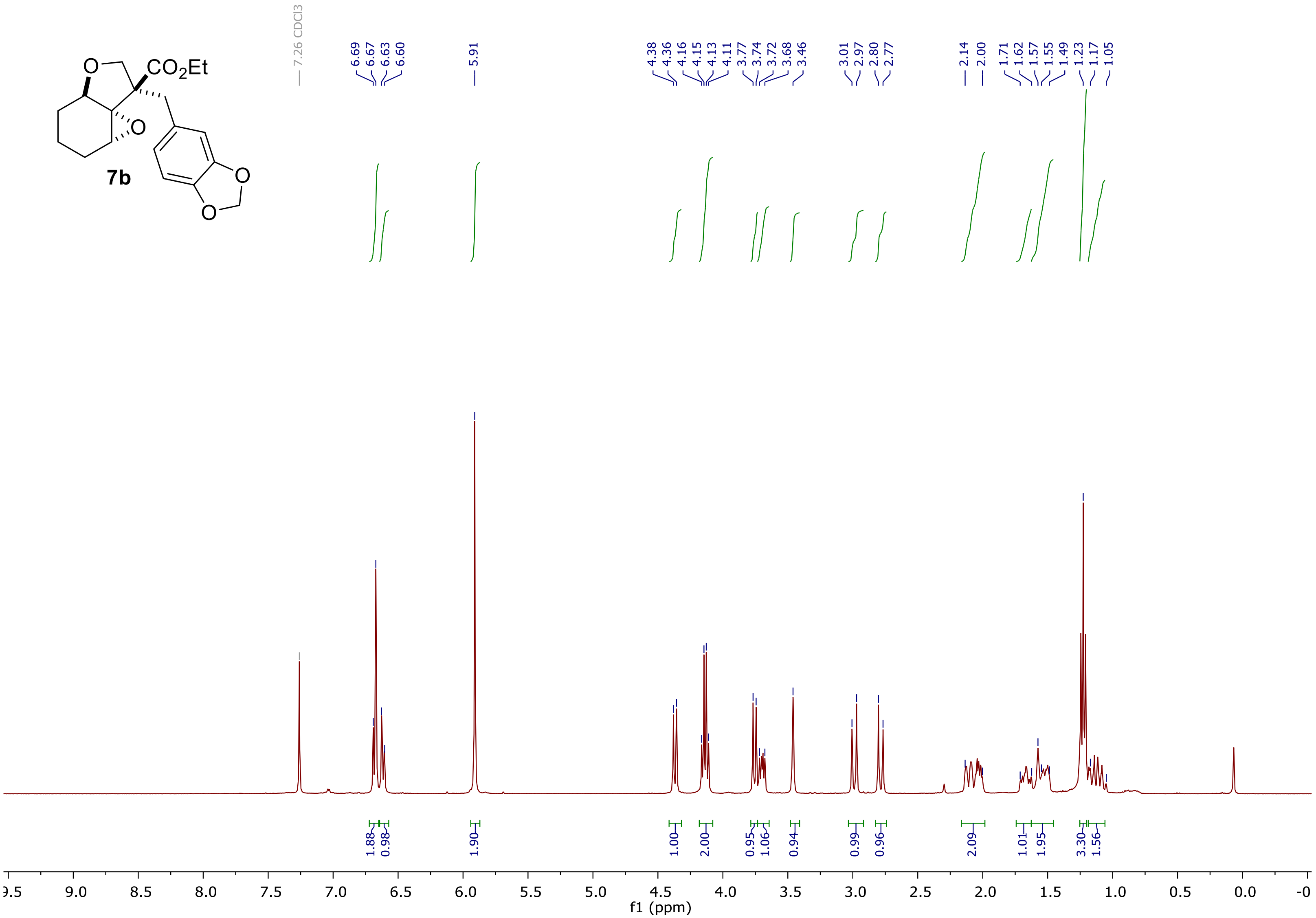

^{1}H NMR spectrum (400 MHz, $CDCl_3$) of compound **7b**

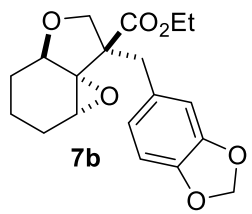

7b

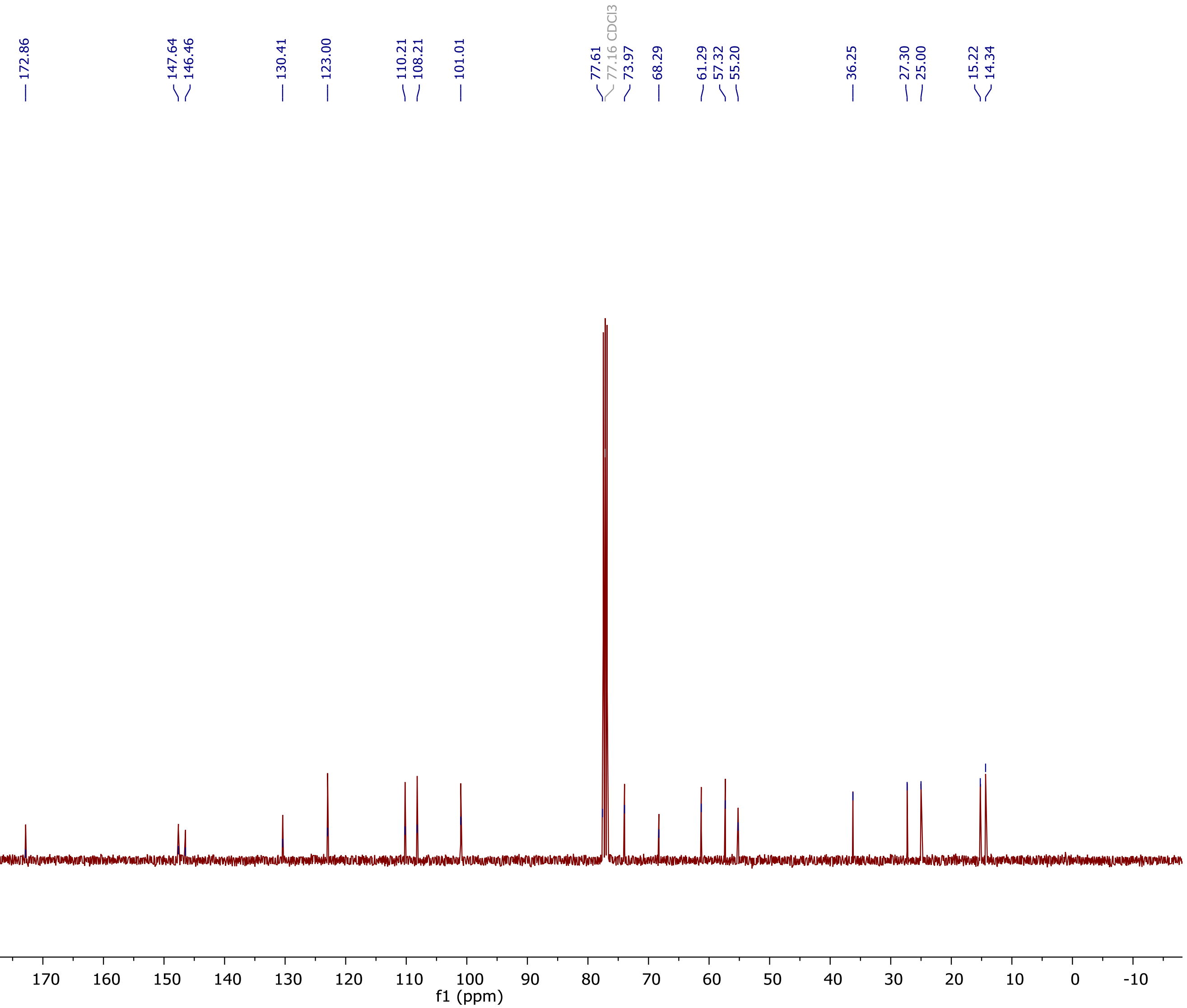

^{13}C NMR spectrum (101 MHz, $CDCl_3$) of compound **7b**

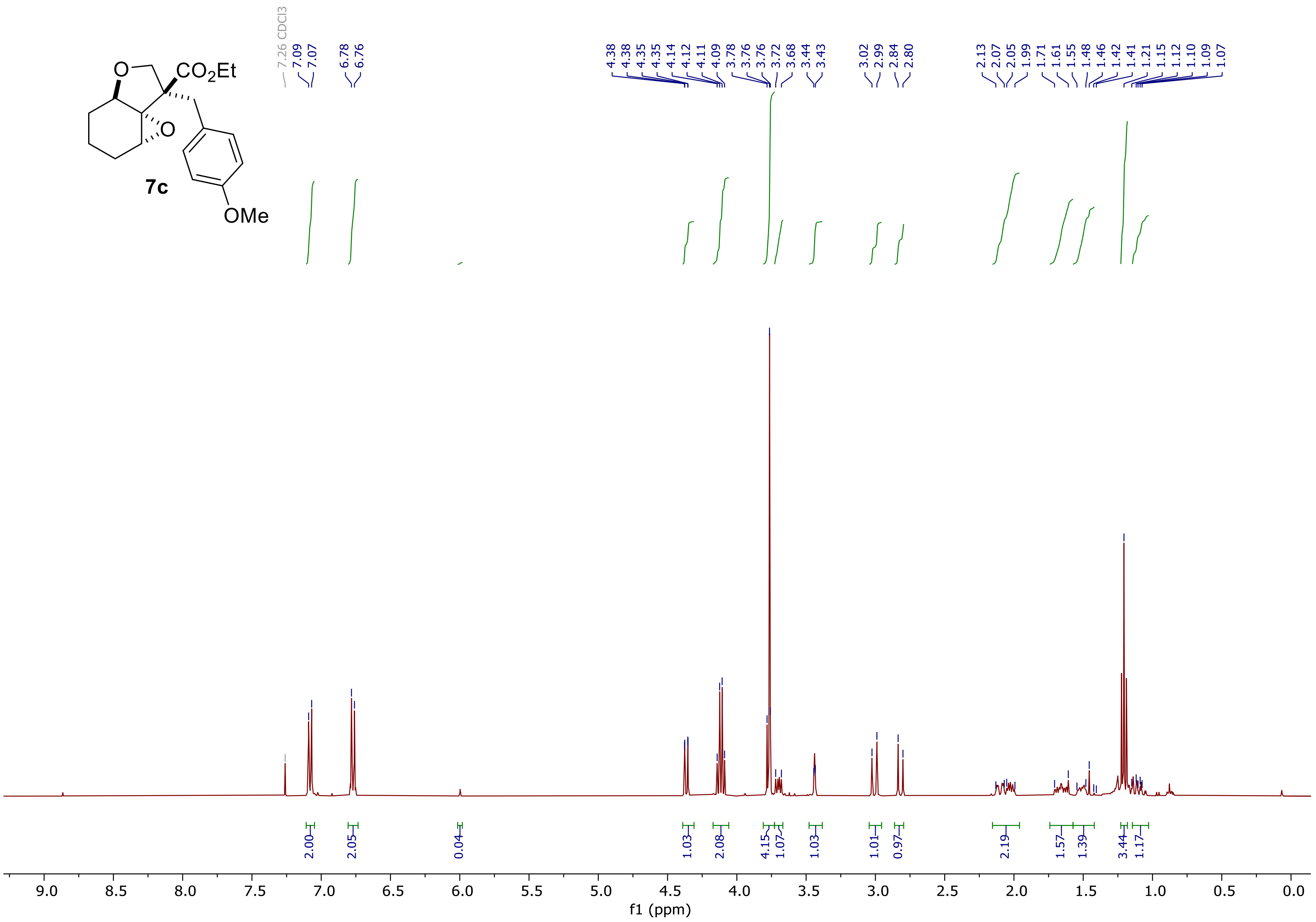

^{1}**H NMR** spectrum (400 MHz, $CDCl_3$) of compound **7c**

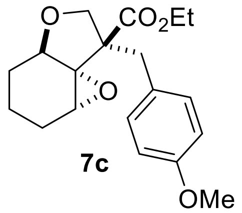

7c

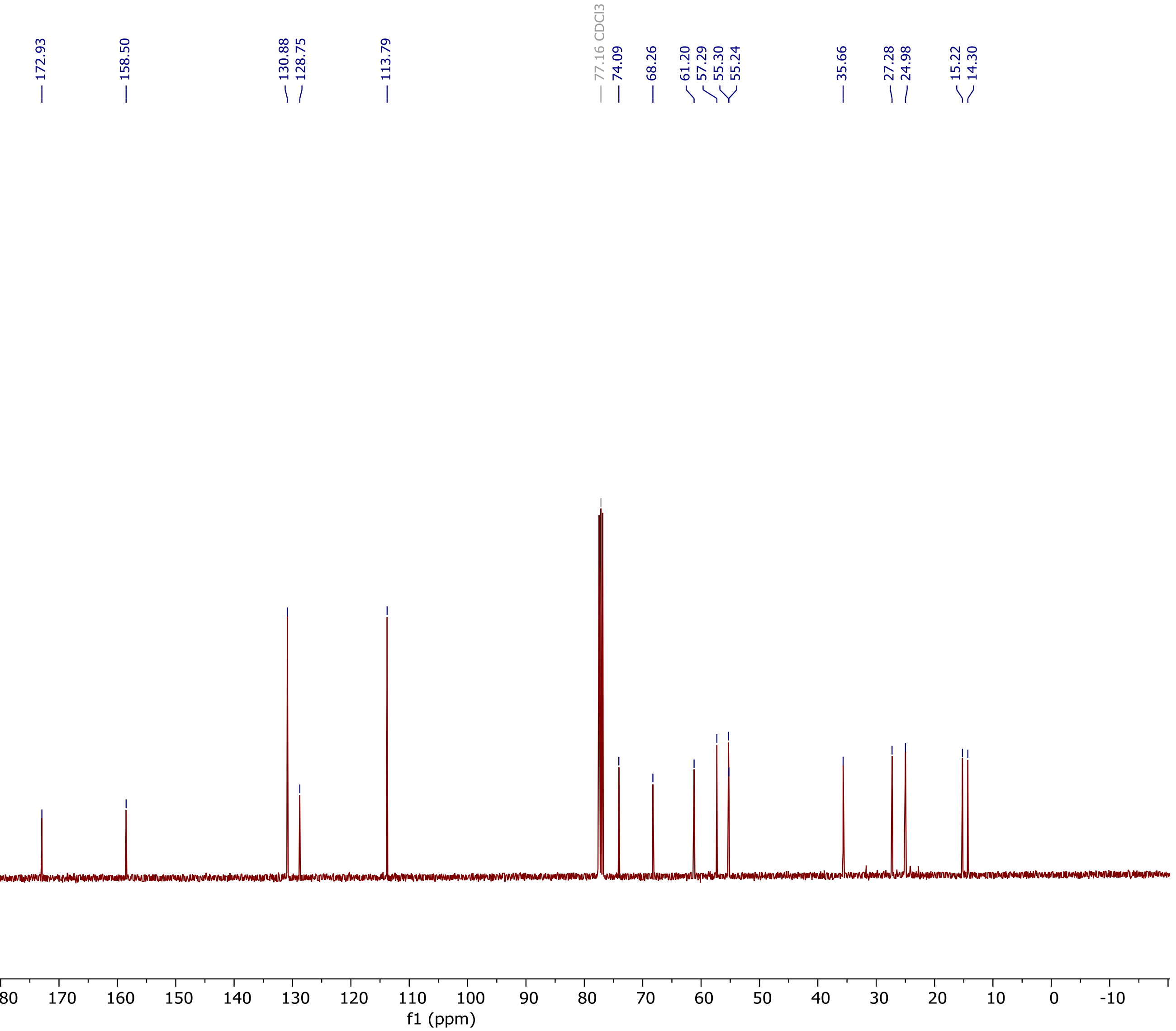

^{13}**C NMR** spectrum (101 MHz, $CDCl_3$) of compound **7c**

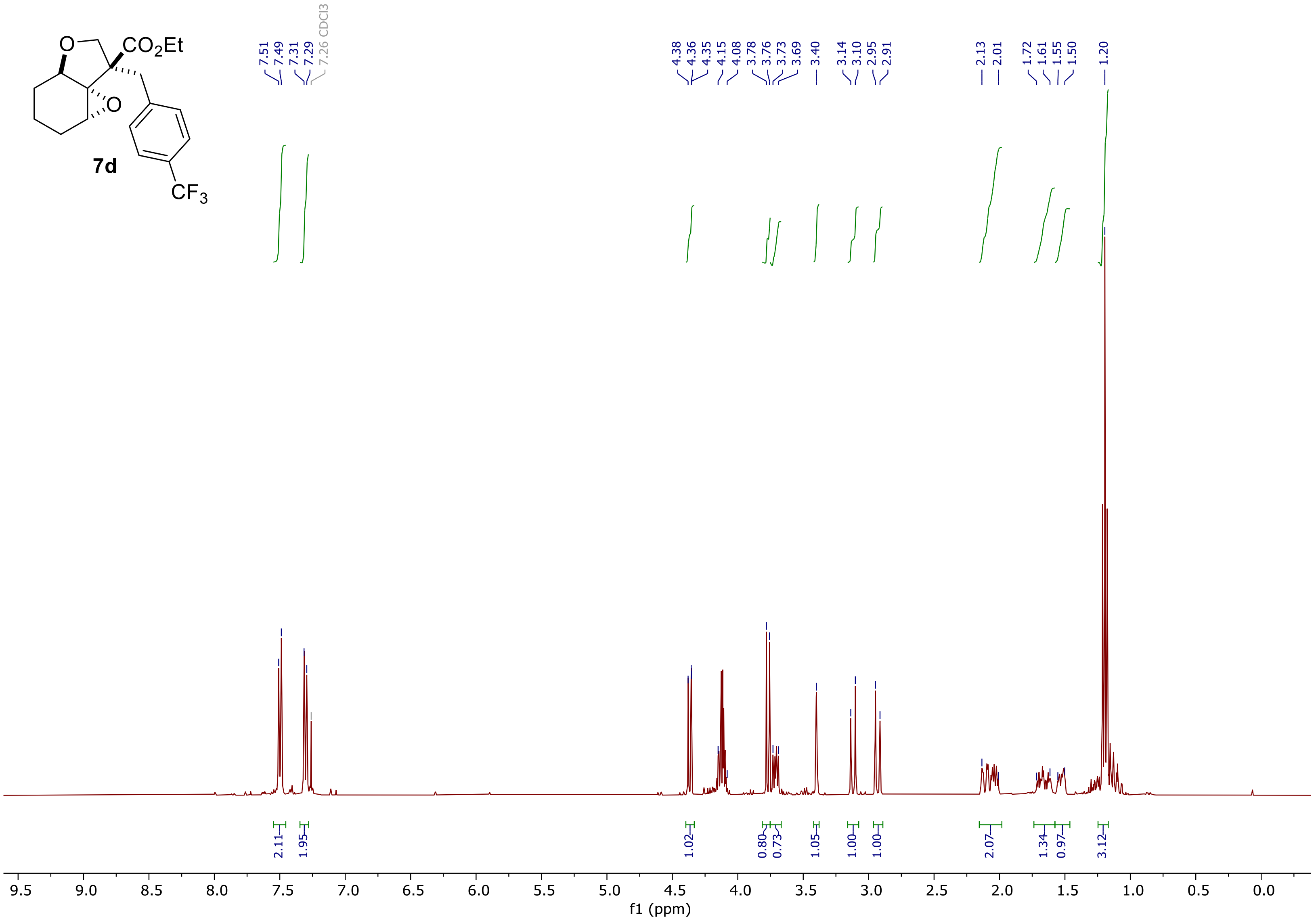

^{1}H NMR spectrum (400 MHz, $CDCl_3$) of compound **7d**

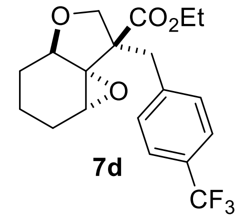

7d

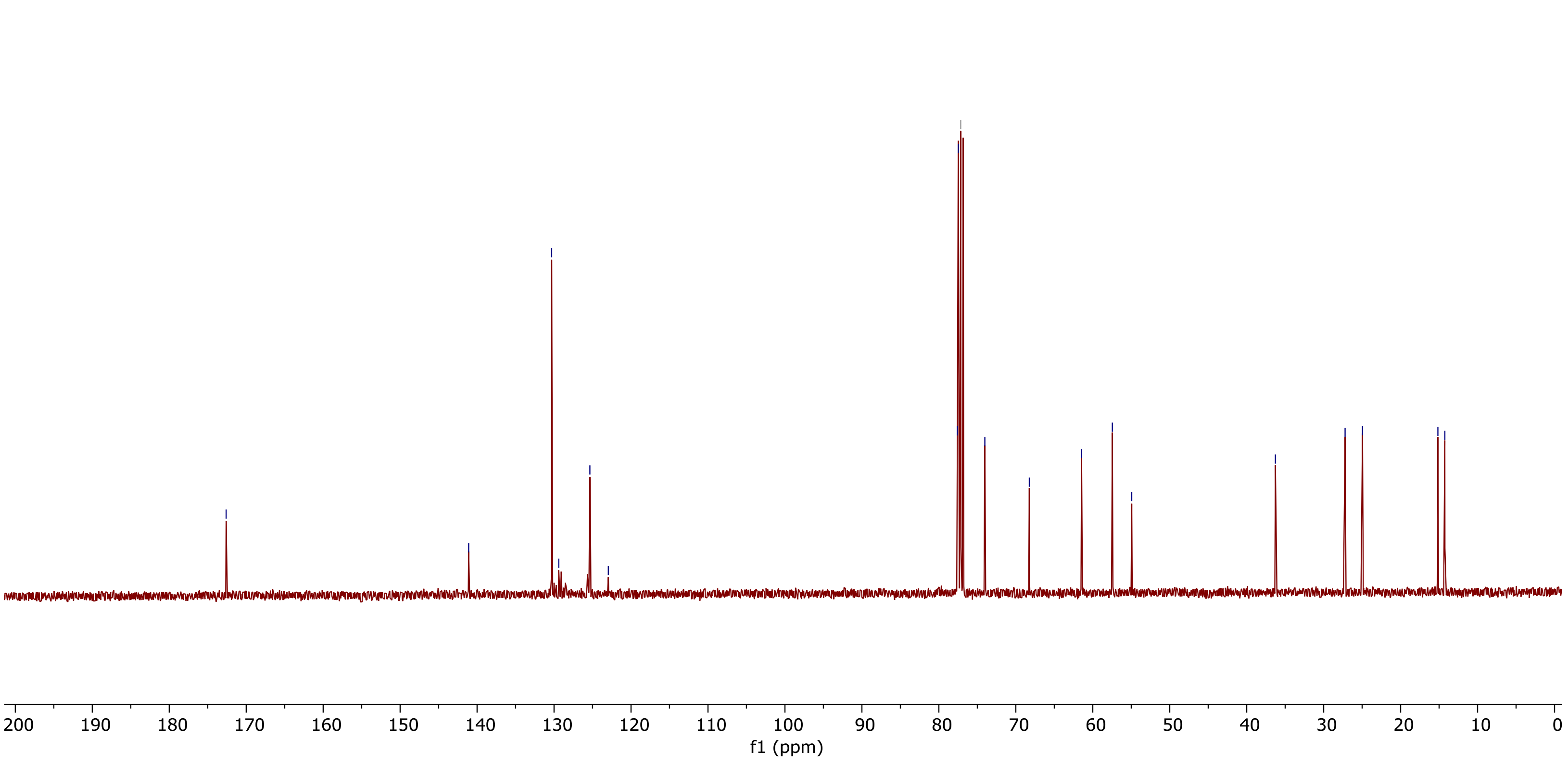

^{13}**C NMR** spectrum (101 MHz, $CDCl_3$) of compound **7d**

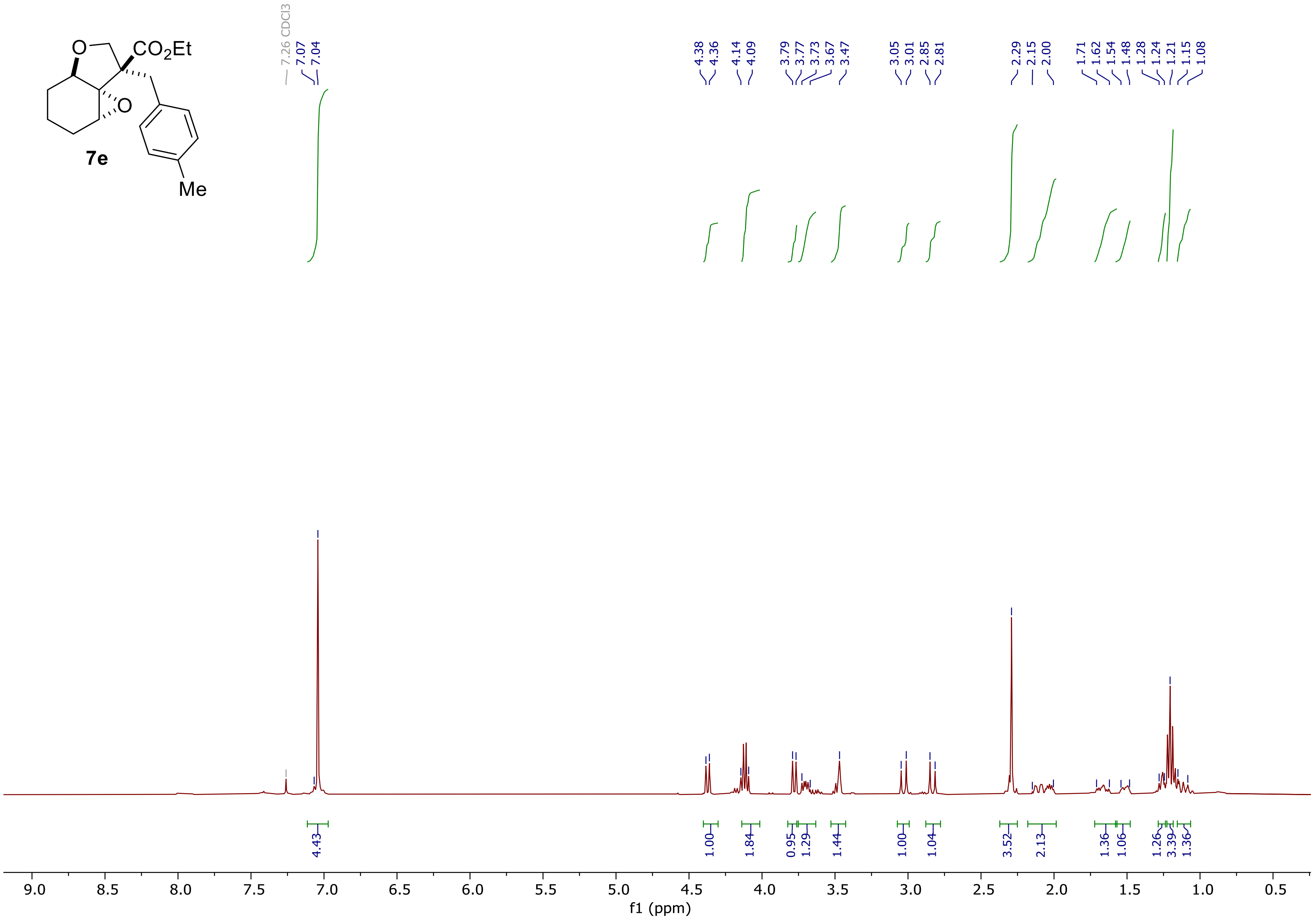

^{1}H NMR spectrum (400 MHz, $CDCl_3$) of compound **7e**

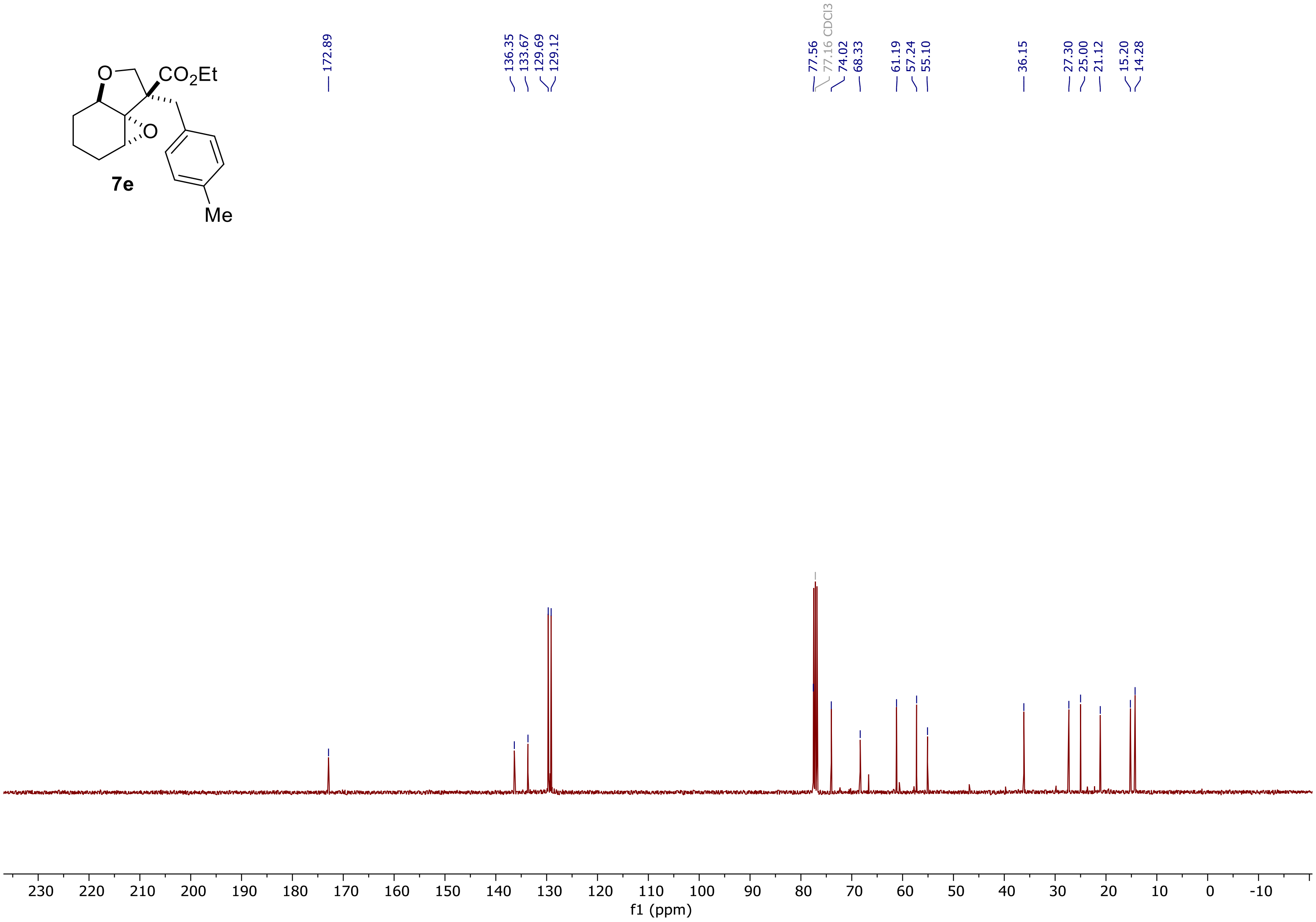

^{13}C NMR spectrum (101 MHz, $CDCl_3$) of compound **7e**

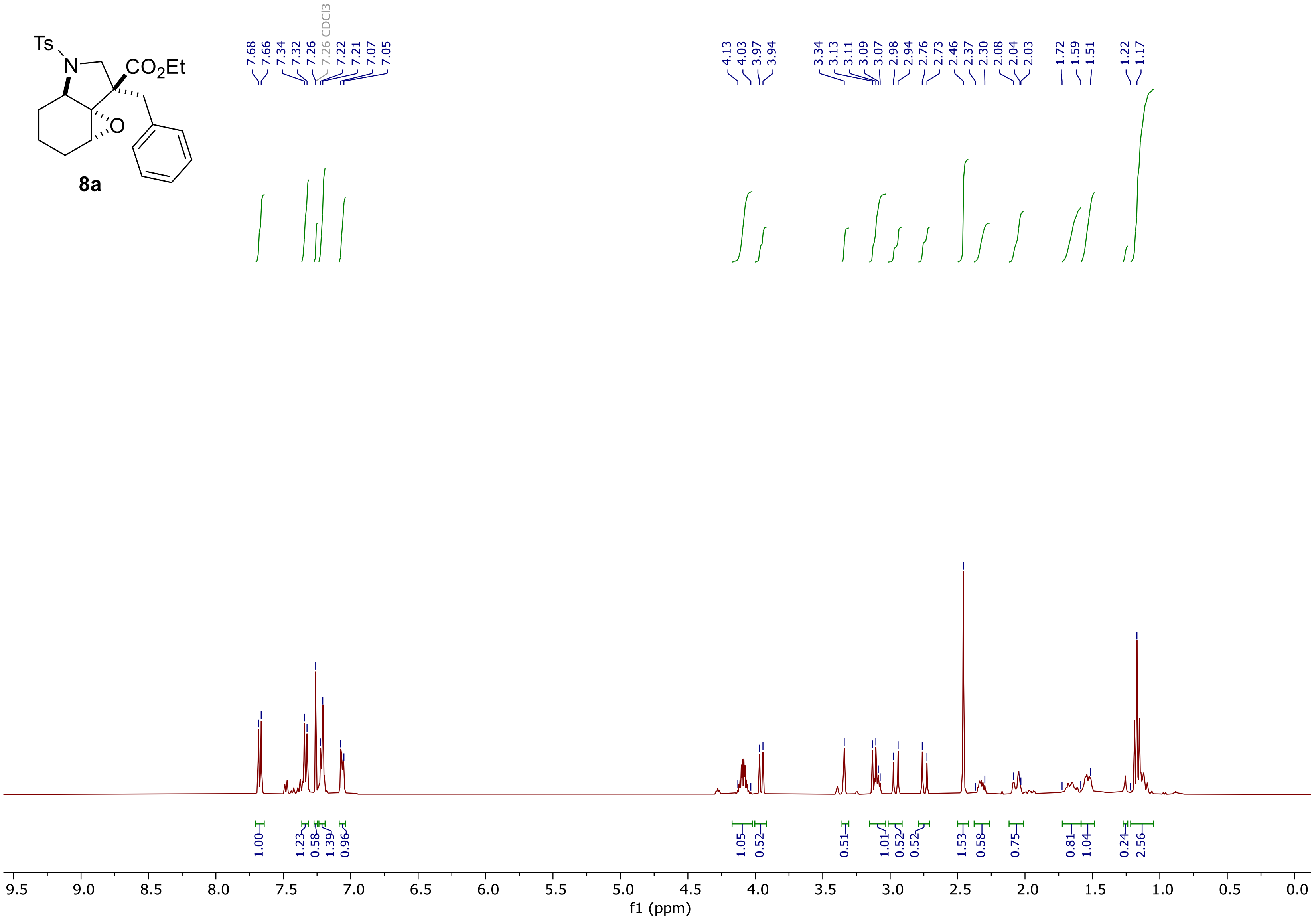

^{1}**H NMR** spectrum (400 MHz, $CDCl_3$) of compound **8a**

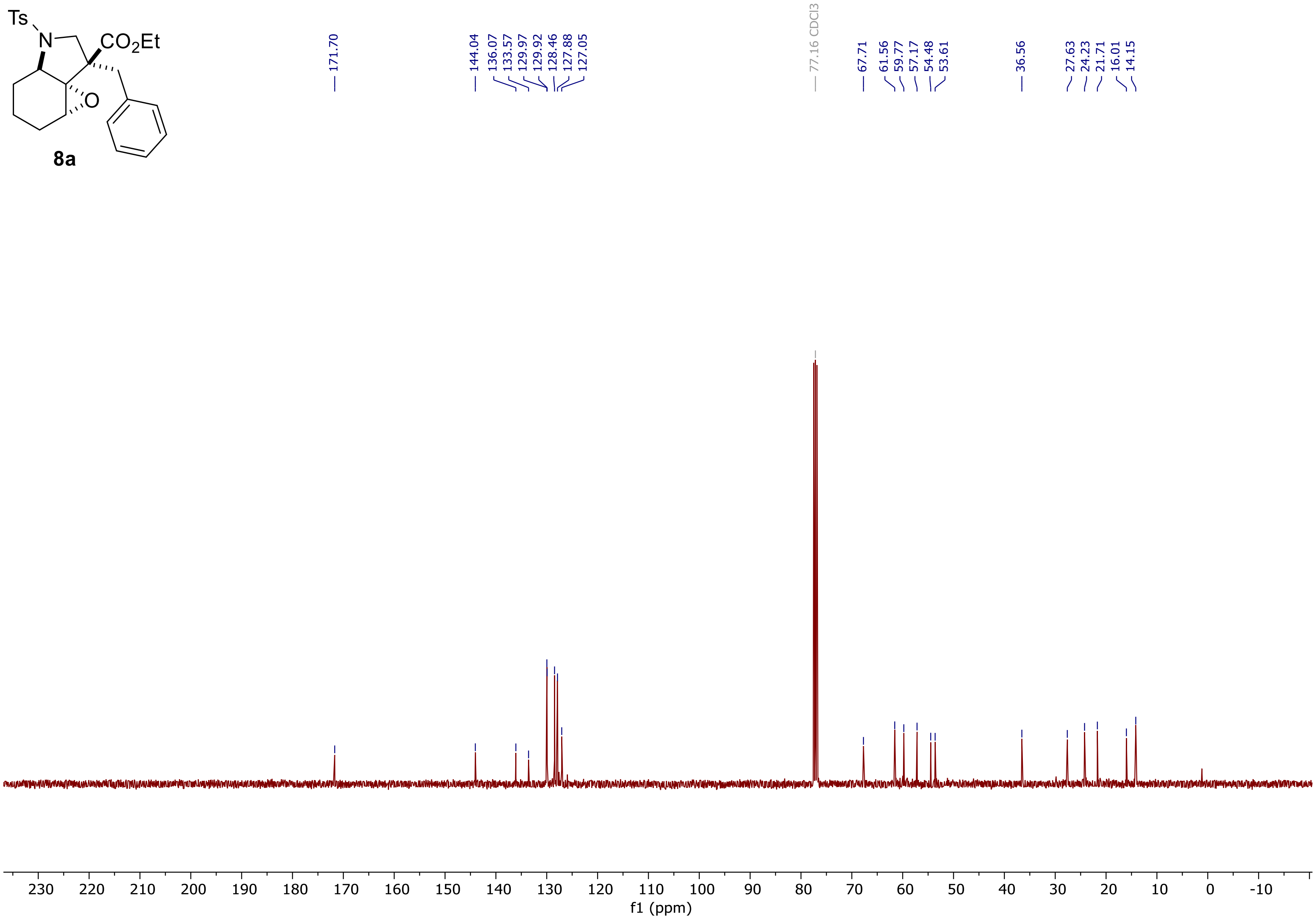

^{13}C NMR spectrum (101 MHz, $CDCl_3$) of compound **8a**

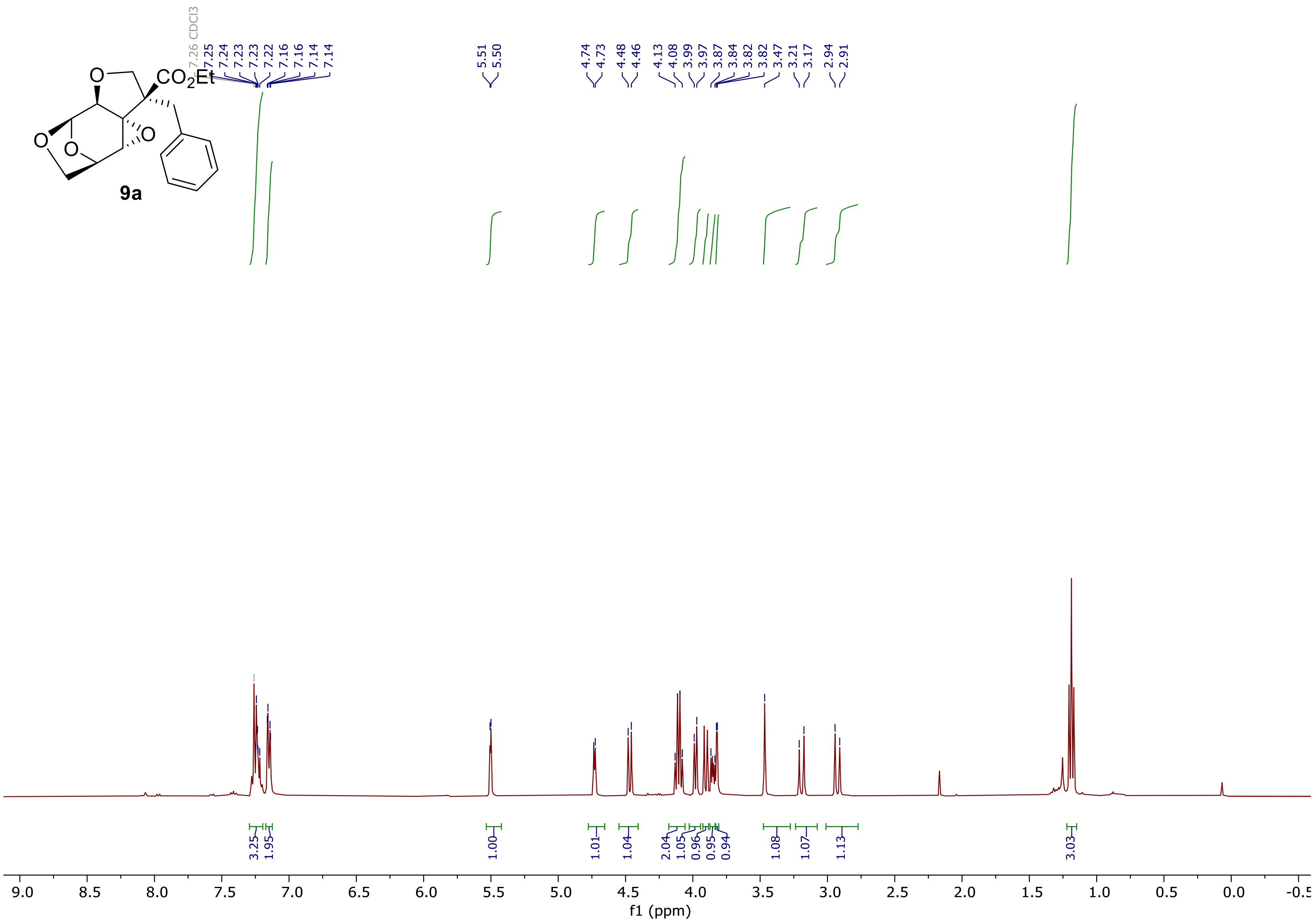

^{1}H NMR spectrum (400 MHz, $CDCl_3$) of compound **9a**

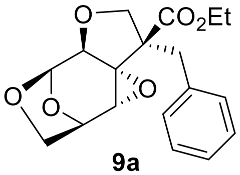

9a

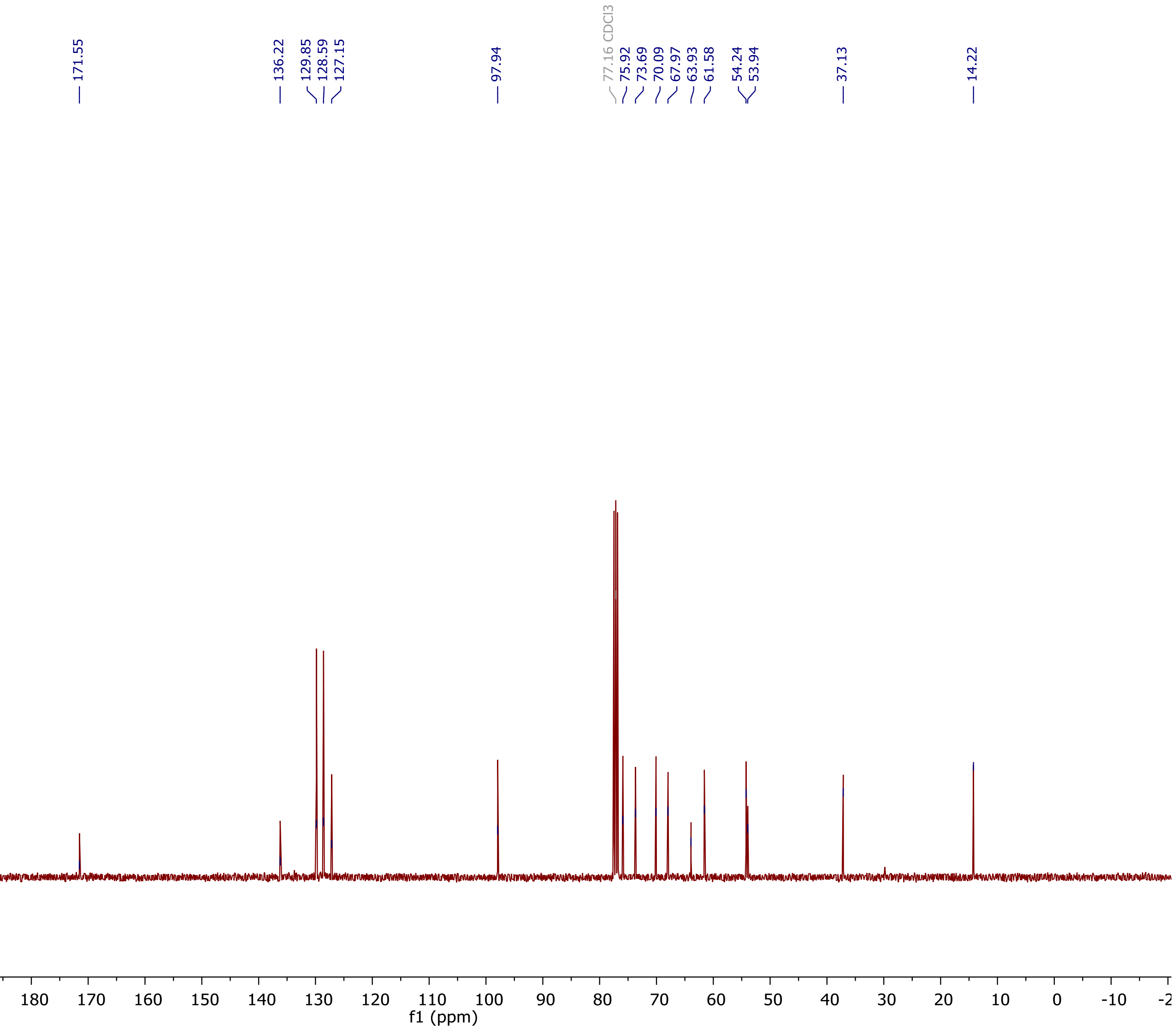

[13]**C NMR** spectrum (101 MHz, $CDCl_3$) of compound **9e**

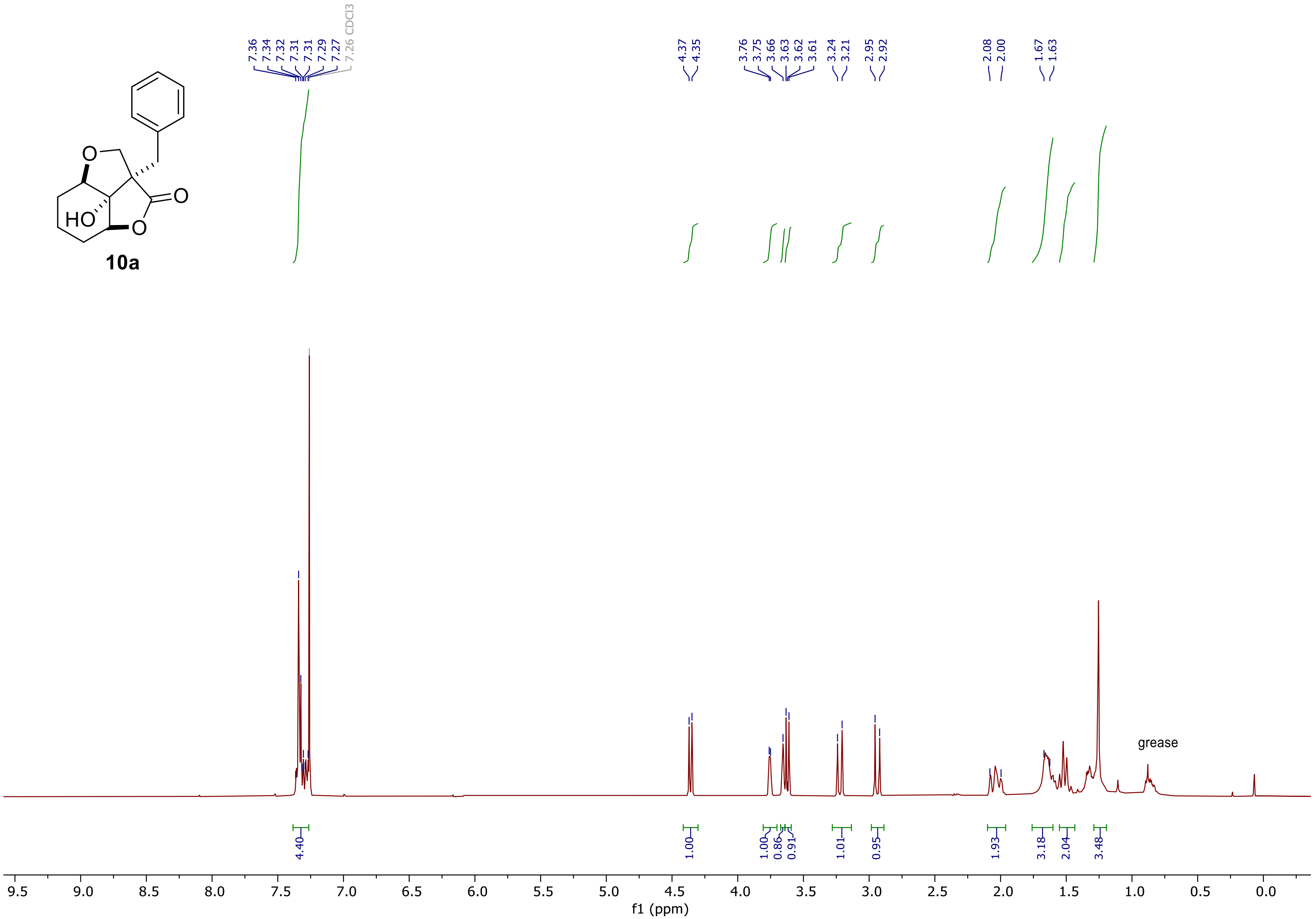

¹H NMR spectrum (400 MHz, $CDCl_3$) of compound **10a**

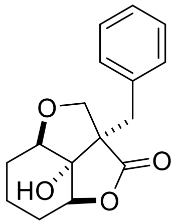

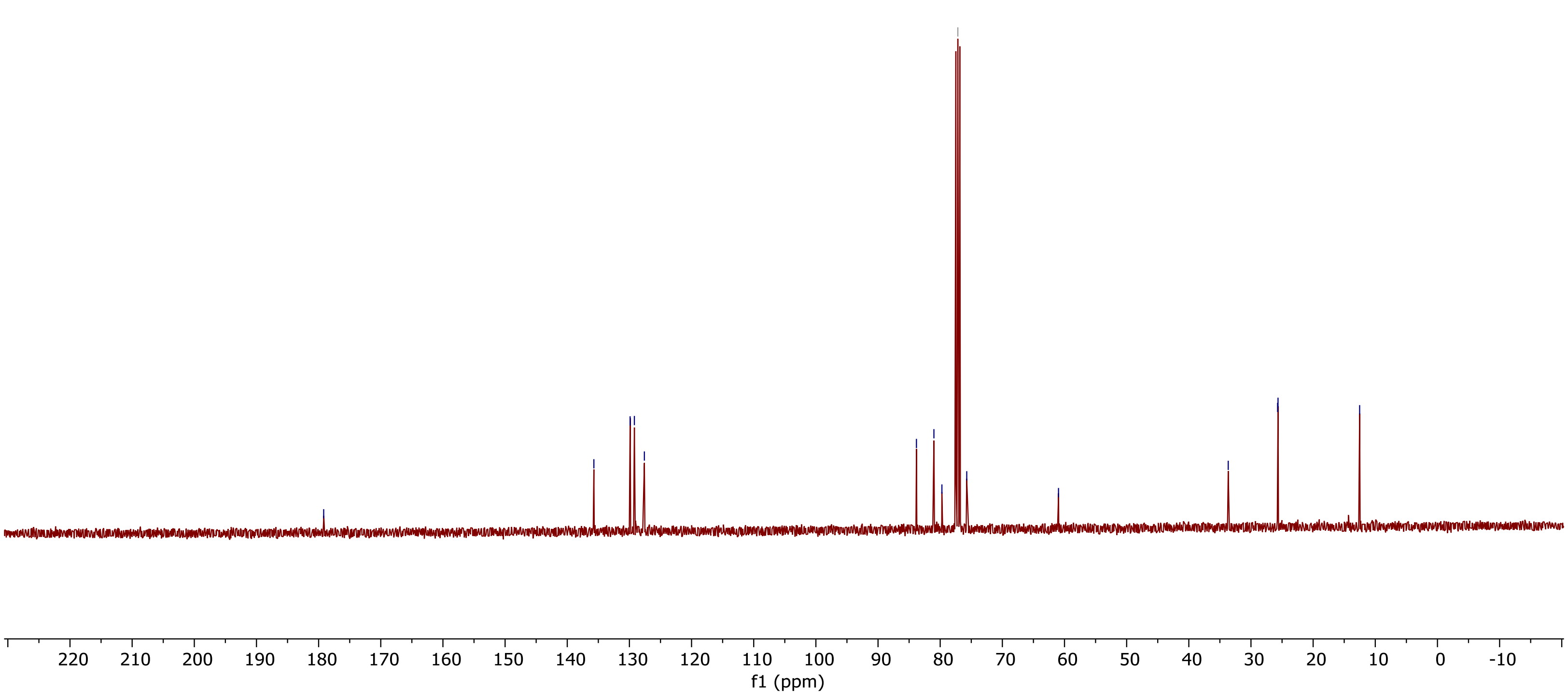

^{13}C NMR spectrum (101 MHz, $CDCl_3$) of compound **10a**

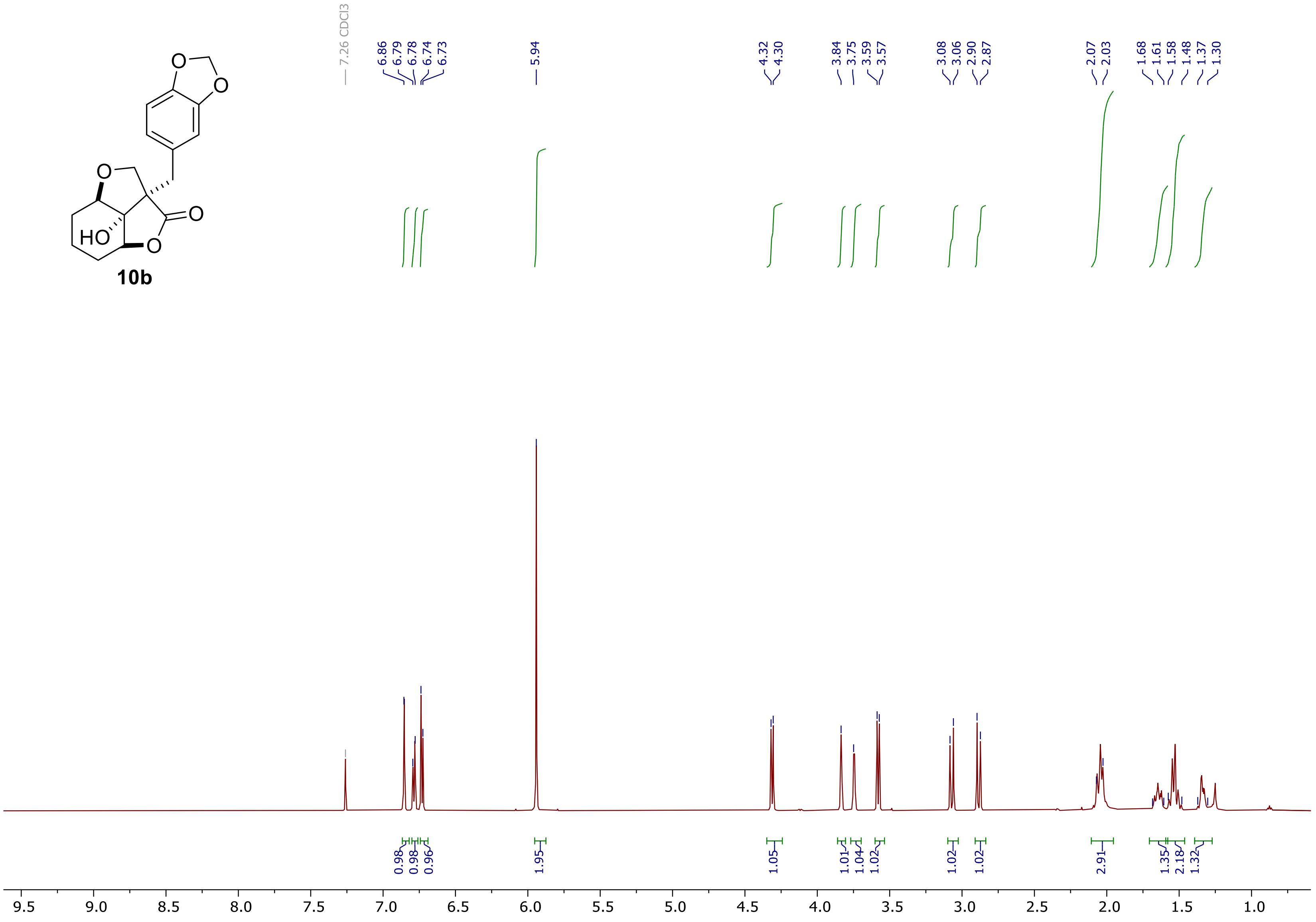

^{1}H NMR spectrum (600 MHz, $CDCl_3$) of compound **10b**

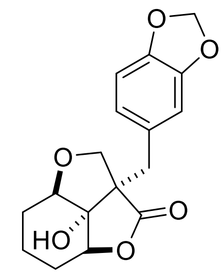

10b

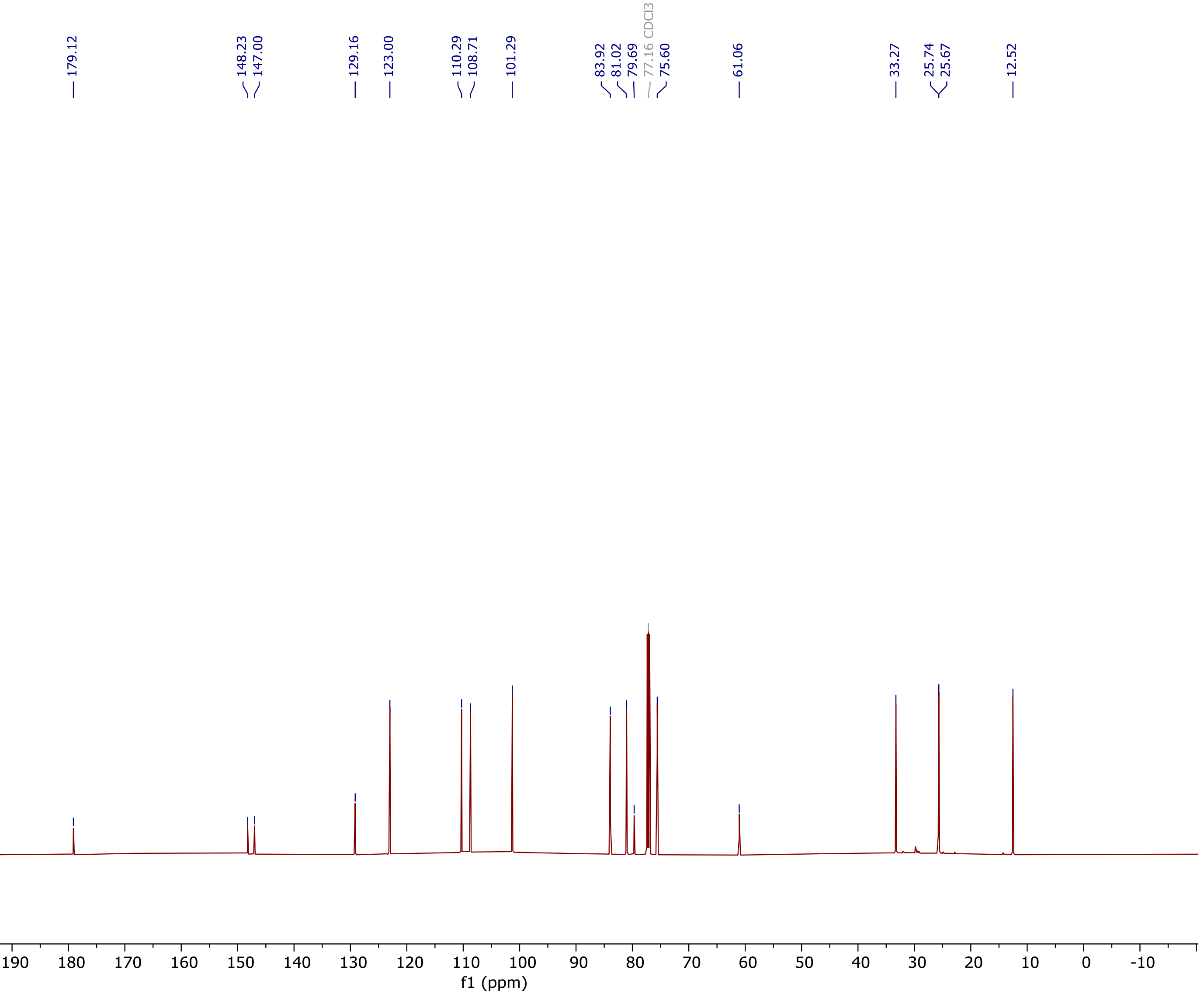

^{13}C NMR spectrum (151 MHz, $CDCl_3$) of compound **10b**

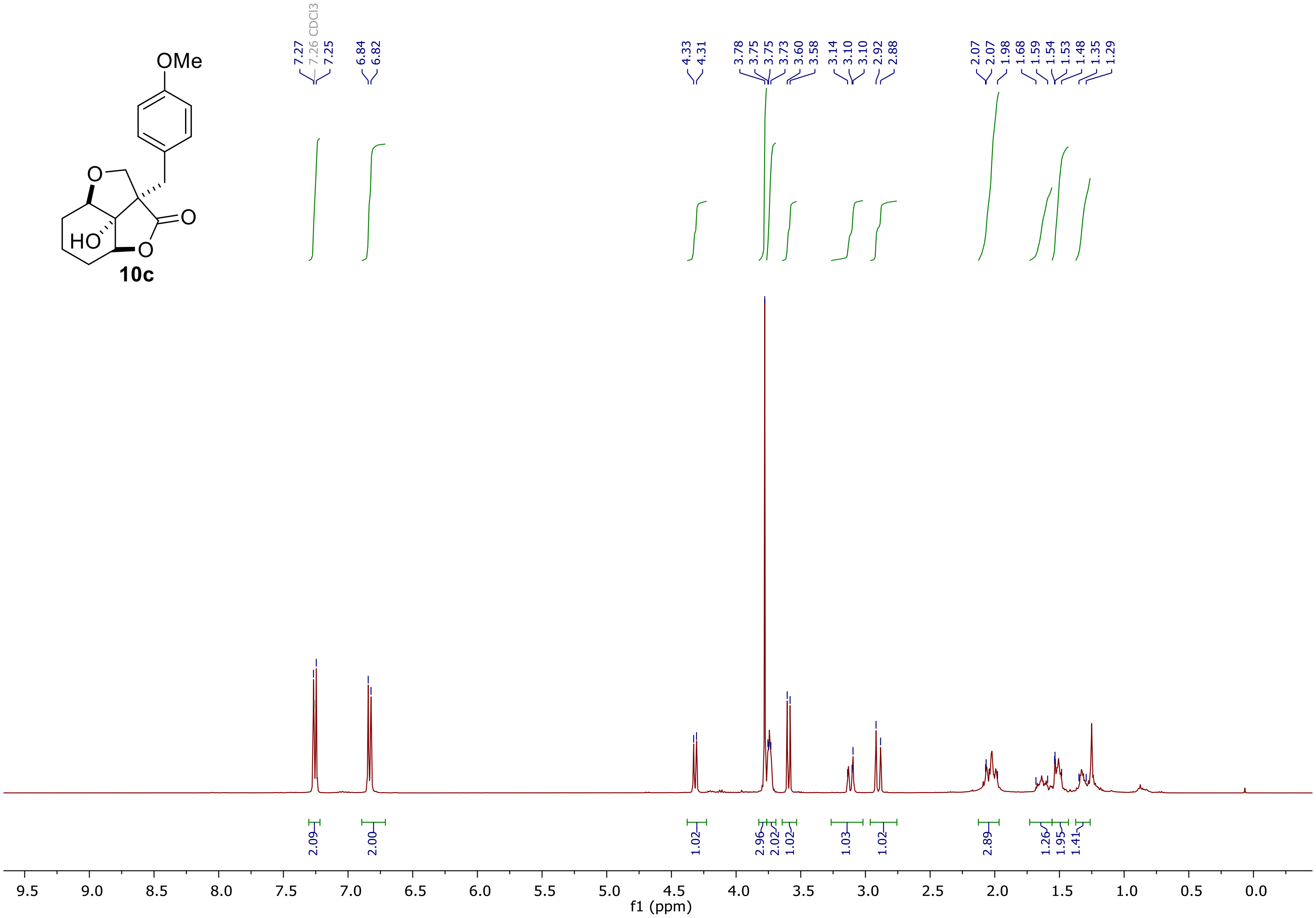

^{1}H NMR spectrum (400 MHz, $CDCl_3$) of compound **10c**

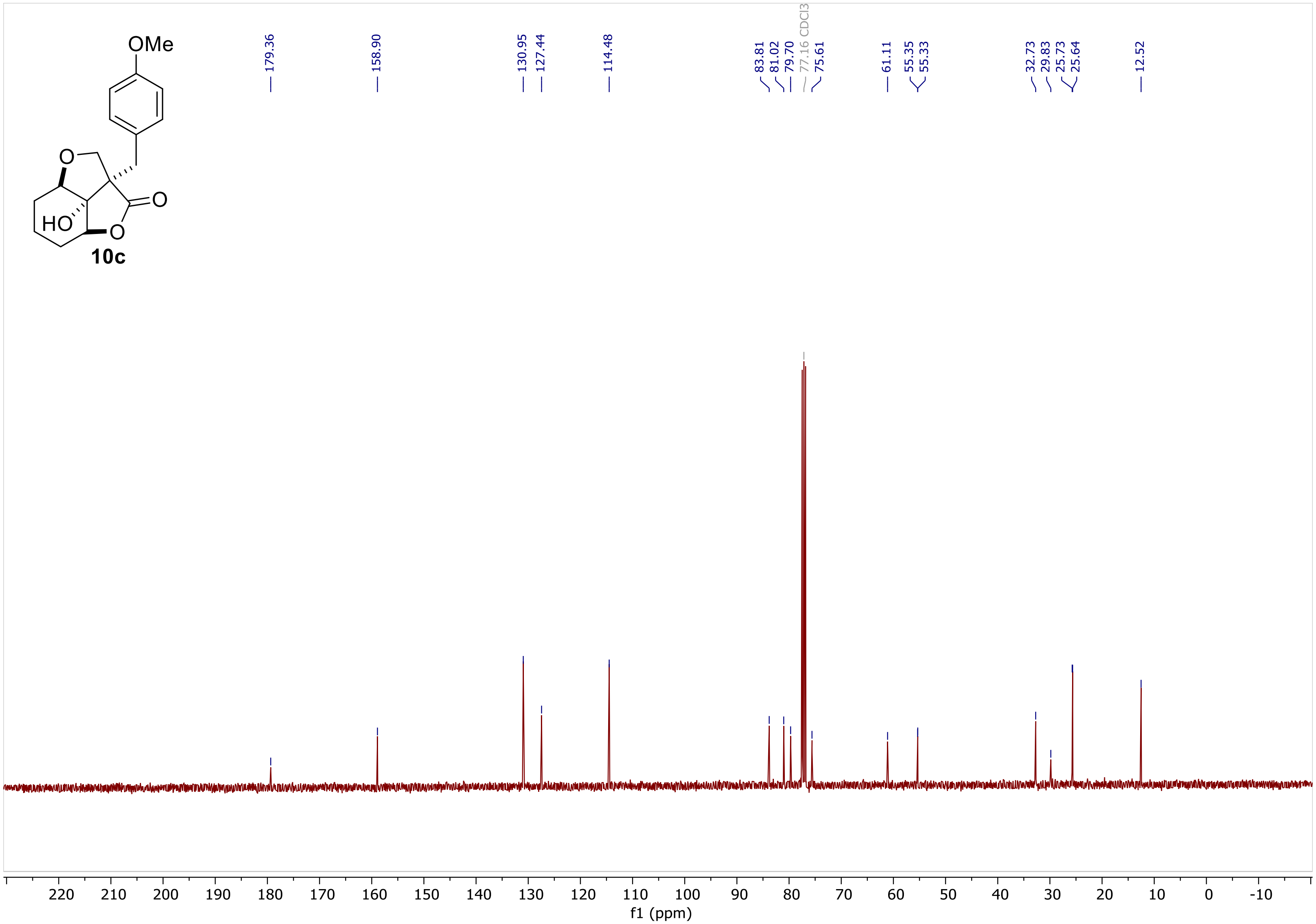

^{13}C NMR spectrum (101 MHz, $CDCl_3$) of compound **10c**

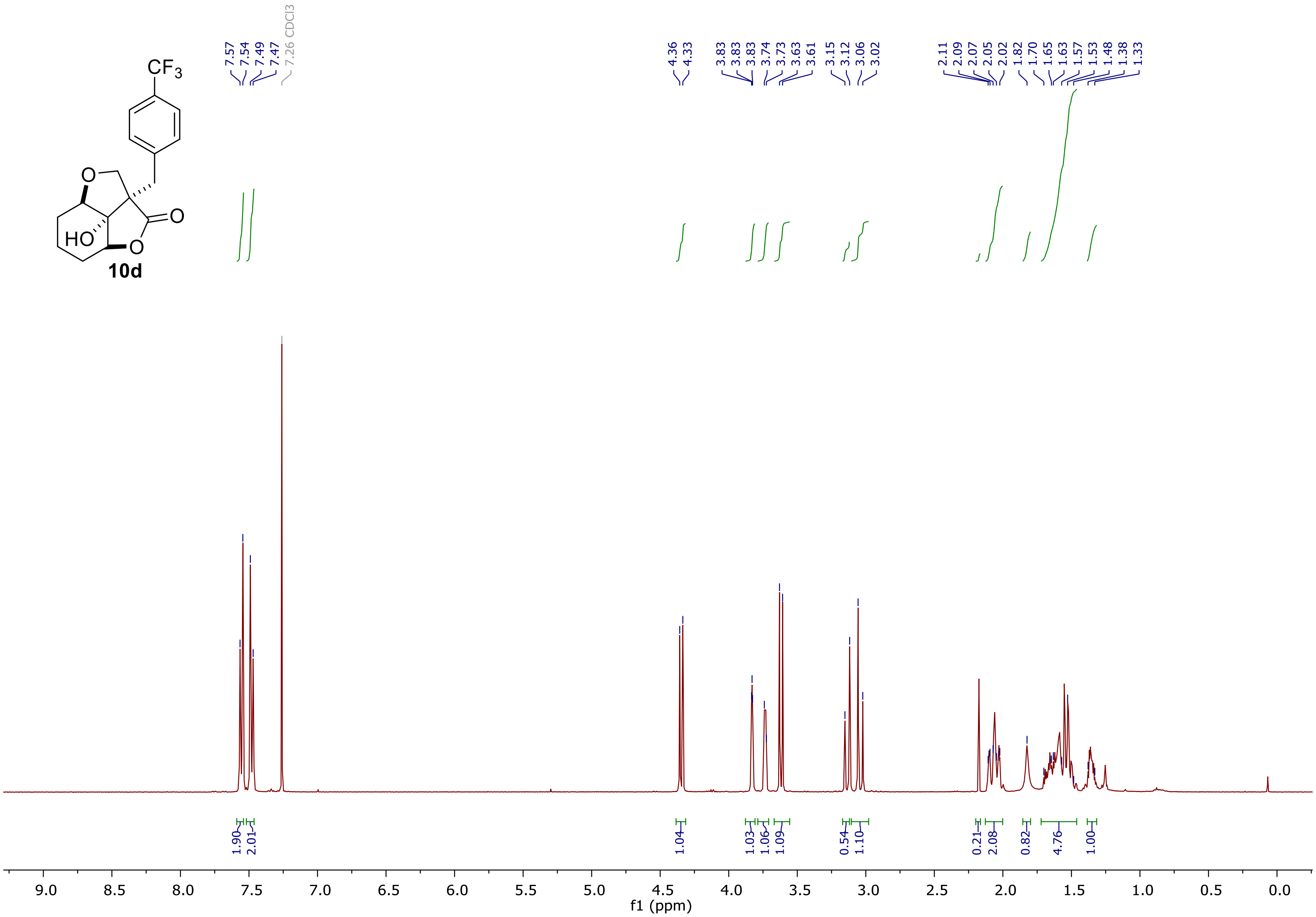

^{1}H NMR spectrum (400 MHz, $CDCl_3$) of compound **10d**

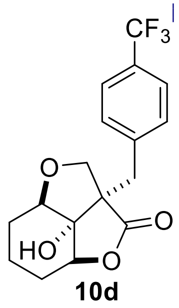

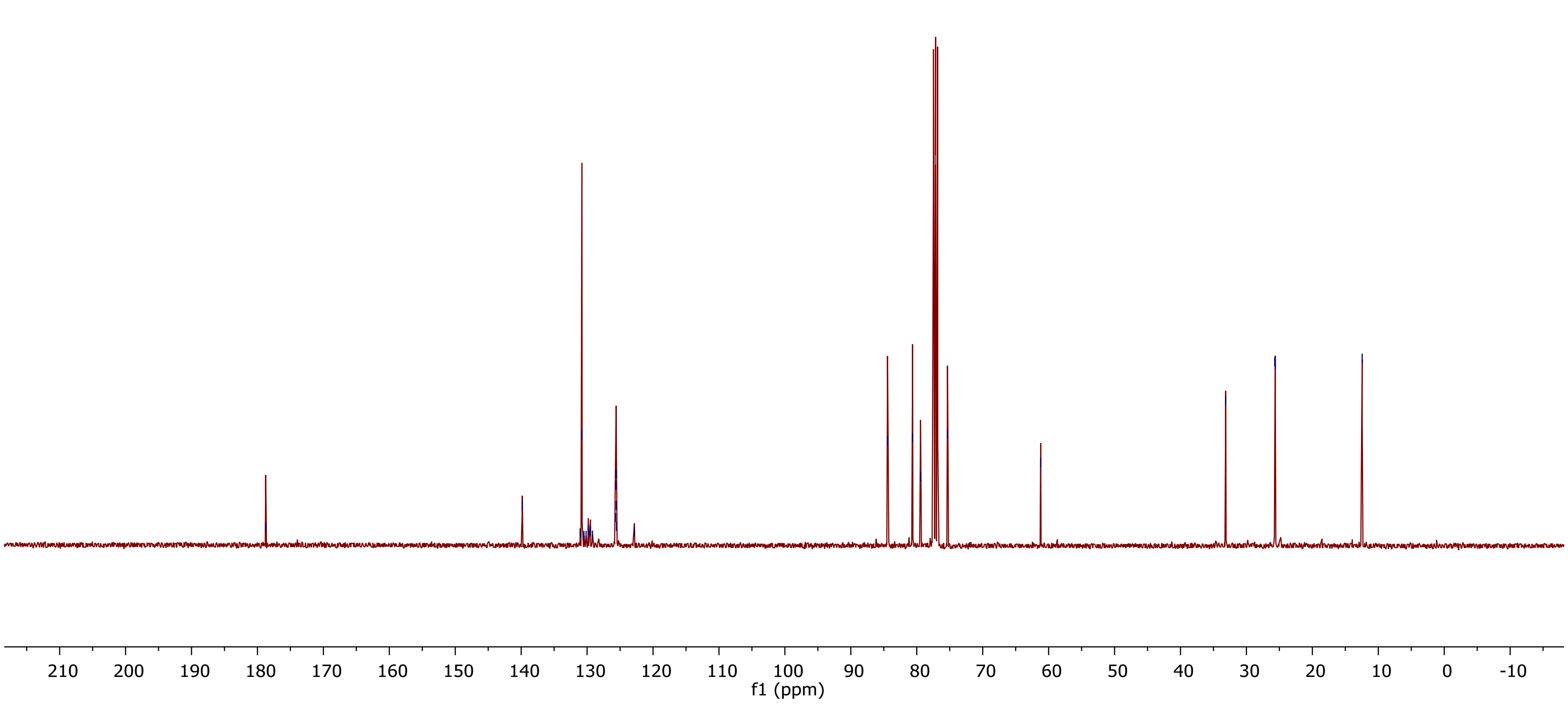

^{13}C NMR spectrum (101 MHz, $CDCl_3$) of compound **10d**

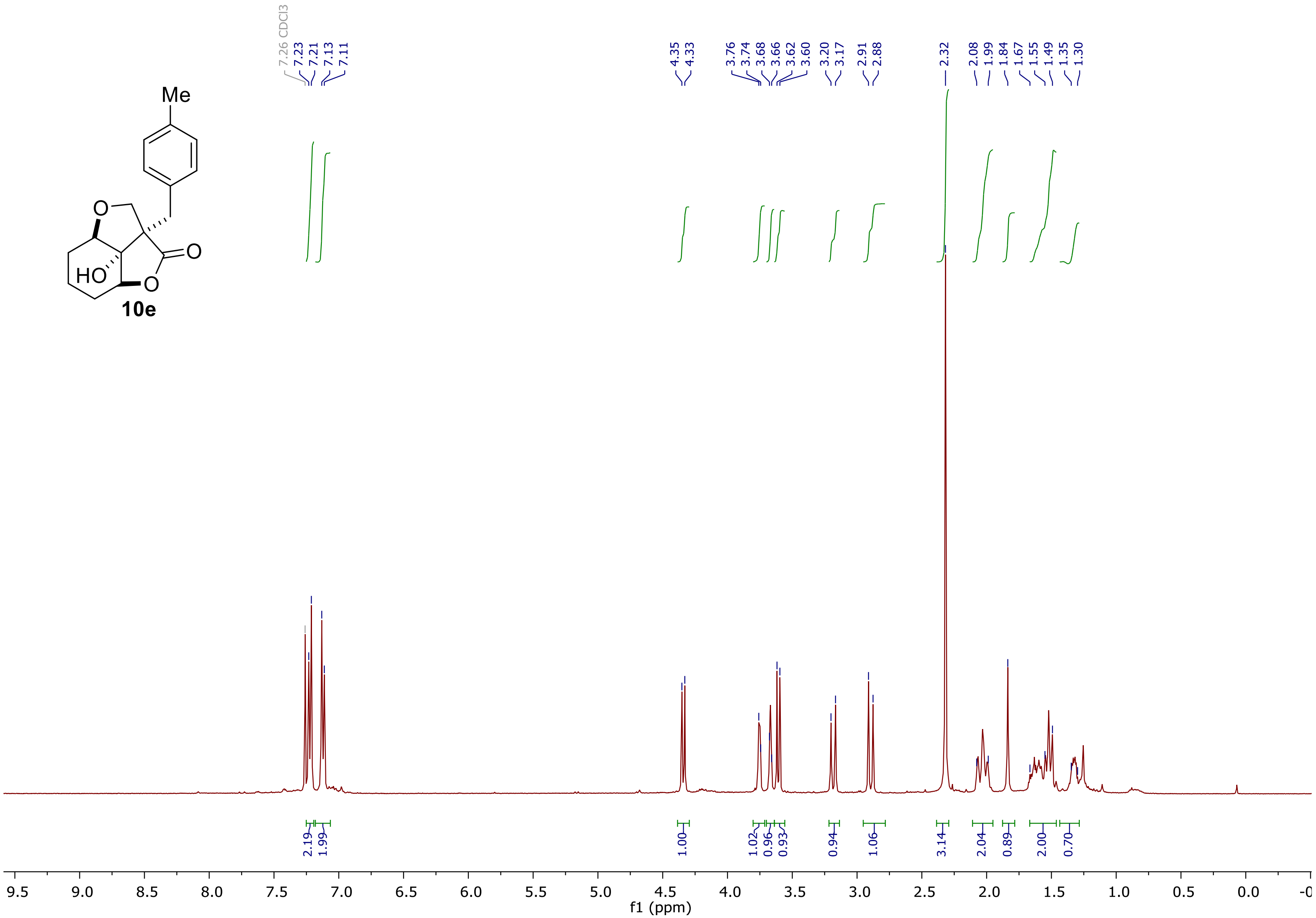

^{1}**H NMR** spectrum (400 MHz, $CDCl_3$) of compound **10e**

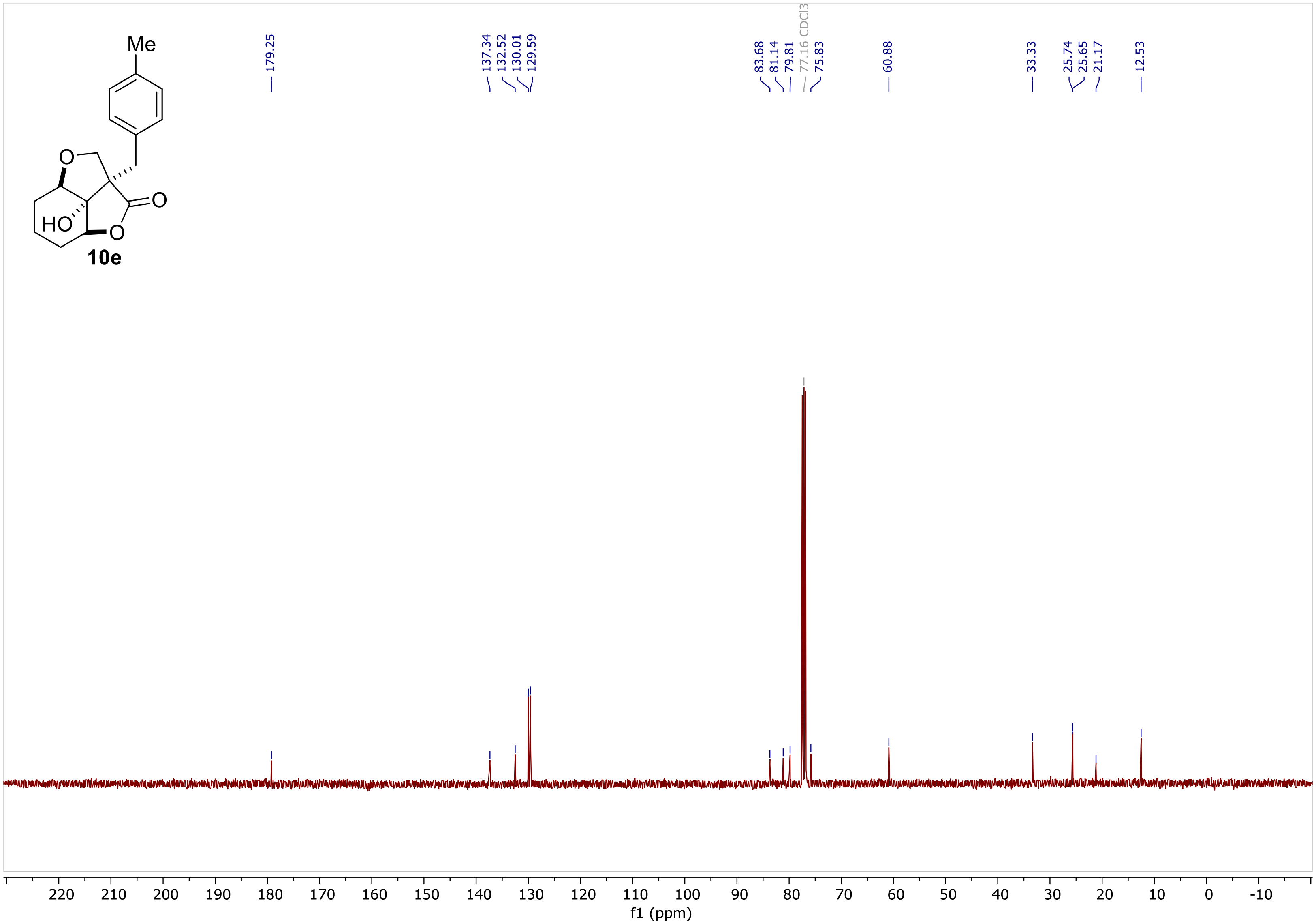

^{13}C NMR spectrum (101 MHz, $CDCl_3$) of compound **10e**

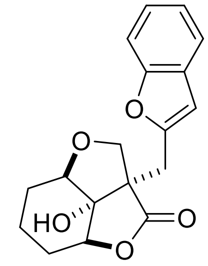

10f

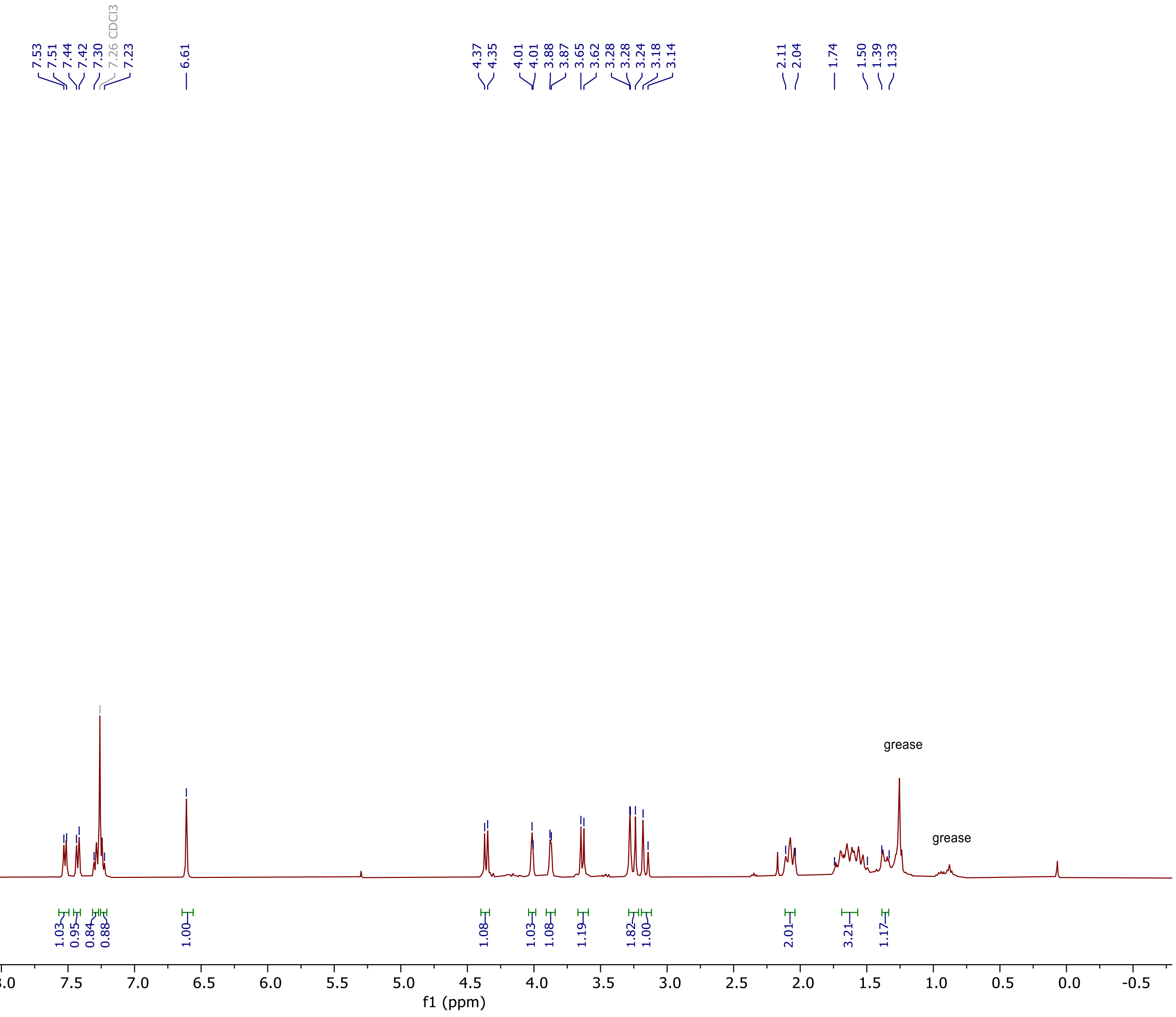

^{1}H NMR spectrum (400 MHz, $CDCl_3$) of compound **10f**

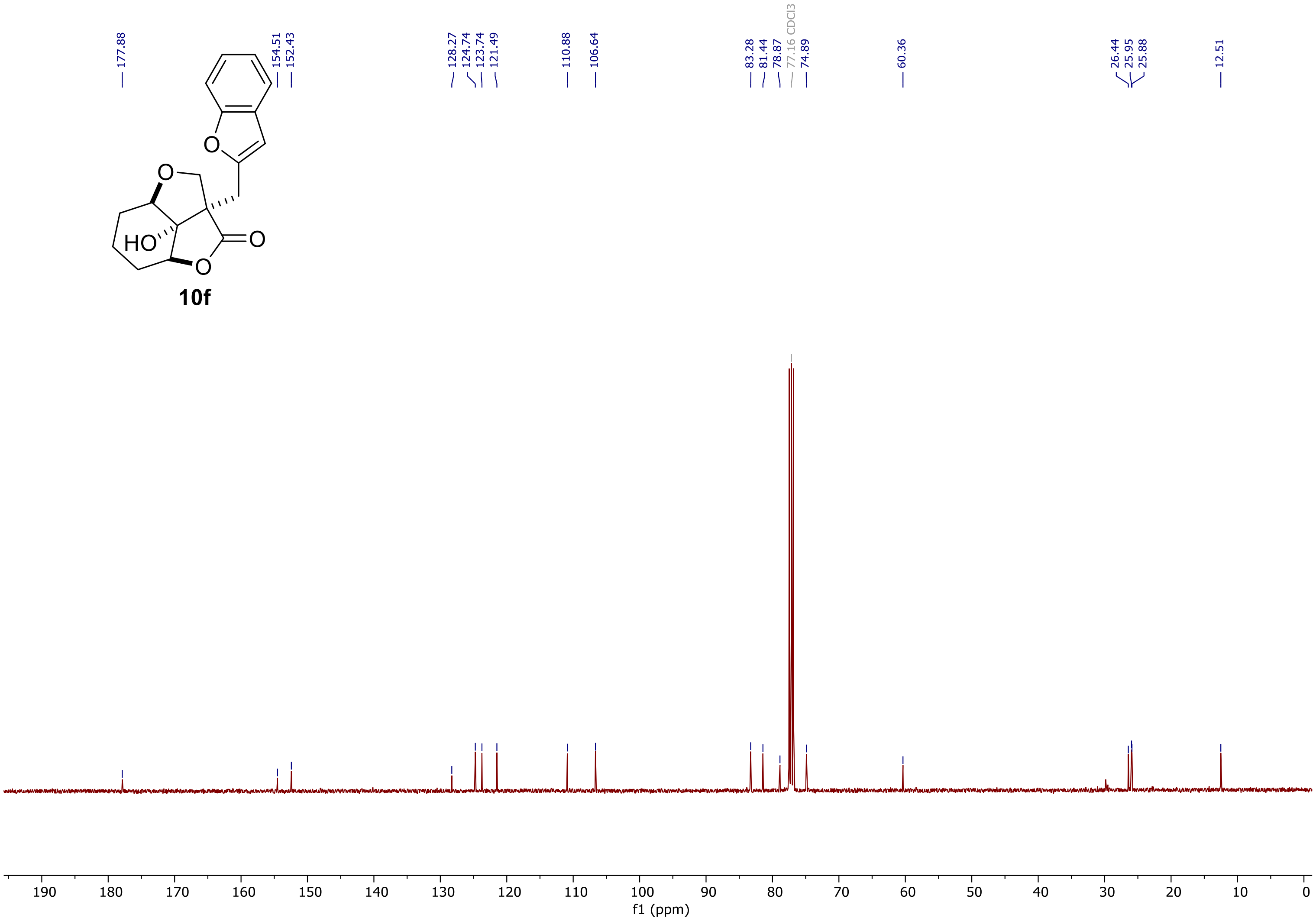

^{13}C NMR spectrum (101 MHz, $CDCl_3$) of compound **10f**

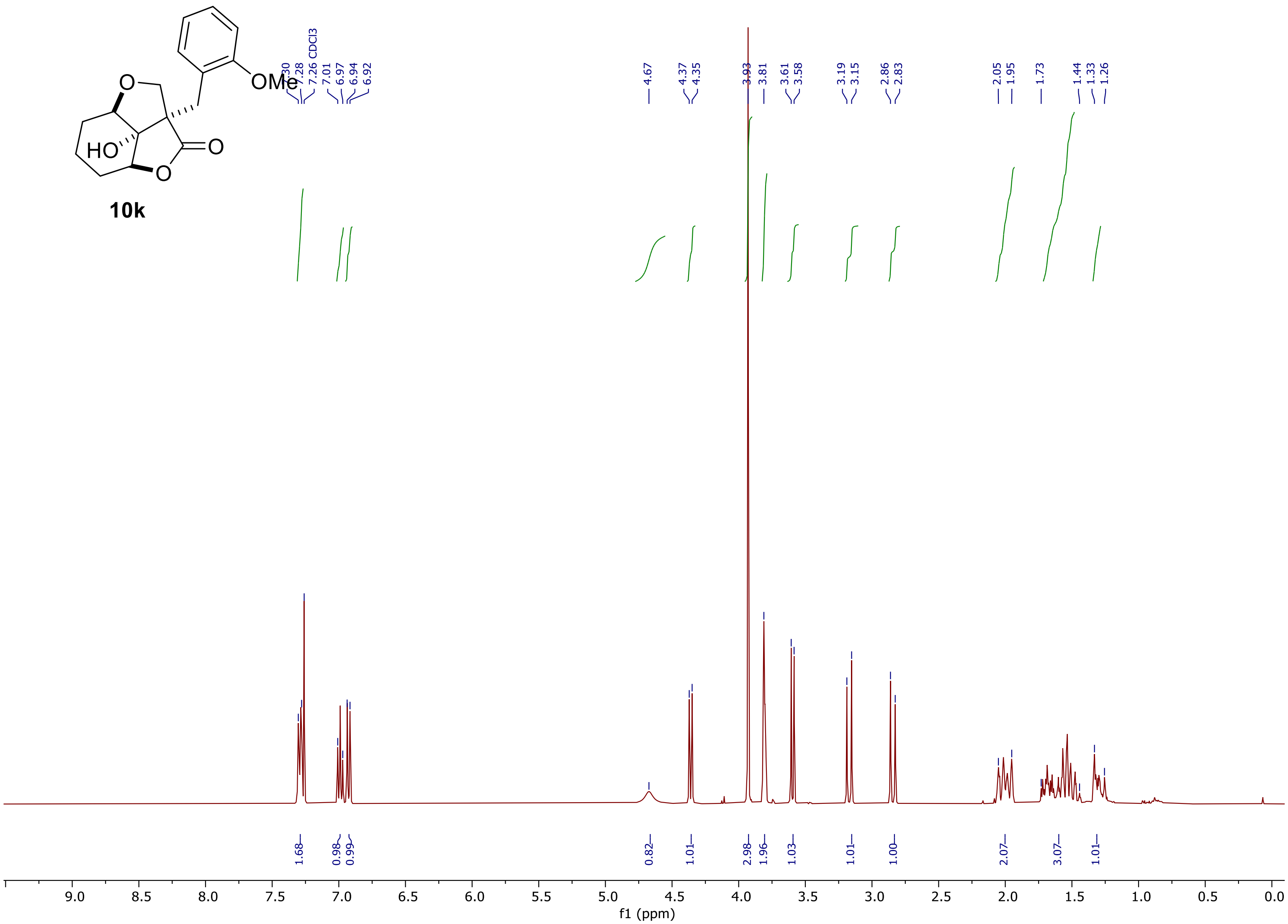

^{1}**H NMR** spectrum (400 MHz, $CDCl_3$) of compound **10k**

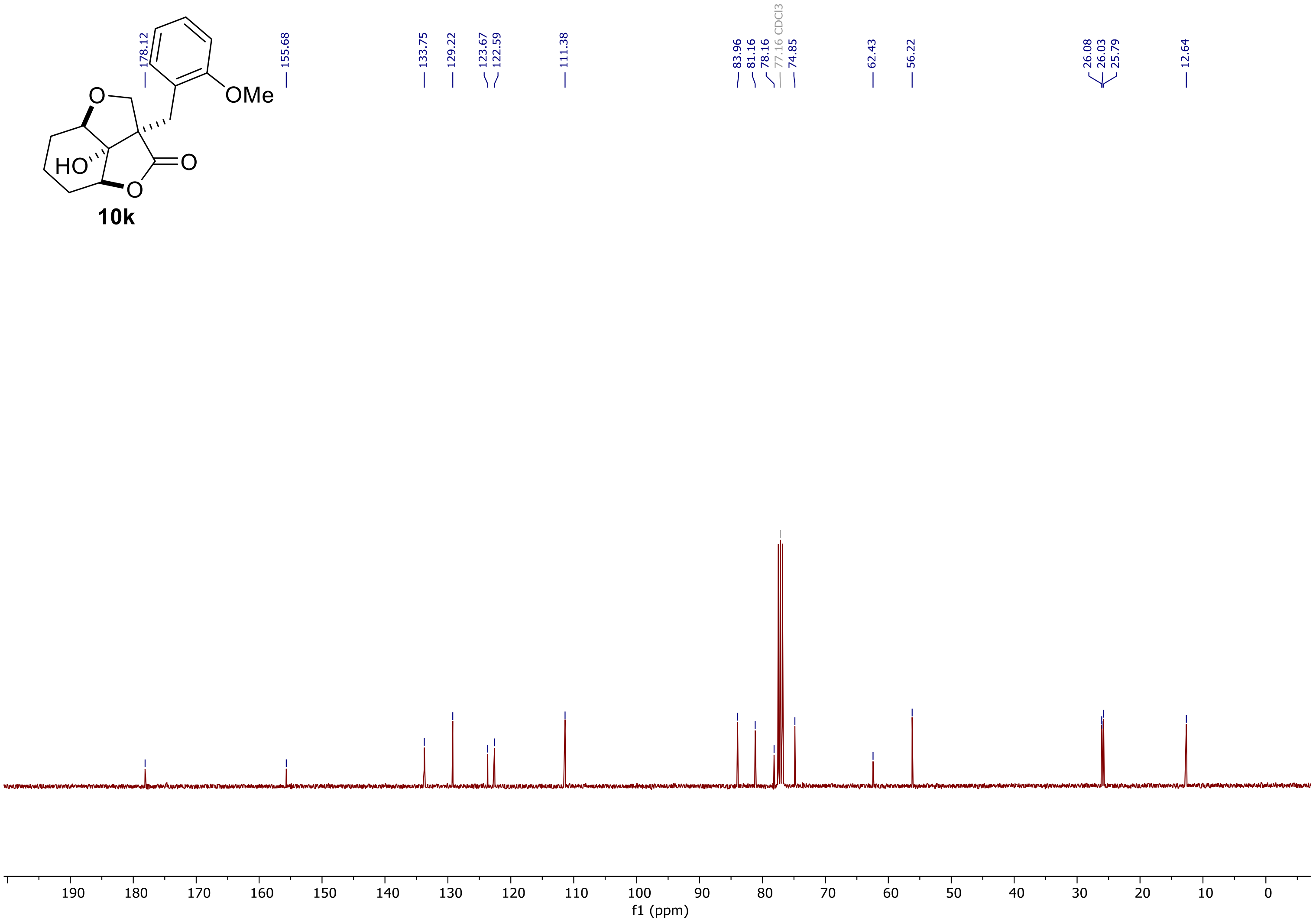

^{13}C NMR spectrum (101 MHz, $CDCl_3$) of compound **10k**

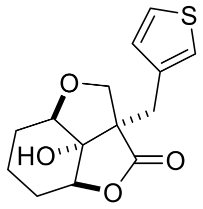

10m

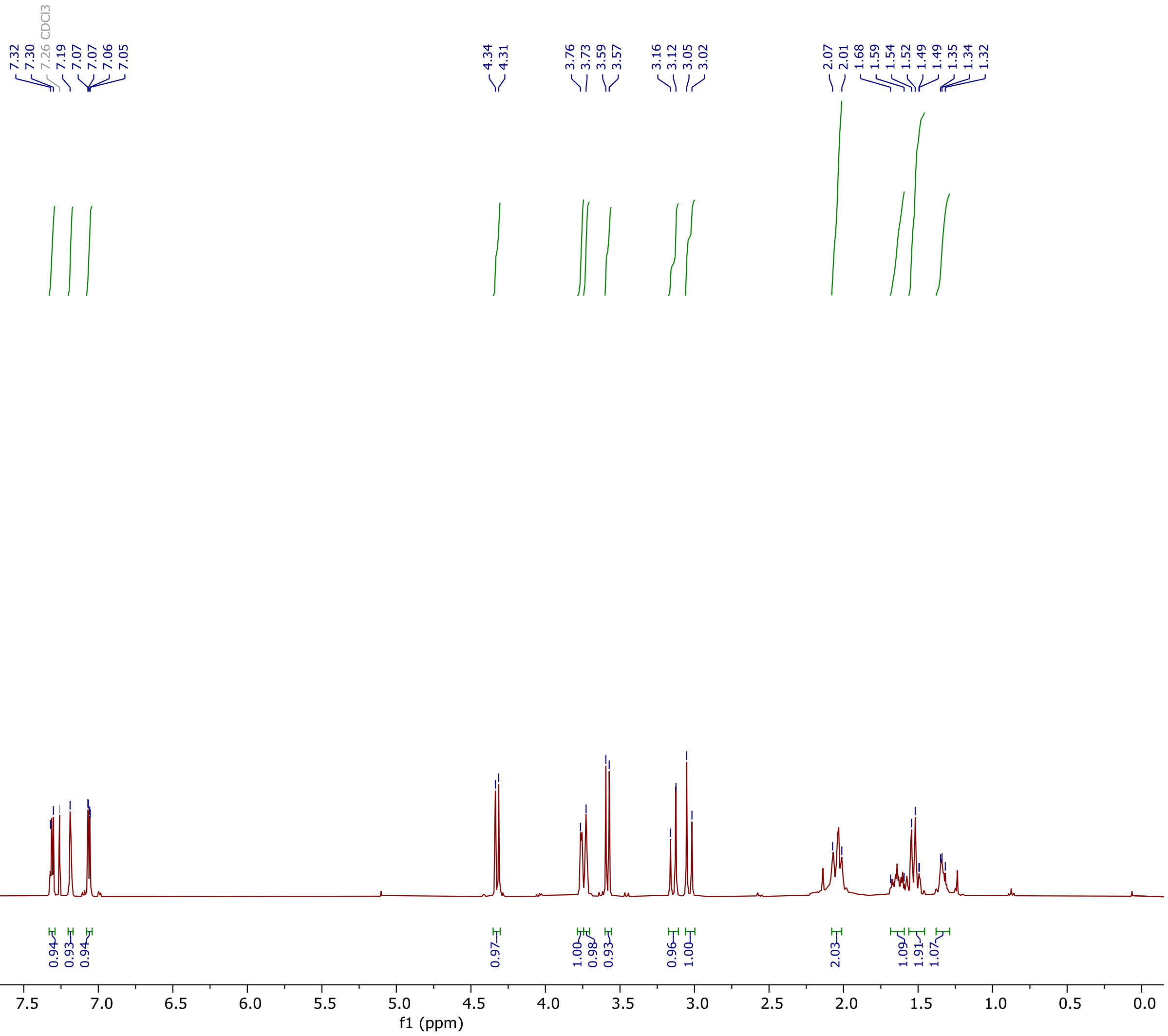

^{1}H NMR spectrum (400 MHz, $CDCl_3$) of compound **10m**

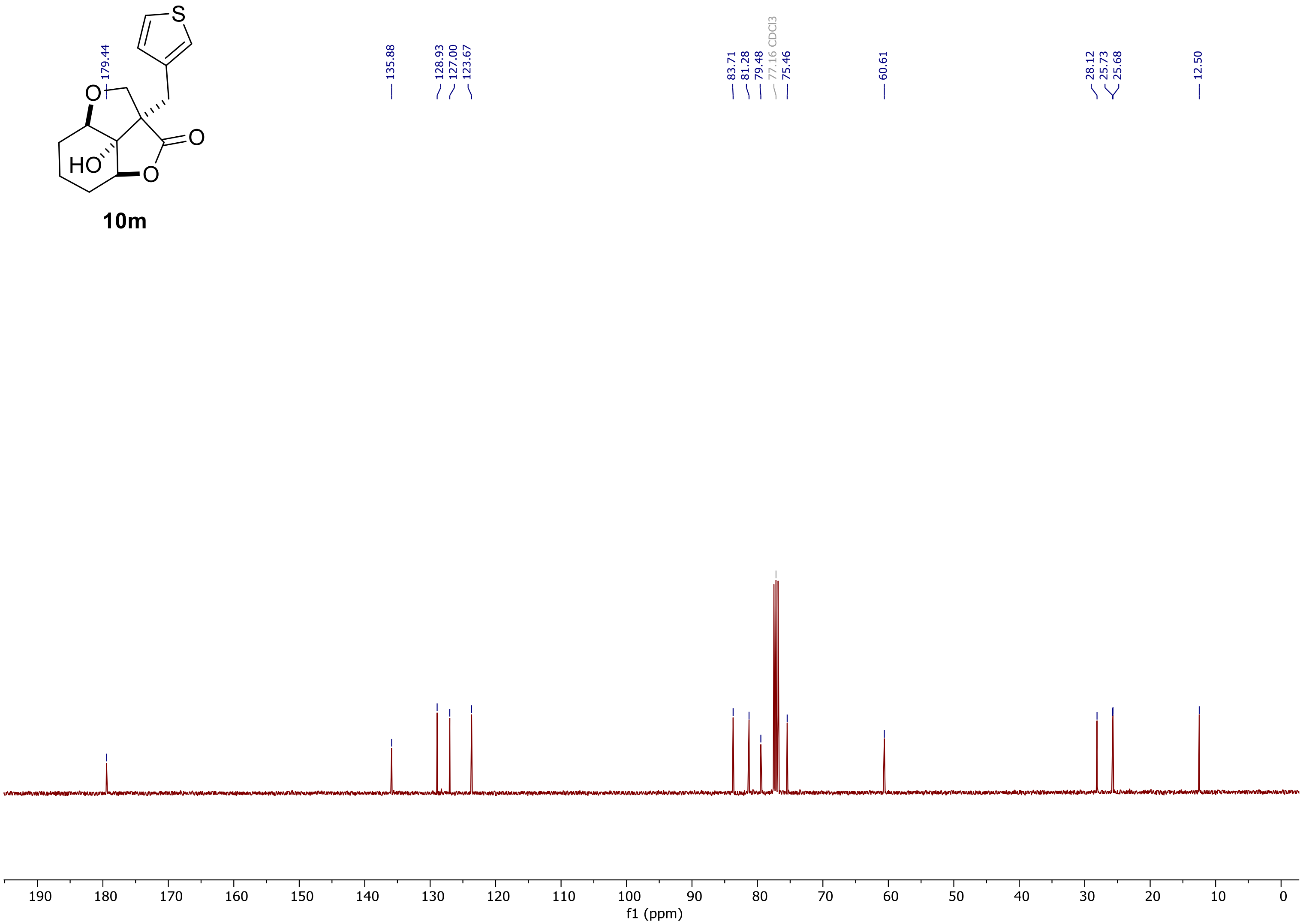

^{13}C NMR spectrum (101 MHz, $CDCl_3$) of compound **10m**

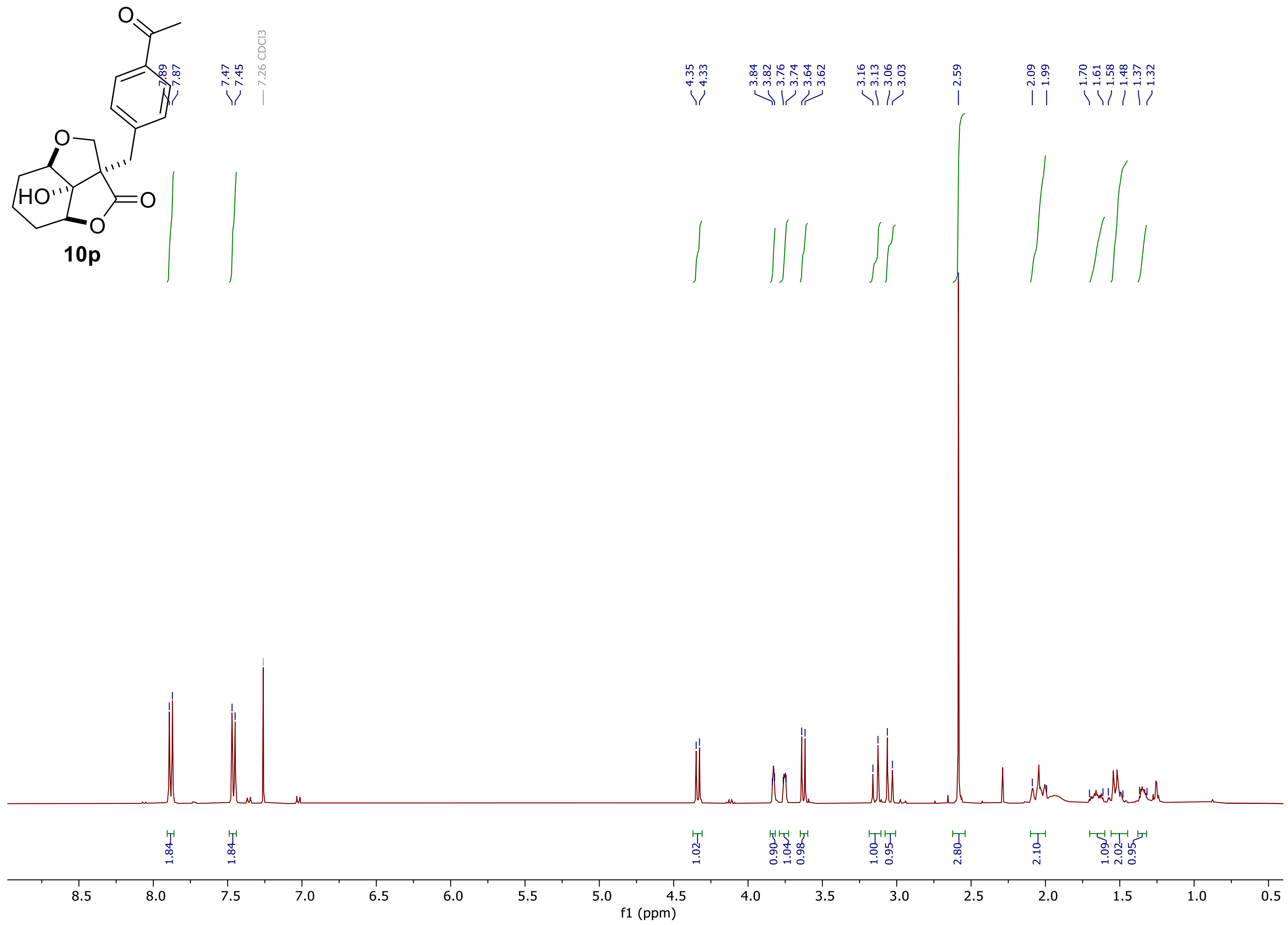

^{1}H NMR spectrum (400 MHz, $CDCl_3$) of compound **10p**

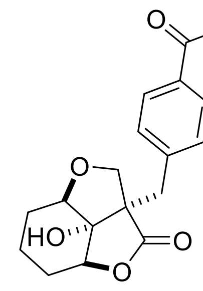

10p

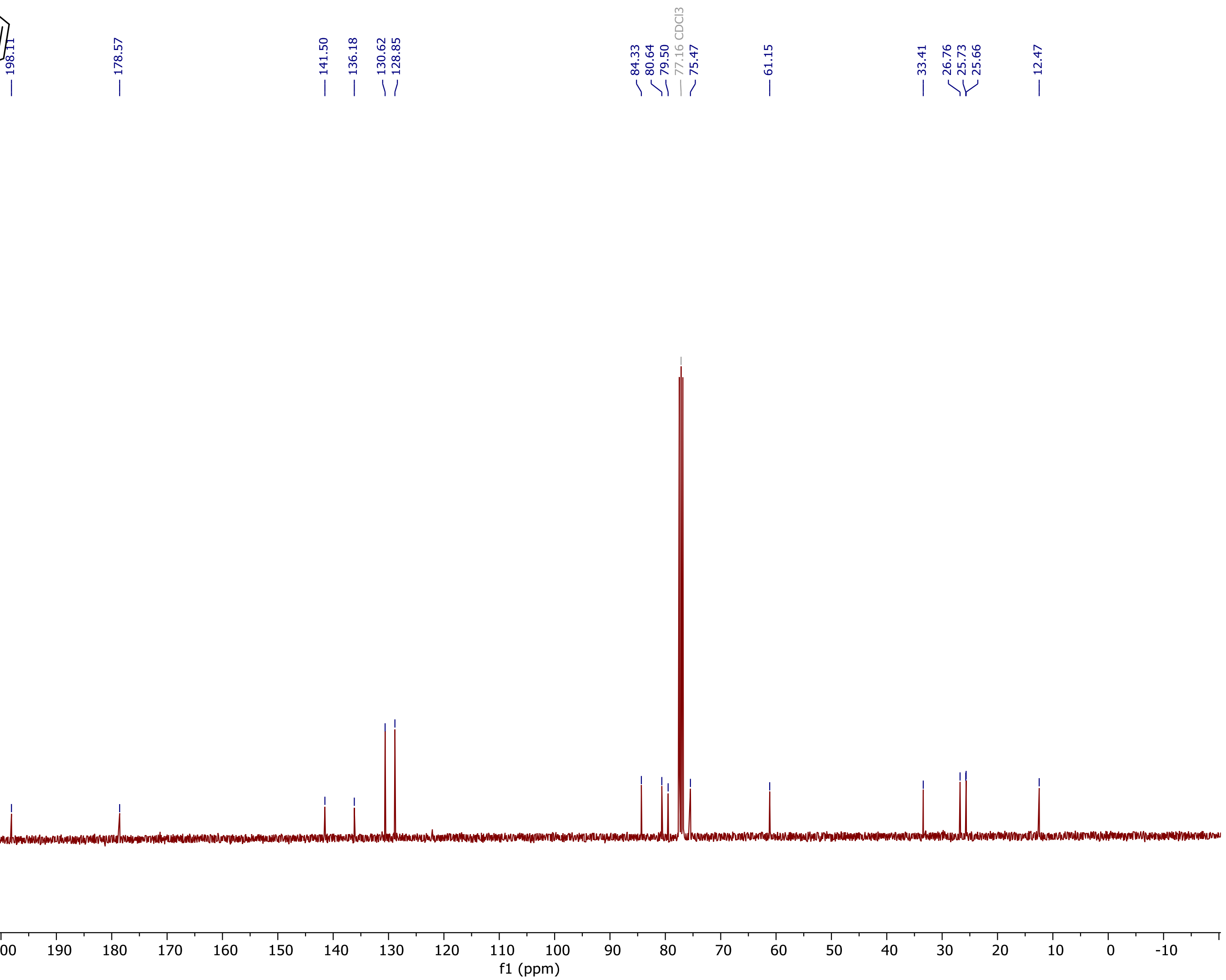

^{13}C NMR spectrum (101 MHz, $CDCl_3$) of compound **10p**

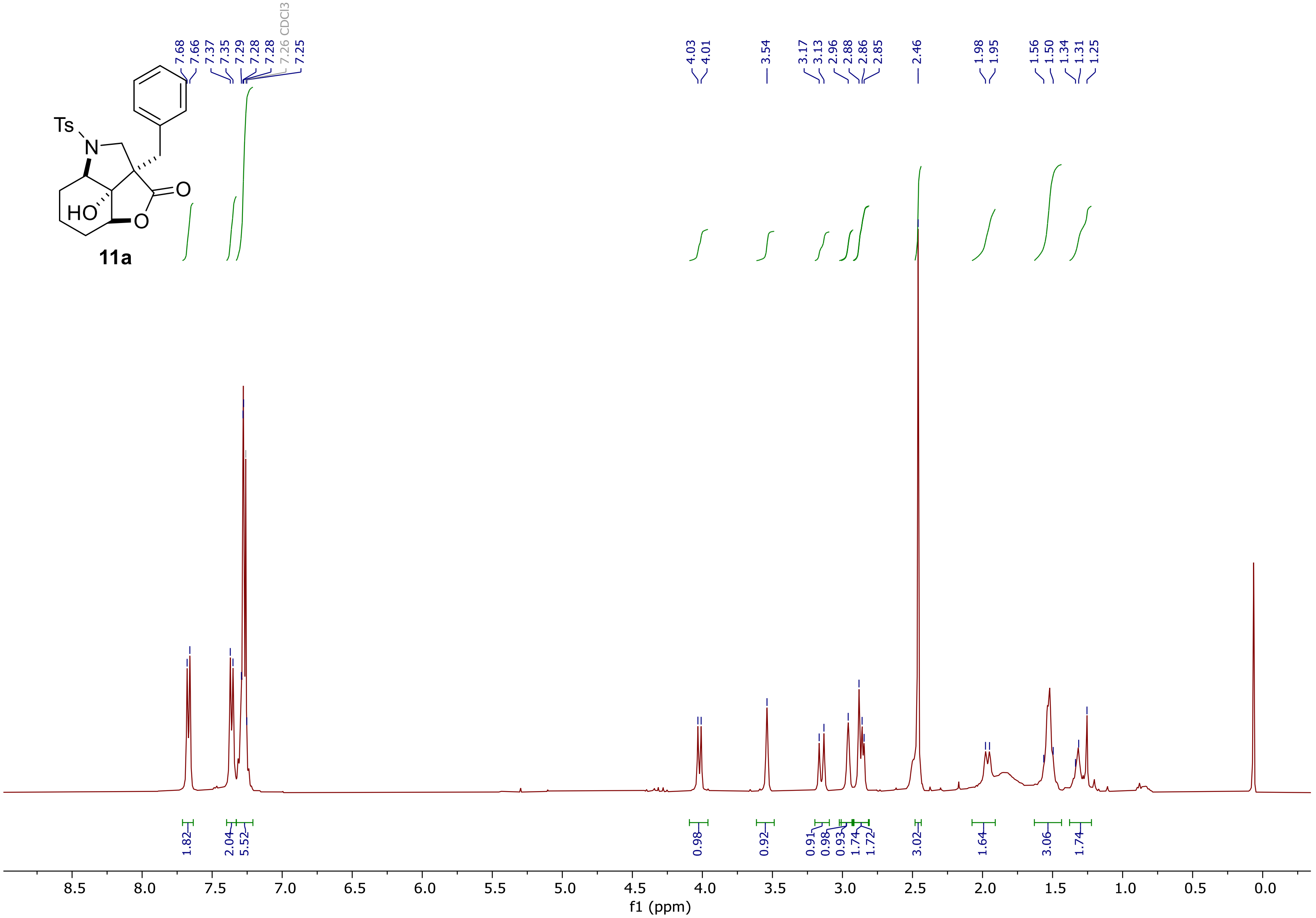

^{1}H NMR spectrum (400 MHz, $CDCl_3$) of compound **11a**

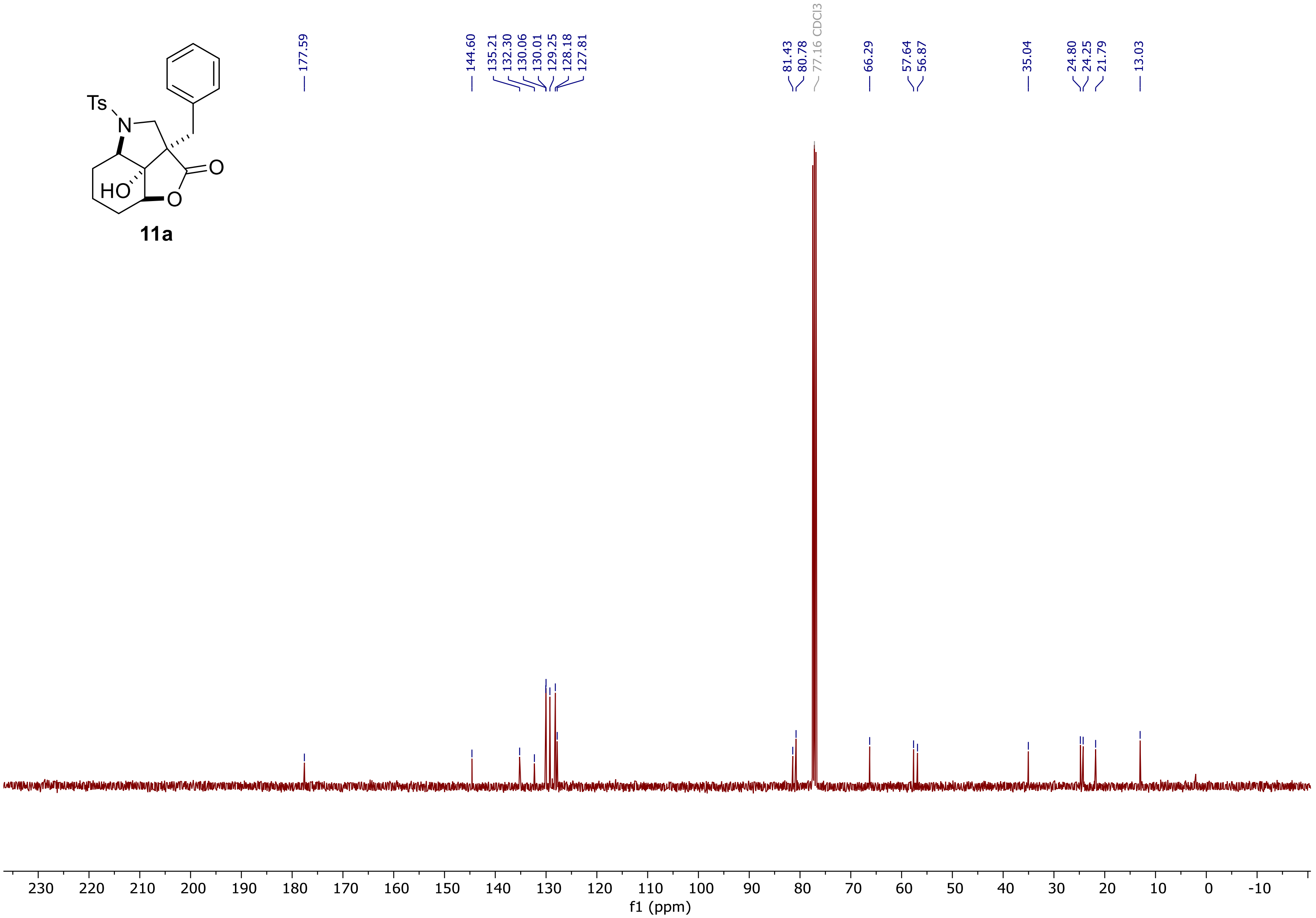

^{13}C NMR spectrum (101 MHz, $CDCl_3$) of compound **11a**

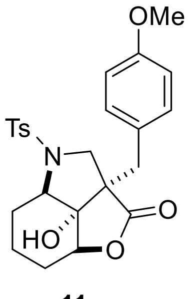

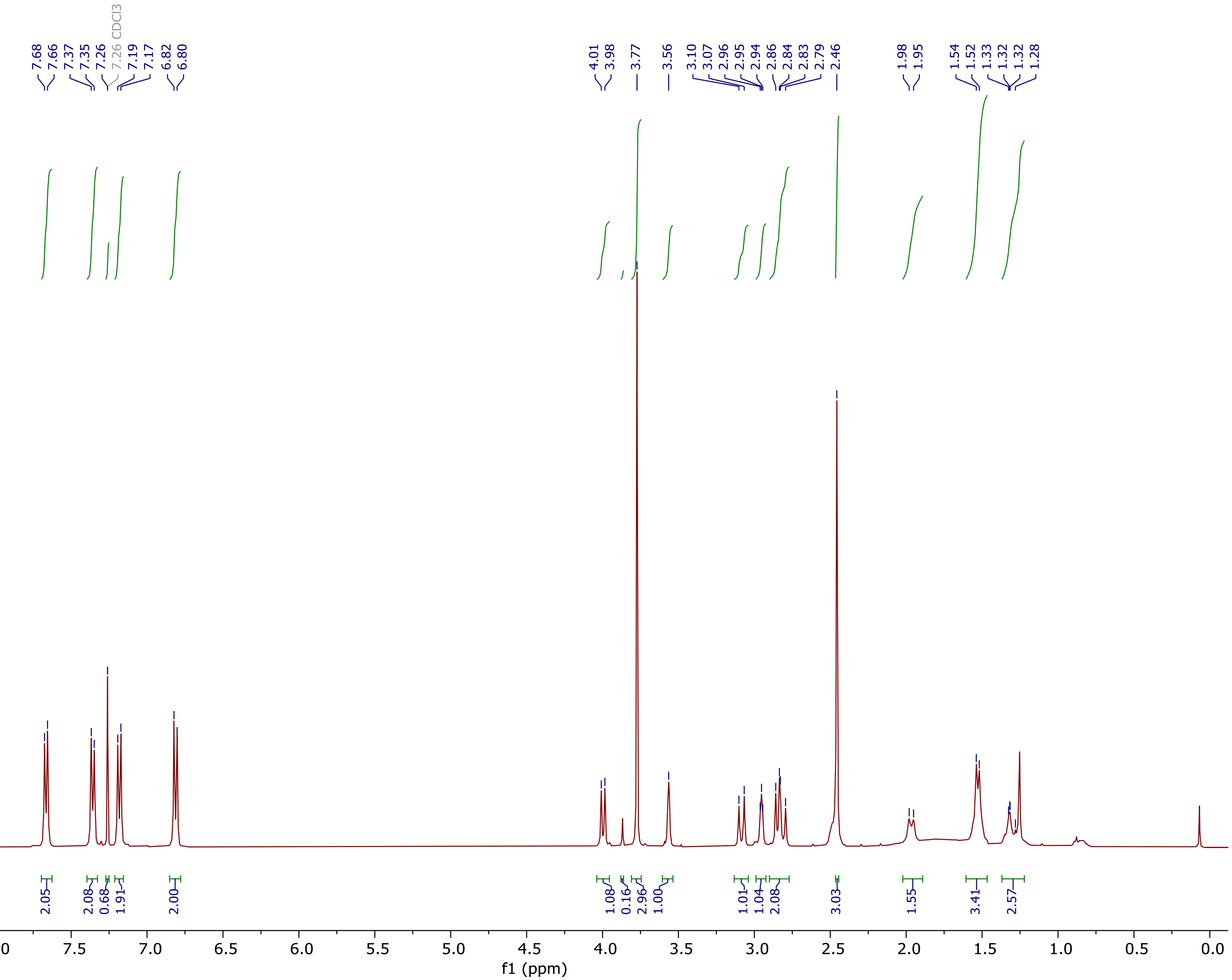

^{1}H NMR spectrum (400 MHz, $CDCl_3$) of compound **11c**

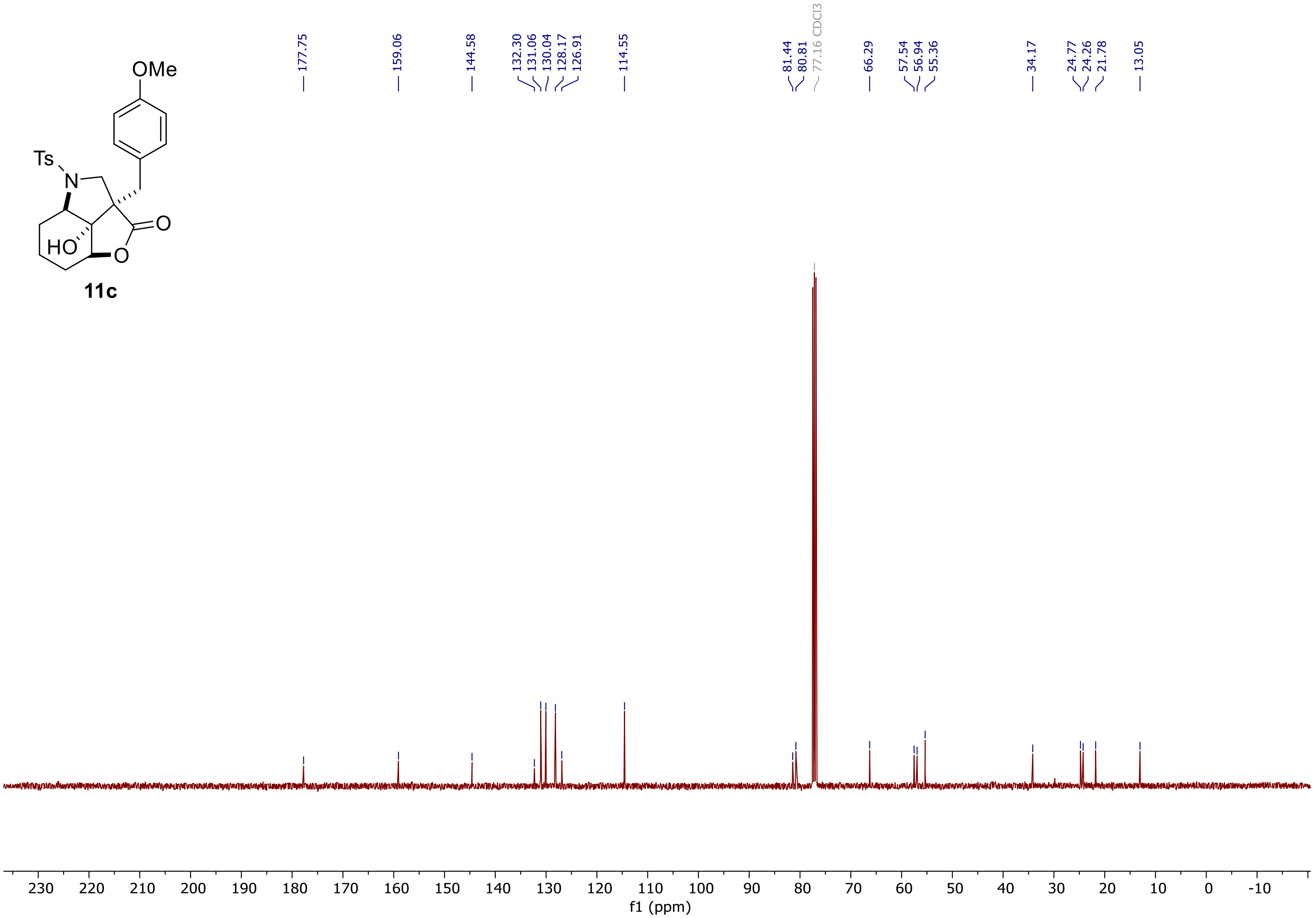

^{13}C NMR spectrum (101 MHz, $CDCl_3$) of compound **11c**

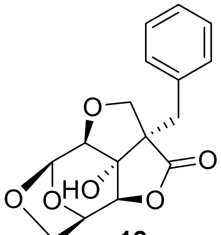

12a

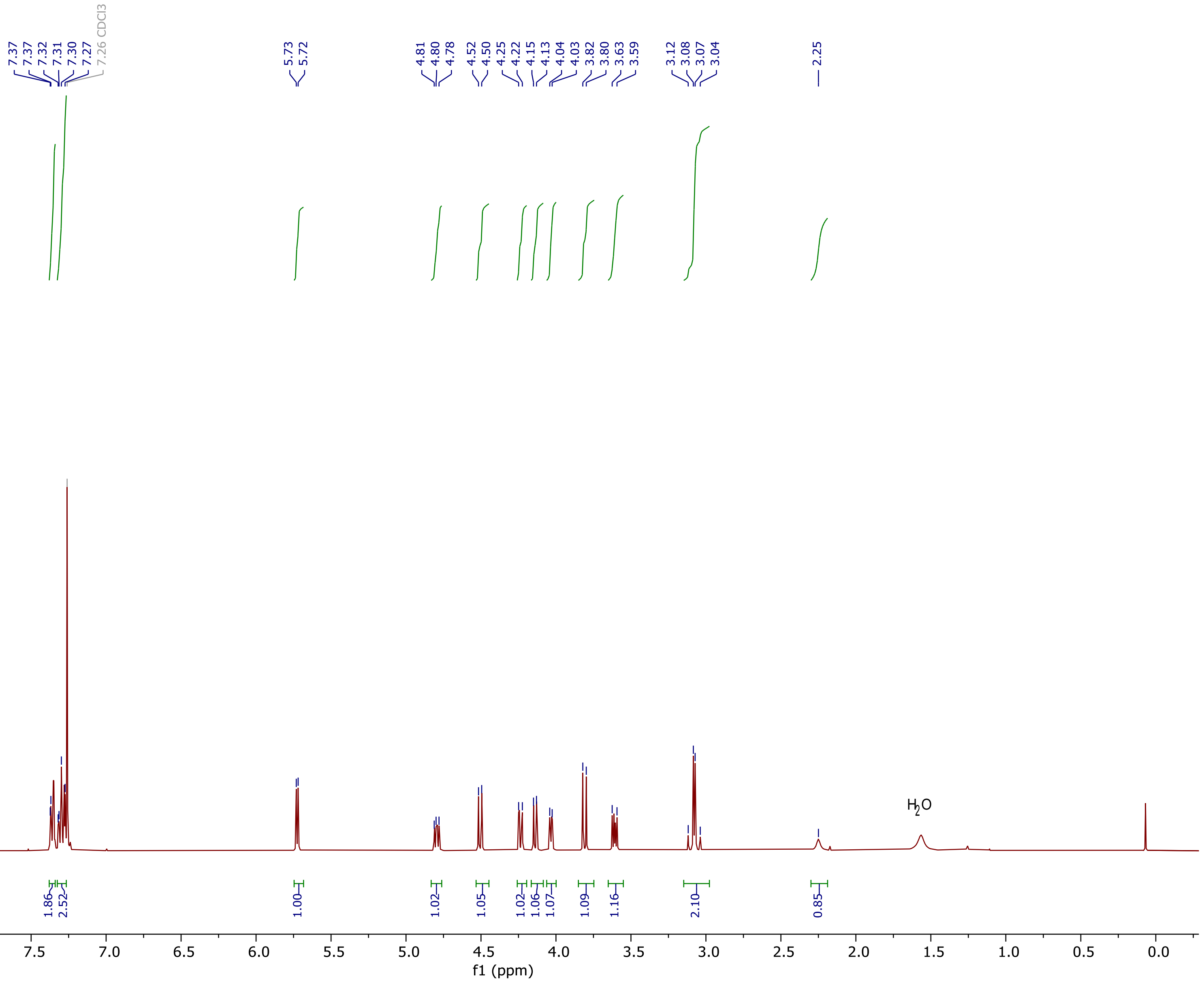

^{1}H NMR spectrum (400 MHz, $CDCl_3$) of compound **15a**

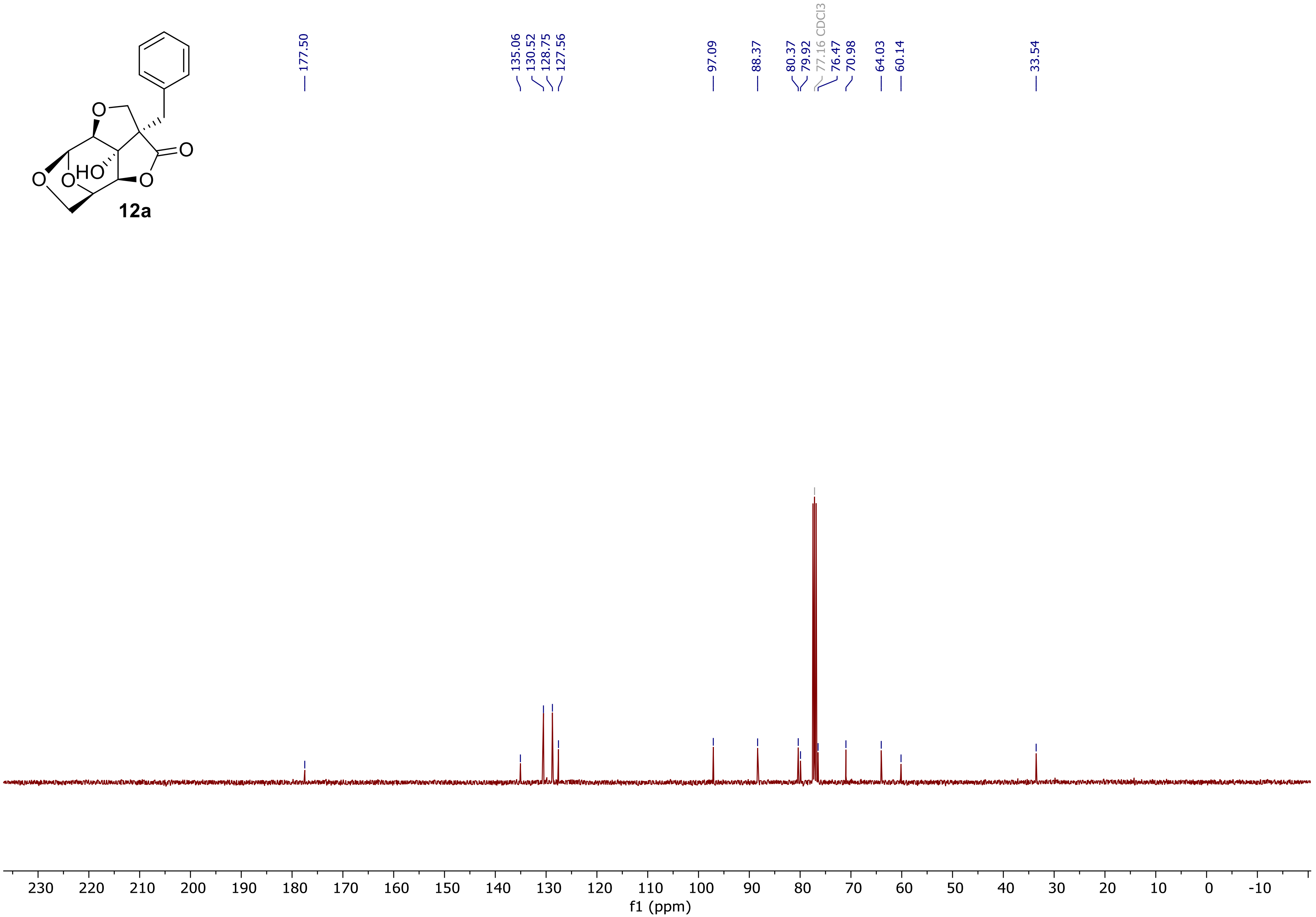

^{13}C NMR spectrum (101 MHz, $CDCl_3$) of compound **12a**

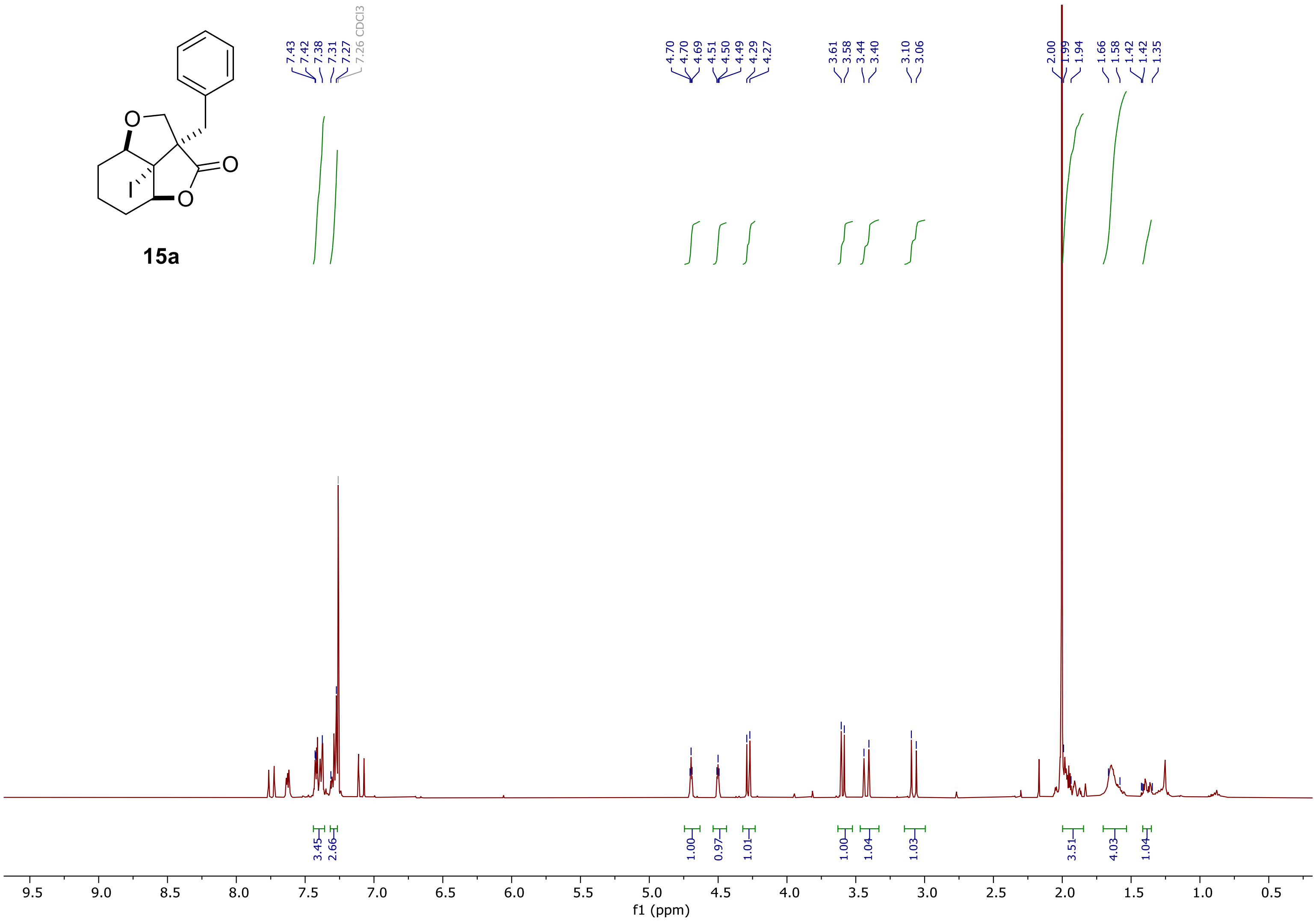

[1]**H NMR** spectrum (400 MHz, $CDCl_3$) of compound **15a**

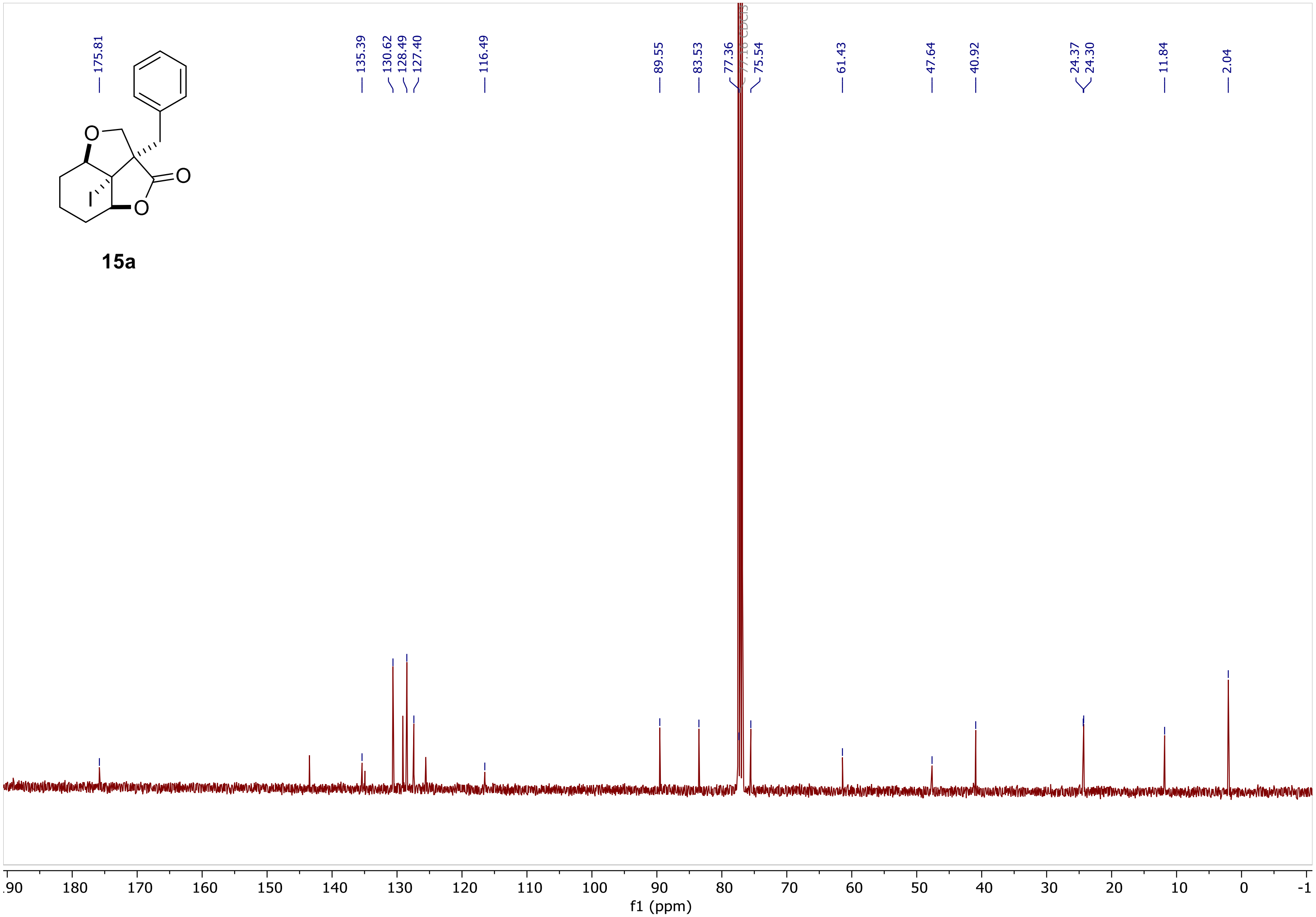

^{13}**C NMR** spectrum (101 MHz, $CDCl_3$) of compound **15a**

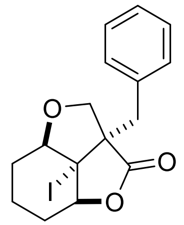

15a

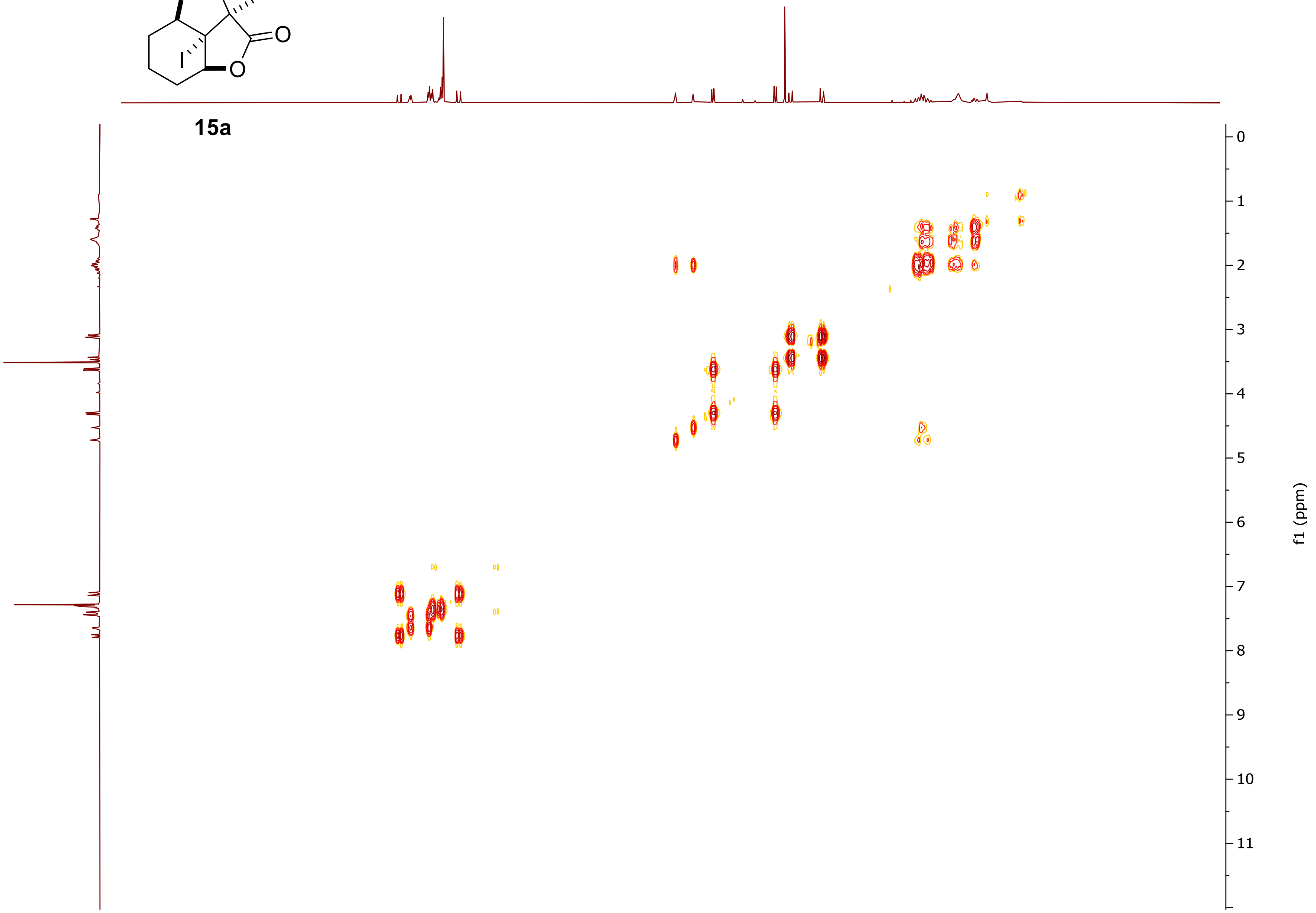

2D COSY Spectrum (400 MHz, $CDCl_3$) of compound **15a**

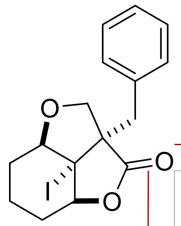

15a

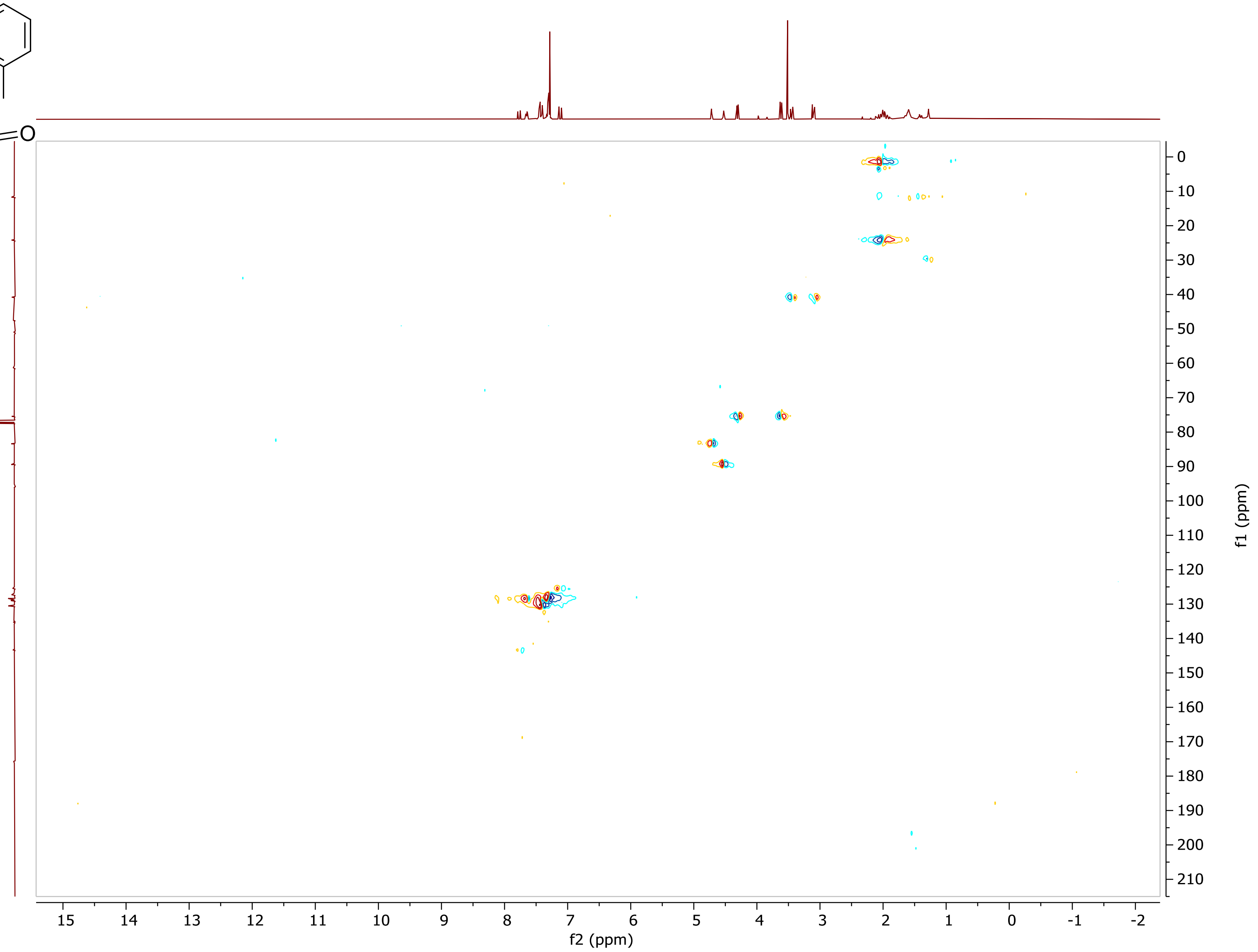

2D HSQC Spectrum (400 MHz, $CDCl_3$) of compound **15a**
